# Contribution of copy number variants to schizophrenia in East Asian populations

**DOI:** 10.1038/s41588-026-02732-6

**Published:** 2026-09-11

**Authors:** Yu Chen, Qidi Feng, Max Lam, Mingrui Yu, Yaoyao Sun, Cong Huai, Bimal Jana, Jack Fu, Calwing Liao, Robert Ye, Soyeon Kim, Justin D. Tubbs, Omar Shanta, Bhooma Thiruvahindrapuram, Yawen Jen, Guorui Zhao, Jess Wang, Stanley Global Asia Initiatives, Cong Huai, Sibylle G. Schwab, Rene Kahn, Kyung-Sue Hong, Ming Tsuang, Masanari Itokawa, Jianjun Liu, Stephen J. Glatt, Nakao Iwata, Masashi Ikeda, Jimmy Lee, Yunfeng Ruan, Ruize Liu, Yasue Horiuchi, Byung-Dae Lee, Eun-Jeong Joo, Woojae Myung, Kyooseob Ha, Hong-Hee Won, Ji-Hyun Baek, Young-Chul Chung, Sung-Wan Kim, Dieter B. Wildenauer, Agung Kusumawardhani, Hai-Gwo Hwu, Kang Sim, Akitoyo Hishimoto, Ikuo Otsuka, Ichiro Sora, Tomoko Toyota, Takeo Yoshikawa, Hiroshi Kunugi, Kotaro Hattori, Sayuri Ishiwata, Shusuke Numata, Tetsuro Ohmori, Makoto Arai, Yuji Ozeki, Kumiko Fujii, Se-Joo Kim, Heon-Jeong Lee, Yong-Min Ahn, Se-Hyun Kim, Kazufumi Akiyama, Kazutaka Shimoda, Makoto Kinoshita, Teruhiko Higuchi, Alice Zheng, Lu Shen, Takeo Saito, Yanhui Liao, Shanshan Chen, Toshiya Murai, Jun Miyata, Yujiro Yoshihara, Norio Ozaki, Itaru Kushima, Takahiro A. Kato, Shigenobu Kanba, Minoru Takebayashi, Shuken Boku, Toshifumi Kishimoto, Michihiro Toritsuka, Hiroki Ishiguro, Tetsuaki Arai, Masayuki Ide, Michio Suzuki, Yoichiro Takayanagi, Hideyuki Okano, Masaru Mimura, Hideto Niimura, Shogyoku Bun, Naohiro Arai, Wenzhao Shi, Feng Zhu, Zhenglin Guo, Akira Sawa, Jinsong Tang, Xiancang Ma, Wei J. Chen, Shengying Qin, Hailiang Huang, Juan Xu, Wenzhao Shi, Stephen W. Scherer, Feng Zhu, Chih-Min Liu, Zhenglin Guo, Daniel Howrigan, Mark Daly, Benjamin M. Neale, Akira Sawa, Jonathan Sebat, Michael E. Talkowski, Jinsong Tang, Xiancang Ma, Wei J. Chen, Shengying Qin, Weihua Yue, Tian Ge, Hailiang Huang

**Affiliations:** 1 Stanley Center for Psychiatric Research, Broad Institute of MIT and Harvard, Cambridge, MA USA; 2 Analytic and Translational Genetics Unit, Department of Medicine, Massachusetts General Hospital, Boston, MA USA; 3 Department of Medicine, Harvard Medical School, Boston, MA USA; 4 Institute of Mental Health, Singapore, Singapore; 5 Lee Kong Chian School of Medicine, Nanyang Technological University, Singapore, Singapore; 6 Peking University Sixth Hospital, Beijing, China; 7 Bio-X Institutes, Key Laboratory for the Genetics of Developmental and Neuropsychiatric Disorders (Ministry of Education), Shanghai Jiao Tong University, Shanghai, China; 8 Center for Genomic Medicine, Massachusetts General Hospital, Boston, MA USA; 9 Department of Neurology, Massachusetts General Hospital, Boston, MA USA; 10 Department of Neurology, Harvard Medical School, Boston, MA USA; 11 Department of Medicine, Kyung Hee University College of Medicine, Seoul, Republic of Korea; 12 Department of Psychiatry, Massachusetts General Hospital, Harvard Medical School, Boston, MA USA; 13 Psychiatric and Neurodevelopmental Genetics Unit, Center for Genomic Medicine, Massachusetts General Hospital, Boston, MA USA; 14 Department of Psychiatry, University of California San Diego, La Jolla, CA USA; 15 The Centre for Applied Genomics, The Hospital for Sick Children, Peter Gilgan Centre for Research and Learning, Toronto, Ontario Canada; 16 Institute of Epidemiology and Preventive Medicine, College of Public Health, National Taiwan University, Taipei, Taiwan; 17 Center for Neuropsychiatric Research, National Health Research Institutes, Zhunan, Taiwan; 18 Digital Health China Technologies Co., Beijing, China; 19 McLaughlin Centre and Department of Molecular Genetics, University of Toronto, Toronto, Ontario Canada; 20 Department of Psychiatry, The First Affiliated Hospital of Xi’an Jiaotong University, Xi’an, China; 21 Department of Psychiatry, National Taiwan University Hospital, Taipei, Taiwan; 22 Program in Medical and Population Genetics, Broad Institute of MIT and Harvard, Cambridge, MA USA; 23 Institute for Molecular Medicine Finland (FIMM), University of Helsinki, Helsinki, Finland; 24 Departments of Psychiatry, Neuroscience, Biomedical Engineering, Genetic Medicine and Mental Health, Johns Hopkins University School of Medicine and Bloomberg School of Public Health, Baltimore, MD USA; 25 Department of Psychiatry, Sir Run Run Shaw Hospital, School of Medicine, Zhejiang University, Hangzhou, China; 26 Sichuan Research Institute, Shanghai Jiao Tong University, Chengdu, China; 27 Peking University Sixth Hospital and Institute of Mental Health; NHC Key Laboratory of Mental Health and National Clinical Research Center for Mental Disorders; Institute of Advanced Clinical Medicine (Peking University), Beijing, China; 28 PKU-IDG/McGovern Institute for Brain Research (Peking University), Beijing, China; 29 School of Chemistry and Molecular Bioscience, University of Wollongong, Wollongong, New South Wales Australia; 30 Illawarra Health and Medical Research Institute, Wollongong, New South Wales Australia; 31 Department of Psychiatry, Icahn School of Medicine at Mount Sinai, New York, NY USA; 32 Department of Psychiatry, University of British Columbia, Vancouver, British Columbia Canada; 33 Department of Psychiatry and Behavioral Sciences, Tokyo Metropolitan Institute of Medical Science, Tokyo, Japan; 34 Genome Institute of Singapore, A*STAR, Singapore, Singapore; 35 Yong Loo Lin School of Medicine, National University of Singapore, Singapore, Singapore; 36 Department of Psychiatry and Behavioral Sciences, SUNY Upstate Medical University, Syracuse, NY USA; 37 Department of Neuroscience and Physiology, SUNY Upstate Medical University, Syracuse, NY USA; 38 Department of Psychiatry, Fujita Health University School of Medicine, Toyoake, Japan; 39 Department of Psychiatry, Pusan National University Hospital, Busan, Korea; 40 Department of Neuropsychiatry, School of Medicine, Eulji University, Daejeon, Korea; 41 Department of Psychiatry, Seoul National University College of Medicine, Seoul, Korea; 42 Department of Neuropsychiatry, Seoul National University Bundang Hospital, Seongnam, Korea; 43 Mood Disorders Center, Department of Psychiatry, University of British Columbia, Vancouver, British Columbia Canada; 44 Samsung Advanced Institute for Health Sciences and Technology (SAIHST), Sungkyunkwan University, Samsung Medical Center, Seoul, Korea; 45 Department of Psychiatry, Sungkyunkwan University, Samsung Medical Center, Seoul, Korea; 46 Department of Psychiatry, Jeonbuk National University Medical School, Jeonbuk, Korea; 47 Department of Psychiatry, Chonnam National University Medical School, Gwangju, Korea; 48 University of Western Australia, Perth, Western Australia Australia; 49 Department of Psychiatry, University of Indonesia, Jakarta, Indonesia; 50 Department of Psychiatry, National Taiwan University, Taipei, Taiwan; 51 Department of Psychiatry, Kobe University Graduate School of Medicine, Kobe, Japan; 52 Laboratory for Statistical Analysis, RIKEN Center for Integrative Medical Sciences, Yokohama, Japan; 53 Laboratory for Molecular Psychiatry, RIKEN Center for Brain Science, Wako, Japan; 54 National Institute of Neuroscience, National Center of Neurology and Psychiatry, Tokyo, Japan; 55 Department of Psychiatry, Institute of Biomedical Sciences, Tokushima University Graduate School, Tokushima, Japan; 56 Department of Psychiatry, Shiga University of Medical Science, Shiga, Japan; 57 Department of Psychiatry and Institute of Behavioral Science in Medicine, Yonsei University College of Medicine, Seoul, Korea; 58 Department of Psychiatry, Korea University College of Medicine, Seoul, Korea; 59 Department of Biological Psychiatry and Neuroscience, Dokkyo Medical University School of Medicine, Mibu, Japan; 60 Department of Psychiatry, Dokkyo Medical University School of Medicine, Mibu, Japan; 61 National Center of Neurology and Psychiatry, Tokyo, Japan; 62 Key Laboratory of Medical Neurobiology of Zhejiang Province, Hangzhou, China; 63 Department of Psychiatry, the Second Xiangya Hospital, Central South University, Changsha, China; 64 National Clinical Research Center on Mental Disorders, Changsha, China; 65 Department of Psychiatry, Kyoto University Graduate School of Medicine, Kyoto, Japan; 66 Department of Psychiatry, Nagoya University Graduate School of Medicine, Nagoya, Japan; 67 Department of Neuropsychiatry, Graduate School of Medical Sciences, Kyushu University, Fukuoka, Japan; 68 Department of Neuropsychiatry, Faculty of Life Sciences, Kumamoto University, Kumamoto, Japan; 69 Department of Psychiatry, Nara Medical University School of Medicine, Nara, Japan; 70 Department of Neuropsychiatry and Clinical Ethics, Graduate School of Medical Science, University of Yamanashi, Yamanashi, Japan; 71 Department of Psychiatry, Division of Clinical Medicine, Faculty of Medicine, University of Tsukuba, Tsukuba, Japan; 72 Department of Neuropsychiatry, University of Toyama Graduate School of Medicine and Pharmaceutical Sciences, Toyama, Japan; 73 Department of Physiology, Keio University School of Medicine, Tokyo, Japan; 74 Department of Neuropsychiatry, Keio University School of Medicine, Tokyo, Japan; 75 Department of Neuropsychiatry, Graduate School of Medicine, Keio University School of Medicine, Tokyo, Japan

**Keywords:** Genetic association study, Schizophrenia

## Abstract

Studies on schizophrenia-associated rare copy number variants (CNVs) have predominantly focused on people of European (EUR) ancestry. Here we present a rare CNV study of schizophrenia in East Asian (EAS) populations, comprising 20,903 cases and 23,258 controls. We observed a significantly elevated genome-wide rare CNV burden in EAS cases compared with controls. Cross-population comparisons showed largely consistent rare CNV effects on schizophrenia risk. In the EAS sample, we identified nine genome-wide-significant schizophrenia-associated rare CNV loci. Meta-analysis with EUR data yielded 14 significant loci, including 8 that reached genome-wide significance for the first time. Genes within these 14 loci were significantly less tolerant to loss-of-function variants than genes in other CNV loci. The new rare CNVs associated with schizophrenia in EAS populations showed higher carrier frequencies in EAS than in EUR populations (0.38% versus 0.0017%). Overall, this study underscores the importance of increasing population diversity to fully capture the genetic underpinnings of schizophrenia.

## Abstract (web summary)

Genomic analyses in populations of East Asian ancestry identify rare copy number variants associated with schizophrenia. Meta-analyses with populations of European ancestry identify additional risk loci enriched in genes less tolerant to loss-of-function variants.

## Main

Copy number variants (CNVs) are DNA segments that are duplicated or deleted, resulting in variation in the number of copies of a genomic region in an individual’s genome^1^. CNVs typically range in length from 1 kb to 5 Mb and often span multiple genes^2^. They represent a major form of genomic variation and have been linked to gene expression^3^, phenotypic variation^4^ and disease susceptibility^5^. For example, approximately 25–30% of individuals carrying the 22q11.2 deletion develop schizophrenia, which is a substantially higher frequency than the prevalence of 1% in the general population^6,7^.

Large-scale genetic studies have established an important role of rare CNVs in schizophrenia etiology^8–11^. On average, rare CNVs are observed at a higher rate in individuals with schizophrenia than in controls, and previously reported CNV associations have substantially larger effect sizes (odds ratios (ORs) ranging from 4 to over 100) than common variants (typical OR of <1.3)^12–14^. For example, a landmark study conducted by the Psychiatric Genomics Consortium (PGC) involving 41,321 participants identified eight schizophrenia-associated rare CNVs, with reported ORs ranging from 3.8 to 67.7 (ref. ^14^).

Despite these advances, large-scale studies of schizophrenia-associated CNVs have predominantly focused on individuals of European (EUR) ancestry^14^. The lack of ancestral diversity limits the discovery of genetic variants, including CNVs, that may be rare in EUR populations but more frequent in other populations. For example, a genome-wide association study (GWAS) in East Asian (EAS) populations identified a schizophrenia-associated locus hosting the gene *CACNA2D2*, which was missed in larger EUR studies due to the low frequency of the associated variants in EUR populations (<1%) and their 60-fold-higher frequency in EAS populations^15^.

**Fig. 1:**
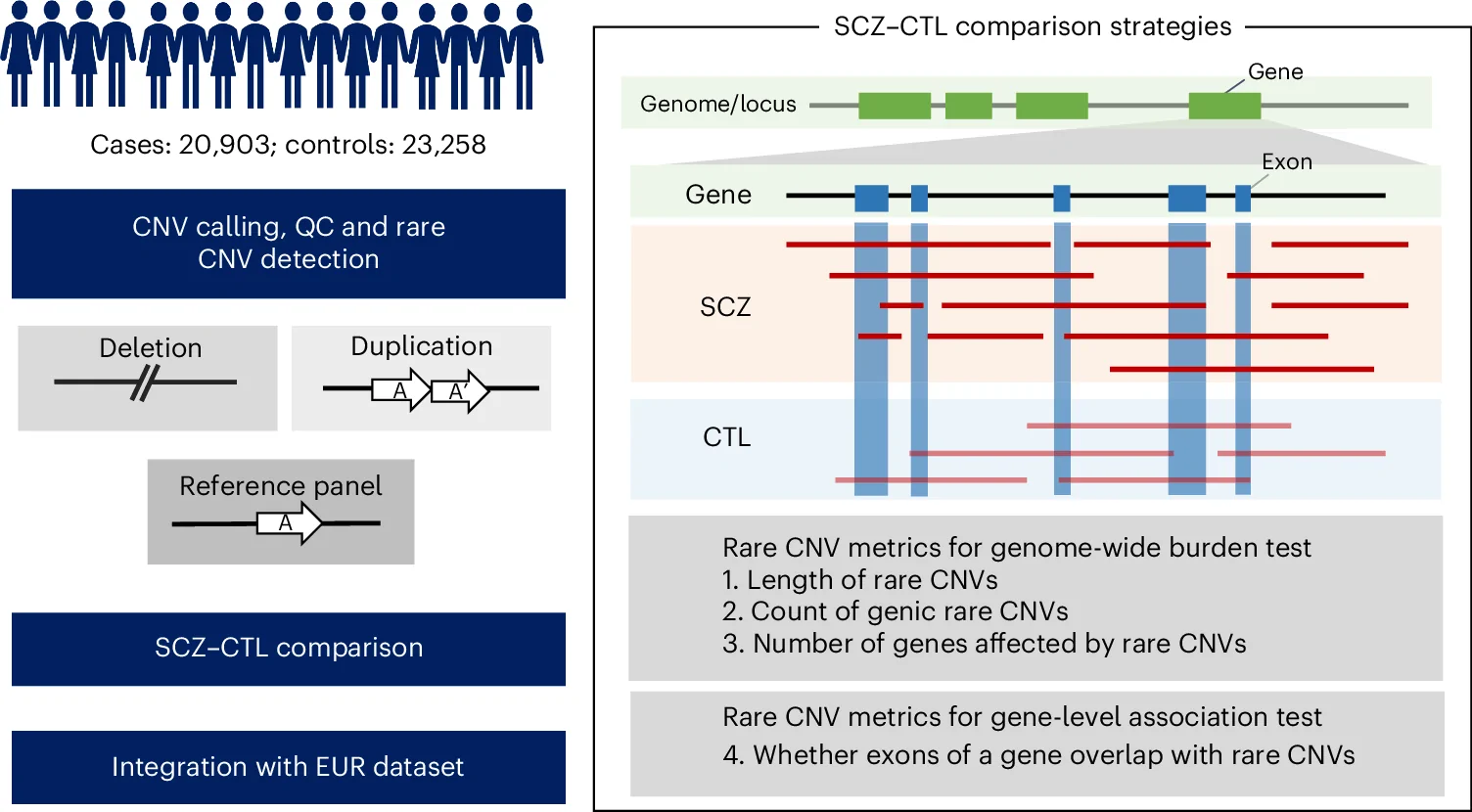
Study design. In the right panel, rare CNVs are depicted in red, genes are depicted in green and exons are depicted in blue. Several rare CNV burden metrics were tested for association with schizophrenia. CTL, control; SCZ, schizophrenia.

Here we present a large-scale study of rare CNVs in schizophrenia in EAS populations, including 20,903 cases and 23,258 controls, tripling the case sample size of previous EAS CNV studies of schizophrenia^16^. We developed a robust pipeline for rare CNV calling and quality control (QC), enabling rigorous analyses of rare CNVs across 15 EAS samples. Using this high-confidence callset, we examined genome-wide rare CNV burden, identified schizophrenia-associated rare CNV loci and compared their effect sizes across ancestries (Fig. 1).

## Results

### Study participants

We analyzed data from 51,667 individuals across 15 EAS schizophrenia samples (Supplementary Table 1 and Supplementary Note). Following harmonization and rigorous QC of rare CNVs across the samples and genotyping platforms (Methods), a total of 44,161 participants, including 20,903 cases and 23,258 controls, were retained for downstream analyses (Table 1). For cross-population comparison, we also incorporated a rare CNV callset from a previously published PGC CNV study^16^, which included 34,257 individuals of EUR ancestry (17,506 cases and 16,751 controls).

**Table 1:** Summary of samples in this study

Population	Region	Platform	Sample	Cases	Controls	Sample size
**EAS**	Mainland China	ASA	xajd	435	629	1,064
	Mainland China	ASA	sxyh1	4,167	2,106	6,273
	Mainland China	ASA	xju2	790	2,258	3,048
	Mainland China	GSAA	biox4	284	452	736
	Mainland China	Om25	xju1	1,629	886	2,515
	Mainland China	Om25	push2	2,520	2,427	4,947
	Mainland China	PSYC	six2	2,201	1,940	4,141
	Taiwan	GSAA	taiml	565	513	1,078
	Taiwan	GSAA	tai2t	1,157	1,777	2,934
	Taiwan	PSYC	tai3	1,584	3,046	4,630
	Japan	GSAA	tmim1	2,721	3,100	5,821
	Japan	GSAA	tmim3	500	1,660	2,160
	Singapore	Il1M	imh1	769	854	1,623
	Singapore	Om25	imh2	707	772	1,479
	Indonesia	PSYC	uwa1	874	838	1,712
EAS total			15	20,903	23,258	44,161
EUR total				17,506	16,751	34,257

Numbers reflect post-QC sample sizes. Platforms: ASA, Infinium Asian Screening Array; GSAA, Infinium Global Screening Array; Il1M, Human1M-Duo; PSYC, Infinium PsychArray; Om25, Infinium OmniZhongHua. For EUR samples, the following platforms were used: Affymetrix 500, Illumina 300 K, Illumina 610 K, Illumina 660 W, Affymetrix 5.0, Affymetrix 6.0, OmniExpress and OmniExpress plus Exome.

### Rare CNV calling and QC

**Extended Data Fig. 1:**
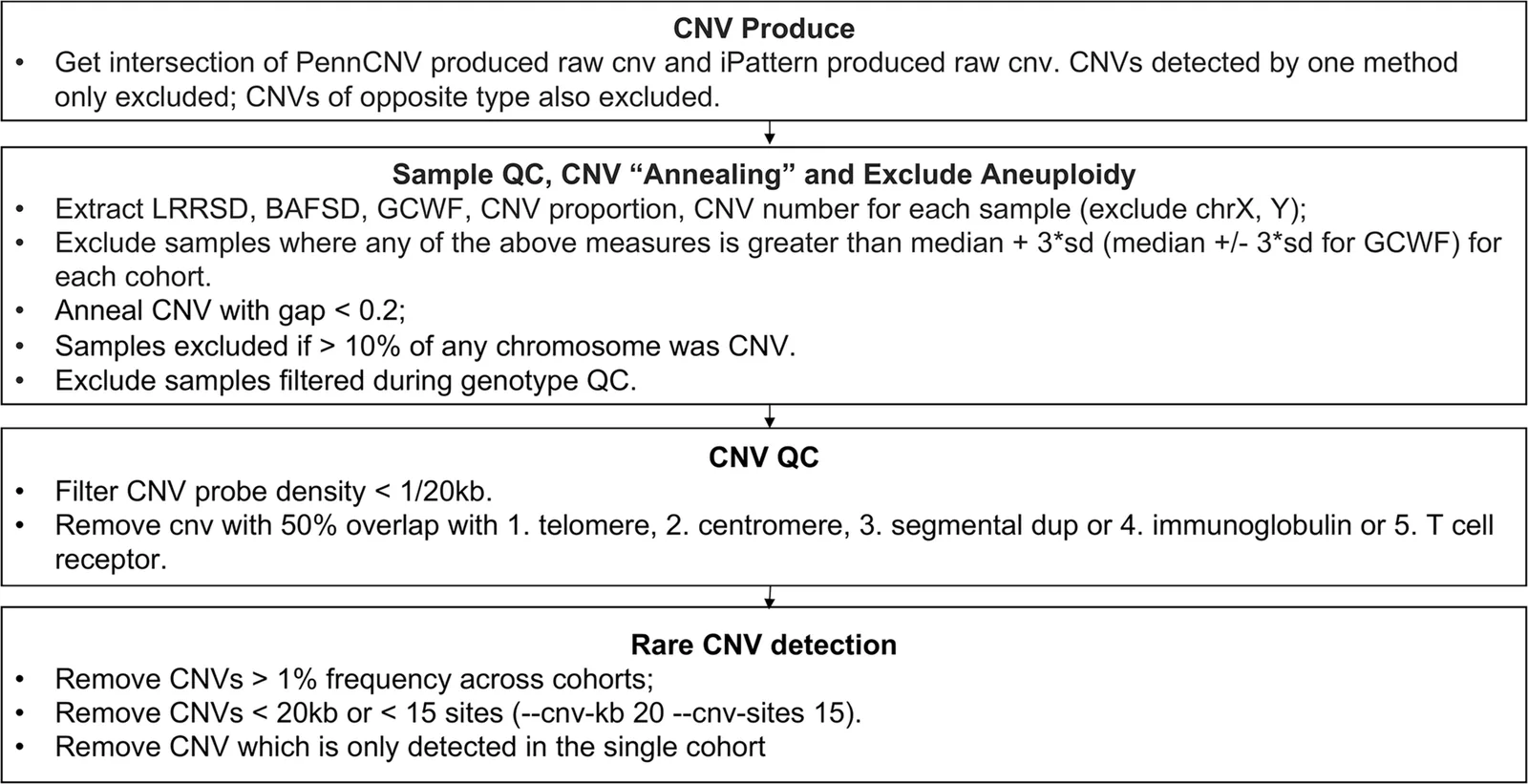
Rare copy number variants (rare CNVs) calling pipeline. Schematic overview of the workflow used to identify high-quality rare CNVs, including consensus calling with PennCNV and iPattern, sample-level quality control, CNV annealing, exclusion of aneuploid samples, CNV-level filtering and rare CNV detection.

**Extended Data Fig. 2:**
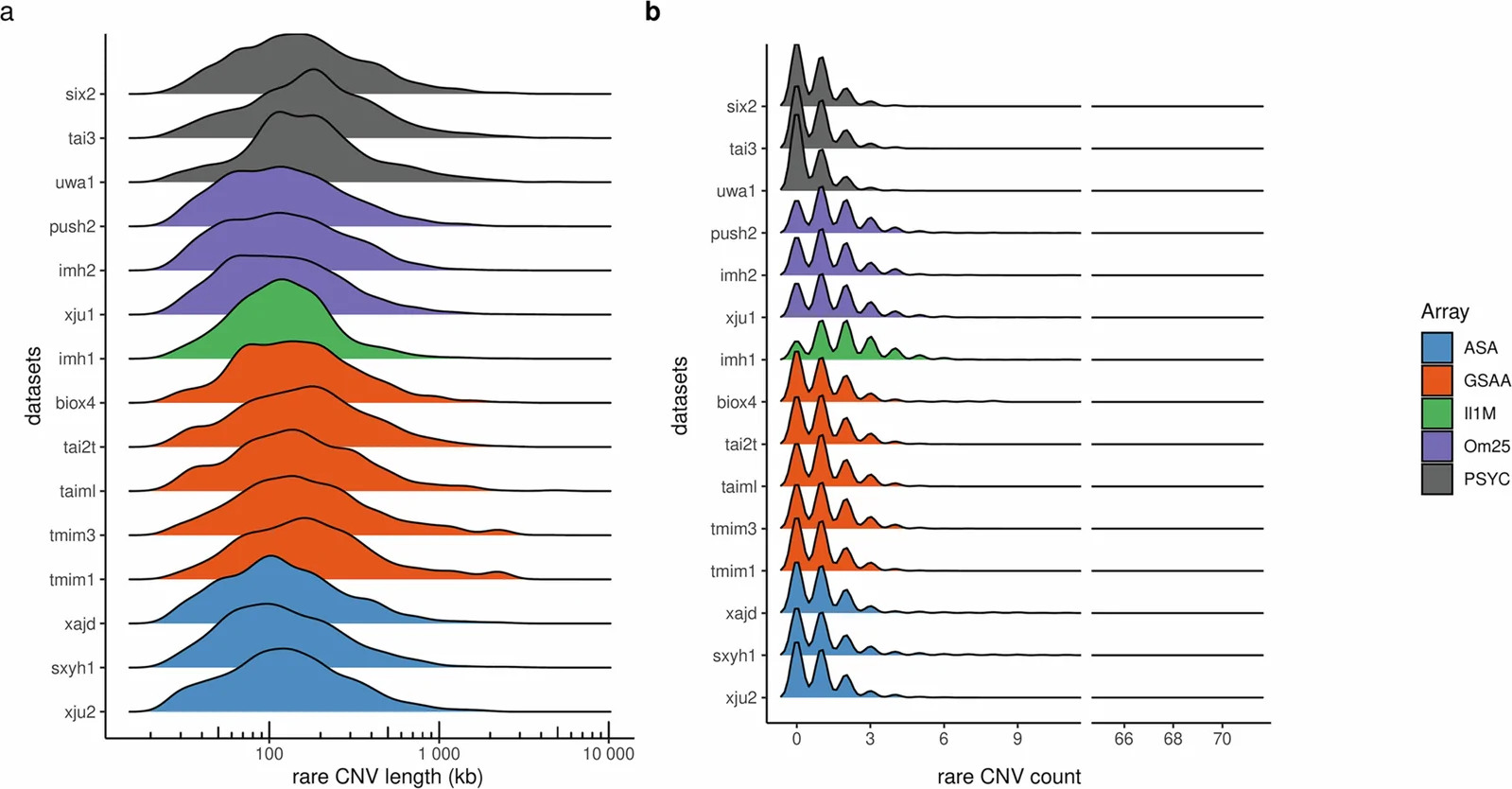
Distribution of rare CNV for each EAS sample. Ridges plot showing the distribution of rare CNV callset information, including **a**, average rare CNV length (kb) and **b**, rare CNV count per carrier across samples. The color represents the Illumina array types. ASA: Infinium Asian Screening Array; GSAA: Infinium Global Screening Array; Il1M: Human1M-Duo; PSYC, Infinium PsychArray; Om25, Infinium OmniZhongHua.

To accurately identify and harmonize rare CNVs across the EAS samples, we implemented a unified and robust pipeline for rare CNV calling and QC, built on the PennCNV^17^ and iPattern^18^ packages (Supplementary Note, Supplementary Fig. 1 and Extended Data Fig. 1). High-quality rare CNVs were defined as segments ≥20 kb in length, supported by ≥15 probes and present at a frequency of <1% across all participants (Supplementary Table 2). The details of parameter selection, including the justification for using 15 probes as the threshold as opposed to the 10 probes used in the published PGC study^14^, are provided in the Supplementary Note. Following QC, the average rare CNV length and number were consistent across samples (Extended Data Fig. 2). A final callset of 58,536 high-quality rare CNVs was available for subsequent analyses.

**Extended Data Fig. 3:**
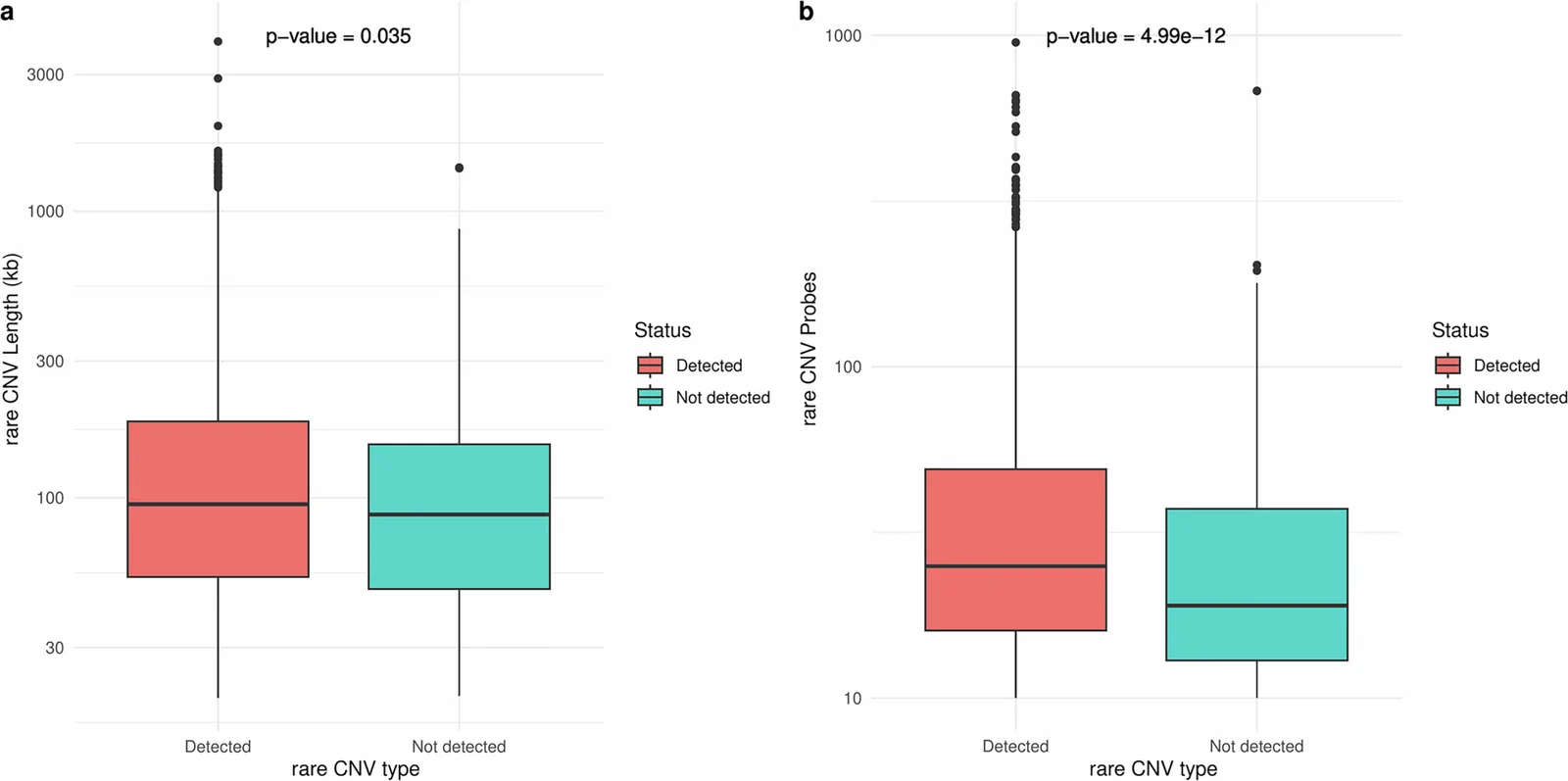
Rare CNV calling pipeline consistency evaluation. **a**, Distribution of rare CNV length (bp) for detected versus not detected rare CNVs. **b**, Distribution of probe counts for detected versus not detected rare CNVs. Detected rare CNVs were defined as rare CNVs identified in the EUR calling pipeline that were also identified in the EAS calling pipeline with >75% reciprocal overlap. Not detected rare CNVs were defined as rare CNVs identified in the EUR calling pipeline that were not replicated in the EAS calling pipeline. Each data point represents one rare CNV event (unit of study). An individual could contribute more than one CNV event. The underlying dataset included 4,954 subjects. The numbers of rare CNV events were n = 8,183 (detected) and n = 2,467 (not detected) in each panel. Box plots were generated using ggplot2 default settings: center line, median; box limits, 25th and 75th percentiles (Q1 and Q3); whiskers, the most extreme values within 1.5 interquartile range from the box; points beyond whiskers, outliers. P values were calculated using two-sided Student’s t-test and are unadjusted.

Rare CNV calling and QC for participants of EUR ancestry were performed as part of a previously published PGC study^17^, resulting in a post-QC callset of 60,562 high-quality rare CNVs. To assess consistency, we applied our CNV calling pipeline to two PGC EUR samples (3,309 participants) and compared the results with the published callset^18^ (Methods). As the published EUR callset used a 10-probe threshold as opposed to the 15-probe threshold in this study, to ensure robustness, we performed our CNV calling using both the 10-probe (same as the published EUR study) and the 15-probe (used in subsequent analyses in this study) thresholds. Both thresholds yielded highly consistent results, confirming that the probe threshold is unlikely to influence the consistency between pipelines (Supplementary Table 3). For instance, using either threshold, our pipeline successfully recapitulated approximately 84% (8,183) of the rare CNVs reported in the published study (Methods and Supplementary Table 3). The difference is likely driven by the fact that QC for the published EUR callset was performed using frequencies calculated on all individuals rather than from the subset used for the consistency check. The remaining ~16% of CNVs not detected by our pipeline tended to be smaller and supported by fewer probes compared with those retained (*P*_length_ = 0.035, *P*_probes_ = 5 × 10^−^^12^, two-sided *t* test; Extended Data Fig. 3).

**Extended Data Fig. 4:**
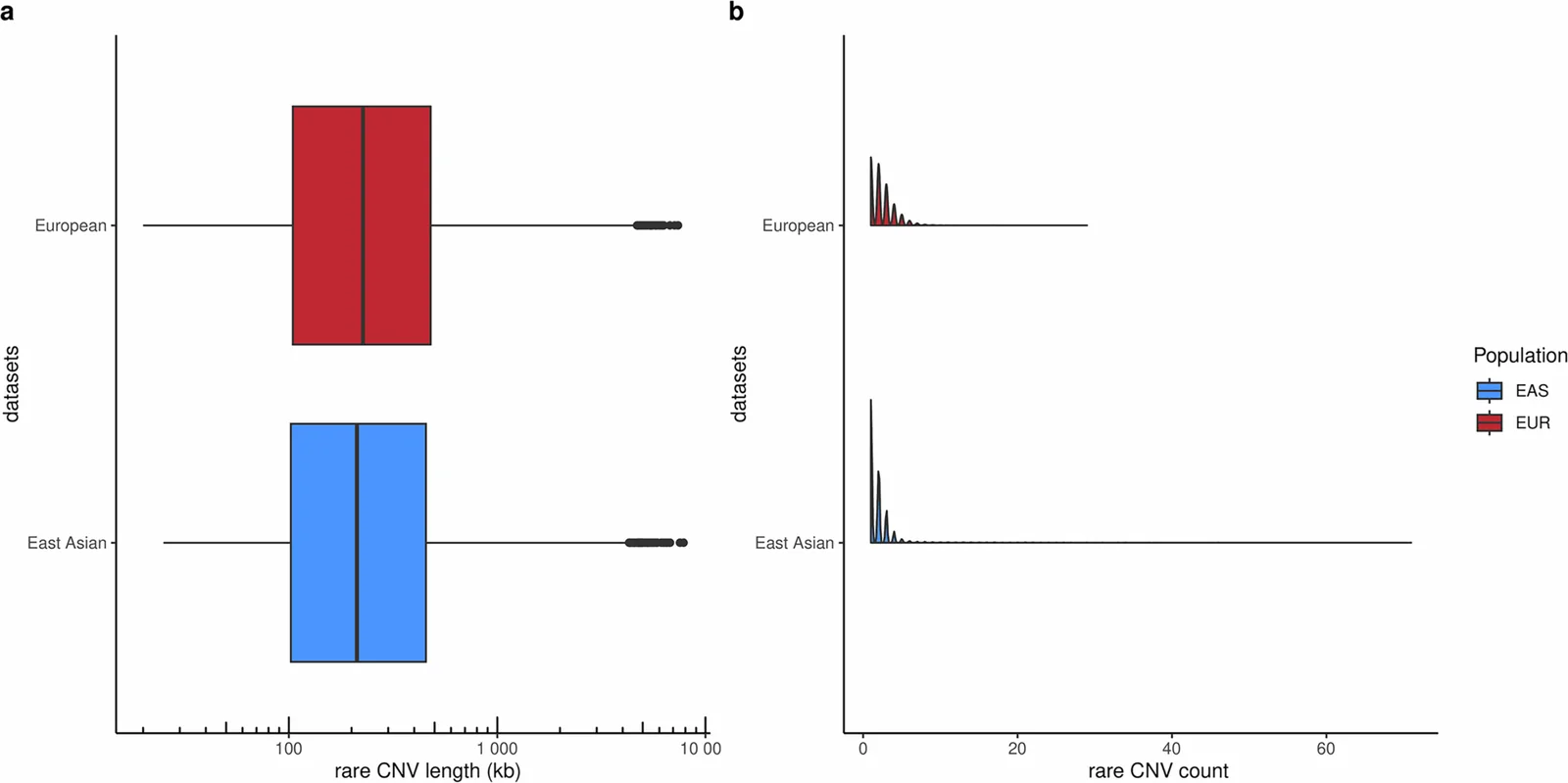
Rare CNV comparison between EAS and EUR populations. **a**, Average rare CNV length across all subjects in EAS and EUR populations (EAS, n = 44,161; EUR, n = 34,257), including cases and controls. The unit of study is the study subject. Each dot represents the average rare CNV length of one subject. Vertical lines denote the median; boxes extend from the 25th to the 75th percentile of each group’s distribution of values; whiskers denote adjacent values (that is, the most extreme values within 1.5 interquartile range of the 25th and 75th percentile of each group); dots denote observations outside the range of adjacent values. **b**, rare CNV count per carrier across all subjects (EAS, n = 44,161; EUR, n = 34,257), including both cases and controls. The unit of study is the individual subject. Violin plots show the distribution of rare CNV counts across subjects in each population; the width of each violin at a given value reflects the relative density of observations at that rare CNV count.

In addition, we compared the median rare CNV length and number per carrier between the EAS and EUR callsets. Both measures were lower in the EAS samples (median CNV length, 92.50 kb versus 227.01 kb; mean CNV number per carrier, 1.32 versus 2.41; Extended Data Fig. 4), probably reflecting reduced CNV detection sensitivity in the EAS samples due to genotyping arrays being predominantly designed and optimized for EUR populations. This conclusion remains valid despite the use of slightly different probe count thresholds for CNV calling in the EUR and EAS datasets (Supplementary Note).

### Genome-wide rare CNV burden in the EAS samples

We assessed the association between genome-wide rare CNV burden and schizophrenia case–control status in EAS samples using a logistic mixed-effects model. We included sex, genotyping platform and the top five principal components (PCs) derived from genome-wide genotypes as fixed-effect covariates and a random effect to account for genetic relatedness (Methods). We examined three genome-wide rare CNV burden metrics: (1) the total length of rare CNVs overlapping genes; (2) the number of genic rare CNVs; and (3) the number of genes affected by rare CNVs. The OR was defined per unit increase in each metric: (1) per 100 kb of rare CNVs; (2) per additional genic rare CNV; and (3) per gene affected by rare CNVs.

**Fig. 2:**
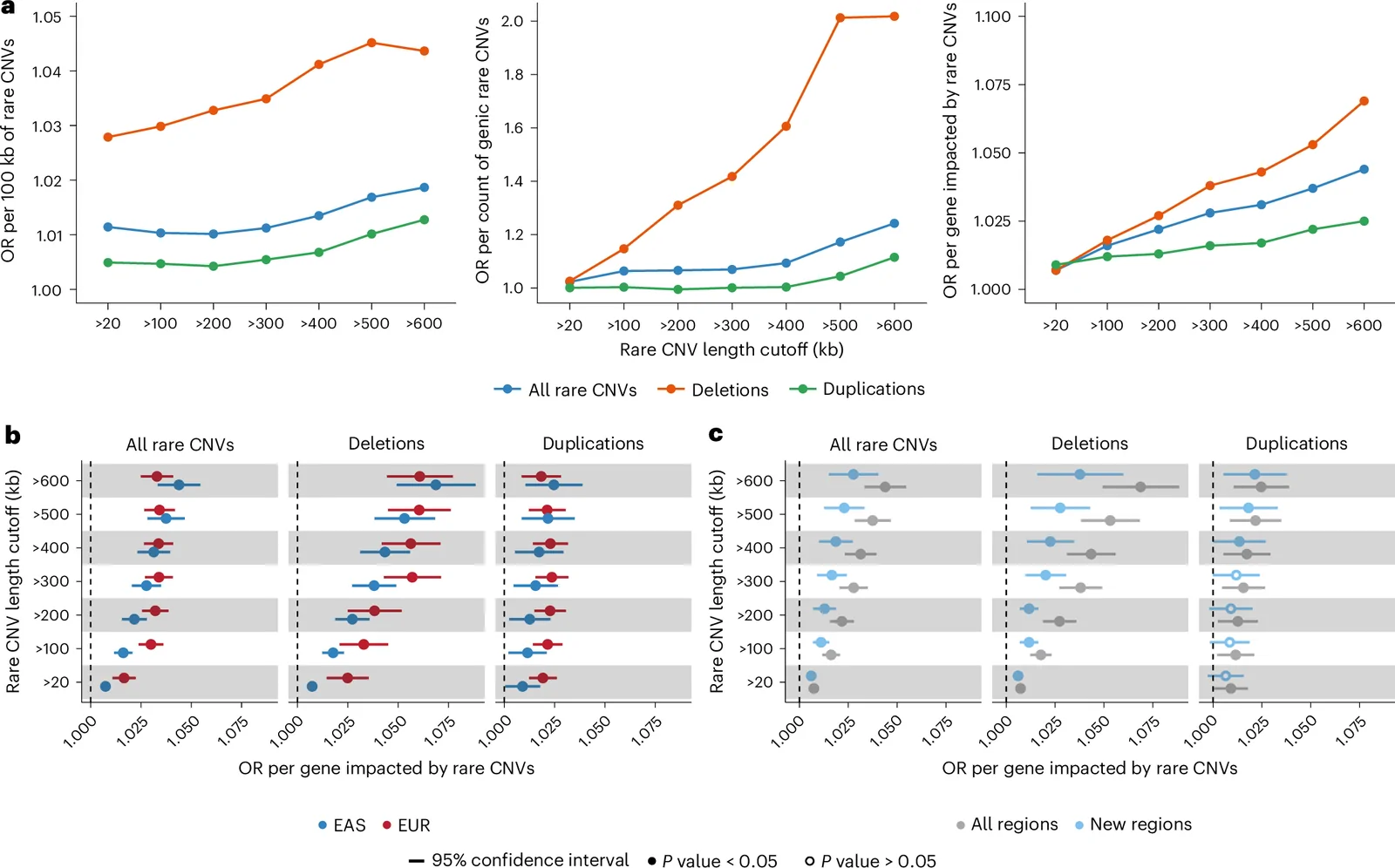
Genome-wide rare CNV burden in schizophrenia across ancestry groups. **a**, The impact of rare CNV length, the number of genic rare CNVs and the number of genes affected by rare CNVs on schizophrenia risk. Effect estimates were obtained using the logistic mixed-effects model. **b**, The effect of genome-wide rare CNV burden on schizophrenia risk in EAS and EUR populations. Horizontal error bars indicate 95% CIs. **c**, The effect of genome-wide rare CNV burden on schizophrenia risk before (‘all regions’) and after (‘new regions’) excluding previously reported schizophrenia-associated CNV loci (defined in the Methods). Horizontal error bars indicate 95% CIs. *P* values were derived from two-sided tests from the logistic mixed-effects model. Sample sizes were *n* = 44,161 for EAS populations and *n* = 34,257 for EUR populations.

**Extended Data Fig. 5:**
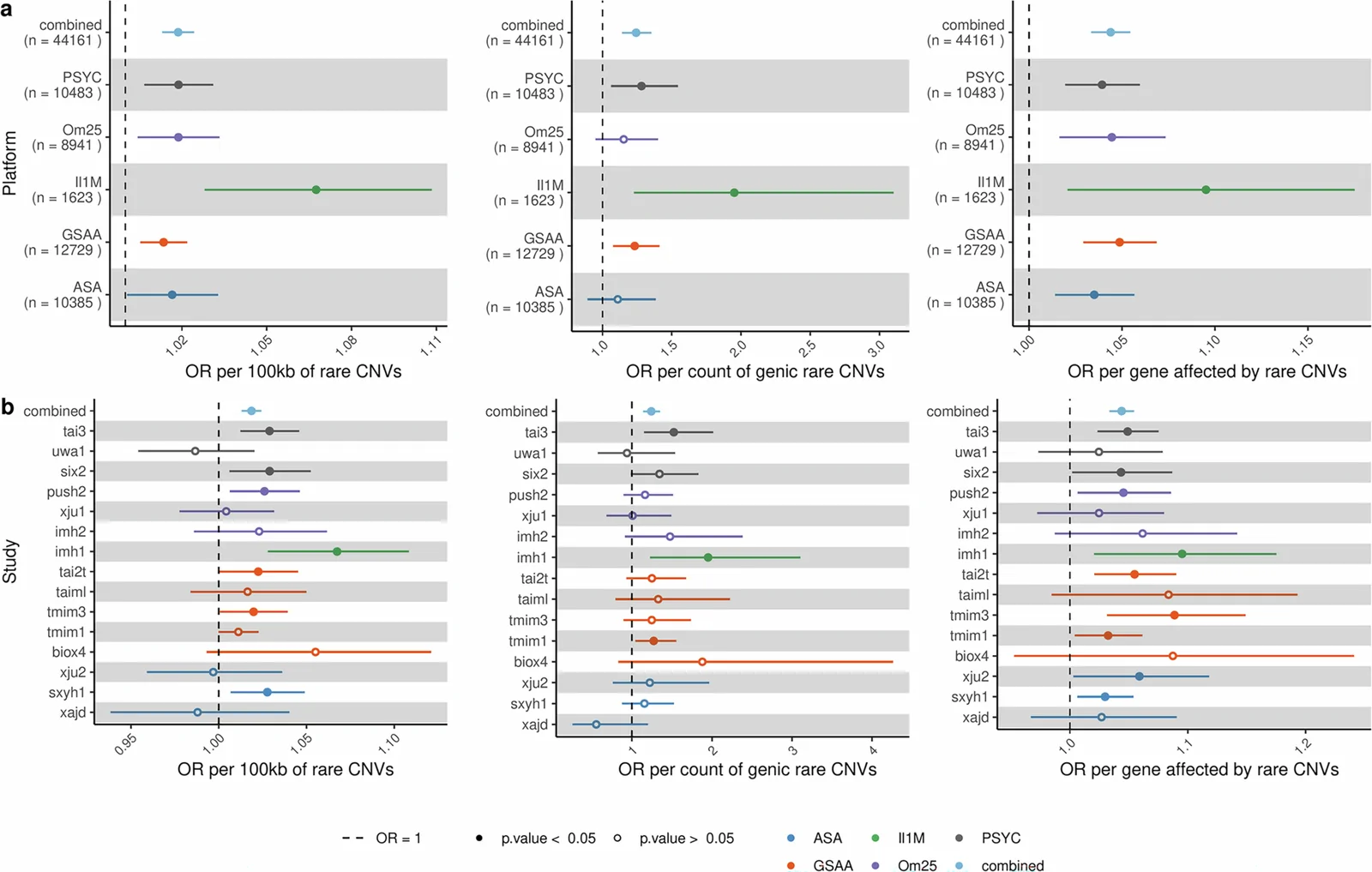
Genome-wide rare CNV burden partitioned by genotyping platforms and samples. **a**, Effect of genome-wide rare CNV burden on schizophrenia risk in each genotyping platform and the combined analysis. ASA: Infinium Asian Screening Array; GSAA: Infinium Global Screening Array; Il1M: Human1M-Duo; PSYC, Infinium PsychArray; Om25, Infinium OmniZhongHua **b**, Effect of genome-wide rare CNV burden on schizophrenia risk in each sample and the combined analysis. Horizontal error bars indicate 95% confidence intervals. The dashed vertical line indicates OR = 1. P-values were unadjusted and estimated from the two-sided test using logistic mixed-effects regression. Exact P values for all plotted tests are provided in the Supplementary Table 5.

All three metrics showed significant enrichment in schizophrenia cases compared with controls (Fig. 2a and Extended Data Table 1). For example, patients with schizophrenia had significantly more genes affected by rare CNVs >20 kb in length than controls (OR = 1.007; 95% confidence interval (CI) = 1.005–1.009; *P* = 6.82 × 10^−^^12^; Fig. 2a). Effect sizes increased with CNV length, with ORs exceeding 1.03 for rare CNVs >500 kb in length. Stratification by CNV type revealed consistently stronger associations for deletions than for duplications (Fig. 2a and Extended Data Table 2). For example, the number of genes affected by deletions >600 kb in length was associated with an OR of 1.069 (95% CI = 1.049–1.088; *P* = 4.10 × 10^−^^11^), compared with an OR of 1.025 for duplications (95% CI = 1.011–1.039; *P* = 5.46 × 10^−^^4^). In general, the OR estimates were consistent across genotyping platforms, suggesting the robustness of the findings (Supplementary Note, Supplementary Tables 4 and 5 and Extended Data Fig. 5). We also confirmed that log R ratio standard deviation (LRR-SD)—a widely used QC metric for genotyping arrays—had minimal impact on our conclusion (Supplementary Note and Supplementary Fig. 2).

**Extended Data Fig. 6:**
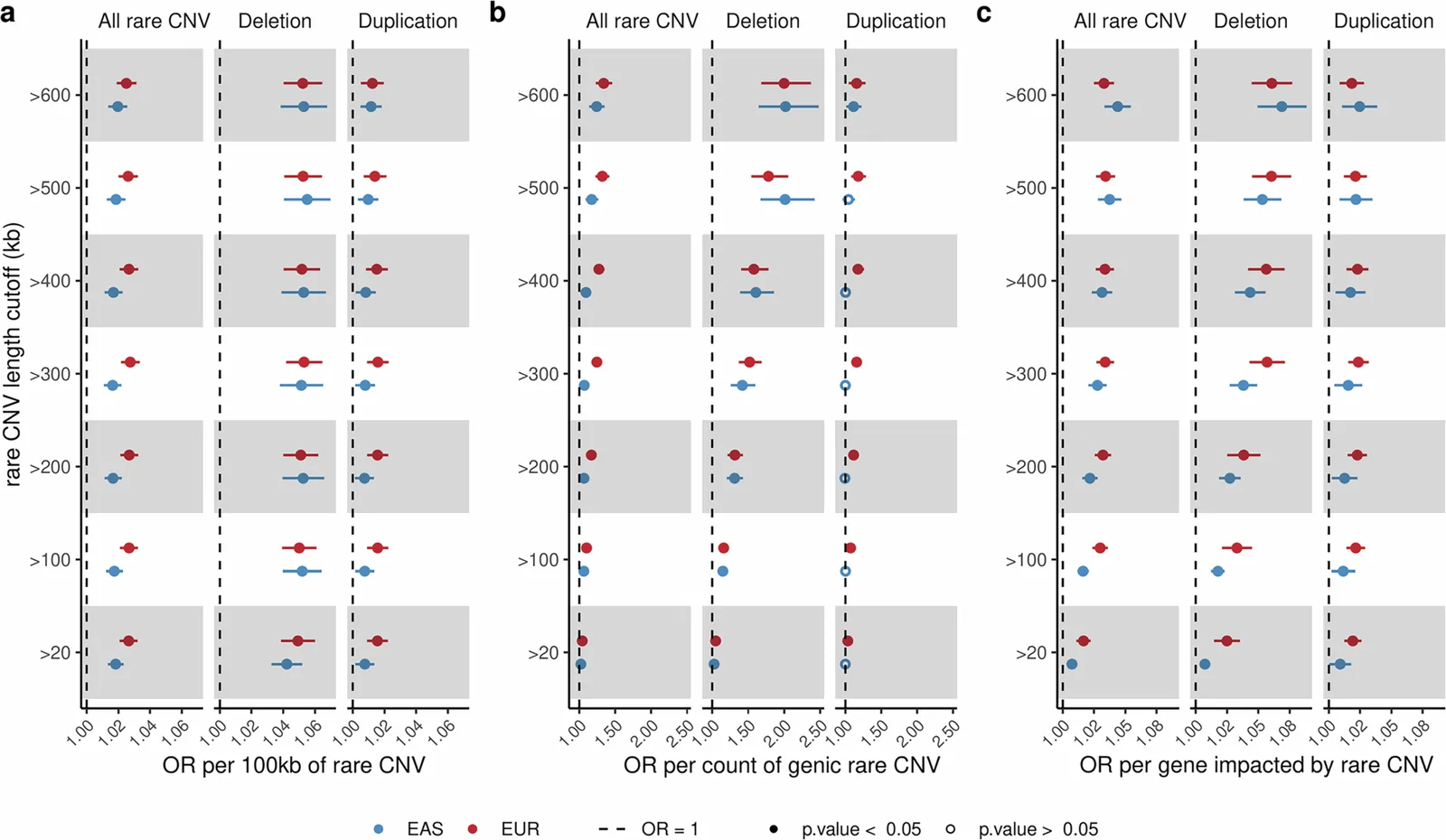
Genome-wide rare CNV burden comparison between EAS and EUR. **a**, Odds ratio (OR) per 100 kb of rare CNVs. **b**, OR per count of genic rare CNVs. **c**, OR per gene impacted by rare CNVs. Results are shown for all rare CNVs, deletions, and duplications. Horizontal error bars indicate 95% confidence intervals. The vertical dashed line indicates OR = 1. Sample sizes: EAS, n = 44,161 and EUR, n = 34,257 subjects. P-values were unadjusted and assessed using two-sided logistic mixed-effects regression; exact P values are provided in Supplementary Table 6.

The effect of genome-wide rare CNV burden on schizophrenia was similar between the EAS and EUR populations. For example, the number of genes affected by rare CNVs >600 kb in length showed comparable associations in EAS (OR = 1.044; 95% CI = 1.033–1.054; *P* = 5.18 × 10^−3^) and EUR (OR = 1.033; 95% CI = 1.024–1.041; *P* = 3.97 × 10^−3^) populations (Fig. 2b). Similar patterns were observed across CNV types and burden metrics (Supplementary Table 6 and Extended Data Fig. 6).

To determine whether the associations of genome-wide burden were driven by known schizophrenia-associated rare CNVs, we repeated the analysis after excluding all previously reported schizophrenia-associated rare CNV loci (Methods and Supplementary Table 7). The enrichment of genome-wide rare CNV burden in schizophrenia cases remained significant. For example, the OR per gene affected by deletions >20 kb in length decreased only slightly from 1.007 to 1.006 (95% CI = 1.004–1.008; *P* = 3.83 × 10^−9^; Fig. 2c, Extended Data Table 3 and Supplementary Table 8), suggesting that additional schizophrenia-associated rare CNVs remain to be discovered in EAS populations.

To investigate the interplay between rare CNV burden and the polygenic risk score (PRS) for schizophrenia, and their collective contribution to disease risk^19,20^, we estimated SNP weights using PRS-CS^21^ based on summary statistics from the EAS schizophrenia GWAS^15^ and calculated individual-level PRSs for the 30,459 participants who were not part of the GWAS (Methods and Supplementary Table 9a). Larger PRS was associated with an increased risk of schizophrenia. Consistent with previous reports^22^, individuals with schizophrenia carrying large deletions (>500 kb) had significantly lower PRS than noncarriers (mean PRS: 0.39 versus 0.49; *P* = 0.01, two-sided Wilcoxon rank-sum test; Supplementary Table 9b), whereas no significant difference in PRS was observed between control carriers and noncarriers. Rare CNV burden was significantly associated with schizophrenia risk, although its variance explained (*R*^2^) on the liability scale was substantially lower than that for PRS (Supplementary Table 9c), which is expected due to the low frequency of rare CNVs in the population. Nevertheless, rare CNV burden contributed additional schizophrenia risk beyond PRS. For example, when measured as the number of genes affected by rare CNVs, the burden remained significantly associated with schizophrenia after adjusting for PRS (*P* = 2.09 × 10^−4^; Supplementary Table 9d). Lastly, we observed a small but statistically significant interaction between PRS and rare CNV burden (Supplementary Table 9e and Supplementary Fig. 3). For instance, an increase of 1 s.d. in the rare CNV burden, measured as the number of genes affected, increased schizophrenia risk while slightly attenuating the effect of PRS (*P* = 8.1 × 10^−9^).

### Gene-level rare CNV associations in EAS populations

To identify rare CNV loci contributing to schizophrenia risk in EAS populations, we tested the association between each ‘rare CNV gene’ and schizophrenia case–control status using logistic mixed-effects models implemented in REGENIE^23^. A gene was defined as a ‘rare CNV gene’ if one or more of its exons overlapped with rare CNVs (Methods). A total of 2,183 rare CNV genes were analyzed, and a genome-wide significance threshold was established via permutations (*P*_cutoff_ = 6.88 × 10^−5^; Methods).

We identified 87 rare CNV genes that were significantly associated with schizophrenia (Supplementary Table 10). Using curated synaptic ontologies from the SynGO Consortium^24^, we assessed gene sets implicated by these rare CNV genes (Methods). We found significant enrichment (false discovery rate-adjusted *P*<0.05) for seven gene sets (Supplementary Table 11). Of these, two gene sets were also significant in the analysis using rare CNV genes from the published EUR study, while the remaining five gene sets were significant only in EAS populations. For example, the gene set for transmitter-gated ion channel activity involved in the regulation of postsynaptic membrane potential (GO:1904315) was significant in EAS (*P* = 0.0013) but not in EUR populations.

**Fig. 3:**
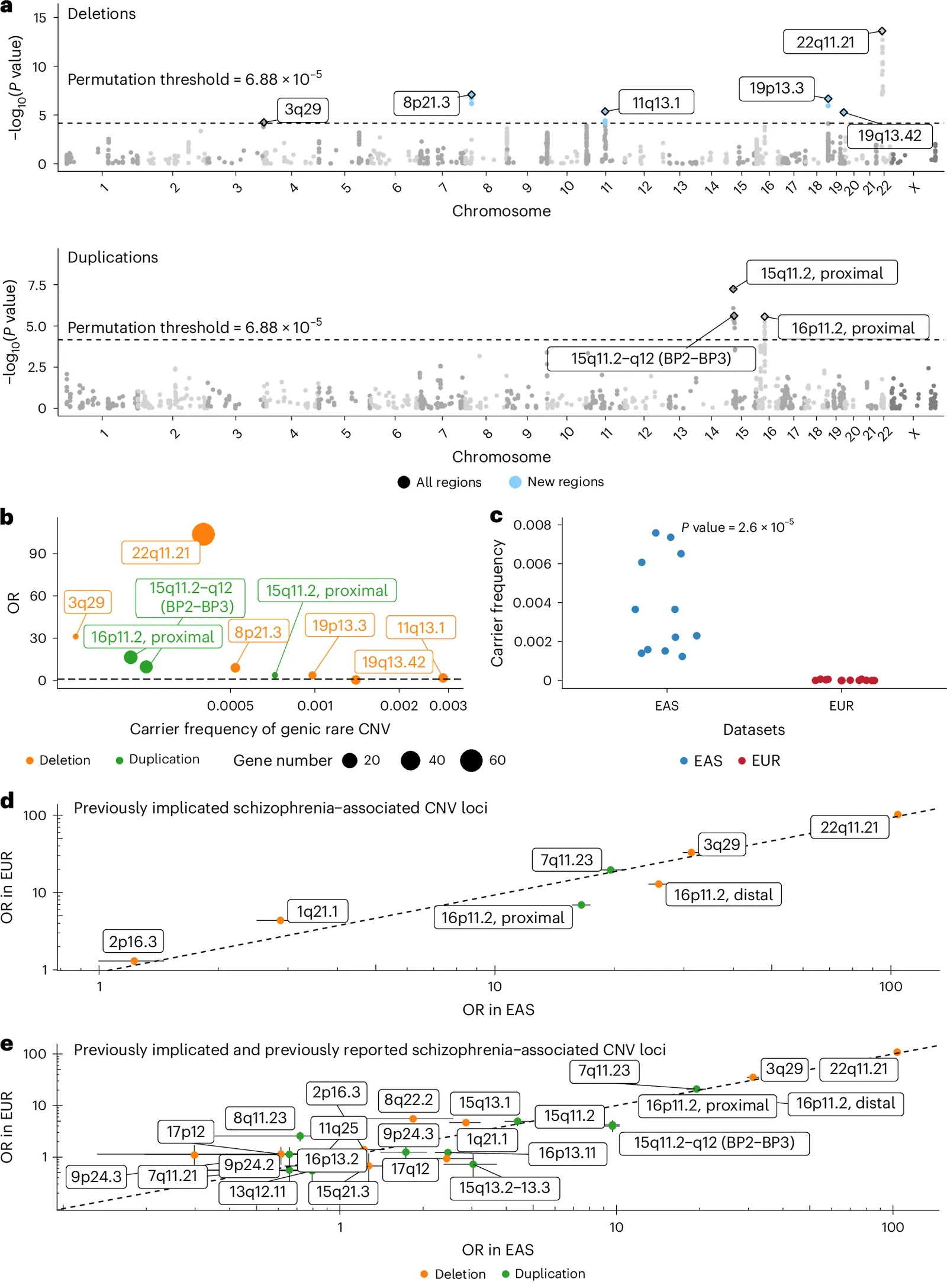
Gene-level rare CNV associations in EAS populations. **a**, Manhattan plots of gene-level associations for rare CNV deletions and duplications in EAS populations. Rare CNV loci reaching genome-wide significance are labeled. Genes within new loci are highlighted in blue. *P* values are unadjusted and were estimated using two-sided logistic mixed-effects regression as implemented in REGENIE (Methods). Deletions and duplications were analyzed separately. The empirical genome-wide significance threshold for the EAS analysis was *P*<6.88 × 10^−5^ based on the permutation test (Methods). Only genes affected by rare CNVs in at least eight participants were tested. **b**, ORs and carrier frequencies of the nine rare CNV loci associated with schizophrenia at genome-wide significance in EAS populations. The dashed line indicates an OR of 1. **c**, Carrier frequencies for the 12 genes reaching genome-wide significance within the four new rare CNV loci identified in EAS populations. CNV loci were defined by aggregating genes affected by rare CNVs with >50% overlap across participants and were annotated using UCSC Genome Browser cytoband annotations. *P* values are unadjusted and were calculated using a two-sided Wilcoxon signed-rank test. **d**, ORs of previously implicated schizophrenia-associated CNV loci. **e**, ORs of previously implicated and previously reported schizophrenia-associated CNV loci. For **d** and **e**, ‘previously implicated’ indicates that the locus was genome-wide significant in the PGC EUR CNV study, and ‘previously reported’ indicates that the locus was described in a publication but did not reach genome-wide significance in the PGC EUR CNV study. ORs were derived from rare CNV association analyses (Methods). Sample sizes for the EAS and EUR analyses were 44,161 and 34,257, respectively. Cochran’s *Q* test (two sided) was used to test heterogeneity. None of these loci showed heterogeneity across populations after Bonferroni correction (*P*>0.05). Results are plotted as mean values ± 95% CIs (error bars).

Given that large rare CNVs often span multiple genes, we aggregated rare CNV genes into CNV loci if their corresponding CNVs overlapped by more than 50% across participants to reflect uncertainty in implicating specific genes. The 87 significant rare CNV genes were grouped into nine independent schizophrenia-associated loci (Table 2), including six deletions (3q29, 8p21.3, 11q13.1, 19p13.3, 19q13.42 and 22q11.21) and three duplications (15q11.2, proximal; 15q11.2–q12, BP2–BP3; and 16p11.2, proximal) (Fig. 3a,b). As the 15q11–q13 region is a known genomic rearrangement hotspot, we analyzed this region using the five previously reported breakpoints (BP1–BP5)^25^. We found significant associations for CNVs located upstream of BP1 (15q11.2, proximal) and between BP2 and BP3 (15q11.2–q12, BP2–BP3; Supplementary Fig. 6). We did not find any significant association with schizophrenia for CNVs between BP1 and BP2, despite previous reports.

**Table 2:** Rare CNV loci associated with schizophrenia in EAS populations reaching genome-wide significance

Locus	Position (hg19)	CNV type	OR	*P* value	Carrier freq. (all)	Carrier freq. (genic)	Cases/controls	Status	Top rare CNV gene	Sig. genes	Refs.
22q11.21	18874965– 22033435	Deletion	103.75	2.38 × 10^−14^	0.00093	0.00042	41/0	Previously implicated	*DGCR2*, *DGCR14*, *CLTCL1*, *DGCR8*, *GSC2*, *TANGO2*, *TBX1*, *C22orf39*, *HIRA*, *UFD1L*, *CLDN5*, *GNB1L*, *TXNRD2*, *SLC25A1*, *GP1BB*, *SEPIN5*, *C22orf29*, *ARVCF*, *CDC45*, *MRPL40*, *COMT*, *TSSK2*	46	^10,14,27–30^
15q11.2, proximal	22368478– 22383507	Duplication	4.04	5.82 × 10^−8^	0.00208	0.00076	69/23	Previously reported	*OR4M2*	2	^31^
8p21.3	21884544– 22038952	Deletion	9.09	8.14 × 10^−8^	0.00159	0.00055	59/11	New loci	*DMTN*	4	–
19p13.3	572293– 2748597	Deletion	3.74	2.12 × 10^−7^	0.00222	0.00103	84/14	New loci	*TMEM259*	2	–
15q11.2-q12 (BP2–BP3)	22383300– 31317385	Duplication	9.71	2.43 × 10^−6^	0.00057	0.00026	22/3	Previously reported	*ATP10A*, *GABRA5*, *GABRB3*	12	^29,30,32^
16p11.2, proximal	28825605– 30208522	Duplication	16.49	2.75 × 10^−6^	0.0005	0.00023	21/1	Previously implicated	*SPN*, *QPRT*	14	^11,14,29^
11q13.1	65294491– 65429164	Deletion	1.84	4.33 × 10^−6^	0.00652	0.00303	213/75	New loci	*MAP3K11*	4	–
19q13.42	55255021– 55351096	Deletion	0.42	5.38 × 10^−6^	0.00365	0.00148	49/112	New loci	*KIR3DL1*, *KIR2DL4*	2	–
3q29	195592967–197394948	Deletion	31.2	5.74 × 10^−5^	0.00118	0.00015	41/11	Previously implicated	*PIGZ*	1	^14,28,29,33^

‘Previously implicated’ indicates loci that reached genome-wide significance in the PGC CNV study, whereas ‘previously reported’ indicates loci previously described in the literature but not meeting genome-wide significance in the PGC CNV study (Methods and Supplementary Table 7). Carrier frequencies (all CNVs) were calculated using individuals carrying rare CNVs. Carrier frequencies (genic CNVs) were calculated using individuals carrying genic rare CNVs. The OR, *P* value and ‘top rare CNV genes’ correspond to genes with the most significant *P* value from the gene-level burden test. The start and end positions reflect the CNV breakpoints containing genes with genome-wide significance. Genomic positions are based on the hg19 reference genome.

We inferred the likely mutational mechanisms, classifying each CNV locus as arising via nonallelic homologous recombination (NAHR) or nonhomologous end joining (NHEJ) based on the breakpoints (Methods). Consistent with prior findings^14^, eight of the nine significant loci were probably mediated by NAHR, with the exception of 8p21.3.

Six of the nine schizophrenia-associated rare CNV loci identified in EAS populations reached genome-wide significance (*P*_cutoff_ = 6.88e × 10^−5^) for the first time. Four of these loci—deletions at 8p21.3, 11q13.1, 19p13.3 and 19q13.42—had not been previously reported in EUR populations (Methods). Within these four new loci, 12 rare CNV genes were significantly associated with schizophrenia in EAS populations. We found that the carrier frequencies of the rare CNVs implicating these 12 genes were significantly higher in EAS than in EUR populations (average carrier frequency, 0.38% in EAS versus 0.0017% in EUR; *P* = 2.6 × 10^−5^, two-sided Wilcoxon signed-rank test; *P* = 0.002, two-sided Wilcoxon rank-sum test; Fig. 3c, Table 3 and Supplementary Table 12).

**Table 3:** Genes in the four new genome-wide-significant rare CNV loci

Gene	Gene name	Locus	Rare CNV type	EAS frequency	EUR frequency	OR	*P* value
ENSG00000173327	*MAP3K11*	11q13.1	Deletion	0.0065	0.00003	1.84	4.33 × 10^−6^
ENSG00000173338	*KCNK7*	11q13.1	Deletion	0.0074	0.00006	1.64	6.51 × 10^−5^
ENSG00000173442	*EHBP1L1*	11q13.1	Deletion	0.0076	0.00006	1.65	3.96 × 10^−5^
ENSG00000176973	*FAM89B*	11q13.1	Deletion	0.0061	0.00006	1.72	5.96 × 10^−5^
ENSG00000116032	*GRIN3B*	19p13.3	Deletion	0.0023	0.00000	3.31	1.10 × 10^−6^
ENSG00000182087	*TMEM259*	19p13.3	Deletion	0.0022	0.00000	3.74	2.12 × 10^−7^
ENSG00000167633	*KIR3DL1*	19q13.42	Deletion	0.0036	0.00000	0.42	5.38 × 10^−6^
ENSG00000189013	*KIR2DL4*	19q13.42	Deletion	0.0036	0.00000	0.42	5.38 × 10^−6^
ENSG00000158815	*FGF17*	8p21.3	Deletion	0.0014	0.00000	11.39	1.31 × 10^−7^
ENSG00000158856	*DMTN*	8p21.3	Deletion	0.0016	0.00000	9.09	8.14 × 10^−8^
ENSG00000158863	*FAM160B2*	8p21.3	Deletion	0.0012	0.00000	6.94	6.55 × 10^−7^
ENSG00000173566	*NUDT18*	8p21.3	Deletion	0.0015	0.00000	5.78	1.91 × 10^−7^

EAS frequency, carrier frequency in the East Asian samples; EUR frequency, carrier frequency in the European samples. *P* values are unadjusted and were assessed at the gene level using the logistic mixed-effects model in REGENIE (two sided). The genome-wide significance threshold and the definitions of new and genome-wide significant loci are provided in the Methods.

### Cross-population comparison of CNV effects on schizophrenia

We next examined the consistency of rare CNV effects across populations. Of the 31 schizophrenia-associated loci in the EUR populations (Supplementary Table 7), 24 had at least eight carriers in both the EUR and EAS datasets, allowing for association testing using REGENIE (Supplementary Table 13). Among these, 13 loci showed nominal significance (*P* < 0.05), and 17 had effect directions consistent with those observed in the EUR study. Across the 236 genes located within these 24 loci, we did not observe significant heterogeneity in the OR estimates between EAS and EUR populations (two-sided Cochran’s *Q* test; Fig. 3e and Supplementary Table 14), suggesting broad consistency in the effects of schizophrenia-associated rare CNVs across populations. We also note that some of the 31 loci were curated from earlier literature based on smaller sample sizes and only 8 of them reached genome-wide significance in the most recent published EUR study. This suggests that some loci may have weaker statistical support and therefore might not be expected to replicate in the current study.

Among the eight rare CNV loci that previously reached genome-wide significance in the PGC EUR study (Table 1 in ref. ^14^), seven had at least eight carriers in the EAS dataset. ORs for these loci were not significantly different between the two populations (*P* > 0.05, two-sample *z* test; Fig. 3d and Methods). Of these seven loci, three achieved genome-wide significance in EAS populations (22q11.21 deletion; 3q29 deletion; and 16p11.2, proximal duplication; *P* < 6.88 × 10^−5^) and an additional three reached nominal significance (1q21.1 deletion; 16p11.2, distal deletion; and 7q11.23 duplication; Supplementary Table 13). Across the 150 genes located within the seven loci, no significant heterogeneity in the OR estimates was observed (two-sided Cochran’s *Q* test; Supplementary Table 14), reinforcing the conclusion that the effects of schizophrenia-associated rare CNVs are largely consistent between EAS and EUR populations.

### Meta-analysis of rare CNV associations across EAS and EUR populations

To enhance discovery power and leverage the observed cross-population consistency in rare CNV effects on schizophrenia, we used a sample-size-weighted fixed-effect meta-analysis to combine the EAS and EUR datasets, totaling 38,409 cases and 40,009 controls. The analysis included 3,413 rare CNV genes, with the genome-wide significance threshold established using permutations of case–control labels in both datasets (*P*_cutoff_ = 3.17 × 10^−5^; Methods).

**Fig. 4:**
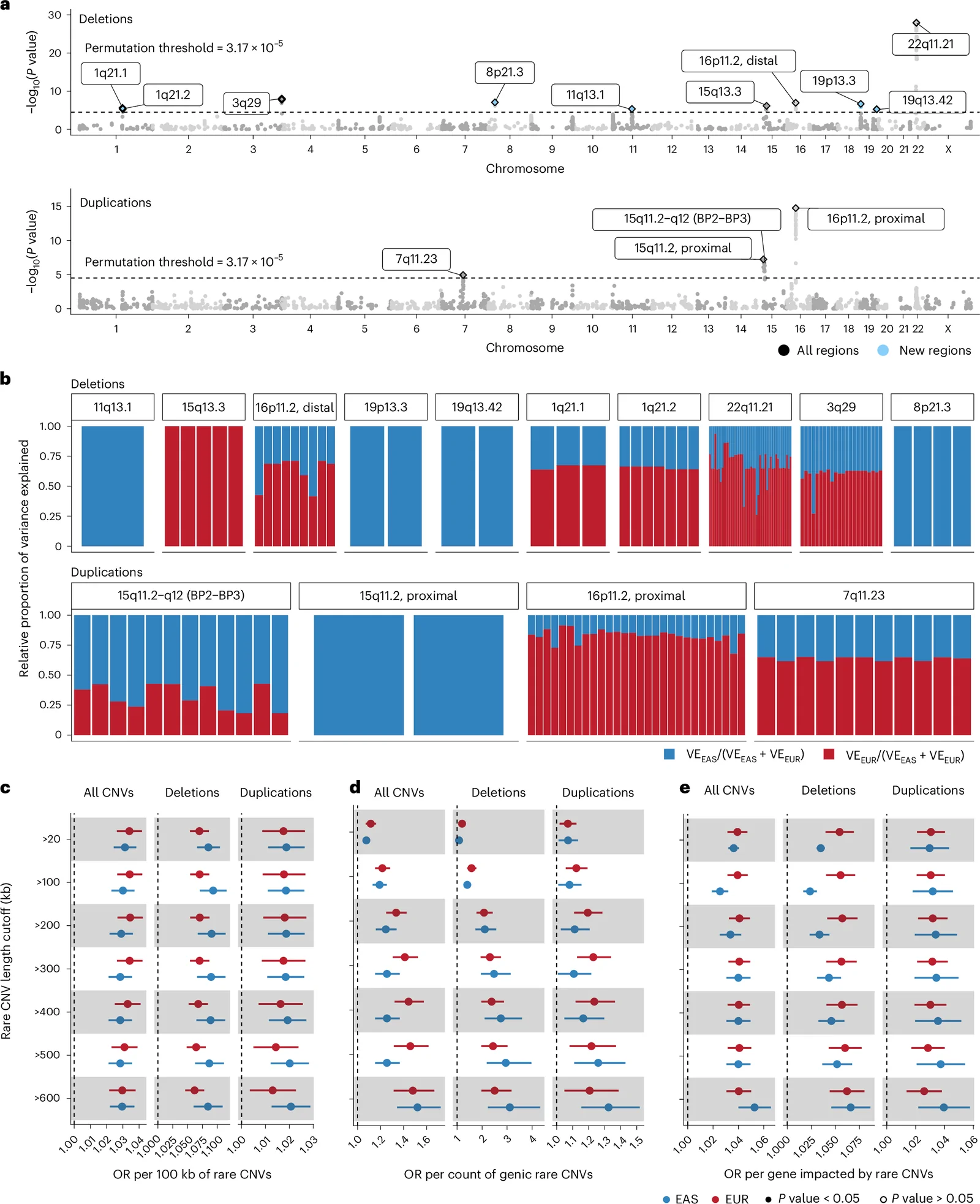
Gene-level rare CNV associations from the meta-analysis of EAS and EUR samples. **a**, Manhattan plots for rare CNV deletions and duplications. CNV loci reaching genome-wide significance are labeled. Genes within new loci are highlighted in blue. *P* values are unadjusted and were estimated using METAL (Methods). Deletions and duplications were analyzed separately. The empirical genome-wide-significant threshold for the meta-analysis was *P* < 3.17 × 10^−5^ based on the permutation test (Methods). **b**, Relative proportion of variance explained (VE) for EUR and EAS populations for the 155 genome-wide significant rare CNV genes, grouped by CNV loci. Note that a single locus may host multiple significant genes. **c**, OR per 100 kb of rare CNVs. **d**, OR per number of genic rare CNVs. **e**, OR per gene affected by rare CNVs. Results are shown for all rare CNVs, deletions and duplications. For **c**–**e**, horizontal error bars indicate 95% CIs. The vertical dashed line indicates OR = 1 (no effect). Sample sizes were as follows: *n* = 44,161 (EAS) and *n* = 34,257 (EUR) independent samples (unit of study: individual participants). *P* values are unadjusted and were estimated using the two-sided logistic mixed-effects model. The exact *P* values are provided in Supplementary Table 19.

The meta-analysis identified 155 rare CNV genes across 14 loci reaching genome-wide significance (Fig. 4a and Supplementary Table 15). We found four gene sets significantly enriched with these 155 genes that were not detected in either the published EUR study or our EAS-specific analysis (Supplementary Table 11 and Methods). Notable examples include gene sets for postsynaptic cytosol (GO:0099524, *P* = 0.011) and presynapse cellular compartment (GO:0098793, *P* = 0.018).

To quantify the contribution of each genome-wide-significant rare CNV gene, we calculated their variance explained, approximated as 2*f*(1 − f)(log(OR))^2^/(π^2^/3), where *f* is the carrier frequency. This metric, which incorporates both effect size and frequency, provides an estimate of the ‘importance’ of a significant rare CNV gene in a given population. We compared the variance explained between EAS and EUR populations for each of the 155 genome-wide significant rare CNV genes, illustrating relative contributions from the two populations to the discovery of the association (Fig. 4b).

Eight of the 14 rare CNV loci reached genome-wide significance for the first time, including deletions at 1q21.2, 8p21.3, 11q13.1, 15q13.3, 19p13.3 and 19q13.42 and duplications at 15q11.2, proximal, and 15q11.2–q12 (BP2–BP3). Intensity plots for these eight new loci are shown in Supplementary Data 1. All new loci were supported by CNV calls in at least two distinct samples and platforms, strengthening the reliability of these findings (Supplementary Fig. 4 and Supplementary Table 16). Validation of the eight new CNV loci using whole-exome sequencing (WES) and droplet digital PCR (ddPCR) was also successful on 100% of available samples (Methods, Supplementary Tables 17 and 18 and Supplementary Note).

### Schizophrenia-associated rare CNVs are under strong evolutionary constraint across populations

**Extended Data Fig. 7:**
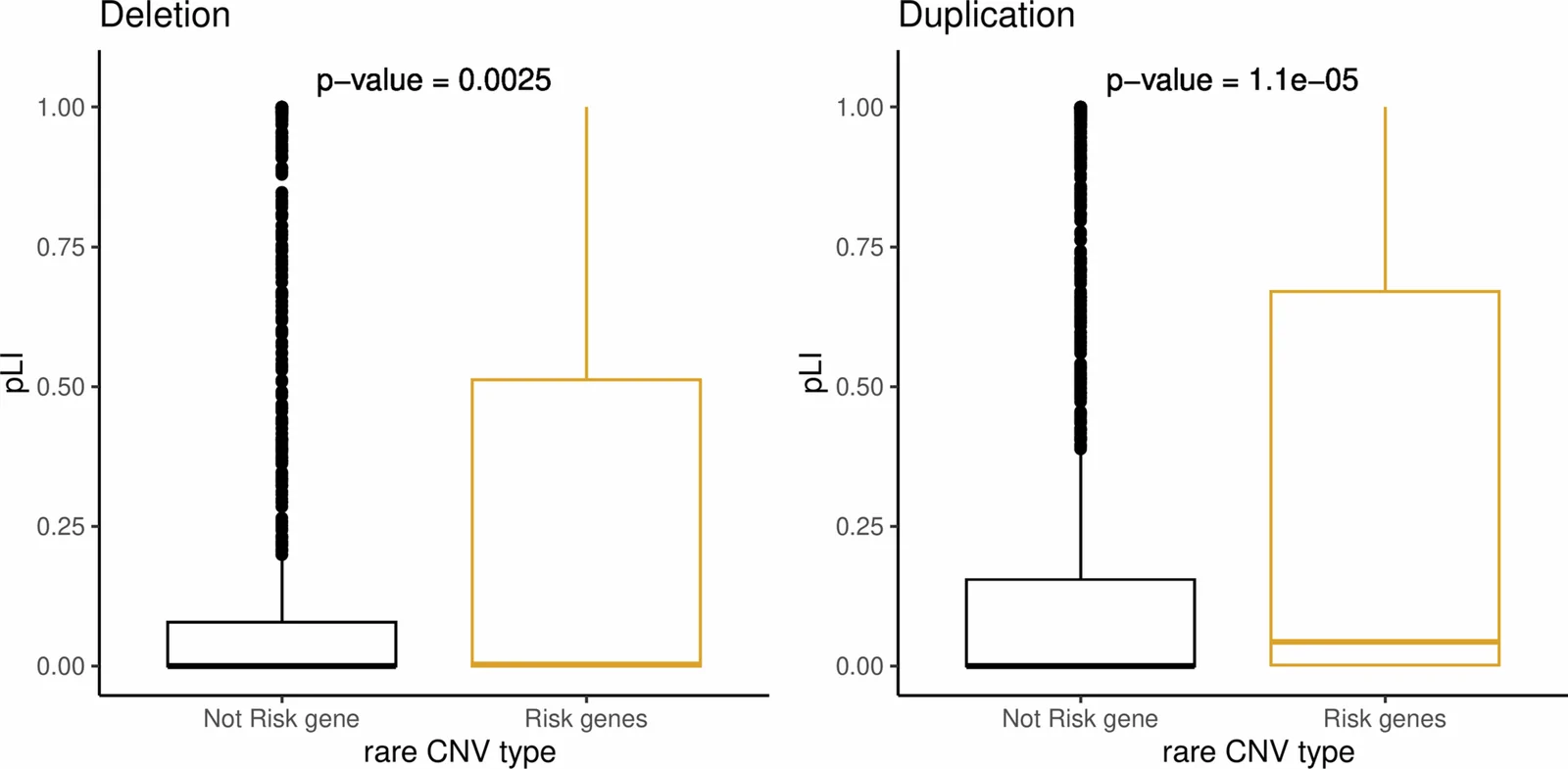
PLI for risk and non-risk rare CNV genes. Risk rare CNV genes were defined as genes achieving genome-wide significance in gene-based rare CNV association analyses; non-risk rare CNV genes include all other genes tested in this study. The unit of study is an individual gene, and each data point represents one gene with its pLI score. For deletions, n = 102 risk genes and n = 3,311 non-risk genes; for duplications, n = 53 risk genes and n = 3,360 non-risk genes. Center lines indicate the median; box bounds indicate the 25th and 75th percentiles; whiskers extend to 1.5× the interquartile range; points beyond the whiskers are shown as outliers. Two-sided Student’s t-tests were used to compare groups. Exact P values are shown in the figure. No multiple-comparison correction was applied.

Previous studies have shown that schizophrenia-associated genetic loci identified through rare coding variants are under strong evolutionary constraint^26^. We extended this analysis to rare CNVs by evaluating their burden in 3,063 genes intolerant to loss of function (LoF; probability of loss-of-function intolerance (pLI) > 0.9; Methods) in schizophrenia cases and controls. In the EAS dataset, we observed a significantly higher rare CNV burden in these constrained genes (OR = 1.036; 95% CI = 1.032–1.040; *P* = 1.16 × 10^−14^) than in genes with pLI < 0.9 (OR = 1.010; 95% CI = 1.006–1.013; *P* = 2.67 × 10^−7^). A total of 155 genes located within the genome-wide-significant rare CNV loci were also significantly enriched for LoF-intolerant genes (*P* = 2.86 × ^−16^, Fisher’s exact test) and had a significantly higher mean pLI score than other genes examined in this study (mean pLI: 0.28 versus 0.17; *P* = 0.001, two-sided *t* test). These patterns were consistent across both rare CNV deletions and duplications (Extended Data Fig. 7). In line with prior findings, we did not observe any statistically significant differences in rare CNV burden among constrained genes between EAS and EUR populations after accounting for multiple testing (Fig. 4c–e). For example, the total length of rare CNVs overlapping LoF-intolerant genes (filtered to >600 kb) was significantly associated with schizophrenia in both EAS (OR = 1.053; 95% CI = 1.040–1.065; *P* = 1.06 × 10^−16^) and EUR (OR = 1.040; 95% CI = 1.030–1.050; *P* = 1.55 × 10^−16^) populations (Supplementary Table 19).

## Discussion

We performed a large-scale study of rare CNVs in schizophrenia in participants of EAS ancestry, totaling 20,903 cases and 23,258 controls. When this sample was combined with 34,257 individuals of EUR ancestry, we identified 8 new rare CNV loci associated with schizophrenia at genome-wide significance, increasing the total number of known schizophrenia-associated rare CNV loci to 15. Four of these new loci—deletions at 8p21.3, 19p13.3, 11q13.1 and 19q13.42—were predominantly driven by substantially higher carrier frequencies in EAS populations. Power analysis suggests that detecting these loci in EUR populations would require over 400,000 schizophrenia cases to achieve 80% power in a balanced case–control design, assuming consistent effect sizes across ancestries (Methods). This highlights the importance of increasing ancestral diversity in genetic studies to fully capture the genetic underpinnings of complex diseases such as schizophrenia.

Our analyses also suggested that the effects of rare CNVs on schizophrenia are largely comparable between EAS and EUR populations. In particular, genome-wide rare CNV burden showed no significant differences between the two populations. At the locus level, the discovery of new CNV associations was primarily driven by differences in carrier frequencies rather than effect sizes. These observations mirror patterns seen in schizophrenia-associated common variants^15^, supporting a broadly shared genetic architecture of schizophrenia between EAS and EUR populations.

This study has several limitations. First, most genotyping arrays were designed for EUR populations, potentially biasing CNV detection in other ancestries. Future studies using unbiased technologies such as whole-genome sequencing will be essential for improved detection and characterization of structural variants. Second, despite the large sample size, statistical power remained limited for capturing rare CNVs with low carrier frequencies. Due to the small number of CNV carriers at some loci, Firth logistic regression was used to estimate ORs, which introduces shrinkage in the effect size estimates. Additionally, many standard error estimates were excessively large, preventing formal heterogeneity testing of ORs across the 15 EAS samples. Third, our control cohort included both screened individuals and population-based samples, with the majority drawn from population cohorts. This heterogeneity may influence the distribution of PRSs and potentially attenuate PRS differences between CNV carriers and noncarriers among controls. Last, age was not consistently ascertained across samples; as a result, we were unable to account for the potential influence of age in our analyses.

In summary, this study provides new insights into the genetic architecture of schizophrenia through one of the largest rare CNV analyses in EAS populations. Our results underscore the importance of including diverse populations in psychiatric genetics and demonstrate how non-EUR genomic resources can uncover new schizophrenia-associated rare CNV loci that may be missed in EUR-centric analyses. Broadening the ancestral representation in genomic research is essential for fully elucidating the genetic underpinnings of schizophrenia.

## Methods

### Ethics

The following institutions provided ethics oversight for EAS samples with schizophrenia used in this work: Samsung Medical Center, Bio-X Institutes of Shanghai Jiao Tong University, Xi’an Jiaotong University, The Second Xiangya Hospital of Central South University, Peking University Sixth Hospital, Fujita Health University, Tokyo Metropolitan Institute of Medical Science, University Medical Center Utrecht, The University of Western Australia, The University of Indonesia, RIKEN Center for Integrative Medical Sciences, Nagoya University, Osaka University, Niigata University, Chonnam National University Hospital and Mass General Brigham (protocols 2014P001342 and 2011P002207). Informed consent and permission to share the data were obtained from all participants in compliance with the guidelines specified by the recruiting center’s institutional review board. We have all relevant approvals from China’s Ministry of Science and Technology for the export of genetic information and materials related to this work.

### Study samples

The samples of EAS ancestry are summarized in Table 1, with detailed descriptions provided in the Supplementary Note. Following rare CNV calling and QC, 44,161 participants were retained for analysis, including 20,903 schizophrenia cases and 23,258 controls. EUR samples were obtained from a previously published analysis by the PGC CNV Working Group^14^, comprising 34,257 participants (17,506 cases and 16,751 controls).

### Rare CNV calling and QC in EAS samples

Rare CNV calling, QC and downstream analyses were conducted using a pipeline implemented on the Google Cloud Platform. Full details are available in the Supplementary Note. For each participant, CNVs were called using two algorithms, PennCNV^17^ and iPattern^18^, and only CNVs detected by both algorithms were retained for analysis. Participant-level QC excluded arrays with excessive probe variance or G+C bias, as well as participants with chromosomal aneuploidies or mismatches between genetically inferred and self-reported sex or ancestry (Supplementary Note). We used a standard format for rare CNVs, as described in PLINK^34^ (https://zzz.bwh.harvard.edu/plink/).

### Rare CNV detection

The high-quality rare CNVs were selected based on the following criteria: (a) length of more than 20 kb; (b) support by at least 15 probes to ensure the robustness of CNV calls; (c) frequency of less than 1% across the combined sample set; (d) detection in at least two samples; and (e) detection by both CNV calling algorithms. The final callset was annotated using Ensembl gene lists. A gene was considered affected if a rare CNV physically overlapped its exonic region. We note that the previous PGC CNV study^14^ in EUR participants used a ten-probe threshold for CNV detection. We used a more stringent threshold in the EAS analysis because CNVs supported by 10–14 probes had lower WES validation rates than those detected with 15 or more probes (Supplementary Note and Supplementary Fig. 5).

### Validation against PGC study pipeline

To validate our rare CNV calling and QC pipeline, we leveraged two EUR samples, ‘swe5’ and ‘swe6’, previously analyzed in the published PGC study^14^. Both samples were genotyped using the Illumina HumanOmniExpress array, and they comprised 3,309 (swe5) and 1,645 (swe6) participants. We benchmarked our rare CNV calls against the PGC-reported CNV calls using bcftools (v1.19)^35^. Two CNVs were considered concordant if they overlapped by >75%. We evaluated the concordance using both the 10-probe and 15-probe thresholds in our pipeline and found that the results were highly consistent (Supplementary Table 3).

### Rare CNV burden test

Genome-wide rare CNV burden was analyzed using a mixed-effects model implemented in the lme4qtl R package^36^:

$${\mathbf{y}} = X{\mathbf{\upbeta}} + {Z}{\mathbf{u}} + {\mathbf{e}},$$

where **y** is the schizophrenia case–control status; *X* is a matrix of fixed effects, including rare CNV burden and covariates (sex, genotyping platform and top five genetic PCs); **β** is the corresponding vector of fixed-effect coefficients; *Z* is the design matrix for random effects; **u** is the vector of random effects, assumed to follow a multivariate normal distribution ${\textbf{u}} \sim N({\textbf{0}},{\sigma }_{{\textrm{g}}}^{2}\times A)$, in which *A* is the full genetic relationship matrix constructed from genotype data; and **e** is a vector of residuals with ${\textbf{e}} \sim N({\textbf{0}},{\sigma }_{{\textrm{e}}}^{2}\times I)$, in which *I* is an identity matrix. **u** and **e** were assumed to be independent.

### Gene-level rare CNV association analysis

Gene-level rare CNV association analysis was conducted using REGENIE^23^, adjusting for sex, genotyping platform and top genetic PCs as covariates. REGENIE uses a two-step procedure for association testing. In step 1, a polygenic effect was estimated using linkage disequilibrium (LD)-pruned common variants (minor allele frequency of > 1%; missing rate of <0.1%) that were imputed across all samples. In step 2, a Firth-regularized logistic regression model was fitted to test the association between each gene and schizophrenia case–control status, with the polygenic effect estimated in step 1 included as an offset to account for sample relatedness. A gene was considered affected and coded as ‘1’ for a participant if a CNV of the relevant type (deletion or duplication) overlapped its coding region. Genes that did not overlap any CNVs of the relevant type were coded as ‘0’. We tested only genes affected by rare CNVs in at least eight participants. CNV loci were defined by aggregating genes affected by rare CNVs with >50% overlap across participants. We annotated the CNV loci using cytoband annotations from the UCSC Genome Browser^37^.

### Establishing genome-wide significance thresholds

To empirically determine the genome-wide significance threshold for the gene-level association analyses, we permuted case–control labels 100 times, recalculated gene-level association statistics and recorded the minimum *P* value across all genes for each permutation. The genome-wide significance threshold was defined as the 5th percentile of this null distribution, corresponding to a family-wise error rate of 5%.

To reduce computational cost, samples from trio-based studies (tai2trios, taiml and tai3) were excluded from permutation analyses, resulting in a slight reduction in the number of genes being tested—73 (~3%) of the 2,183 genes in the EAS analysis and 56 (~1%) of the 3,413 genes in the meta-analysis. The final empirical genome-wide significance thresholds were 6.88 × 10^−5^ for the EAS analysis and 3.17 × 10^−5^ for the meta-analysis. 

### Previously reported schizophrenia-associated loci

We created a list of previously reported schizophrenia-associated CNV loci (Supplementary Table 7) by combining (1) the eight genome-wide-significant loci from the latest PGC CNV study^14^ and (2) additional loci in the ‘list of reported CNV associations to schizophrenia’ compiled in the same publication (Table 1 and Supplementary Table 1 in ref. ^14^). CNV locus coordinates were harmonized to the hg19 reference genome using liftover^38^. The largest prior CNV study in the Chinese population^16^ did not yield any genome-wide-significant findings and thus was not included.

### SynGO gene set test

We assessed the enrichment of synapse-related biological functions using the SynGO ontology terms^24^ and gene-level CNV associations from the EAS, EUR and EAS + EUR meta-analyses. Gene sets were obtained from the SynGO portal (https://www.syngoportal.org/), including 135 defined through ‘biological processes’ and 60 defined through ‘cellular components’. To specifically assess synaptic-related signals while controlling for general brain expression, we restricted our analysis to 10,360 genes with detectable expression in brain tissues based on publicly available transcriptomic data^39^. For each gene set, we applied a one-sided Fisher’s exact test (greater-than alternative) to assess its enrichment.

### Annotation of CNV formation mechanisms

To investigate the formation mechanisms of significant rare CNV loci, we followed previously established approaches and created 55-kb windows around the start and end coordinates of each locus. Specifically, the start window extended from –50 kb to +5 kb relative to the CNV’s start position, and the end window spanned from –5 kb to +50 kb relative to the CNV’s end position. CNVs were classified as NAHR mediated if both the start and end windows overlapped segmental duplications as annotated in the hg19 build of the UCSC Table Browser (‘Segmental Dups’ track in https://genome.ucsc.edu/cgi-bin/hgTables) and filtered to include only duplications ≥1 kb in size. CNVs were classified as NHEJ mediated if they overlapped previously reported NHEJ-associated rearrangements, including 26 Alu-mediated deletions (NHEJ-ARMDs) and 10 L1 retrotransposon-associated deletions (NHEJ-L1RADs)^40^.

### Genetic effect comparison between EAS and EUR populations

To compare the effects of rare CNVs between EAS and EUR populations, we performed a two-sample *z* test on log-transformed OR estimates:

$$Z=\frac{\mathrm{ln}\left({\mathrm{{OR}}}_{{{\textrm{EAS}}}}\right)-\mathrm{ln}\left({\mathrm{{OR}}}_{{{\textrm{EUR}}}}\right)}{\sqrt{\frac{\mathrm{{{SE}}}_{{{\textrm{EAS}}}}^{2}}{{N}_{{{\textrm{EAS}}}}}+\frac{\mathrm{{{SE}}}_{{{\textrm{EUR}}}}^{2}}{{N}_{{{\textrm{EUR}}}}}}}$$

where SE^2^_EAS_ and SE^2^_EUR_ are the standard errors of the log-transformed effect sizes and *N*_EAS_ and *N*_EUR_ are the respective sample sizes. A *P* value was then calculated from the *z* statistic.

### PRS calculation

We applied PRS-CS^21^ to estimate the posterior effect sizes of genetic variants using summary statistics from the EAS schizophrenia GWAS^15^. The LD reference panel was precomputed using 1000 Genomes Project phase 3 EAS samples (https://github.com/getian107/PRScs). The global shrinkage parameter was set to 0.01. For each target participant, the PRS of each chromosome was calculated using the ‘--score’ function in PLINK2(ref. ^41^), which computes a linear combination of genotypes weighted by the estimated effect sizes. Strand-ambiguous variants and the MHC region were excluded. The final genome-wide PRS was obtained by summing the scores over chromosomes 1 to 22. The PRS was calculated for participants who were not part of the original GWAS study. The PRS target dataset included 14,788 schizophrenia cases and 15,671 controls from eight samples: sxyh1, tai2t, taiml, tmim1, tmim3, xajd, xju2 and push2. We standardized the PRS to have a mean of 0 and an s.d. of 1.

### Collective contribution from PRS and rare CNV burden to schizophrenia risk

We fitted a series of logistic regression models to investigate the individual and collective effects of PRS and rare CNV burden on schizophrenia risk:

1 $$Y \sim {\beta }_{0}+C$$

2 $$Y \sim {\beta }_{0}+X+C$$

3 $$Y \sim {\beta }_{0}+{{\textrm{PRS}}}+C$$

4 $$Y \sim {\beta }_{0}+X+{\mathrm{PRS}}+C$$

5 $$Y \sim {\beta }_{0}+X+{\mathrm{PRS}}+{\mathrm{PRS}}\times X+C$$

where *Y* is the schizophrenia case–control status; *X* is the rare CNV burden, measured as (1) the total length of rare CNVs; (2) the number of genic rare CNVs; and (3) the number of genes affected by rare CNVs; *β*_0_ is the intercept; *C* is a set of covariates including sex, genotyping platform and the top five PCs derived from the genetics data; and PRS is the polygenic risk score.

Model (1) served as the null model. To evaluate the predictive power of rare CNV burden and PRS individually, we estimated their incremental *R*^*2*^ on the observed scale by comparing models (2) and (3) with model (1), respectively, and subsequently transformed *R*^*2*^ to the liability scale following ref. ^42^, assuming a schizophrenia population prevalence of 1%. To assess the additional contribution of rare CNV burden to schizophrenia risk beyond PRS, we compared model (4) with model (3) and estimated the incremental *R*^*2*^ on the liability scale. We used model (5) to test the interaction between rare CNV burden and PRS. For interpretability, we standardized the CNV burden to have a mean of 0 and an s.d. of 1.

### Meta-analysis of gene-level associations

We performed a meta-analysis to combine gene-level association results from the EAS and EUR analyses using a sample-size-weighted fixed-effect model implemented in METAL^43^. Rare CNVs from the EUR samples, originally mapped to hg18 coordinates, were converted to hg19 coordinates using the UCSC liftover tool. Genes were annotated using Ensembl IDs. All genes with association results from either the EAS or EUR analyses were included in the meta-analysis.

### CNV calling using WES data

To validate array-detected rare CNVs, we leveraged existing WES data from a subset of 16,623 individuals. CNV analysis on the exome data was performed using GATK-gCNV^44^, a Bayesian framework designed for accurate CNV discovery from sequencing data. This method accounts for inherent biases in exome capture and sequencing, such as G+C content, and uses statistical models to adjust for technical variations across samples. Exon-level read counts were generated from the raw sequencing data following the established WES processing pipeline^44^. Stringent QC measures were applied, where we removed participants who had too many calls (>200 raw autosomal calls or >35 calls with a QS score of >20), variants that did not achieve a sufficient score as recommended by GATK-gCNV (a variable threshold based on the size of the event), variants that did not span three or more exons and intervals, and variants that were called in more than 1% of the samples. A CNV identified from array data was considered validated if at least 75% of the individuals carrying the CNV based on array data had a concordant GATK-gCNV call (that is, the same CNV type of deletion or duplication) that overlapped at least 75% of the targeted genomic region.

### Experimental validation of rare CNV calls by ddPCR

To experimentally validate rare CNV calls, probe-based ddPCR assays targeting selected CNV regions were designed. Each assay included forward and reverse primers and a FAM-labeled probe, which were synthesized by Integrated DNA Technologies. Details of the assays are provided in Supplementary Table 18a. For each locus, we selected three noncarriers as negative controls. A reference assay amplifying the housekeeping *RPP30* gene was purchased from Bio-Rad (10031243). ddPCR reactions were performed using 2× ddPCR Supermix for Probes (no dUTP) (Bio-Rad). First, each assay was validated by confirming a target copy number of 2 for the target in the control DNA isolated from the highly characterized GM08330 cell line. Individual ddPCR reactions were prepared in a 20-μl solution consisting of 10 μl of 2× Supermix, 0.5 μl FAM-labeled 20× target assay, 0.5 μl HEX-labeled 20× reference assay, 2.5 U of HaeIII restriction enzyme (NEB), 20 ng of DNA and nuclease-free water. Reaction mix was transferred to the Bio-Rad QX200 automated droplet generator to produce approximately 20,000 droplets per reaction using the Bio-Rad droplet generator cartridge (1864109) and oil for probes (1864110) following the manufacturer’s instructions. After droplet generation, the 96-well plate was heat sealed and transferred to the Mastercycler X50s (Eppendorf) for ddPCR reaction, starting with an initial activation at 95 °C for 10 min, followed by 45 cycles of 94 °C for 30 s, 60 °C for 60 s with ramp up of 2 °C s^−1^, deactivation for 10 min at 98 °C and a final hold at 4 °C. Lastly, ddPCR droplets with amplicons were read on the Bio-Rad QX200 droplet reader, and copy numbers were calculated using QuantaSoft software from Bio-Rad.

### Sample size estimation

We followed the method described in ref. ^45^ to estimate the sample size needed to achieve 80% power for detecting a rare CNV association. Specifically, the number of cases needed to detect an association was estimated using the likelihood ratio test based on CNV frequency and effect size:

$${n}_{a,b}\approx \frac{{\nu }_{a,b}}{4{f}_{{\textrm{c}}}g\left({\lambda }_{{\textrm{c}}}\right)},$$

where *f*_c_ is the frequency of the rare CNV in the population, *λ*_c_ is the OR, (g)*λ*_c_ is a function of *λ*_c_: *g*(*λ*) = ((l + *λ*)ln(1 + *λ*) – *λ*) and *V*_*a*,*b*_ is a constant that controls the desired false-positive rate *a* and false-negative rate *b*. In this study, the false-positive rate *a* was set to the genome-wide significance threshold, and the false-negative rate *b* was set to 20% to achieve 80% power.

### Constrained genes selection

We used the pLI scores from the gnomAD v2 database^46^ to quantify the selective constraint for each gene. A total of 3,063 genes with pLI of >0.9 were classified as LoF intolerant. These constrained genes are typically under strong purifying selection, with LoF mutations being selectively removed from the population over time.

### Rare CNV burden enrichment analysis in constrained genes

We assessed rare CNV burden enrichment in constrained genes using a mixed-effects model, as previously described in the genome-wide rare CNV burden analysis:

$${\mathbf{y}} = X{\mathbf{\upbeta}} + Z{\mathbf{u}} + {\mathbf{e}},$$

where **y** is the schizophrenia case–control status; *X* is the matrix of fixed effects, including rare CNV burden of the constraint genes—measured by (1) the total length of rare CNVs overlapping constrained genes; (2) the number of rare CNVs overlapping constrained genes; and (3) the total number of constrained genes affected by rare CNVs—along with covariates (sex, genotyping platform and the top five genetic PCs); **β** is the vector of fixed-effect coefficients; *Z* is the design matrix for random effects; **u** is the random effect vector that follows a multivariate normal distribution ${\textbf{u}} \sim N({\textbf{0}},{\sigma }_{{\textrm{g}}}^{2}\times A)$, in which *A* is the genetic relationship matrix constructed from genotype data and **e** is a vector of residuals with ${\textbf{e}} \sim N({\textbf{0}},{\sigma }_{{\textrm{e}}}^{2}\times I)$, in which *I* is an identity matrix. **u** and **e** were assumed to be independent.

### Reporting summary

Further information on research design is available in the Nature Portfolio Reporting Summary linked to this article.

## Online content

Any methods, additional references, Nature Portfolio reporting summaries, source data, extended data, supplementary information, acknowledgements, peer review information; details of author contributions and competing interests; and statements of data and code availability are available at https://doi.org/10.1038/s41588-026-02732-6.

## Supplementary information

Supplementary Information Supplementary Note, Figs. 1–6, Supplementary Data 1 and 2 legend.

Reporting Summary

Peer Review File

Supplementary Tables Supplementary Tables 1–19

Supplementary Data 1 Signal intensity for 8p21.3 (pages 1–3), 19q13.42 (pages 4–9), 1q21.2 (pages 10 and 11), 11q13.1 (pages 12–16), 15q11.2 (pages 17 and 18), 15q12 (pages 19 and 20), 15q13.3 (page 21) and 19p13.3 (pages 22 and 23). Log R ratio (LRR) values are shown for CNV carriers across a region defined as the new CNV locus ± 5 × its length. Each dot represents the LRR at an individual probe, with the *x* axis indicating genomic position and the *y* axis showing the LRR. Red points highlight probes within the new CNV locus.

Supplementary Data 2 Read depth profiles of participants for all WES-validated CNVs. Both panels show the normalized copy number (*y* axis) against genome position (*x* axis, left panel) and exome position (*x* axis, right panel). We merged CNVs on the same chromosome for the single participant. The gray shading represents the distribution of the underlying data points from the reference participant, typically centered around the diploid state (copy number = 2). Colored line segments show the estimated copy number state across each segment, with a solid dot indicating the mean copy number. Blue segments indicate increased copy number (duplication), while red segments indicate decreased copy number (deletions).

## Figures and Tables

**Extended Data Table 1:**
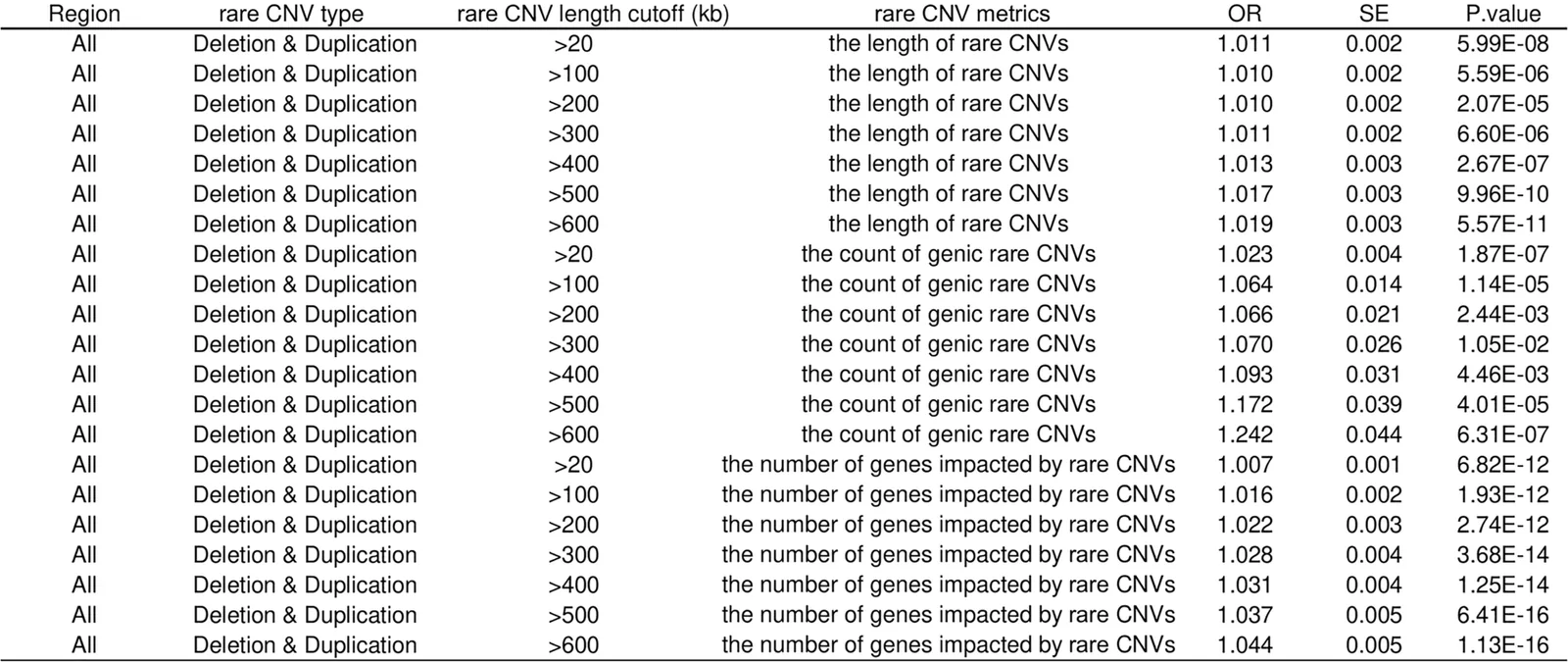
Genome-wide rare CNV burden in EAS

OR indicates the odds ratio per 100 kb of rare CNVs for “the length of rare CNVs”, per count of genic rare CNVs for “the count of genic rare CNVs”, per gene impacted for “the number of genes impacted by rare CNVs”.

**Extended Data Table 2:**
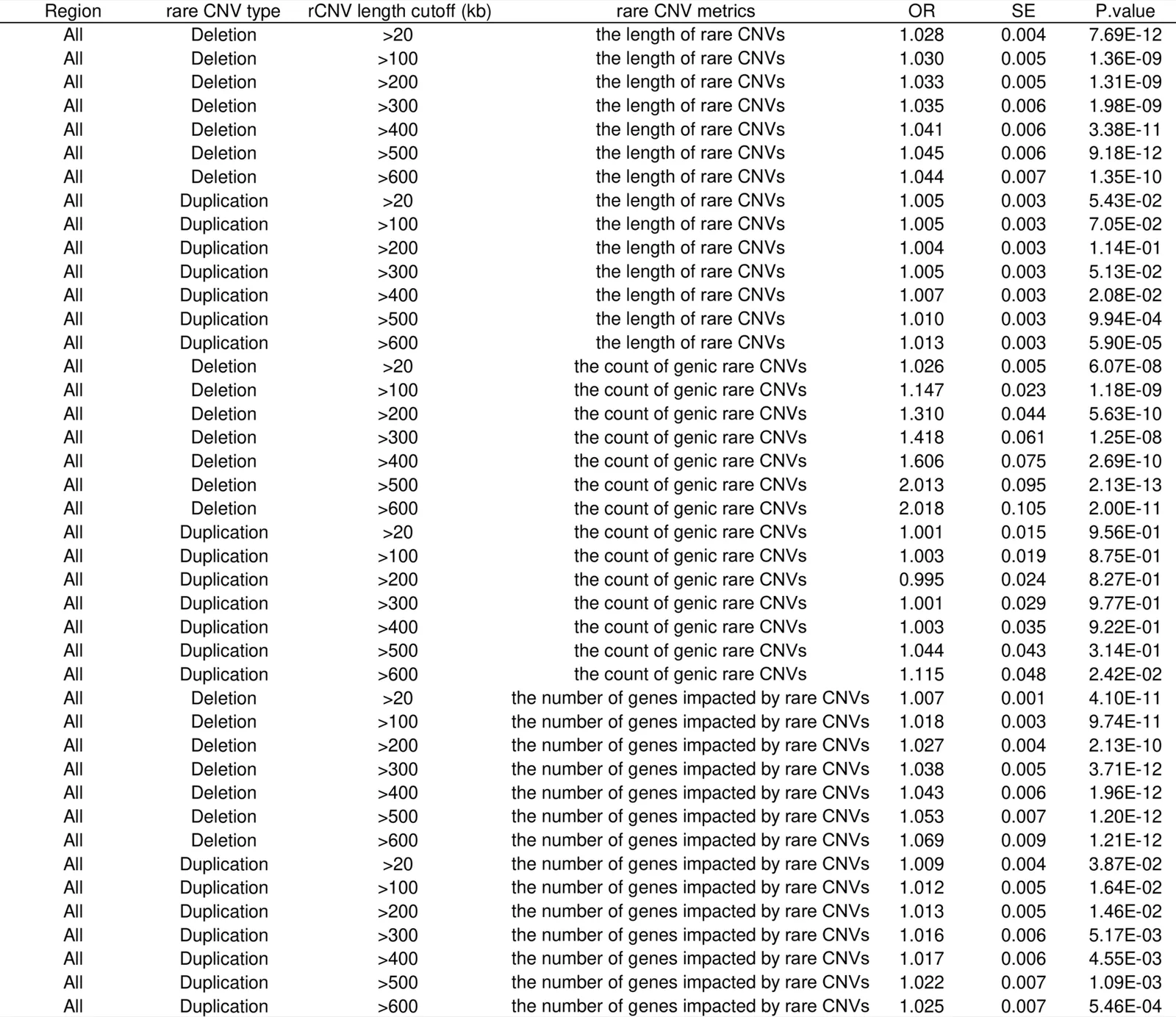
Genome-wide rare CNV burden in EAS partitioned by different rare CNV types

OR indicates the odds ratio per 100 kb of rare CNVs for “the length of rare CNVs”, per count of genic rare CNVs for “the count of genic rare CNVs”, per gene impacted for “the number of genes impacted by rare CNVs”.

**Extended Data Table 3:**
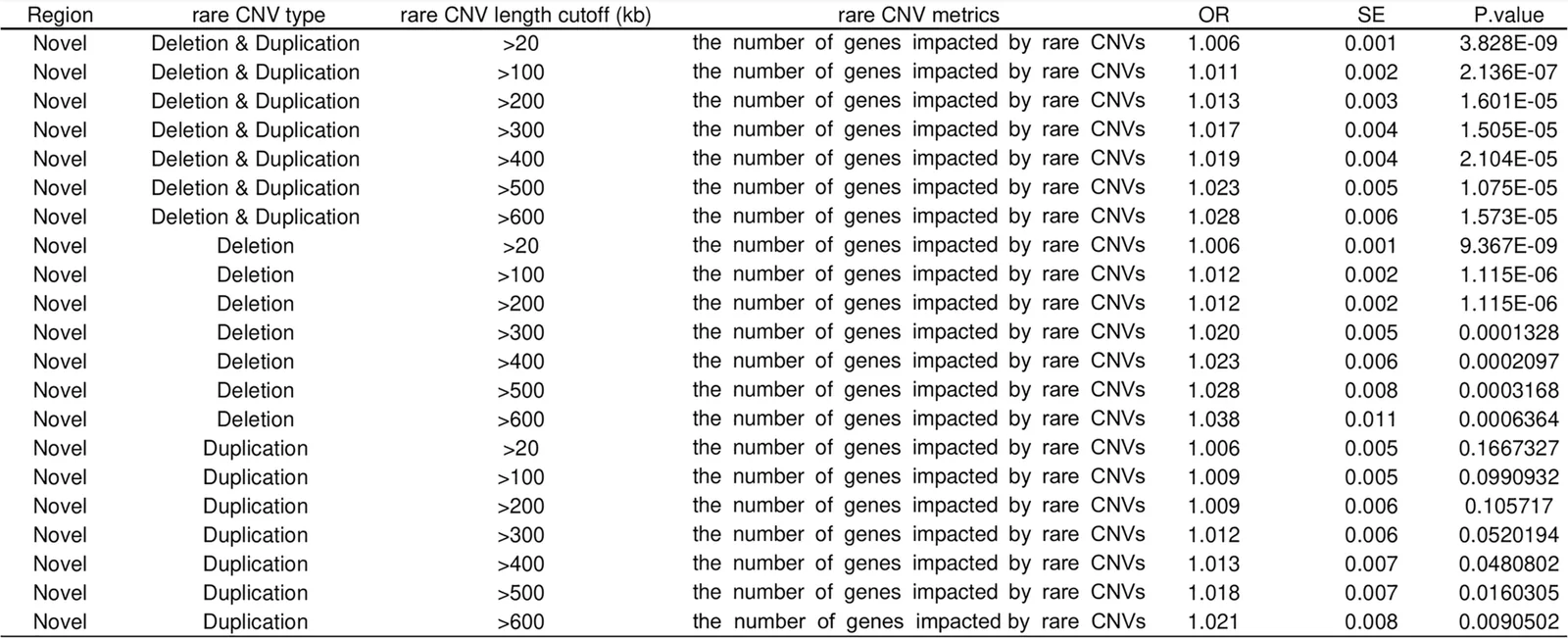
Genome-wide rare CNV burden excluding previously implicated loci in EUR

OR indicates the odds ratio per gene impacted for “the number of genes impacted by rare CNVs”.

## Data Availability

The summary statistics from this study are available at https://doi.org/10.5281/zenodo.19057959 (ref. ^47^). For individual-level data, tmim1 and tmim3 can be accessed at the Data Use Oversight System (DUOS; https://duos.broadinstitute.org/) under DUOS-005863, DUOS-005864, DUOS-005865, DUOS-005866, DUOS-005867, DUOS-005868, DUOS-005869, DUOS-005870, DUOS-005871, DUOS-005872, DUOS-005873, DUOS-005874, DUOS-005875, DUOS-005876, DUOS-005877 and DUOS-005878, and tai3 can be accessed at https://dbgap.ncbi.nlm.nih.gov/home/ under phs001196 and phs001413. The conditions of access, the timeframe for response and the restrictions for the following samples were described in an earlier publication^48^: imh1, imh2, uwa1, six2, xju1 and biox4. The conditions of access, the timeframe for response and the restrictions for the rest of the samples are described in the Supplementary Information.

## Code availability

Computer code relating to this study includes the following: PennCNV v1.0.5, https://penncnv.openbioinformatics.org/en/latest/; iPattern v0.582, https://www.tcag.ca/tools/index.html; RICOPILI v2019_Jun_25.001, https://sites.google.com/a/broadinstitute.org/ricopili; Plink-1.07-x86_64, https://zzz.bwh.harvard.edu/plink/; Bcftools v1.19, http://samtools.github.io/bcftools/bcftools.html; METAL v2011_03_25, https://csg.sph.umich.edu/abecasis/Metal/index.html; REGENIE v3.2.2, https://rgcgithub.github.io/regenie/; and Lme4qtl v0.1.10, https://github.com/variani/lme4qtl. Scripts used for CNV calling and QC in the production of the EAS CNV dataset are publicly available at https://github.com/QidiFeng/cnv_calling_QC_association. Custom scripts^49^ used for downstream analysis and visualization of the EAS CNV dataset are available at https://github.com/yu-1011/EAS_SCZ_rCNV_analysis or https://doi.org/10.5281/zenodo.20802211.
