# Supplementary material for: Contribution of copy number variants to schizophrenia in East Asian populations
Source: Nat Genet. 2026 Sep 11;58(10):2514–24. doi: 10.1038/s41588-026-02732-6 (PMC13649597; doi:10.1038/s41588-026-02732-6)
Supplement: Supplementary file 1 [file 41588_2026_2732_MOESM1_ESM.pdf]

nature genetics

Supplementary information https://doi.org/10.1038/s41588-026-02732-6

Contribution of copy number variants to schizophrenia in East Asian populations

In the format provided by the authors and unedited

Supplementary Information

Table of contents

Table of contents 1

SUPPLEMENTARY NOTE 3

Sample collection information 3

Sample collection: imh1 and imh2 3

Sample collection: biox4 and xju1 3

Sample collection: tmim1 and tmim3 3

Sample collection: tai3 and tai2trios 4

Sample collection: six2 4

Sample collection: uwa1 4

Sample collection: sxyh1 5

Sample collection: xajd and xju2 5

Sample collection: tai1multiplex 5

Sample collection: push2 6

Rare CNV calling 6

Input preprocessing of CNV calling 6

Composite CNV calling pipeline 7

Quality control of samples. 7

Quality control of CNV callset. 7

Quality control of genotype data. 7

Principal component analysis and kinship matrix estimation. 7

CNV annealing and aneuploidy. 7

Number of probes as the filter 8

Impact of probe thresholds on the difference between EAS and EUR CNV callsets 8

CNV length 8

Count of rare CNV per Carrier 8

Rare CNV burden by genotyping platforms 8

Impact of LRR-SD 8

WES validation of array-based CNV calls 9

Novel risk rare CNV genes of interest 9

REFERENCES 11

Supplementary Figures 13

Supplementary Figure 1. The distribution of sample QC metrics across EAS samples. 13

Supplementary Figure 2: Impact of LRR_SD on rare CNV burden test. 14

Supplementary Figure 3: The proportion of schizophrenia cases across PRS percentiles, stratified by rare CNV carrier status. 15

Supplementary Figure 4: Locus-wide rare CNV burden stratified by samples. **16**

Supplementary Figure 5: Validation of WES-derived CNV calls partitioned by the number of overlapping exons **17**

Supplementary Figure 6. Risk CNV duplications at chromosome 15q **18**

Supplementary Data **19**

SUPPLEMENTARY NOTE

Sample collection information

Sample collection: imh1 and imh2
Type: Case-Control
Contact: Liu, Jianjun (liuj3@gis.a-star.edu.sg) and Lee, Jimmy (jimmy.lee@nhghealth.com.sg)
Description: Subjects were part of the Singapore Translational and Clinical Research in Psychosis (STCRP) Study and comprised of cases and healthy controls of Chinese ancestry. Schizophrenia patients were recruited from the Institute of Mental Health, Singapore. Diagnosis of schizophrenia was made using the Structured Clinical Interview for DSM-IV, Research Version, Patient Edition (SCID-I/P) by trained raters. And healthy controls were screened using the DSM-IV, Research Version, Non-Patient Edition (SCID-N/P). All participants gave consent according to IRB/DSRB guidelines prior to participating.

Sample collection: biox4 and xju1
Type: Case-Control
Contact: Qin, Shengying (chinsir@sjtu.edu.cn)
Description: **biox4**: Subjects were recruited from the Shanghai Mental Health Center, Shanghai Jiao Tong University School of Medicine between August 2015 and August 2017. Patients were diagnosed by at least two experienced psychiatrists according to the DSM-IV based on structured Clinical Interview for DSM-IV Axis 1 Disorders (SCID-1). Healthy controls were selected from the general public of the Chinese population in Shanghai. The control subjects were also excluded with major mental illness and matched with patients in terms of age and gender. All the subjects were Han Chinese in origin. The study was approved by the Ethical Committee of Human Genetic Resources in Shanghai. All subjects or their guardians understood the procedure and gave written informed consent, according to the Declaration of Helsinki. **xju1**: Schizophrenia patients were recruited from the inpatients in nine clinical centers in Shaan Xi Province, China, including 1) Department of Psychiatry of the First Affiliated Hospital of Xi'an Jiaotong University (TFAHXJTU) and Mental Health Centers of 2) Baoji City, 3) Xianyang City, 4) Weinan City, 5) Hanzhong City, 6) Huayin City, 7)Yulin City, 8)Yanan City. Healthy controls were recruited from native residents of communities and villages in the city where each clinical center is located. Two professional psychiatrists interviewed all participants and evaluated their mental status according to DSM-IV criteria using SCID and/or Mini-International Neuropsychiatric Interview. The present study was approved by the Medical Ethics Committee of TFAHXJTU. All subjects participated voluntarily and signed written informed consent prior to the enrollment.

Sample collection: tmim1 and tmim3
Type: Case-Control
Contact: Sawa, Akira (asawa1@jhmi.edu)
Description: All the subjects were recruited from multiple university hospitals and local hospitals in Japan. Patients were diagnosed according to the Diagnostic and Statistical Manual of Mental Disorders, 4th Edition (DSM-IV), with consensus from at least two experienced psychiatrists. All patients agreed to participate in the study and provided written informed consent. The study was approved by the Institutional Review Boards of the Tokyo Metropolitan Institute of Medical Science and all affiliated institutions. DNA samples were genotyped on the Illumina Infinium Global Screening Array-24 v.1.0 (GSA) BeadChip at the Broad Institute, using standard reagents and HTS workflow procedures.

Sample collection: tai3 and tai2trios
Type: trios
Contact: Tsuang, Ming (mtsuang@ucsd.edu) and Chen, Wei J. (wjchen@nhri.edu.tw)
Description: Schizophrenia probands and their first-degree relatives were recruited from Schizophrenia Trio Genomic Research in Taiwan (S-TOGET) funded by the National Institute of Mental Health in the United States. Schizophrenia patients were interviewed with the Diagnostic Interview for Genetic Studies (DIGS), which was designed specifically for family-genetic studies of schizophrenia and bipolar disorder. Research diagnostic assessment was made independently by two board-certified psychiatrists based on integrated clinical information of DIGS interview data and summary notes of clinical course, symptom manifestations, and social functioning derived from the records of the medical charts according to DSM-IV for schizophrenia. This study was approved by the institutional review boards of the participating hospitals. Written informed consent was obtained from all subjects after a complete description. Study details have been reported elsewhere (Rees et al., 2015; Wang et al., 2017; Wang et al., 2019; Daniel P. Howrigan et al., 2020). The recruited trios had been genotyped using the Illumina PsychChip array funded by PGC, the Stanley Center for Psychiatric Research, the Friedman Brain Institute, and the Icahn Institute for Genomics and Multiscale Biology at Mount Sinai (i.e., tai3 sample). tai3 can be accessed at https://dbgap.ncbi.nlm.nih.gov/home/ under phs001196, phs001413. Access to tai2trios requires the submission of a research proposal and evidence of ethics approval to the contact authors. Requests will be reviewed by the contact authors, with decisions typically provided within two weeks. Reasonable requests accompanied by the required regulatory approvals will generally be approved.

Sample collection: six2
Type: Case-Control
Contact: Kahn, Rene (rene.kahn@mssm.edu) and Yu, Xin (yuxin@bjmu.edu.cn)
Description: Funded by the Division of Neuroscience of the University Medical Center in Utrecht (UMC, Utrecht), a collaborative genetic study was developed between UMC Utrecht and Peking University Institute of Mental Health (PKUIMH). The goal of the study was to identify susceptibility genes that contribute to the development of schizophrenia and related disorders. Subjects were recruited from thirteen major cities across mainland China. All patients were interviewed with Structured Clinical Interview for DSM-IV axis I Disorder (SCID–I) by extensively trained Chinese psychiatrists and researchers involved in the study. All included patients fulfilled DSM-IV criteria for schizophrenia. The SCID interviews were tape-recorded and randomly checked by senior psychiatrists not involved in the recruitment of interviewing. Healthy controls were matched with patients in terms of age, gender, and geographic information, and educational level, ethnic and economic background and were free of a history of psychiatric illness based on GHQ-20 and SCL-90. Regular inter-rater reliability sessions and onsite check-ups were conducted to secure the long-term research quality. This study was approved by the institutional review boards of the participating hospitals, including that at UMC Utrecht. Written informed consent was obtained from all subjects after a complete description of the study and its procedures.

Sample collection: uwa1
Type: Case-Control
Contact: Schwab, Sibylle (schwab@uow.edu.au)
Description: Patients with schizophrenia admitted consecutively to psychiatric hospitals in the greater Jakarta area were informed about the study and were asked to sign the Informed Consent document for participation in interviews, blood withdrawal and subsequent genetic studies. The study was approved by the institutional review board of the University of Indonesia and by local Ethics Committees. Clinical consensus diagnosis of schizophrenia was made by

psychiatrists according to the DSM-IV criteria. Non-psychiatric controls were recruited from students and staff of the University of Indonesia, Jakarta, and from the participating hospitals. Details of the study were previously reported[1].

Sample collection: sxyh1
Type: Case-Control
Contact: Tang, Jinsong (tangjinsong@zju.edu.cn)
Description: Subjects were recruited from the Second Xiangya Hospital of Central South University, China. Patients were diagnosed by at least two experienced psychiatrists according to the DSM-IV based on structured Clinical Interview for DSM-IV Axis 1 Disorders (SCID-1). Healthy controls were selected from the general public of Chinese population in Changsha city, Hunan Province. The control subjects were also excluded with major mental illness and matched with patients in terms of age and gender. All the subjects were Han Chinese in origin. The study was approved by the Ethical Committee of the Second Xiangya Hospital of Central South University in Changsha. All subjects or their guardians understood the procedure and gave written informed consent, according to the Declaration of Helsinki. Access to this sample requires approval from the Human Genetic Resources Administration, National Health Commission of the People's Republic of China. Upon obtaining the necessary approval, applicants may submit a research proposal and evidence of ethics approval to the contact author as above. Requests will be reviewed by the contact author, with decisions typically provided within two weeks. Reasonable requests accompanied by the required legal and regulatory approvals will generally be approved.

Sample collection: xajd and xju2
Type: Case-Control
Contact: Zhu, Feng (zhufeng0714@163.com) and Yang, Jian (yjiann@outlook.com)
Schizophrenia patients were recruited from the inpatients in nine clinical centers in Shan Xi Province, China, including 1) Department of Psychiatry of the First Affiliated Hospital of Xi'an Jiaotong University (TFAHXJTU) and Mental Health Centers of 2) Baoji City, 3) Xianyang City, 4) Weinan City, 5) Hanzhong City, 6) Huayin City, 7)Yulin City, 8)Yanan City, 9) Xunyang city. Healthy controls were recruited from native residents of communities and villages in the city where each clinical center located in. Two professional psychiatrists interviewed all participants and evaluated their mental status according to DSM-IV criteria using SCID and/or Mini-International Neuropsychiatric Interview. The present study was approved by the Medical Ethics Committee of TFAHXJTU. All subjects participated voluntarily and signed written informed consent prior to the enrollment. Access to this sample requires approval from the Human Genetic Resources Administration, National Health Commission of the People's Republic of China. Upon obtaining the necessary approval, applicants may submit a research proposal and evidence of ethics approval to the contact authors as above. Requests will be reviewed by the contact authors, with decisions typically provided within two weeks. Reasonable requests accompanied by the required legal and regulatory approvals will generally be approved.

Sample collection: tai1multiplex
Type: multiplex families
Contact: Tsuang, Ming (mtsuang@ucsd.edu) and Chen, Wei J. (wjchen@nhri.edu.tw)
Description: Multiplex families were recruited from the Taiwan Schizophrenia Linkage Study (TSLS) between 1998 to 2002 in Taiwan funded by the National Institute of Mental Health in the United States. Each family had co-affected sib-pairs with schizophrenia and had at least two living first-degree relatives. The affected sib-pair probands, who met the Diagnostic and Statistical Manual of Mental Disorders, Fourth Edition (DSM-IV) criteria for schizophrenia or schizoaffective disorder, depressed type, were identified from 78 psychiatric hospitals or health

centers throughout Taiwan during the study period. This study was approved by the Department of Health and Human Services office and the National Taiwan University Hospital's Internal Review Board of Human Studies. Written informed consent was obtained from all subjects after a complete description. Study details have been reported elsewhere (Hwu et al., 2005; Faraone et al., 2006; Chen, 2013). Some of the recruited families (333 families) were genotyped using the Illumina PsychChip array funded by the National Health Research Institutes in Taiwan. The rest of the families (224 families) were genotyped using the Illumina Infinium Global Screening Array-24 v.1.0 (GSA) BeadChip at the Broad Institute. Access to this sample requires the submission of a research proposal and evidence of ethics approval to the contact authors. Requests will be reviewed by the contact authors, with decisions typically provided within two weeks. Reasonable requests accompanied by the required regulatory approvals will generally be approved.

Sample collection: push2
Type: Case-controls
Contact: Yue, Weihua (puh60380@bjmu.edu.cn)
Description: The cases were from the Chinese Antipsychotics Pharmacogenomics Consortium (CAPOC), which was established in 2010 with members from five research centres (Peking University Sixth Hospital, West China Hospital of Sichuan University, the Second Xiangya Hospital of Central South University, Beijing Anding Hospital Affiliated to Capital Medical University, and Beijing HuiLongGuan Hospital). Individuals included in our study had a diagnosis of schizophrenia based on the Structured Clinical Interview of DSM-IV, were aged 18–45 years, were of Han Chinese ancestry, scored more than 60 on the Positive and Negative Syndrome Scale (PANSS) (and scored more than four on at least three positive items), were physically healthy with all laboratory parameters within normal limits, had a condition that could be treated with oral medication, and were able to provide informed consent. All study protocols were approved by each site's institutional ethics review boards, and written informed consent was obtained. All participants were asked to appoint a family member or close friend who was involved with the informed consent discussion and helped the patient with decision-making. Access to this sample requires approval from the Human Genetic Resources Administration, National Health Commission of the People's Republic of China. Upon obtaining the necessary approval, applicants may submit a research proposal and evidence of ethics approval to the contact authors as above. Requests will be reviewed by the contact authors, with decisions typically provided within two weeks. Reasonable requests accompanied by the required legal and regulatory approvals will generally be approved.

Rare CNV calling

Input preprocessing of CNV calling.

All of our samples were genotyped on the Illumina platform. Varying chip types were utilized for each sample. We generated rare CNV calls for each sample separately. The calling pipeline started from 'idat' files, which contain the signal intensity of each probe. We downloaded all supporting files (including the manifest and cluster files) from Illumina's official website. We used the 'iaap-cli' tool (https://github.com/freeseek/gtc2vcf) to transform 'idat' files to 'gtc' files, the binary form of signal intensity files. Then, we used the IlluminaGenomeStudio package to convert the 'gtc' files to Final Reports. The exported final TXT files contained the following information: sample ID, SNP name, chr, position, allele1-forward, allele2-forward, normalized X, normalized Y, B allele frequency and log R ratio. We also called genotypes from 'gtc' files by the 'gtc2vcf' tool (https://github.com/freeseek/gtc2vcf). Genotypes were used to estimate PC1-5 of each sample. We did quality controls with genotypes, filtering out the subjects with high missing

rates and relatedness. Further details are discussed in the subsequent section "Quality control of genotype data".

Composite CNV calling pipeline.
The composite pipeline comprises CNV callers PennCNV[2] and iPattern[3]. PennCNV uses a Hidden Markov Model (HMM) to segment the genome into regions with similar signal intensity values and to infer the copy number state of each segment. It also incorporates population-level data and pedigree information to improve CNV calling accuracy. iPattern, on the other hand, implements a non-parametric density-based clustering model that integrates intensity data across subjects to assign individual subjects to distinct copy number states. Specifically, each analysis batch of iPattern contained approximately 200 samples and included a minimum of 50 individuals of each sex to ensure balanced representation.

Quality control of samples.
Sample QC was conducted based on the CNV and genotype matrix (see Quality control of genotype data seesion). We applied filtering criteria for removing subjects with (i) excessive probe variance or GC bias; (ii) a mismatch between self-reported and genetically inferred gender or ethnicity; and (iii) chromosomal aneuploidies. For each subject, we extracted a) LRRSD, b) BAFSD, and c) GCWF (waviness) from PennCNV log files and calculated the CNV proportion and CNV number for each subject. We excluded subjects within each sample where any of the extracted metrics was greater than 3 SD away from the median (within 3 SD from the median of GCWF). We did this within each sample instead of across all samples.

Quality control of CNV callset.
We combined PennCNV and iPattern results which leveraged complementary algorithms to improve CNV calling accuracy and reduce the number of false positives. CNV calls that were made by only one of the algorithms were excluded. Calls discordant for type of CNV (deletions or duplications) were also excluded. CNV calls that were detected by only one platform were excluded. Final CNV calls that were a) longer than 20 kb; b) supported by at least 15 probes; c) frequency less than 1% across the combined sample set; d) detected in at least two samples; e) detected by both CNV calling algorithms.

Quality control of genotype data.
Genotype quality control procedures were carried out on RICOPILI[4] using default parameters for each sample. Subjects with a pi_het value exceeding 0.9 were excluded, which indicated potential duplications or identical twins. Genomic imputation across all samples was carried out via the TOPMed server[5]. Variants with INFO score < 0.6 were excluded. Pre-imputation SNP filtration was conducted using the following criteria: 1) A minimum allele frequency of 0.05; 2) A genotype missingness threshold of 0.02; 3) A Hardy-Weinberg equilibrium p-value cutoff of 1e-10; 4) The exclusion of all but ACTG SNPs; 5) The removal of long range LD regions including MHC and chromosome 8 inversion; 6) Removal of all strand ambiguous SNPs.

Principal component analysis and kinship matrix estimation.
Principal Component Analysis (PCA) was carried out utilizing efficientPCA [https://github.com/hailianghuang/efficientPCA]. We first generated the genetic relationship matrix using the controls. Then, we calculated the PCs and the loadings of SNPs, and projected each subject into the PC space. The estimation of pairwise pi_het values was accomplished through the command plink --genome gz full.

CNV annealing and aneuploidy.

Since CNV callers tend to break long CNVs into short segments, we annealed multiple fragmented calls into large CNVs if their gap was shorter than 20% of the total CNV length. We also calculated the proportion of each chromosome (excluding chrY) tagged as a copy number variable. We excluded subjects where >10% of the chromosome was a copy number variable as possible aneuploidies. To ensure each CNV has sufficient SNPs to support, we filtered CNVs with probe densities less than 0.05 kb. Further, we excluded CNVs with 50% overlap with specific regions, including a) centromere and telomere, b) segmental duplication, c) immunoglobulin, and d) T-cell receptors.

Number of probes as the filter

We assessed the quality of CNV calling by applying filtering criteria that required each CNV to be supported by at least 15 or 10 probes. The 15-probe pipeline called fewer CNVs than the 10-probe pipeline (4% and 1% fewer for deletions and duplications, respectively). CNVs only called using the 10-probe pipeline are less likely to be replicated by the WES data: the percentage replicated by the WES was 26% and 54% for deletions called using 10-14 probes and 15+ probes, respectively; and 45% and 61% for duplications using 10- 14 probes and 15+ probes. Therefore, 15 probes was chosen as the threshold (Supplementary Figure 5).

Impact of probe thresholds on the difference between EAS and EUR CNV callsets

CNV length

Increasing the thresholds from 10 to 15 probes slightly increases the median CNV length. The median CNV length for the EAS subjects is 92.50 KB using a 15-probe threshold, compared to 91.86 KB with a 10-probe threshold. Since our conclusion is that EUR subjects have a longer median CNV length (227.01 KB in EUR vs. 92.50 KB in EAS, P = 2.2e−16), a slight increase in the EAS median, driven by the probe threshold, does not alter this conclusion; it only slightly reduces our power to detect the difference.

Count of rare CNV per Carrier

Increasing the probe threshold from 10 to 15 slightly reduces the average number of rare CNVs per carrier. Among EAS subjects, this number decreases from 1.38 with the 10-probe threshold to 1.32 with the 15-probe threshold—a modest difference of 0.06. In contrast, the difference between ancestries is substantially larger: using the 10-probe threshold, the average rare CNV count per carrier is 2.41 in EUR compared to 1.38 in EAS, a difference of 1.03. Thus, our conclusion that EUR individuals carry rarer CNVs than EAS individuals remains robust, even if the threshold introduces a slight downward bias in the estimates for EAS.

Rare CNV burden by genotyping platforms

To explore platform differences, we grouped the samples by array types and conducted heterogeneity tests across platforms or samples. We found no significant heterogeneity across the five platforms for the three burden metrics (P_{length} = 0.16, P_{count} = 0.04, P_{gene} = 0.66, Cochran's Q) nor the 15 samples (P_{length} = 0.21, P_{count} = 0.15, P_{gene} = 0.86, Cochran's Q). Rare CNV burden results for individual platforms and samples are provided in Supplementary Tables 4-5 and Extended Data Figures 5. We noted that the effect size of the number of genes impacted by rare CNVs was consistent across all genotyping platforms and samples.

Impact of LRR-SD

To assess the potential impact of LRR-SD on our findings, we performed logistic regression to evaluate the association between schizophrenia case-control status and LRR-SD, adjusting for sex, array type, and the top five principal components derived from genotypes. We did not observe a significant association between the disease status and LRR-SD (OR per standard deviation increase in LRR-SD = 0.55, 95% CI: [0.30, 1.01], P = 0.054). In addition, LRR-SD only

explained 0.0083% of the variance in disease case-control status (Cox and Snell pseudo-R^2 from logistic). We also found that, among EAS individuals, adjusting for LRR-SD had a negligible impact on the association between case-control status and genome-wide rare CNV burden (Supplementary Figure 2). Taken together, these results suggest that LRR-SD is unlikely to have a meaningful impact on our conclusion.

WES validation of array-based CNV calls

We have access to biospecimens from 18,335 subjects, including 54 individuals who are "carriers" of 4 of the 8 "novel" rare CNVs. These include 18 carriers for 15q11.2, 14 for 15q12, 3 for 15q13.3, and 15 for 1q21.2. We do not have access to biospecimens from any carriers of the remaining 4 rare CNVs: 8p21.3, 11q13.1, 19p13.3, and 19q13.42 —and thus were unable to perform validation for those loci.

Of the 54 carriers, 51 underwent WES and could be validated using this dataset. We performed CNV calling using GATK-gCNV on the WES data (Methods). The resulting WES-based CNV calls confirmed all CNVs in these 51 carriers at the four loci (Supplementary data 2 and Supplementary Table 17b).

For the 3 carriers who did not have WES data, we validated their CNVs—at loci 15q11.2, 15q12, and 1q21.2—using ddPCR. In addition, we included 7 carriers with WES-confirmed CNVs at the same loci as positive controls (3 for 15q11.2, 2 for 15q12, and 2 for 1q21.2) and 3 non-carriers as negative controls. All CNVs were confirmed by ddPCR: the 3 test samples and 7 positive controls showed the expected copy number changes, while the 3 negative controls showed no change in the copy number.

We also evaluated the overall exome validation using WES data. For rare CNV with more than one exon overlapped, WES validated 81% of deletions and 80% of duplications CNV called from array data in 16,623 subjects (Supplementary Table 17a and Supplementary Figure 5). This number aligns with previous reports and confirms the quality of our CNV calling from arrays[6–8].

Novel risk rare CNV genes of interest

Here, we provide a brief review of literature on exome-wide significant rare CNV genes identified in this study. Considering our gene-level association analyses tested 18,531 consensus coding sequence (CCDS) genes across two CNV types (deletions and duplications), we set a Bonferroni-corrected P-value threshold of 1.3e-6 as exome-wide significance. Six novel risk rare CNV genes reached this significance threshold:

NUDT18 (Nudix Hydrolase 18): This gene encodes a protein belonging to the Nudix hydrolase family. Notably expressed in the nervous system, *NUDT18* has been implicated in multisite chronic pain, according to past research[9]. Its specific role in neural pathways, however, warrants further exploration.

FAM160B2 (FHF Complex Subunit HOOK Interacting Protein 2B, also called RAI16): Identified as a facilitator of the MAPK/ERK and TGFB signaling pathways, *FAM160B2* is recognized for its potential influence on cell proliferation[10]. It is expressed within the nervous system and has been previously highlighted in studies examining psychiatric and substance use comorbidity[11], suggesting a broader role in mental health.

TMEM259 (Transmembrane Protein 259): This gene is implicated in regulating the endoplasmic reticulum ER-associated degradation pathway and managing endoplasmic reticulum stress

responses, vital functions within cellular health. Its expression profile in the nervous system underscores its potential significance in neurological homeostasis.

GRIN3B (Glutamate Ionotropic Receptor NMDA Type Subunit 3B): Central to motor neuron function, Grin3b partners with grin1, forming a receptor that facilitates excitatory glycine responses. Its expression within the nervous system and links to schizophrenia points to its role in psychiatric health, with variations in *GRIN3B* potentially contributing to the complexity of schizophrenia's genetic landscape[12].

DMTN (Dematin Actin Binding Protein) is a Protein Coding gene. Gene Ontology (GO) annotations related to this gene include signaling receptor binding and protein self-association.

FGF17 (Fibroblast Growth Factor 17) encodes a member of the fibroblast growth factor (FGF) family. Members of the FGF family possess broad mitogenic and cell survival activities and are involved in a variety of biological processes including embryonic development, cell growth, morphogenesis, tissue repair, tumor growth and invasion. This protein is expressed during embryogenesis and in the adult cerebellum and cortex and may be essential for vascular growth and normal brain development.

REFERENCES

1. Lam, M. *et al.* Comparative genetic architectures of schizophrenia in East Asian and European populations. *Nat. Genet.* **51**, 1670–1678 (2019).
2. Wang, K. *et al.* PennCNV: an integrated hidden Markov model designed for high-resolution copy number variation detection in whole-genome SNP genotyping data. *Genome Res.* **17**, 1665–1674 (2007).
3. Pinto, D. *et al.* Comprehensive assessment of array-based platforms and calling algorithms for detection of copy number variants. *Nat. Biotechnol.* **29**, 512–520 (2011).
4. Lam, M. *et al.* RICOPILI: Rapid Imputation for COnsortias PIpeLIne. *Bioinforma. Oxf. Engl.* **36**, 930–933 (2020).
5. Taliun, D. *et al.* Sequencing of 53,831 diverse genomes from the NHLBI TOPMed Program. *Nature* **590**, 290–299 (2021).
6. Hujoel, M. L. A. *et al.* Influences of rare copy-number variation on human complex traits. *Cell* **185**, 4233-4248.e27 (2022).
7. Retterer, K. *et al.* Assessing copy number from exome sequencing and exome array CGH based on CNV spectrum in a large clinical cohort. *Genet. Med. Off. J. Am. Coll. Med. Genet.* **17**, 623–629 (2015).
8. Tsuchida, N. *et al.* Detection of copy number variations in epilepsy using exome data. *Clin. Genet.* **93**, 577–587 (2018).
9. Johnston, K. J. A., Cote, A. C., Hicks, E., Johnson, J. & Huckins, L. M. Genetically Regulated Gene Expression in the Brain Associated With Chronic Pain: Relationships With Clinical Traits and Potential for Drug Repurposing. *Biol. Psychiatry* S0006-3223(23)01554–8 (2023) doi:10.1016/j.biopsych.2023.08.023.
10. Wang, W. *et al.* Retinoic acid induced 16 enhances tumorigenesis and serves as a novel tumor marker for hepatocellular carcinoma. *Carcinogenesis* **33**, 2578–2585 (2012).

11. Martínez-Magaña, J. J. *et al.* Genome-wide association study of psychiatric and substance use comorbidity in Mexican individuals. *Sci. Rep.* **11**, 6771 (2021).

12. Tarabeux, J. *et al.* Rare mutations in N-methyl-D-aspartate glutamate receptors in autism spectrum disorders and schizophrenia. *Transl. Psychiatry* **1**, e55 (2011).

Supplementary Figures

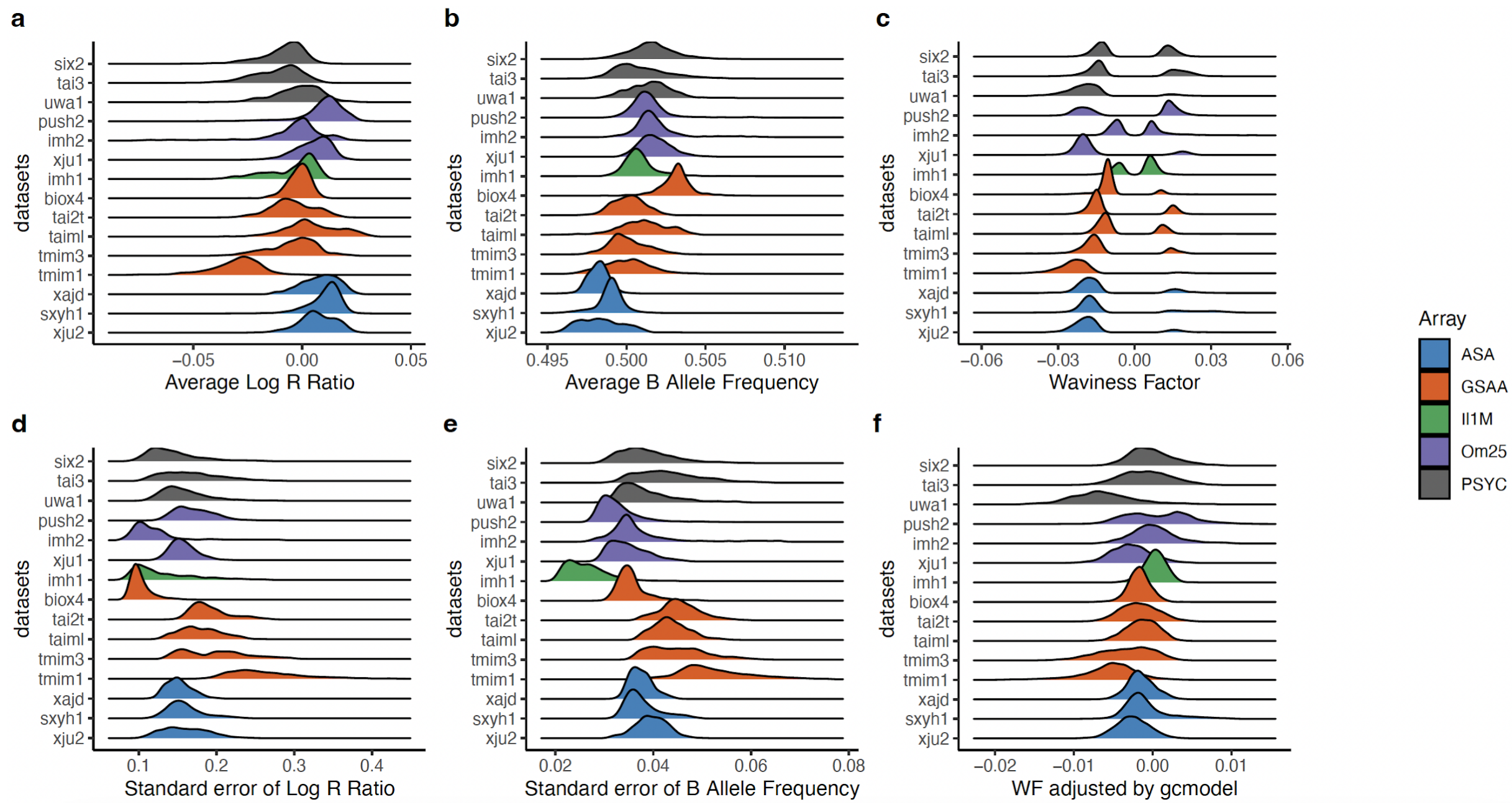

Supplementary Figure 1. The distribution of sample QC metrics across EAS samples. Density plot for **a,** average Log R Ratio; **b,** average B allele Frequency; **c,** waviness factor; **d,** standard error of Log R Ratio; **e,** standard error of B allele Frequency; **f,** waviness factor adjusted by gcmodel. Color represents the array types. ASA: Infinium Asian Screening Array; GSAA: Infinium Global Screening Array; Il1M: Human1M-Duo; PSYC, Infinium PsychArray; Om25, Infinium OmniZhongHua.

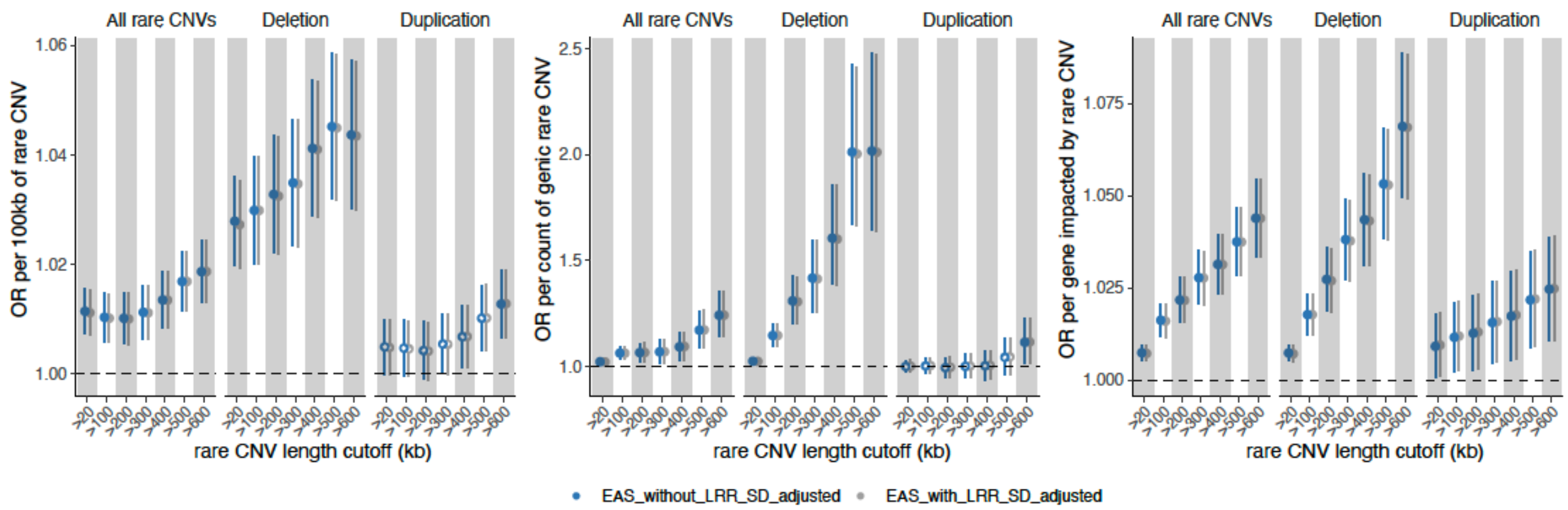

<u>Supplementary Figure 2: Impact of LRR_SD on rare CNV burden test.</u>

Odds ratios (ORs) for schizophrenia were estimated using regression models of genome-wide rare CNV burden in the East Asian (EAS) cohort, with and without adjustment for LRR_SD. Analyses were performed across multiple rare CNV length cutoffs (x axis) and stratified by all rare CNVs, deletions and duplications. ORs were estimated using mixed-effects logistic regression from 44,161 individuals. The left panel shows the OR per 100 kb increase in rare CNV length, the middle panel shows the OR per unit increase in genic rare CNV count, and the right panel shows the OR per gene impacted by rare CNV. Blue denotes models without LRR_SD adjustment and gray denotes models with LRR_SD adjustment. Points indicate OR estimates and error bars represent 95% confidence intervals. The dashed line marks the null expectation (OR = 1).

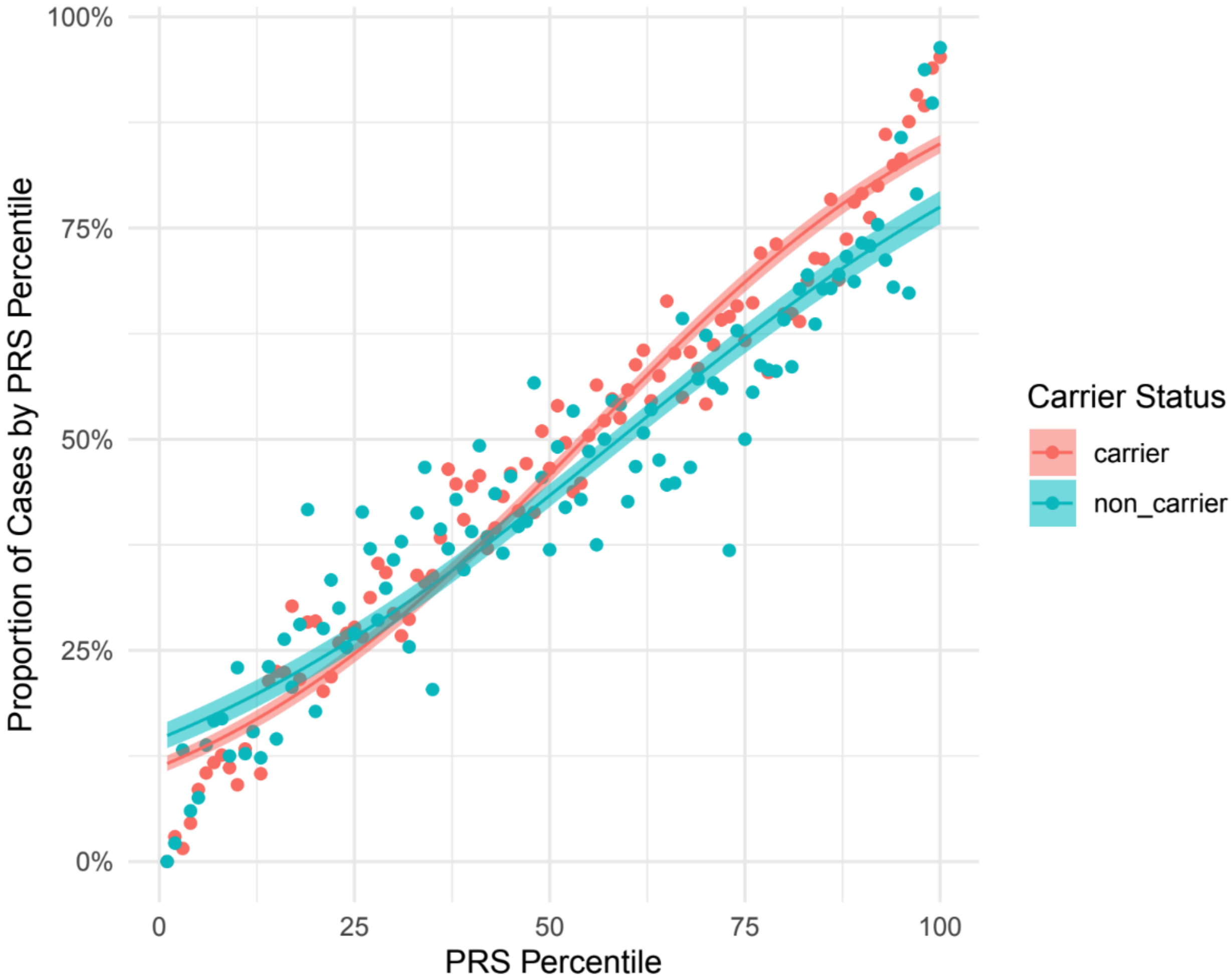

Supplementary Figure 3: The proportion of schizophrenia cases across PRS percentiles, stratified by rare CNV carrier status.

The proportion of schizophrenia cases is plotted across PRS percentiles for rare CNV carriers and non-carriers. Lines represent fitted values from logistic regression models based on the observed data points, and shaded areas denote 95% confidence intervals. The x axis indicates PRS percentile and the y axis indicates the proportion of schizophrenia cases within each percentile group.

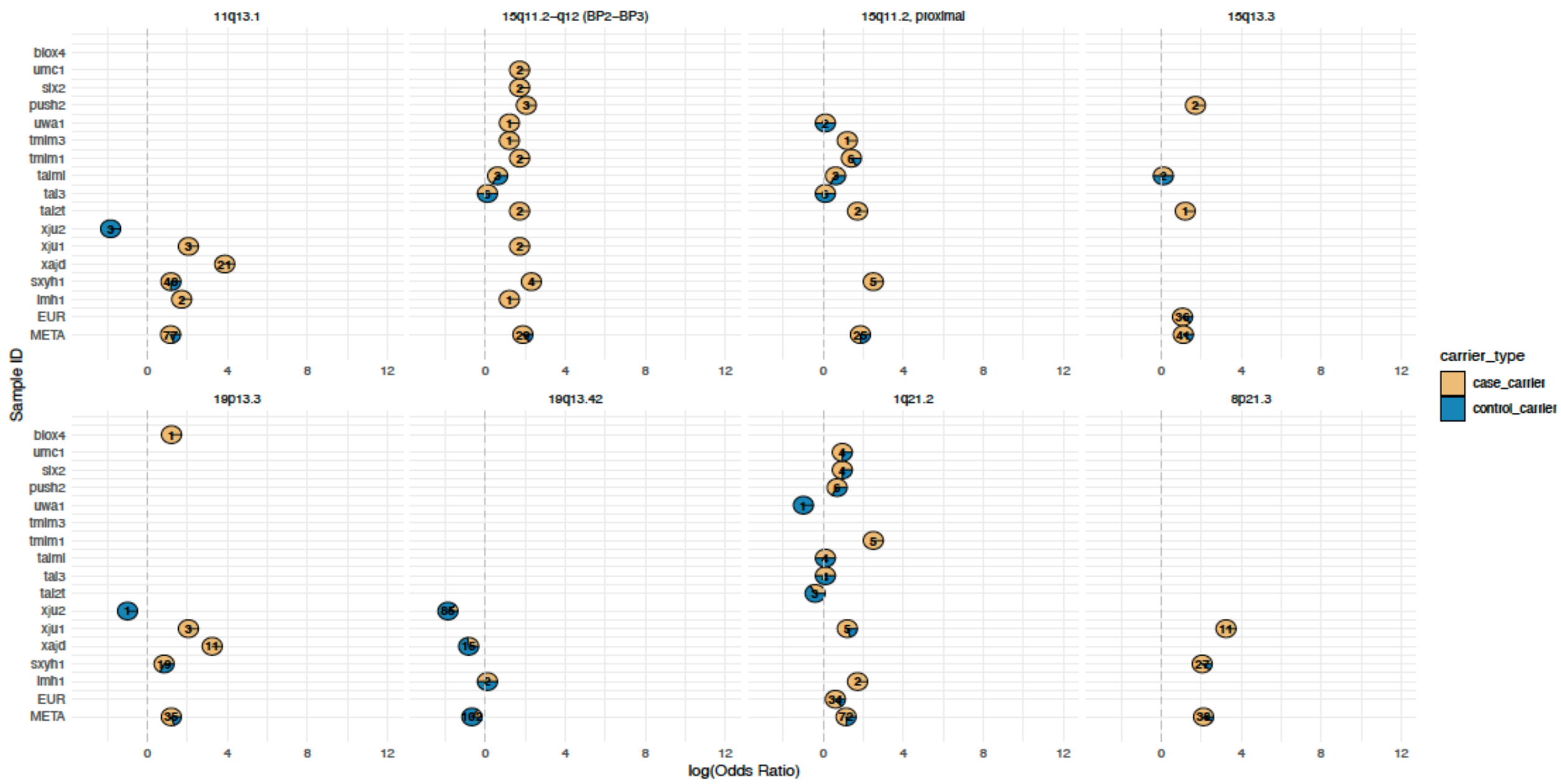

Supplementary Figure 4: Locus-wide rare CNV burden stratified by samples.
The x-axis represents the odds ratio (OR) on a logarithmic scale, and the y-axis lists the sample IDs. The vertical dashed line indicates an OR of 1. Each OR estimate is displayed as a pie chart, with the proportion of case carriers (orange) and control carriers (blue) represented within each sample shown. The number inside each pie chart indicates the total number of carriers in that sample.

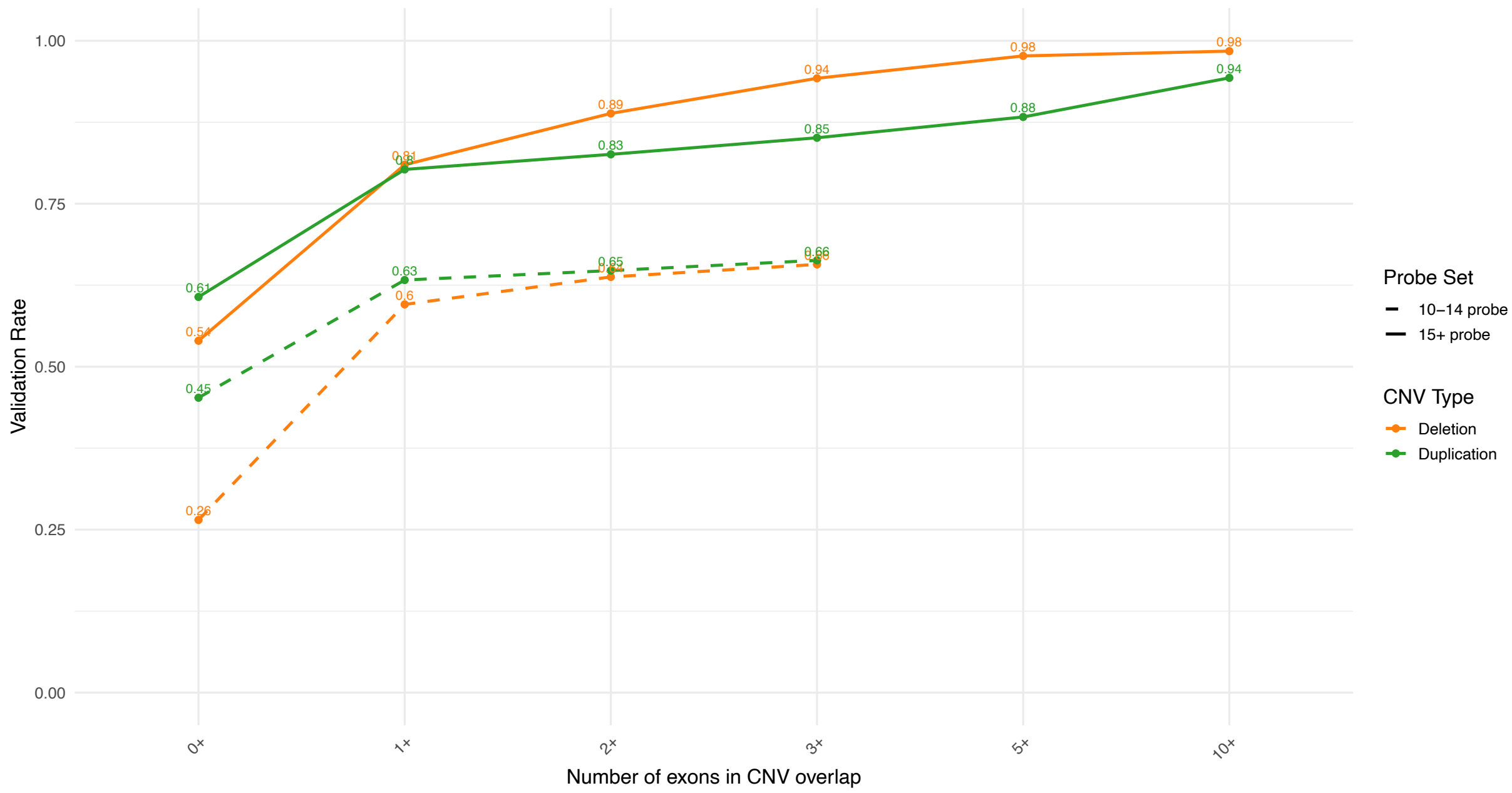

Supplementary Figure 5: Validation of WES-derived CNV calls partitioned by the number of overlapping exons.

Validation rates (y-axis) are showed separately for deletions (orange lines) and duplications (green lines), stratified by the number of exons spanned by each CNV (x-axis, categorized as 0, 1, 2, 3, 5, or 10+). Results are shown for both 10-14probe (dashed lines) and 15+probe (solid lines) thresholds.

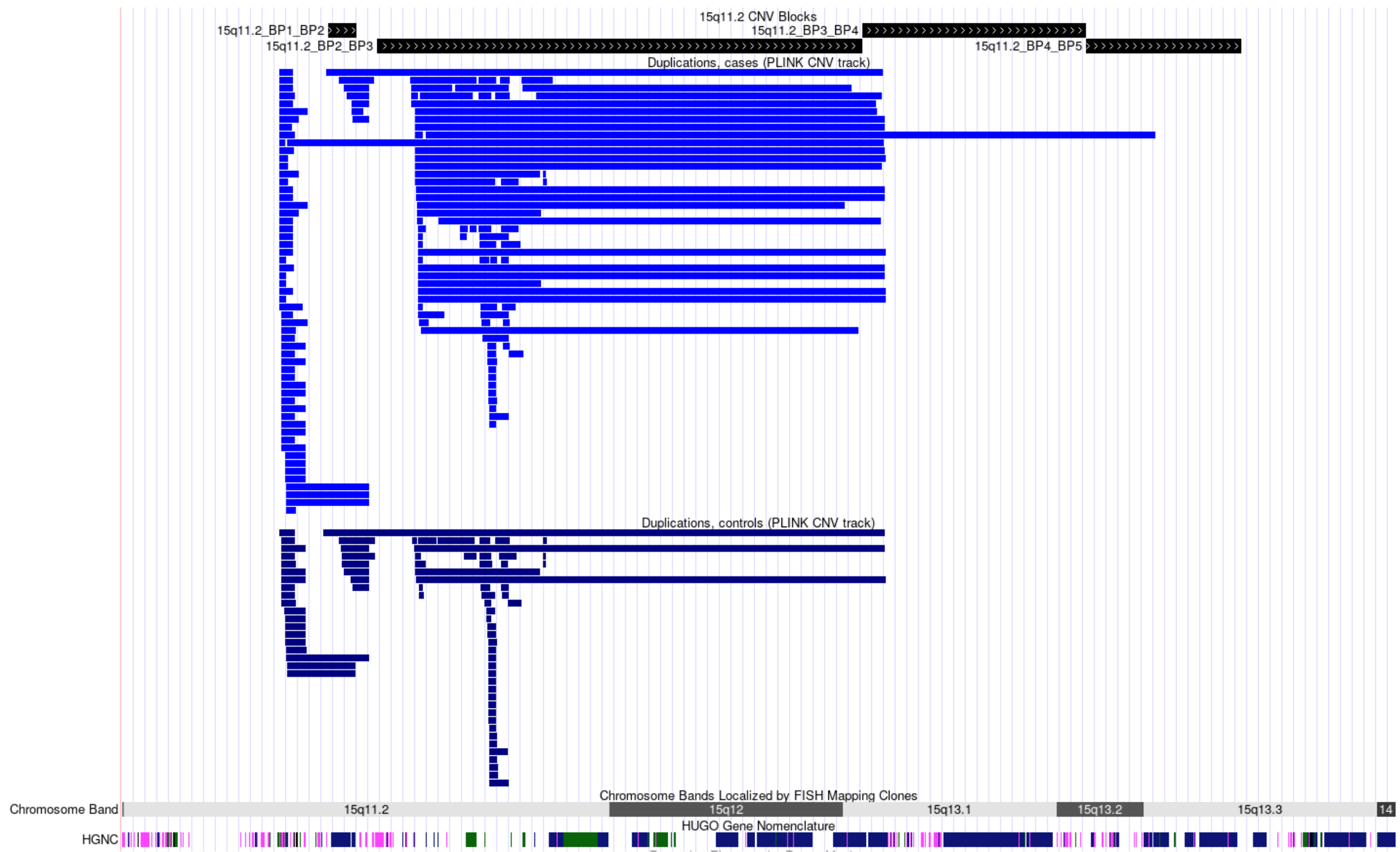

Supplementary Figure 6. Risk CNV duplications at chromosome 15q.

CNV tracks display duplications detected in cases and controls from the EAS samples. 15q11.2BP2-BP3 is part of the PWS/AS region that was previously reported as associated with schizophrenia.

Supplementary Data

Supplementary Data 1 and 2 are provided as separate pdf files.

Supplementary Data 1: Signal intensity for 8p21.3 (pages 1-3), 19q13.42 (pages 4-9), 1q21.2 (pages 10 and 11), 11q13.1 (pages 12-16), 15q11.2 (pages 17 and 18), 15q12 (pages 19 and 20), 15q13.3 (page 21), 19p13.3 (pages 22 and 23). Log R ratio (LRR) values are shown for CNV carriers across a region defined as the novel CNV locus ± 5 × its length. Each dot represents the LRR at an individual probe, with the x-axis indicating genomic position and the y-axis showing the LRR. Red points highlight probes within the novel CNV locus.

Supplementary Data 2: Read depth profiles of subjects for all WES-validated CNVs. Both panels show the normalized copy number (y-axis) against genome position (x-axis, left panel) and exome position (x-axis, right panel). We merged CNV on the same chromosome for the single subject. The grey shading represents the distribution of underlying data points from reference subject, typically centered around the diploid state (copy number = 2). Colored line segments show the estimated copy number state across each segment, with a solid dot indicating the mean copy number. Blue segments indicate increased copy number (duplication), while red segments indicate decreased copy number (deletions).
