# Supplementary material for: Contribution of copy number variants to schizophrenia in East Asian populations
Source: Nat Genet. 2026 Sep 11;58(10):2514–24. doi: 10.1038/s41588-026-02732-6 (PMC13649597; doi:10.1038/s41588-026-02732-6)
Supplement: Supplementary file 2 [file 41588_2026_2732_MOESM2_ESM.pdf]

nature portfolio

Corresponding author(s): Jinsong Tang, Xiancang Ma, Wei J Chen, Shengying Qin, Weihua Yue, Tian Ge, Hailiang Huang

Last updated by author(s): Apr 10, 2026

Reporting Summary

Nature Portfolio wishes to improve the reproducibility of the work that we publish. This form provides structure for consistency and transparency in reporting. For further information on Nature Portfolio policies, see our Editorial Policies and the Editorial Policy Checklist.

Statistics

For all statistical analyses, confirm that the following items are present in the figure legend, table legend, main text, or Methods section.

n/a	Confirmed	
☐	☒	The exact sample size (*n*) for each experimental group/condition, given as a discrete number and unit of measurement
☐	☒	A statement on whether measurements were taken from distinct samples or whether the same sample was measured repeatedly
☐	☒	The statistical test(s) used AND whether they are one- or two-sided<br>*Only common tests should be described solely by name; describe more complex techniques in the Methods section.*
☐	☒	A description of all covariates tested
☐	☒	A description of any assumptions or corrections, such as tests of normality and adjustment for multiple comparisons
☐	☒	A full description of the statistical parameters including central tendency (e.g. means) or other basic estimates (e.g. regression coefficient) AND variation (e.g. standard deviation) or associated estimates of uncertainty (e.g. confidence intervals)
☐	☒	For null hypothesis testing, the test statistic (e.g. *F*, *t*, *r*) with confidence intervals, effect sizes, degrees of freedom and *P* value noted<br>*Give P values as exact values whenever suitable.*
☒	☐	For Bayesian analysis, information on the choice of priors and Markov chain Monte Carlo settings
☒	☐	For hierarchical and complex designs, identification of the appropriate level for tests and full reporting of outcomes
☐	☒	Estimates of effect sizes (e.g. Cohen's *d*, Pearson's *r*), indicating how they were calculated

Our web collection on statistics for biologists contains articles on many of the points above.

Software and code

Policy information about availability of computer code

Data collection	No software was used for data collection.

Data analysis

Computer code relating to this study includes:
PennCNV v1.0.5: https://penncnv.openbioinformatics.org/en/latest/
iPattern v0.582: https://www.tcag.ca/tools/index.html
RICOPILI v2019_Jun_25.001: https://sites.google.com/a/broadinstitute.org/ricopili
Plink-1.07-x86_64: https://zzz.bwh.harvard.edu/plink/
Bcftools v1.19: http://samtools.github.io/bcftools/bcftools.html
METAL v2011_03_25: https://csg.sph.umich.edu/abecasis/Metal/index.html
REGENIE v3.2.2: https://rgcgithub.github.io/regenie/
Lme4qtl v0.1.10: https://github.com/variani/lme4qtl
Custom scripts used in the production of the EAS CNV dataset are publicly available via GitHub: https://github.com/QidiFeng/cnv_calling_QC_association
Custom scripts used in the analysis and plots of the EAS CNV dataset are publicly available via GitHub: https://github.com/yu-1011/EAS_SCZ_rCNV_analysis
Analysis codes are also deposit at Zenodo: https://doi.org/10.5281/zenodo.19058548

For manuscripts utilizing custom algorithms or software that are central to the research but not yet described in published literature, software must be made available to editors and reviewers. We strongly encourage code deposition in a community repository (e.g. GitHub). See the Nature Portfolio guidelines for submitting code & software for further information.

Data

Policy information about availability of data

All manuscripts must include a data availability statement. This statement should provide the following information, where applicable:

- Accession codes, unique identifiers, or web links for publicly available datasets
- A description of any restrictions on data availability
- For clinical datasets or third party data, please ensure that the statement adheres to our policy

Subject-level data for EAS samples are available upon request. We have deposited the data to the best of our ability, given the patient consent and national laws and regulations. Requests will be reviewed and responded to within two weeks. Access to subject-level data from samples recruited within mainland China is subject to the policies and approvals from the Human Genetic Resource Administration, Ministry of Science and Technology of the People's Republic of China. The summary statistic from this study is available at https://doi.org/10.5281/zenodo.19057959

Research involving human participants, their data, or biological material

Policy information about studies with human participants or human data. See also policy information about sex, gender (identity/presentation), and sexual orientation and race, ethnicity and racism.

Reporting on sex and gender

biox4: 42.7% of subjects are male, 33.7% of cases are male, and 51.8% of controls are male.
push2: 47.1% of subjects are male, 51.4% of cases are male, and 42.9% of controls are male
imh1: 64.9% of subjects are male, 66.6% of cases are male, and 63.3% of controls are male.
imh2: 52.9% of subjects are male, 53.6% of cases are male, and 52.3% of controls are male.
tmim3: 44.1% of subjects are male, 52.1% of cases are male, and 40.7% of controls are male.
tai2t: 55.0% of subjects are male, 61.1% of cases are male, and 51.0% of controls are male.
taiml: 55.1% of subjects are male, 64.5% of cases are male, and 44.5% of controls are male.
tai3: 53.7% of subjects are male, 61.2% of cases are male, and 50.9% of controls are male.
tmim1: 45.1% of subjects are male, 56.0% of cases are male, and 34.7% of controls are male.
uwa1: 62.6% of subjects are male, 74.3% of cases are male, and 51.4% of controls are male.
xajd: 64.5% of subjects are male, 72.0% of cases are male, and 58.1% of controls are male.
xju1: 41.4% of subjects are male, 53.4% of cases are male, and 18.7% of controls are male.
xju2: 59.3% of subjects are male, 61.8% of cases are male, and 58.2% of controls are male.
six2: 46.6% of subjects are male, 48.4% of cases are male, and 44.6% of controls are male.
sxyh1: 50.0% of subjects are male, 52.1% of cases are male, and 46.3% of controls are male.

Reporting on race, ethnicity, or other socially relevant groupings

Data was obtained from subjects living in East Asia (Mainland China, Taiwan, Indonesia, Japan, Korea, Singapore), North America and Europe. All cases were clinically diagnosed to have schizophrenia. Controls were either unaffected healthy controls from the schizophrenia studies or population based controls from various public resources.

biox4: All subjects are from Mainland China involved in a Case-Control study on SCZ. 50% of subjects are SCZ cases.

xju1: All subjects are from Mainland China involved in a Case-Control study on SCZ. 65.4% of subjects are SCZ cases.

tmim1: All subjects are from Japan, involved in a Case-Control study on SCZ 49.1% of subjects are SCZ cases.

tmim3: All subjects are from Japan, involved in a Case-Control study on SCZ 32.4% of subjects are SCZ cases.

tai1multiplex: All subjects are from Taiwan, involved in a Multiplex family study on SCZ. 53.4% of subjects are SCZ cases.

tai3: All subjects are from Taiwan, involved in a Trios study on SCZ. 34.5% of subjects are SCZ cases.

tai2t: All subjects are from Taiwan, involved in a Trios study on SCZ. 39.5% of subjects are SCZ cases.

six1: All subjects are from Mainland China, involved in a Case-Control study on SCZ. 77.4% of subjects are SCZ cases.

uwa1: All subjects are from Indonesia, involved in a Case-Control study on SCZ. 48.8% of subjects are SCZ cases.

xju2: All subjects are from Mainland China, involved in a Case-Control study on SCZ. 29.2% of subjects are SCZ cases.

xajd: All subjects are from Mainland China, involved in a Case-Control study on SCZ 49.7% of subjects are SCZ cases.

sxyh1: All subjects are from Mainland China, involved in a Case-Control study on SCZ. 66.4% of subjects are SCZ cases.

imh1: All subjects are from Singapore, involved in a Case-Control study on SCZ. 52.4% of subjects are SCZ cases.

imh2: All subjects are from Singapore, involved in a Case-Control study on SCZ. 46.0% of subjects are SCZ cases.

umc1: All subjects are from Mainland China, involved in a Case-Control study on SCZ.. 48.6% of subjects are SCZ cases.

push2: All subjects are from Mainland China, involved in a Case-Control study on SCZ,. 48.1% of subjects are SCZ cases.

Samples from EUR PGC samples described in Marshall et al., Nature Genetics 2017

Population characteristics

see above

Recruitment

Sample collection: imh1 and imh2
Type: Case-Control
Description: Subjects were part of the Singapore Translational and Clinical Research in Psychosis (STCRP) Study and comprised of cases and healthy controls of Chinese ancestry. schizophrenia patients were recruited from the Institute of Mental Health, Singapore. Diagnosis of schizophrenia was made using the Structured Clinical Interview for DSM-IV, Research Version, Patient Edition (SCID-I/P) by trained raters. And healthy controls were screened using the DSM-IV, Research Version, Non-Patient Edition (SCID-N/P). All participants gave consent according to IRB/DSRB guidelines prior to participating.

Sample collection: biox4 and xju1
Type: Case-Control
Description: biox4: Subjects were recruited from the Shanghai Mental Health Center, Shanghai Jiao Tong University School of Medicine between August 2015 and August 2017. Patients were diagnosed by at least two experienced psychiatrists according to the DSM-IV based on structured Clinical Interview for DSM-IV Axis 1 Disorders (SCID-1). Healthy controls were selected from the general public of Chinese population in Shanghai. The control subjects were also excluded with major mental illness and matched with patients in terms of age and gender. All the subjects were Han Chinese in origin. The study was approved by the Ethical Committee of Human Genetic Resources in Shanghai. All subjects or their guardians understood the procedure and gave written informed consent, according to the Declaration of Helsinki. xju1 Schizophrenia patients were recruited from the inpatients in nine clinical centers in Shaan Xi Province, China, including 1) Department of Psychiatry of the First Affiliated Hospital of Xi'an Jiaotong University (TFAHXJTU) and Mental Health Centers of 2) Baoji City, 3) Xianyang City, 4) Weinan City, 5) Hanzhong City, 6) Huayin City, 7)Yulin City, 8)Yanan City. Healthy controls were recruited from native residents of communities and villages in the city where each clinical center is located. Two professional psychiatrists interviewed all participants and evaluated their mental status according to DSM-IV criteria using SCID and/or Mini-International Neuropsychiatric Interview. The present study was approved by the Medical Ethics Committee of TFAHXJTU. All subjects participated voluntarily and signed written informed consent prior to the enrollment.

Sample collection: tmim1 and tmim3
Type: Case-Control
Description: All the subjects were recruited from multiple university hospitals and local hospitals in Japan. Patients were diagnosed according to the Diagnostic and Statistical Manual of Mental Disorders, 4th Edition (DSM-IV), with consensus from at least two experienced psychiatrists. All patients agreed to participate in the study and provided written informed consent. The study was approved by the Institutional Review Boards of the Tokyo Metropolitan Institute of Medical Science and all affiliated institutions. DNA samples were genotyped on the Illumina Infinium Global Screening Array-24 v.1.0 (GSA) BeadChip at the Broad Institute, using standard reagents and HTS workflow procedures.

Sample collection: tai3 and tai2trios
Type: trios
Description: Schizophrenia probands and their first-degree relatives were recruited from Schizophrenia Trio Genomic Research in Taiwan (S-TOGET) funded by the National Institute of Mental Health in the United States. Schizophrenia patients were interviewed with the Diagnostic Interview for Genetic Studies (DIGS), which was designed specifically for family-genetic studies of schizophrenia and bipolar disorder. Research diagnostic assessment was made independently by two board-certified psychiatrists based on integrated clinical information of DIGS interview data and summary notes of clinical course, symptom manifestations, and social functioning derived from the records of the medical charts according to DSM-IV for schizophrenia. This study was approved by the institutional review boards of the participating hospitals. Written informed consent was obtained from all subjects after a complete description. Study details have been reported elsewhere (Rees et al., 2015; Wang et al., 2017; Wang et al., 2019; Daniel P. Howrigan et al., 2020). The recruited trios had been genotyped using the Illumina PsychChip array funded by PGC, the Stanley Center for Psychiatric Research, the Friedman Brain Institute, and the Icahn Institute for Genomics and Multiscale Biology at Mount Sinai (i.e., tai3 sample).

Sample collection: six2
Type: Case-Control
Description: Funded by the Division of Neuroscience of the University Medical Center in Utrecht (UMC, Utrecht), a collaborative genetic study was developed between UMC Utrecht and Peking University Institute of Mental Health (PKUIMH). The goal of the study was to identify susceptibility genes that contribute to the development of schizophrenia and related disorders. Subjects were recruited from thirteen major cities across mainland China. All patients were interviewed with Structured Clinical Interview for DSM-IV axis I Disorder (SCID–I) by extensively trained Chinese psychiatrists and researchers

involved in the study. All included patients fulfilled DSM-IV criteria for schizophrenia. The SCID interviews were tape-recorded and randomly checked by senior psychiatrists not involved in the recruitment of interviewing. Healthy controls were matched with patients in terms of age, gender, and geographic information, and educational level, ethnic and economic background and were free of a history of psychiatric illness based on GHQ-20 and SCL-90. Regular inter-rater reliability sessions and onsite check-ups were conducted to secure the long-term research quality. This study was approved by the institutional review boards of the participating hospitals, including that at UMC Utrecht. Written informed consent was obtained from all subjects after a complete description of the study and its procedures.

Sample collection: uwa1
Type: Case-Control
Description: Patients with schizophrenia admitted consecutively to psychiatric hospitals in the greater Jakarta area were informed about the study and were asked to sign the Informed Consent document for participation in interviews, blood withdrawal and subsequent genetic studies. The study was approved by the institutional review board of the University of Indonesia and by local Ethics Committees. Clinical consensus diagnosis of schizophrenia was made by psychiatrists according to the DSM-IV criteria. Non-psychiatric controls were recruited from students and staff of the University of Indonesia, Jakarta, and from the participating hospitals. Details of the study were previously reported1.

Sample collection: sxyh1
Type: Case-Control
Description: Subjects were recruited from the Second Xiangya Hospital of Central South University, China. Patients were diagnosed by at least two experienced psychiatrists according to the DSM-IV based on structured Clinical Interview for DSM-IV Axis 1 Disorders (SCID-1). Healthy controls were selected from the general public of Chinese population in Changsha city, Hunan Province. The control subjects were also excluded with major mental illness and matched with patients in terms of age and gender. All the subjects were Han Chinese in origin. The study was approved by the Ethical Committee of the Second Xiangya Hospital of Central South University in Changsha. All subjects or their guardians understood the procedure and gave written informed consent, according to the Declaration of Helsinki.

Sample collection: xajd and xju2
Type: Case-Control
Schizophrenia patients were recruited from the inpatients in nine clinical centers in Shan Xi Province, China, including 1) Department of Psychiatry of the First Affiliated Hospital of Xi'an Jiaotong University (TFAHXJTU) and Mental Health Centers of 2) Baoji City, 3) Xianyang City, 4) Weinan City, 5) Hanzhong City, 6) Huayin City, 7)Yulin City, 8)Yanan City, 9) Xunyang city. Healthy controls were recruited from native residents of communities and villages in the city where each clinical center located in. Two professional psychiatrists interviewed all participants and evaluated their mental status according to DSM-IV criteria using SCID and/or Mini-International Neuropsychiatric Interview. The present study was approved by the Medical Ethics Committee of TFAHXJTU. All subjects participated voluntarily and signed written informed consent prior to the enrollment.

Sample collection: tai1multiplex
Type: multiplex families
Description: Multiplex families were recruited from the Taiwan Schizophrenia Linkage Study (TSLS) between 1998 to 2002 in Taiwan funded by the National Institute of Mental Health in the United States. Each family had co-affected sib-pairs with schizophrenia and had at least two living first-degree relatives. The affected sib-pair probands, who met the Diagnostic and Statistical Manual of Mental Disorders, Fourth Edition (DSM-IV) criteria for schizophrenia or schizoaffective disorder, depressed type, were identified from 78 psychiatric hospitals
or health centers throughout Taiwan during the study period. This study was approved by the Department of Health and Human Services office and the National Taiwan University Hospital's Internal Review Board of Human Studies. Written informed consent was obtained from all subjects after a complete description. Study details have been reported elsewhere (Hwu et al., 2005; Faraone et al., 2006; Chen, 2013). Some of the recruited families (333 families) were genotyped using the Illumina PsychChip array funded by the National Health Research Institutes in Taiwan. The rest of the families (224 families) were genotyped using the Illumina Infinium Global Screening Array-24 v.1.0 (GSA) BeadChip at the Broad Institute funded by PGC.

Sample collection: push2
Type: Case-controls
Description: The cases were from the Chinese Antipsychotics Pharmacogenomics Consortium (CAPOC), which was established in 2010 with members from five research centres (Peking University Sixth Hospital, West China Hospital of Sichuan University, the Second Xiangya Hospital of Central South University, Beijing Anding Hospital Affiliated to Capital Medical University, and Beijing HuiLongGuan Hospital). Individuals included in our study had a diagnosis of schizophrenia based on the Structured Clinical Interview of DSM-IV, were aged 18–45 years, were of Han Chinese ancestry, scored more than 60 on the Positive and Negative Syndrome Scale (PANSS) (and scored more than four on at least three positive items), were physically healthy with all laboratory parameters within normal limits, had a condition that could be treated with oral medication, and were able to provide informed consent. All study protocols were approved by each site's institutional ethics review boards, and written informed consent was obtained. All participants were asked to appoint a family member or close friend who was involved with the informed consent discussion and helped the patient with decision-making.

Samples from EUR PGC Recruitment described in Marshall et al., Nature Genetics 2017

Ethics oversight

The following institutions provided ethics oversight: Samsung Medical Center; Bio-X Institutes of Shanghai Jiao Tong University; Xi'an Jiaotong University; The Second Xiangya Hospital of Central South University; Peking University Sixth Hospital; Tokyo Metropolitan Institute of Medical Science; University Medical Center Utrecht; The University of Western Australia; The University of Indonesia; Chonnam National University Hospital, and Mass General Brigham (Protocols 2014P001342 and 2011P002207). Informed consent and permission to share the data were obtained from all subjects, in compliance with the guidelines specified by the recruiting center's institutional review board.

Note that full information on the approval of the study protocol must also be provided in the manuscript.

Field-specific reporting

Please select the one below that is the best fit for your research. If you are not sure, read the appropriate sections before making your selection.

☒ Life sciences ☐ Behavioural & social sciences ☐ Ecological, evolutionary & environmental sciences

For a reference copy of the document with all sections, see nature.com/documents/nr-reporting-summary-flat.pdf

Life sciences study design

All studies must disclose on these points even when the disclosure is negative.

Sample size

A complete list of datasets included in the EAS analysis can be found in the Supplementary Note. The EUR dataset was shared by the PGC CNV work group. This analysis included samples across EAS and EUR ancestries from 38,409 cases and 40,009 controls. Within the EAS population, 44,161 individuals were retained, including 20,903 cases and 23,258 controls. Within the EUR population, 34,257 individuals were included, including 17,506 cases and 16,751 controls.

Data exclusions

Quality control of genotype data. Genotype quality control procedures were carried out on RICOPILI4 using default parameters for each sample. Samples with a pi_het value exceeding 0.9 were excluded, as they indicate potential duplications or identical twins. Genomic imputation across all samples was carried out via the TOPMed server. Variants > 0.6 were excluded. Pre-imputation SNP filtration is conducted under the stringent guidelines of six conditions: 1) A minimum allele frequency of 0.05; 2) A genotype missingness threshold of 0.02; 3) A Hardy-Weinberg equilibrium p-value cutoff of 1e-10; 4) The exclusion of all but ACTG SNPs; 5) The removal of specified regions including MHC & chr8inv; 6) Removal of all ambiguous SNPs.Quality control of genotype data. Genotype quality control procedures were carried out on RICOPILI using default parameters for each sample. Samples with a pi_het value exceeding 0.9 were excluded, as they indicate potential duplications or identical twins. Genomic imputation across all samples was carried out via the TOPMed server5. Variants > 0.6 were excluded. Pre-imputation SNP filtration is conducted under the stringent guidelines of six conditions: 1) A minimum allele frequency of 0.05; 2) A genotype missingness threshold of 0.02; 3) A Hardy-Weinberg equilibrium p-value cutoff of 1e-10; 4) The exclusion of all but ACTG SNPs; 5) The removal of specified regions including MHC & chr8inv; 6) Removal of all ambiguous SNPs.

CNV Sample QC. Sample QC was conducted based on the CNV and genotype matrix. Following merging, we applied filtering criteria for removing samples with excessive probe variance or GC bias and removing samples with a mismatch in gender or ethnicity or chromosomal aneuploidies. For each sample, we extracted a) LRRSD, b) BAFSD, and c) GCWF (waviness) from PennCNV log files and calculated the CNV proportion and CNV number for each sample. We excluded samples within each sample where any of the extracted metrics is greater than 3 SD of the median (within 3 SD of the median for GCWF). We did this within each sample instead of across all samples.

CNV callset QC. Combining PennCNV and iPattern leverages complementary algorithms to call CNVs from different genotyping platforms. A standard approach is combining their results to improve CNV calling accuracy and reduce the number of false positives. CNV calls that were made by only one of the algorithms were excluded. Calls discordant for type of CNV (deletions or duplications) were also excluded.

CNV annealing and aneuploidy. Since CNV callers tend to break long CNVs into short segments, we anneal those fragmented multiple calls into large CNVs if their gap is less than 20% of the total length. We also calculated the proportion of each chromosome (excluding chrY) tagged as a copy number variable. We excluded samples where >10% of the chromosome was a copy number variable as possible aneuploidies. To make sure each CNV has sufficient SNPs to support, we filtered CNVs with probe densities less than 0.05 kb. Further, we excluded CNVs with 50% overlap with specific regions, including a) Centromere and telomere, b) segmental duplication, c) immunoglobulin, and d) T-cell receptors.

WES CNV QC: Stringent quality control measures were applied on whole exome sequencing calling data, where we removed samples that had too many calls (>200 raw autosomal calls or >35 calls with QS score > 20), variants that did not achieve a sufficient score as recommended by GATK-gCNV (a variable threshold based on size of the event), variants that did not span three or more exons and intervals, and variants that were called in more than 1% of the cohort.

Replication

The analysis was performed on independent samples that serve the purpose of replication with each other.

Randomization

No randomization was conducted. The current study is a large-scale genetics study. Randomness is achieved from the nature of how alleles are distributed in the population. Alleles are randomly passed from parent to offspring during meiosis, hence the nature of these types of studies tend to be shielded from extraneous confounds of standard epidemiological studies. One exception of this is the population structure, which can confound the random passing of genetic alleles. To resolve the problem, we performed the principal component (PC) analysis to capture the population structure as PCs. We then included PCs in analysis as covariates to control the confounding driven by the population structure.

Blinding

The nature of the study is in of itself blind to study recruiters. No one would beforehand know the genotype make up of the cases and controls recruited in the current study.

Reporting for specific materials, systems and methods

We require information from authors about some types of materials, experimental systems and methods used in many studies. Here, indicate whether each material, system or method listed is relevant to your study. If you are not sure if a list item applies to your research, read the appropriate section before selecting a response.

Materials & experimental systems

n/a	Involved in the study
☒	☐ Antibodies
☒	☐ Eukaryotic cell lines
☒	☐ Palaeontology and archaeology
☒	☐ Animals and other organisms
☒	☐ Clinical data
☒	☐ Dual use research of concern
☒	☐ Plants

Methods

n/a	Involved in the study
☒	☐ ChIP-seq
☒	☐ Flow cytometry
☒	☐ MRI-based neuroimaging

Plants

Seed stocks

Report on the source of all seed stocks or other plant material used. If applicable, state the seed stock centre and catalogue number. If plant specimens were collected from the field, describe the collection location, date and sampling procedures.

Novel plant genotypes

Describe the methods by which all novel plant genotypes were produced. This includes those generated by transgenic approaches, gene editing, chemical/radiation-based mutagenesis and hybridization. For transgenic lines, describe the transformation method, the number of independent lines analyzed and the generation upon which experiments were performed. For gene-edited lines, describe the editor used, the endogenous sequence targeted for editing, the targeting guide RNA sequence (if applicable) and how the editor was applied.

Authentication

Describe any authentication procedures for each seed stock used or novel genotype generated. Describe any experiments used to assess the effect of a mutation and, where applicable, how potential secondary effects (e.g. second site T-DNA insertions, mosiacism, off-target gene editing) were examined.
