# Supplementary material for: Contribution of copy number variants to schizophrenia in East Asian populations
Source: Nat Genet. 2026 Sep 11;58(10):2514–24. doi: 10.1038/s41588-026-02732-6 (PMC13649597; doi:10.1038/s41588-026-02732-6)
Supplement: Supplementary file 3 [file 41588_2026_2732_MOESM3_ESM.pdf]

nature portfolio

Peer Review File

Contribution of copy number variants to schizophrenia in East Asian populations

Corresponding Author: Professor Hailiang Huang

This file contains all editorial decision letters in order by version, followed by all author rebuttals in order by version.

Version 0:

Decision Letter:

26th November 2024

Dear Hailiang,

Your Article "Contribution of copy number variants to schizophrenia in East Asian populations" has been seen by two referees. You will see from their comments below that, while they find your work of interest, they have raised several relevant points. We are interested in the possibility of publishing your study in Nature Genetics, but we would like to consider your response to these points in the form of a revised manuscript before we make a final decision on publication.

To guide the scope of the revisions, the editors discuss the referee reports in detail within the team, including with the chief editor, with a view to identifying key priorities that should be addressed in revision, and sometimes overruling referee requests that are deemed beyond the scope of the current study. In this case, we ask that you address all technical queries related to the CNV calling and association analyses, revising the presentation and interpretations where appropriate and extending the analyses and validation where feasible as requested by the referees. We hope you will find this prioritized set of referee points to be useful when revising your study. Please do not hesitate to get in touch if you would like to discuss these issues further.

We therefore invite you to revise your manuscript taking into account all reviewer and editor comments. Please highlight all changes in the manuscript text file. At this stage, we will need you to upload a copy of the manuscript in MS Word .docx or similar editable format.

We are committed to providing a fair and constructive peer-review process. Do not hesitate to contact us if there are specific requests from the reviewers that you believe are technically impossible or unlikely to yield a meaningful outcome.

When revising your manuscript:

*1) Include a “Response to referees” document detailing, point-by-point, how you addressed each referee comment. If no action was taken to address a point, you must provide a compelling argument. This response will be sent back to the referees along with the revised manuscript.

*2) If you have not done so already, please begin to revise your manuscript so that it conforms to our Article format instructions, available
here.
Refer also to any guidelines provided in this letter.

*3) Include a revised version of any required Reporting Summary: https://www.nature.com/documents/nr-reporting-summary.pdf
It will be available to referees (and, potentially, statisticians) to aid in their evaluation if the manuscript goes back for peer review.
A revised checklist is essential for re-review of the paper.

Please be aware of our guidelines on

digital image standards.

Please use the link below to submit your revised manuscript and related files:

Link Redacted

Note: This URL links to your confidential home page and associated information about manuscripts you may have submitted, or that you are reviewing for us. If you wish to forward this email to co-authors, please delete the link to your homepage.

We hope to receive your revised manuscript within 8-12 weeks. If you cannot send it within this time, please let us know.

Please do not hesitate to contact me if you have any questions or would like to discuss these revisions further.

Nature Genetics is committed to improving transparency in authorship. As part of our efforts in this direction, we are now requesting that all authors identified as 'corresponding author' on published papers create and link their Open Researcher and Contributor Identifier (ORCID) with their account on the Manuscript Tracking System (MTS), prior to acceptance. ORCID helps the scientific community achieve unambiguous attribution of all scholarly contributions. You can create and link your ORCID from the home page of the MTS by clicking on 'Modify my Springer Nature account'. For more information, please visit www.springernature.com/orcid.

We look forward to seeing the revised manuscript and thank you for the opportunity to review your work.

Sincerely,
Kyle

Kyle Vogan, PhD
Senior Editor
Nature Genetics
https://orcid.org/0000-0001-9565-9665

Referee expertise:

Referee #1: Genetics, structural variation, psychiatric diseases

Referee #2: Genetics, structural variation, psychiatric diseases

Reviewers' Comments:

Reviewer #1 (Remarks to the Author):

Chen et al performed the largest schizophrenia rCNV study of EAS ancestries to date followed by a meta-analysis with the largest EUR study previously published in NG. They identified 9 rCNV loci associated with schizophrenia in EAS, and 15 when meta-analyzed with EUR individuals.

1. This manuscript is submitted under the "Article" category, which is defined as a substantial novel research study with a complex story often involving several techniques or approaches. However, the novelty of this work mostly lies within the large sample size of EAS ancestries. Only standard analyses were carried out here using established methods. No new ideas or approaches were used.

2. The authors reported 8 rCNV loci for the first time, however, they did not carry out any confirmatory experimental validation of these CNVs. It may not be possible to verify every CNV carrier but at least a subset of samples would be warranted.

3. The authors stated that their CNV calling and QC pipeline were consistent with those used in the EUR study. However, the EUR study (ref 14) required CNVs to encompass at least 10 probes while this study 15 probes.

4. The authors carried out a consistency check by comparing CNVs of a subset of EUR individuals called using their pipeline vs those previously published. There are problems as to how the results were interpreted. For example, they found only 84% overlap but called this a "slight" difference and attributed this to differences in allele frequency calculation. However, item #3 above should be considered in interpreting the difference. Indeed, they found that the 16% rCNV not detected by their pipeline were smaller with fewer probes. Isn't that the obvious consequence given the different filters based on the number of probes spanned by a CNV?

5. In addition, the authors compared the average rCNV length and rCNV count per carrier between EAS and EUR and found both measures smaller in the EAS than in EUR samples. They attributed this observation to genotyping arrays optimized for EUR. Again, the difference in the probe number filter (item #3) should be considered here too.

6. Line 219 – The Wilcoxon signed-rank test is for paired samples. Wilcoxon rank-sum test for two independent samples is appropriate here.

7. The study consisted of multiple samples and a variety of genotyping platforms. It would be useful to present an evaluation/comparison on data quality metrics, for example, LRR-SD from the Illumina arrays.

8. Maybe I missed those plots somewhere, but providing probe intensity plots for some of those novel rCNVs identified would be helpful.

9. Supplementary Note line 185 – "iPattern, on other hand, also uses an HMM-based approach to call CNVs" - This is wrong. In addition, the authors should provide details on how they formed iPattern calling batches that includes multiple samples in each.

10. More analyses could be done to improve the manuscript and provide biological insights to the novel association loci. For example, gene-set (pathway) analysis, evaluation of the CNV formation mechanism (NAHR-mediated?), comparison of the relative contribution of rCNV burden and polygenic risk score from common variant associations.

Reviewer #2 (Remarks to the Author):

Contribution of copy number variants to schizophrenia in East Asian populations by Chen et al.

Testing reported copy number variants (CNVs) associated with schizophrenia in a substantial East Asian sample (20,903 cases and 23,258 controls) is both important and timely. Additionally, the authors investigate this sample for novel CNVs and identify associations with eight new variants.

However, several issues need to be addressed:

First, the authors introduce the term "rare copy number variants (rCNVs)." More conventional terms like "recurrent CNVs," "neuropsychiatric CNVs," or "schizophrenia CNVs" are typically used in the literature. Is there a reason to introduce rCNV – can't they simply be called rare CNVs and those that associate with schizophrenia called schizophrenia CNVs?

Secondly, the authors use conventional names for CNVs already associated with schizophrenia but do not adhere to the standard start and stop positions for these CNVs (e.g., the 15q11.2 is not referring to the typical 15q11.2 BP1-BP2 CNV). Additionally, the authors group the 16p11.2 distal and 16p11.2 proximal deletions together, which are usually tested separately. This creates confusion when data are presented under the same CNV name but with different genomic positions. This inconsistency needs to be corrected by using the conventional start and stop positions for the different CNVs. The manuscript would be much clearer if the approximately 25 previously reported schizophrenia-associated CNVs were tested in the East Asian sample and presented in this manuscript using the same definitions that have been standard in the literature for the last 20 years.

Also, authors have to be more specific, two examples below:

In the abstract you say:
"Genes significantly associated with schizophrenia in these rCNV loci tend to be intolerant to protein-truncating variants." Which CNVs are you referring to? The eight novel CNVs or CNVs that have already been associated with schizophrenia? What does tend mean in this context? – can you be more specific?

Also in the abstract you say:
"Many novel rCNV associations in this study had a much higher carrier frequency in EAS and EUR, underscoring the pressing need to expand the population diversity to capture the complete genetic risk factors underpinning schizophrenia." Here you should be more specific. What does "much higher" mean – readers may get the impression that you are referring to common variants?

For these relatively rare variants, the primary concerns revolve around the methodology used to call CNVs in this category. Additionally, it's important to ensure that cases and controls are well-matched in terms of age and gender. The study used different genotyping platforms, raising the question of whether the SNP content is comparable across the CHIPs used, including the number of SNPs. It is important to investigate if there are any batch effects seen for the eight novel CNVs identified. Another key point is to assess whether the frequency and odds ratios (OR) are consistent across all 15 East Asian (EAS) schizophrenia samples included in this study. A forest plot for these 15 East Asian samples is required to visualize this data.

Version 1:

Decision Letter:

12th June 2025

Dear Hailiang,

Your revised Article "Contribution of copy number variants to schizophrenia in East Asian populations" has been seen by Reviewer #2. (Reviewer #1 was also contacted but was unable to provide comments on the revision.) As you will see from the comments below, Reviewer #2 finds the study improved but has requested a few additional clarifications. We remain interested in the possibility of publishing your study in Nature Genetics, but we would like to see your response to these points in the form of a further revision before we make a final decision on publication.

We therefore invite you to further revise your manuscript taking into account these comments. Please highlight all changes in the manuscript text file. At this stage, we will need you to upload a copy of the manuscript in MS Word .docx or similar editable format.

We are committed to providing a fair and constructive peer-review process. Do not hesitate to contact us if there are specific requests from the reviewers that you believe are technically impossible or unlikely to yield a meaningful outcome.

When revising your manuscript:

*1) Include a “Response to referees” document detailing, point-by-point, how you addressed each referee comment. If no action was taken to address a point, you must provide a compelling argument. This response will be sent back to the referees along with the revised manuscript.

*2) If you have not done so already, please begin to revise your manuscript so that it conforms to our Article format instructions, available
here.
Refer also to any guidelines provided in this letter.

*3) Include a revised version of any required Reporting Summary: https://www.nature.com/documents/nr-reporting-summary.pdf
It will be available to referees (and, potentially, statisticians) to aid in their evaluation if the manuscript goes back for peer review.
A revised checklist is essential for re-review of the paper.

Please be aware of our guidelines on digital image standards.

EXTENDED DATA FIGURES

When re-submitting your manuscript, please ensure that any supplementary figures and tables that are crucial to the manuscript’s conclusions are converted into Extended Data figures and tables to increase visibility of these data. Extended Data figures and tables are online-only (present in the online PDF and full-text HTML versions of the paper), peer-reviewed display items that provide essential background to the article but are not included in the main article due to space constraints. A maximum of ten Extended Data display items (figures and tables) is permitted.

Please use the link below to submit your revised manuscript and related files:

Link Redacted

Note: This URL links to your confidential home page and associated information about manuscripts you may have submitted, or that you are reviewing for us. If you wish to forward this email to co-authors, please delete the link to your homepage.

We hope to receive your revised manuscript within 4-8 weeks. If you cannot send it within this time, please let us know.

Please do not hesitate to contact me if you have any questions or would like to discuss these revisions further.

Nature Genetics is committed to improving transparency in authorship. As part of our efforts in this direction, we are now requesting that all authors identified as ‘corresponding author’ on published papers create and link their Open Researcher and Contributor Identifier (ORCID) with their account on the Manuscript Tracking System (MTS), prior to acceptance. ORCID helps the scientific community achieve unambiguous attribution of all scholarly contributions. You can create and link your ORCID from the home page of the MTS by clicking on ‘Modify my Springer Nature account’. For more information, please visit www.springernature.com/orcid.

We look forward to seeing the revised manuscript and thank you for the opportunity to review your work.

Sincerely,
Kyle

Kyle Vogan, PhD
Senior Editor
Nature Genetics
https://orcid.org/0000-0001-9565-9665

Referee expertise:

Referee #2: Genetics, structural variation, psychiatric diseases

Reviewers' Comments:

Reviewer #2 (Remarks to the Author):

The authors have addressed many of my previous comments and concerns. However, there remain a few points that require further clarification. I encourage the authors to more clearly specify and clarify the CNVs being studied for the interpretation of the findings.

As an example, a couple of questions and suggestions regarding Table 2 in your manuscript:

Top Rare CNV Gene: In Table 2, there is a column labeled "Top rare CNV gene". Below the table, you note that the OR, P-value, and top rare CNV gene correspond to the most significantly associated gene within each locus. Could you clarify how the top rare CNV gene is determined? Specifically, is this based on statistical significance from gene-level burden testing, or simply the most well-known or previously reported gene within the region?

References for CNV associations: It would be very helpful to include references in Table 2 to support the associations listed. For example, the 15q11.2 duplication is listed, but to my knowledge, there is no strong evidence for its association with schizophrenia. In fact, large cohorts such as the UK Biobank have reported no significant association between duplications at 15q11.2 (BP1-BP2 spanning NIPA1, NIPA2, CYFIP1, and TUBGCP5) and schizophrenia.

Could there be a mix-up with 15q11.2 duplications that extend beyond BP2, for example, to CHRNA7 (i.e., BP1–BP5), which are associated with schizophrenia? Is it possible that the CNV definition differs in the Asian cohorts—for example, if longer duplications spanning BP1–BP5 are grouped under “15q11.2 duplication”?

I think clarifying this distinction and providing references for each listed locus would improve the transparency and robustness of Table 2.

Version 2:

Decision Letter:

Our ref: NG-A65555R1

23rd December 2025

Dear Hailiang,

Thank you for submitting your revised manuscript "Contribution of copy number variants to schizophrenia in East Asian populations" (NG-A65555R1). In light of your revisions and clarifications in response to Reviewer #2, we will be happy in principle to publish your study in Nature Genetics as an Article pending final revisions to comply with our editorial and formatting guidelines.

We are now performing detailed checks on your paper, and we will send you a checklist detailing our editorial and formatting requirements soon. Please do not upload the final materials or make any revisions until you receive this additional information from us.

Thank you again for your interest in Nature Genetics. Please do not hesitate to contact me if you have any questions.

Sincerely,
Kyle

Kyle Vogan, PhD
Senior Editor
Nature Genetics
https://orcid.org/0000-0001-9565-9665

Open Access This Peer Review File is licensed under a Creative Commons Attribution 4.0 International License, which permits use, sharing, adaptation, distribution and reproduction in any medium or format, as long as you give appropriate credit to the original author(s) and the source, provide a link to the Creative Commons license, and indicate if changes were made.
In cases where reviewers are anonymous, credit should be given to 'Anonymous Referee' and the source.
The images or other third party material in this Peer Review File are included in the article's Creative Commons license, unless indicated otherwise in a credit line to the material. If material is not included in the article's Creative Commons license and your intended use is not permitted by statutory regulation or exceeds the permitted use, you will need to obtain permission directly from the copyright holder.
To view a copy of this license, visit https://creativecommons.org/licenses/by/4.0/

We thank all reviewers for their thoughtful and constructive comments on our manuscript. We have carefully considered the points made by the reviewers and attempted to respond thoroughly to these comments. In summary, we have experimentally validated our CNV calls when possible, comprehensively evaluated the quality of the CNV calling, and performed new analyses for deeper biological insights. We have also edited the manuscript throughout for clarity.

We have provided the following point-by-point response to specific reviewer concerns. Our response is colored in light blue for ease of review. Major revisions in the manuscript are highlighted in red and quoted in our responses when helpful.

Reviewer #1 (Remarks to the Author):
Chen et al performed the largest schizophrenia rCNV study of EAS ancestries to date followed by a meta-analysis with the largest EUR study previously published in NG. They identified 9 rCNV loci associated with schizophrenia in EAS, and 15 when meta-analyzed with EUR individuals.

1. This manuscript is submitted under the “Article” category, which is defined as a substantial novel research study with a complex story often involving several techniques or approaches. However, the novelty of this work mostly lies within the large sample size of EAS ancestries. Only standard analyses were carried out here using established methods. No new ideas or approaches were used.
We thank the reviewer for raising this comment. While it is true that we applied established methods, the novelty and impact of our study lie in the unprecedented sample size of individuals with East Asian (EAS) ancestry, which enabled new biological insights that were not previously attainable. Specifically:

- **Discovery of novel CNV loci:** The increased sample size allowed us to identify eight previously unreported schizophrenia-associated CNV loci. Given the large effect sizes typically associated with CNVs, these findings offer important biological insights into schizophrenia pathogenesis. For instance, suggested by the reviewer, gene set enrichment analysis of CNVs from this study revealed new biological pathways underpinning schizophrenia, such as the postsynaptic cytosol compartment.
- **First genome-wide significant CNV loci in EAS:** Prior to this study, no CNV loci had reached genome-wide significance in EAS or any non-European population. Our findings highlight the power of ancestry-specific analyses and demonstrate the critical importance of including underrepresented populations to fully characterize the genetic architecture of schizophrenia. While some CNVs may be specific to certain populations, their discovery can yield biologically meaningful insights relevant across ancestries. For example, *PCSK9* was initially discovered as a gene regulating lipid metabolism in individuals of African ancestry, yet *PCSK9* inhibitors benefit individuals globally. Similarly, our ancestry-specific findings may reveal biological mechanisms that are broadly relevant to schizophrenia pathogenesis.
- **Cross-ancestry comparison of CNV effects:** This is the first systematic genome-wide comparison of schizophrenia-associated CNVs between EAS and EUR. Our findings

revealed that the genetic risk conferred by CNVs is quite consistent across ancestries. However, certain schizophrenia-associated CNVs appear to exist across multiple ancestries, while others are ancestry-specific.

- **Rare CNV burden and PRS association:** In response to the reviewer's suggestion, we extensively investigated the collective contribution from rare CNV burden and polygenic risk scores (PRS) to schizophrenia, revealing new insights into the interplay between two major types of human genetic variations (details in the response to comment 10).

In summary, the increased sample size, novel cross-ancestry comparisons, and additional analyses introduced during revision have collectively advanced our understanding of schizophrenia genetics. Respectfully, we believe these contributions align with the scope and expectations of the *Article* category.

2. The authors reported 8 rCNV loci for the first time, however, they did not carry out any confirmatory experimental validation of these CNVs. It may not be possible to verify every CNV carrier but at least a subset of samples would be warranted.
We fully agree with the reviewer. We have validated our findings using whole-exome sequencing (WES) and/or droplet digital PCR (ddPCR) on biospecimens that we have access to.

Specifically, we have access to biospecimens from 18,335 subjects, including 54 individuals who are "carriers" of 4 of the 8 "novel" rare CNVs suggested by the reviewer for confirmatory experiments. These include 18 carriers for 15q11.2, 14 for 15q12, 3 for 15q13.3, and 15 for 1q21.2. Unfortunately, we do not have access to biospecimens from any carriers of the remaining 4 rare CNVs: 8p21.3, 11q13.1, 19p13.3, and 19q13.42 —and thus were unable to perform experimental validation for those loci.

Exome Sequencing Validation. Of the 54 carriers, 51 underwent WES and could be validated using this dataset. We performed CNV calling using GATK-gCNV on the WES data. The resulting WES-based CNV calls confirmed all CNVs in these 51 carriers at the four loci (Supplementary Table 17b).

Droplet Digital PCR (ddPCR) validation. For the 3 carriers who did not have WES data, we validated their CNVs—at loci 15q11.2, 15q12, and 1q21.2—using ddPCR. In addition, we included 7 carriers with WES-confirmed CNVs at the same loci as positive controls (3 for 15q11.2, 2 for 15q12, and 2 for 1q21.2) and 3 non-carriers as negative controls. All CNVs were confirmed by ddPCR: the 3 test samples and 7 positive controls showed the expected copy number changes, while the 3 negative controls showed no change in the copy number.

With a 100% success rate in the confirmatory experimental validation, we argue that our CNV calling pipeline is robust, and the novel rare CNV loci reported in our study are reliable. We have added this confirmatory validation to the manuscript (page 15) and Supplementary Note (pages 8-9).

3. The authors stated that their CNV calling, and QC pipeline were consistent with those used in the EUR study. However, the EUR study (ref 14) required CNVs to encompass at least 10 probes while this study 15 probes.
We appreciate that the reviewer caught this glitch and apologize for this oversight. We've revised the statement that our pipelines were consistent with those used in the EUR study.

We chose 15 probes as the threshold because it improved the quality of the CNV calling. The 15-probe pipeline called fewer CNVs than the 10-probe pipeline (4% and 1% fewer for deletions and duplications, respectively). CNVs only called using the 10-probe pipeline are less likely to be replicated by the WES data: the percentage replicated by the WES was 60% and 81% for deletions called using 10-14 probes and 15+ probes, respectively; and 63% and 80% for duplications using 10-14 probes and 15+ probes, estimated using CNVs overlapping 1+ exon (Supplementary Figure 5).

In our responses to the subsequent comments 4 and 5, we have additionally justified that applying this slightly different threshold in EAS from EUR does not affect our conclusions in the population comparison. We have revised the manuscript (pages 6 and 19) and in Supplementary Note (page 7) accordingly.

4. The authors carried out a consistency check by comparing CNVs of a subset of EUR individuals called using their pipeline vs those previously published. There are problems as to how the results were interpreted. For example, they found only 84% overlap but called this a "slight" difference and attributed this to differences in allele frequency calculation. However, item #3 above should be considered in interpreting the difference. Indeed, they found that the 16% rCNV not detected by their pipeline were smaller with fewer probes. Isn't that the obvious consequence given the different filters based on the number of probes spanned by a CNV?
We again apologize for the confusion. For this comparison, we actually used the same 10-probe threshold as in the published EUR study. While for other analyses, we indeed used 15 probes (impact addressed in comments #3 and #5). We have thoroughly revised the manuscript so that it now accurately describes what we did.

In response to this comment, we additionally called CNVs using the 15-probe threshold on the two PGC EUR samples. Results from using the 10- and 15-probe thresholds are highly similar (Supplementary Table 3), with both showing 84-88% overlap with the published CNV calls. These findings indicate that the choice of probe threshold does not substantially influence the consistency across pipelines (while it does help with the QC in EAS, response to comment 3).

Therefore, the ~16% CNVs not detected by our pipeline are not due to the difference in probe threshold. Given that the only difference between our pipeline and the one used to generate the published PGC CNV calls was the frequency used to filter the CNVs, we would like to maintain our original statement. We have added these discussions, removed the "slight" and clarified that this is an informed hypothesis rather than a definitive finding (page 7).

We have worked closely with the authors of the published PGC EUR studies. However, since the EUR publication was over eight years ago and the PGC server has since migrated from Lisa to Snellius, we no longer have access to the original files needed to directly test that the frequency calculation was driving the observed difference. Nevertheless, we hope that the additional evidence we have provided regarding the technical robustness of our pipeline (responses to comments 2, 3, 7, and 8) reassures the reviewer of our pipeline's quality and reliability.

5. In addition, the authors compared the average rCNV length and rCNV count per carrier between EAS and EUR and found both measures smaller in the EAS than in EUR samples. They attributed this observation to genotyping arrays optimized for EUR. Again, the difference in the probe number filter (item #3) should be considered here too.
Thank you for pointing this out! We have now updated our comparison considering the difference in the number of probes as the QC threshold across studies.

CNV length: Increasing the thresholds from 10 to 15 probes slightly increases the median CNV length. The median CNV length for the EAS subjects is 92.50 KB using a 15-probe threshold, compared to 91.86 KB with a 10-probe threshold. Since our conclusion is that EUR subjects have a longer median CNV length (227.01 KB in EUR vs. 92.50 KB in EAS, P = 2.2e−16), a slight increase in the EAS median, driven by the probe threshold, does not alter this conclusion; it only slightly reduces our power to detect the difference.

Count of rare CNV per Carrier: Increasing the probe threshold from 10 to 15 slightly reduces the average number of rare CNVs per carrier. Among EAS subjects, this number decreases from 1.38 with the 10-probe threshold to 1.32 with the 15-probe threshold—a modest difference of 0.06. In contrast, the difference between ancestries is substantially larger: using the 10-probe threshold, the average rare CNV count per carrier is 2.41 in EUR compared to 1.38 in EAS, a difference of 1.03. Thus, our conclusion that EUR individuals carry more rare CNVs than EAS individuals remain robust, even if the threshold introduces a slight downward bias in the estimates for EAS.

In summary, our additional analyses showed that the difference in the probe number filter did not introduce a meaningful bias to invalidate our conclusion. We appreciate the reviewer for pointing this out and have added these discussions to the manuscript (page 7) and supplementary note (pages 7-8).

6. Line 219 – The Wilcoxon signed-rank test is for paired samples. Wilcoxon rank-sum test for two independent samples is appropriate here.
We thank the reviewer for pointing this out. We used the Wilcoxon signed-rank test because we treated the CNV frequencies in the EAS and EUR populations as *paired* observations – i.e., the frequency in EAS was matched to the corresponding frequency in EUR at the same CNV locus. In response to the reviewer's suggestion, we also performed a Wilcoxon rank-sum test, treating the frequencies as *unpaired* across populations. The difference in CNV frequency remained

statistically significant (P = 0.002). We have included the rank-sum test results in the revised text (page 11).

7. The study consisted of multiple samples and a variety of genotyping platforms. It would be useful to present an evaluation/comparison on data quality metrics, for example, LRR-SD from the Illumina arrays.

Thank you for the suggestion! We have now added data quality metrics, including LRR standard deviation (LRR-SD), to Supplementary Figure 1 (copied here for convenience). Upon visual inspection, the quality metrics look very good. The metrics do suggest that array type is an important variable to account for, and that GC wave adjustment is a good idea. Indeed, in the original manuscript, we had controlled for array type in all analyses and applied the -gcmodel adjustment during CNV calling.

To assess the potential impact of LRR-SD on our findings, we performed logistic regression to evaluate the association between schizophrenia case-control status and LRR-SD, adjusting for sex, array type, and the top five principal components derived from genotypes. We did not observe a significant association between the disease status and LRR-SD (OR per standard deviation increase in LRR-SD = 0.55, 95% CI: [0.30, 1.01], P = 0.054). In addition, LRR-SD only explained 0.0083% of the variance in disease case-control status (Cox and Snell pseudo-R^2 from logistic regression). We also found that, among EAS individuals, adjusting for LRR-SD had a negligible impact on the association between case-control status and genome-wide rare CNV burden (Supplementary Figure 2). Taken together, these results suggest that LRR-SD is unlikely to have a meaningful impact on our conclusion.

We have added these new analyses to the manuscript (pages 6 and 8), and Supplementary Note (page 8).

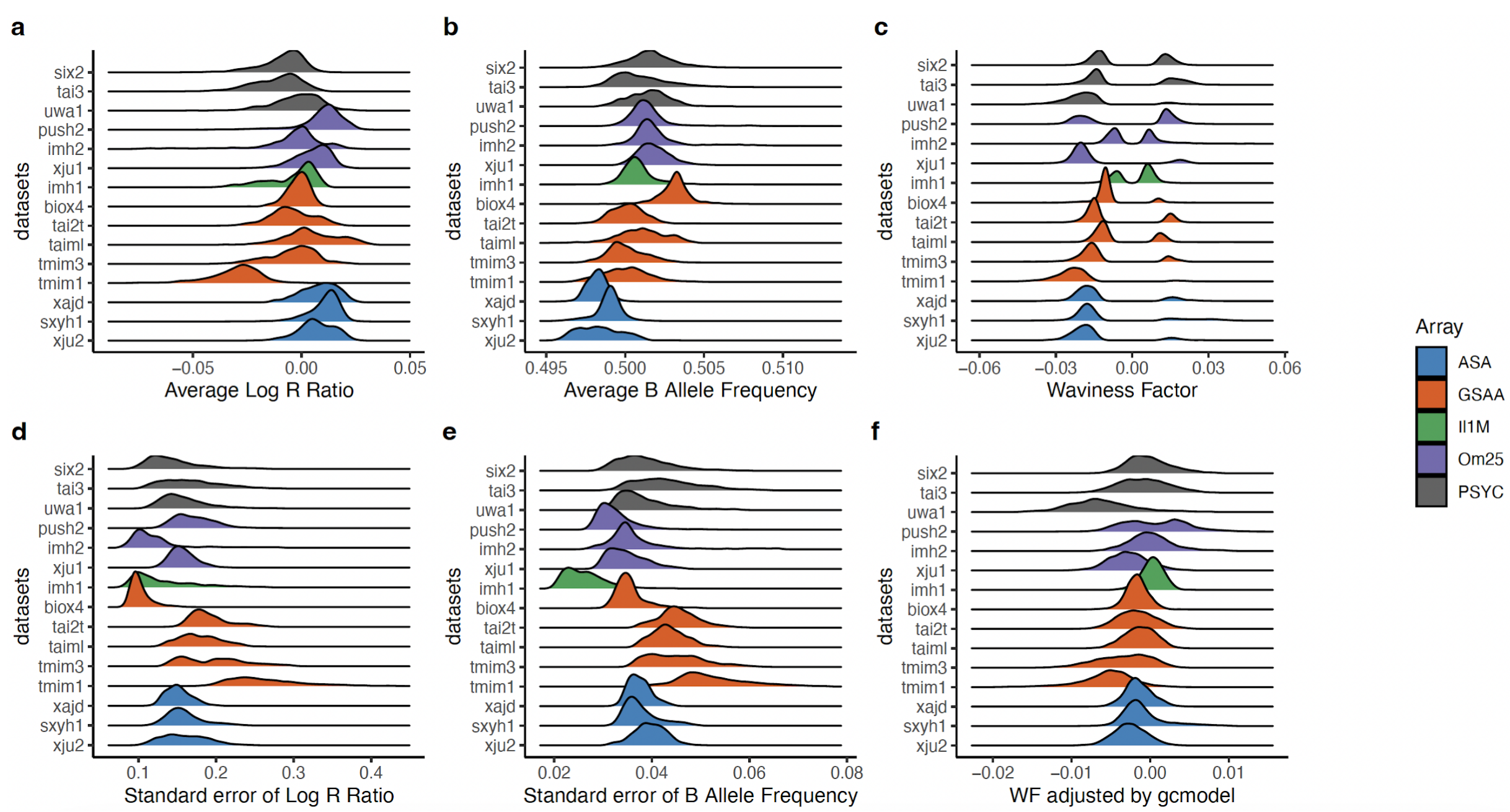

Supplementary Figure 1. The distribution of sample QC metrics across EAS samples. Density plot for **a,** average Log R Ratio; **b,** average B allele Frequency; **c,** waviness factor; **d,** standard error of Log R Ratio; **e,** standard error of B allele Frequency; **f,** waviness factor adjusted by gcmodel. Color represents the array types. ASA: Infinium Asian Screening Array; GSAA: Infinium Global Screening Array; Il1M: Human1M-Duo; PSYC, Infinium PsychArray; Om25, Infinium OmniZhongHua.

8. Maybe I missed those plots somewhere, but providing probe intensity plots for some of those novel rCNVs identified would be helpful.
Thank you for the suggestion. This is a good idea. We have included probe intensity plots for carriers of all eight novel rare CNVs as Supplementary Data 1. As an example, here are the probe intensity plots of 5 carriers of the 1q21.2 locus (copied from Supplementary Data 1).

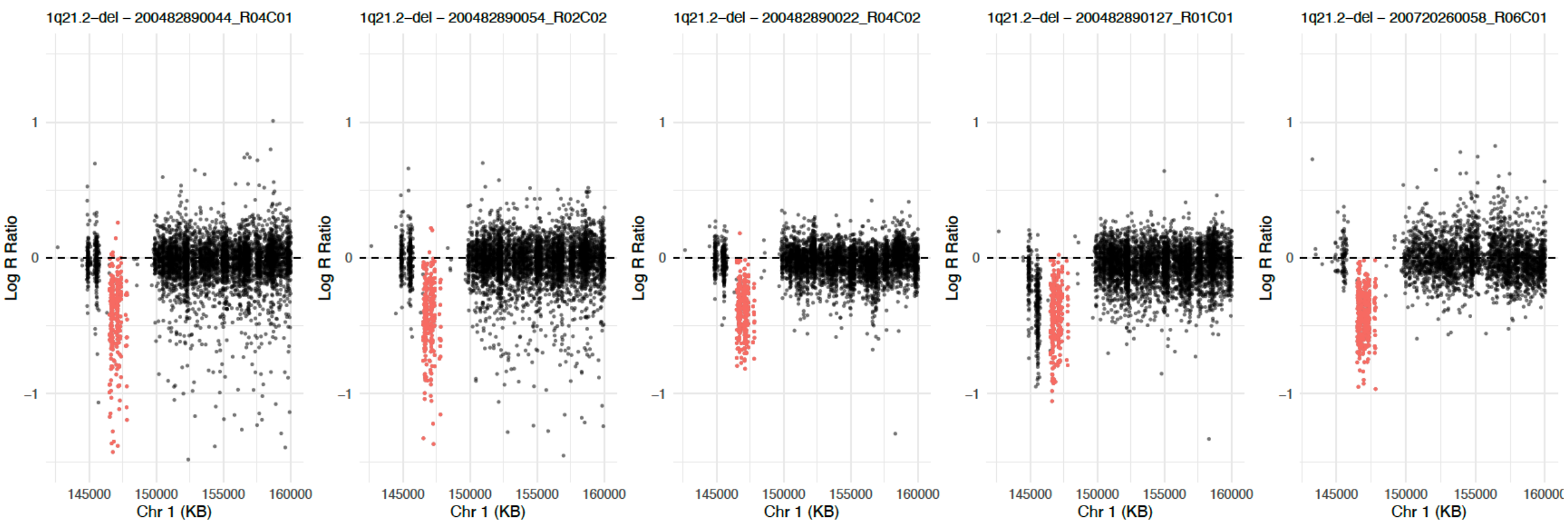

Supplementary Data 1. The Log R Ratio (LRR) is shown for CNV carriers across the region spanning the novel CNV locus ± 5× the CNV length. Each dot represents the LRR at an individual probe. The x-axis denotes genomic position, and the y-axis represents the LRR. Red points indicate probes located within the boundaries of the novel CNV locus.

9. Supplementary Note line 185 – "iPattern, on other hand, also uses an HMM-based approach to call CNVs" - This is wrong. In addition, the authors should provide details on how they formed iPattern calling batches that includes multiple samples in each.
Thank you for spotting this! We have revised the manuscript accordingly and included additional details on how the iPattern calling batches were formed in the supplementary note (page 6).

10. More analyses could be done to improve the manuscript and provide biological insights to the novel association loci. For example, gene-set (pathway) analysis, evaluation of the CNV formation mechanism (NAHR-mediated?), comparison of the relative contribution of rCNV burden and polygenic risk score from common variant associations.
We appreciate this suggestion and have added several new analyses to the revised manuscript.

Gene-Set Enrichment Analysis: We performed gene-set enrichment analysis using the SynGO ontology database and results from the EAS, EUR (published), and EAS+EUR meta-analysis (page 21, Methods). EAS and the meta-analysis led to the discovery of significantly enriched gene sets that were not discovered using the published EUR CNVs, including the gene

set for the postsynaptic cytosol and the gene set for transmitter-gated ion channel activity involved in the regulation of postsynaptic membrane potential. We have added these findings to the manuscript (pages 10 and 14-15, Supplementary Table 11).

CNV Formation Mechanism: We have annotated the CNV formation mechanism following established approaches (pages 21-22, Methods). The annotations have been added to Table 2 and are described on page 10. Consistent with prior findings, most significant CNV loci are likely mediated by NAHR.

Collective contribution from PRS and rare CNV burden to disease risk: We found that among cases, carriers of large deletions showed significantly lower PRS than non-carriers. We also found rare CNV burden contributes a smaller yet significant amount of risk in addition to the risk conferred by PRS. Lastly, we observed a small yet significant interaction between PRS and rare CNV burden. We have added these findings to the manuscript (pages 8-9 and 22-23), Supplementary Table 9, and Supplementary Figure 3.

Reviewer #2 (Remarks to the Author):
Contribution of copy number variants to schizophrenia in East Asian populations by Chen et al. Testing reported copy number variants (CNVs) associated with schizophrenia in a substantial East Asian sample (20,903 cases and 23,258 controls) is both important and timely. Additionally, the authors investigate this sample for novel CNVs and identify associations with eight new variants.
However, several issues need to be addressed:

1. First, the authors introduce the term "rare copy number variants (rCNVs)." More conventional terms like "recurrent CNVs," "neuropsychiatric CNVs," or "schizophrenia CNVs" are typically used in the literature. Is there a reason to introduce rCNV – can't they simply be called rare CNVs and those that associate with schizophrenia called schizophrenia CNVs?
Thank you for your feedback. The term "rare copy number variants (rCNVs)" was initially adopted from ref.1. However, to eliminate potential ambiguity and ensure consistency with established terminology, we have replaced "rCNVs" with "rare CNVs" throughout the manuscript. For those specifically associated with schizophrenia, we now refer to them as "schizophrenia-associated rare CNVs."

2. Secondly, the authors use conventional names for CNVs already associated with schizophrenia but do not adhere to the standard start and stop positions for these CNVs (e.g., the 15q11.2 is not referring to the typical 15q11.2 BP1-BP2 CNV). Additionally, the authors group the 16p11.2 distal and 16p11.2 proximal deletions together, which are usually tested separately. This creates confusion when data are presented under the same CNV name but with different genomic positions. This inconsistency needs to be corrected by using the conventional start and stop positions for the different CNVs. The manuscript would be much clearer if the approximately 25 previously reported schizophrenia-associated CNVs were tested in the East Asian sample and presented in this manuscript using the same definitions that have been standard in the literature for the last 20 years.

Thank you for pointing this out. In the revised manuscript, we have separately reported the results for the 16p11.2 distal and 16p11.2 proximal deletions.

We fully agree with the reviewer's suggestion to use conventional names and annotations for CNVs. In the original manuscript, we annotated the CNV loci using Cytoband annotations from the UCSC Genome Browser[2]. We adopted this approach because CNV annotations in the schizophrenia literature have been inconsistent (e.g., ref.3,4), and several of our identified CNVs are novel and not reported in prior studies. Some of the apparent discrepancies may also stem from our use of lift over to convert genomic coordinates from hg18 (used in the PGC study) to hg19, which may result in differences in start/end positions relative to earlier publications. We would be happy to update our annotations if a more appropriate resource exists for CNVs in the context of schizophrenia research and would be grateful if the reviewer could recommend such a resource. Our current annotation approach has now been described in the revised manuscript for clarity (page 21).

For the "approximately 25 previously reported schizophrenia-associated CNVs" referred to by the reviewer, we thought the reviewer meant the 31 CNVs associated with schizophrenia reported in the PGC European ancestry CNV study[3]. If our interpretation is incorrect, we would be happy to revise accordingly and would appreciate it if the reviewer could suggest the intended reference. For these 31 CNV loci, the results in EAS are highly consistent with those from the EUR ancestry. We have added these findings to the revised manuscript (page 14, Supplementary Tables 13 and 14, and Extended Data Figure 7).

3.Also, authors have to be more specific, two examples below:
In the abstract you say:
"Genes significantly associated with schizophrenia in these rCNV loci tend to be intolerant to protein-truncating variants."
Which CNVs are you referring to? The eight novel CNVs or CNVs that have already been associated with schizophrenia? What does tend mean in this context? – can you be more specific?
Sorry for the confusion. We were referring to the 15 CNV loci identified as significantly associated with schizophrenia in the meta-analysis. We found that genes located within these loci were significantly enriched for loss-of-function-intolerant genes ($P = 2.86e-16$, Fisher's exact test) and had a significantly higher pLI score compared with other genes examined (mean pLI: 0.28 vs. 0.17; $P = 0.001$, two-sided t-test). These results were presented in the original manuscript (pages 16-17 in the current revision). We have revised the abstract for clarity and have included the updated version below for the reviewer's convenience.

Genes located within the 15 schizophrenia-associated rare CNV loci were significantly less tolerant to loss-of-function variants compared to genes in other CNV loci.

4. Also in the abstract you say:

"Many novel rCNV associations in this study had a much higher carrier frequency in EAS and EUR, underscoring the pressing need to expand the population diversity to capture the complete genetic risk factors underpinning schizophrenia."
Here you should be more specific. What does "much higher" mean – readers may get the impression that you are referring to common variants?
Thank you for pointing this out! We have revised the abstract for clarity and have included the updated version below for the reviewer's convenience.

Novel rare CNVs associated with schizophrenia in EAS showed substantially higher carrier frequencies compared to their frequencies in EUR (mean carrier frequency 0.38% vs. 0.0017%). Overall, this study underscores the importance of increasing population diversity to fully capture the genetic underpinnings of schizophrenia.

5. For these relatively rare variants, the primary concerns revolve around the methodology used to call CNVs in this category. Additionally, it's important to ensure that cases and controls are well-matched in terms of age and gender. The study used different genotyping platforms, raising the question of whether the SNP content is comparable across the CHIPs used, including the number of SNPs. It is important to investigate if there are any batch effects seen for the eight novel CNVs identified. Another key point is to assess whether the frequency and odds ratios (OR) are consistent across all 15 East Asian (EAS) schizophrenia samples included in this study. A forest plot for these 15 East Asian samples is required to visualize this data.
Thank you for raising these important points. We have addressed each concern as follows:

CNV calling: We employed a combined approach using PennCNV and iPattern, leveraging their complementary algorithms to enhance CNV detection across multiple genotyping platforms. This integrative strategy improves calling accuracy and reduces false positives. In the revised manuscript, as requested by Reviewer 1, we validated our CNV calling pipeline using exome sequencing and droplet digital PCR (ddPCR) (response to Reviewer 1, Comment 2). The validation was 100% successful, demonstrating the robustness of our CNV detection.

Sex matching: Across the 15 EAS samples, the sex distribution was generally balanced, with moderate variation between cases and controls. The proportion of male participants ranged from 41.4% to 64.9% across samples. Among cases, male representation ranged from 33.7% to 74.3%, and among controls, from 18.7% to 63.3%. Overall, the combined sample consisted of approximately 51% males and 49% females. Importantly, no dataset exhibited extreme sex imbalance (e.g., all-male cases or all-female controls). These details are provided in the Reporting Summary and Supplementary Table 1. Additionally, sex was included as a covariate in all genome-wide and gene-level regression models to account for potential confounding.

Age matching: Unfortunately, age information was not available across all samples. Among those with available data, the mean ages were as follows: PUSH2 = 30.6, XAJD = 43.4, XJU2 = 37.5, and SXYH1 = 38.0. Within each dataset, no significant differences in age distribution were observed between cases and controls after multiple testing correction (P_{push2} = 0.23, P_{xajd} =

0.044, P_{xju2} = 0.076, P_{sxyh1} = 0.038, two-sided *t* test). We have acknowledged this limitation in the Discussion (page 18).

Age distribution within each sample

Dataset	Group	25% quantile	50% quantile	75% quantile
PUSH2	case	25	30	40
PUSH2	control	24	32	40
XJU2	case	31	47	54
XJU2	control	30	41	51
SXYH1	case	28	38	49
SXYH1	control	25	34	44
XAJD	case	38	47	54
XAJD	control	30	41	51

Batch Effects in associations: We confirm that the genotyping platform was included as a covariate in all association analyses to account for potential batch effects. As also suggested by Reviewer 1, we evaluated the data quality metrics – specifically LRR standard deviation (LRR-SD) – on association results. Our analyses confirmed good quality in metrics, we have controlled potential confounding factors, and LRR-SD is unlikely to have a meaningful impact on our conclusion (response to Reviewer 1, Comment 7).

We encountered challenges in generating an informative forest plot due to the large standard errors associated with rare CNVs. To address this, we developed a modified version of the forest plot that uses sample count instead of standard error, which we believe provides a more informative representation. We visualized the eight novel rare CNV loci identified in this study (figure included here for the reviewer's convenience). All eight CNV loci showed consistent support across multiple independent samples, providing confidence in their quality. We have added this figure and additional discussions to the manuscript (pages 15 and 18) and Supplementary Figure 4.

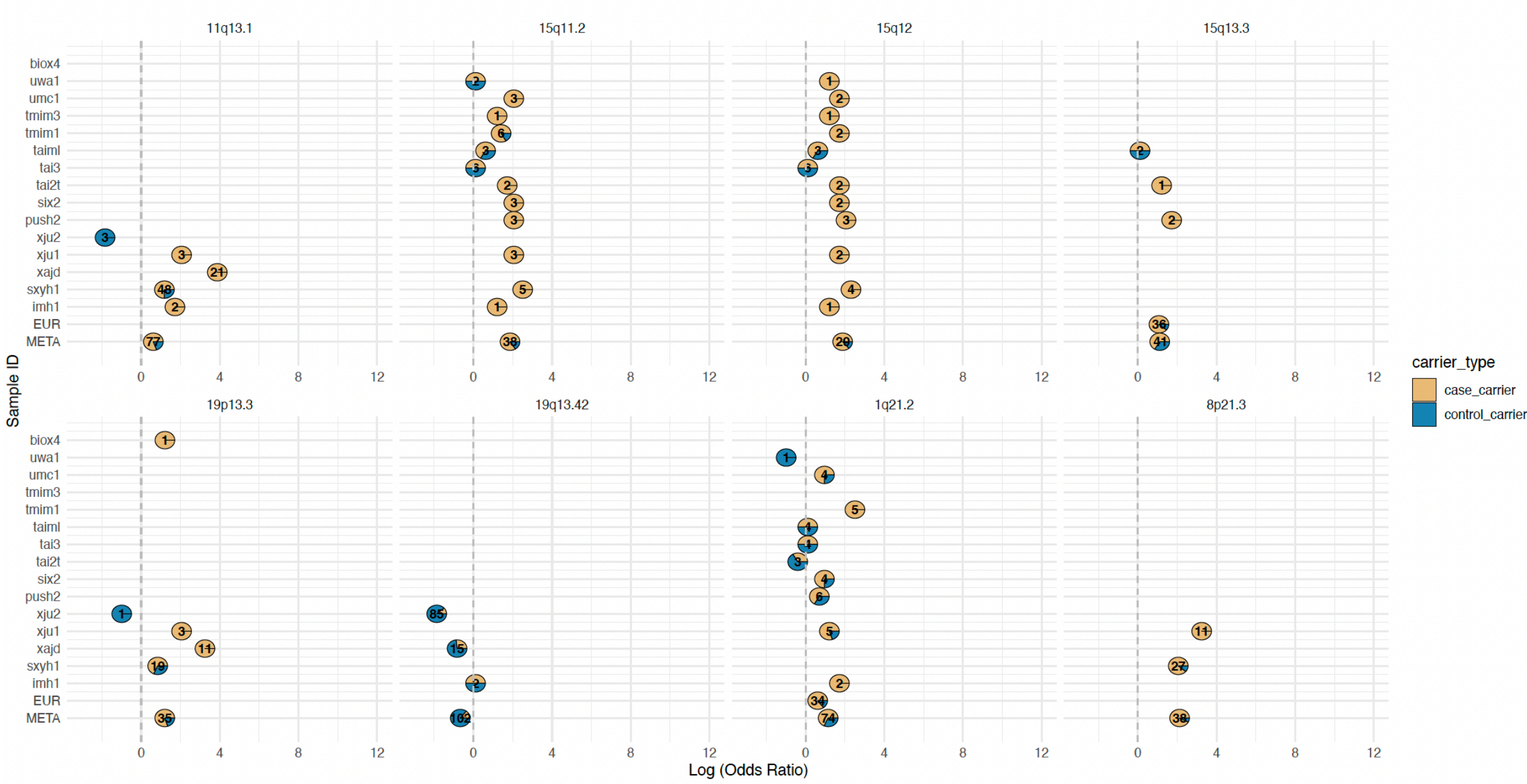

Supplementary Figure 4: Locus-wide rare CNV burden stratified by samples. The x-axis represents the odds ratio (OR) on a logarithmic scale, and the y-axis lists the sample IDs. The vertical dashed line indicates an OR of 1. Each OR estimate is displayed as a pie chart, with the proportion of case carriers (orange) and control carriers (blue) represented within each sample shown. The number inside each pie chart indicates the total number of carriers in that sample.

References

1. Collins, R. L. *et al.* A cross-disorder dosage sensitivity map of the human genome. *Cell* **185**, 3041-3055.e25 (2022).
2. Cheung, V. G. *et al.* Integration of cytogenetic landmarks into the draft sequence of the human genome. *Nature* **409**, 953–958 (2001).
3. Marshall, C. R. *et al.* Contribution of copy number variants to schizophrenia from a genome-wide study of 41,321 subjects. *Nat. Genet.* **49**, 27–35 (2017).
4. Rees, E. *et al.* Analysis of intellectual disability copy number variants for association with schizophrenia. *JAMA Psychiatry* **73**, 963–969 (2016).

We thank the reviewer for the thoughtful and constructive comments on our manuscript. In this revision, we have addressed the reviewer's remaining concerns, with the point-by-point response provided below and the changes to the manuscript highlighted in red.

Reviewer #2

The authors have addressed many of my previous comments and concerns. However, there remain a few points that require further clarification. I encourage the authors to more clearly specify and clarify the CNVs being studied for the interpretation of the findings.

As an example, a couple of questions and suggestions regarding Table 2 in your manuscript:

1. Top Rare CNV Gene: In Table 2, there is a column labeled "Top rare CNV gene". Below the table, you note that the OR, P-value, and top rare CNV gene correspond to the most significantly associated gene within each locus. Could you clarify how the top rare CNV gene is determined? Specifically, is this based on statistical significance from gene-level burden testing, or simply the most well-known or previously reported gene within the region?

   The 'Top rare CNV gene' was defined as the gene within each locus that had the most significant p-value in the gene-level burden test. In instances where multiple genes shared the same significance, one gene was manually selected. While Supplementary Table 7 reported the full list of genes and their corresponding significance, we acknowledge that the manual selection introduced bias in Table 2. Therefore, in this revision, we reported all genes that shared the most significant p-value in the gene-level burden test in Table 2. We thank the reviewer for identifying this issue and appreciate the opportunity to clarify our approach.

2. References for CNV associations: It would be very helpful to include references in Table 2 to support the associations listed. For example, the 15q11.2 duplication is listed, but to my knowledge, there is no strong evidence for its association with schizophrenia. In fact, large cohorts such as the UK Biobank have reported no significant association between duplications at 15q11.2 (BP1-BP2 spanning NIPA1, NIPA2, CYFIP1, and TUBGCP5) and schizophrenia.

   We have added references supporting "previously reported" and "previously implicated" loci in Table 2 and Supplementary Table 7. This annotation and the references were taken from the earlier PGC CNV study (Supplementary Table 1, Marshall et al., *Nature Genetics* 2017) and matched to our study using the cytoband (the 'Locus' column in Table 1 and Supplementary Table 7).

   The supporting evidence for the 15q11.2 duplication was primarily from the broader 15q11.2–q13 region, often referred to as the Prader–Willi/Angelman syndrome (PWS/AS) region[1,2]. Only one family-based study provided limited evidence implicating the smaller 15q11.2 interval[3]. We fully agree with the reviewer that no previous studies provided strong statistical support directly

associating 15q11.2 BP1-BP2 duplications with schizophrenia, and that the level of evidence is weak in prior studies for the association between 15q11.2 deletion and schizophrenia. Nevertheless, we prefer to retain the annotation for consistency with the previous PGC publication, as changing it could introduce additional sources of confusion. This approach is also conservative, helping us avoid overstating the novelty of our findings. To minimize ambiguity, we have provided a more precise annotation of this region (response to the next comment).

3. Could there be a mix-up with 15q11.2 duplications that extend beyond BP2, for example, to CHRNA7 (i.e., BP1–BP5), which are associated with schizophrenia? Is it possible that the CNV definition differs in the Asian cohorts—for example, if longer duplications spanning BP1–BP5 are grouped under “15q11.2 duplication”?

   As the 15q11–q13 region is a known genomic rearrangement hotspot, we re-analyzed this region using the five previously reported breakpoints (BP1-BP5)[4]. While there was no significant schizophrenia association with genes implicated by CNVs between BP1 and BP2, we observed significant associations for genes upstream of BP1 and between BP2 and BP3 (Supplementary Figure 6). The associations located upstream of BP1 fall within the 15q11.2 cytoband and do not extend into adjacent cytobands such as 15p11.2 and 15q12 or beyond BP3.

   We agree that it was confusing to use the broad "15q11.2 " annotation given the complexity of this region. We have therefore annotated the region upstream of BP1 as **15q11.2, proximal**, and the significant associations between BP2 and BP3 as **15q11.2-q12 (BP2-BP3)**.

   In addition, we have refined the genomic coordinates in Table 2 so that the start and end positions reflect only the CNV breakpoints containing genes that reach genome-wide significance. This was done for all EAS CNV loci and provides a higher resolution than the cytoband-level annotation.

   We thank the reviewer for raising this important point. We hope the changes we made have clarified the confusion. We are happy to make additional revisions to our locus annotation if the reviewer has any recommendations.

I think clarifying this distinction and providing references for each listed locus would improve the transparency and robustness of Table 2.

We fully agree!

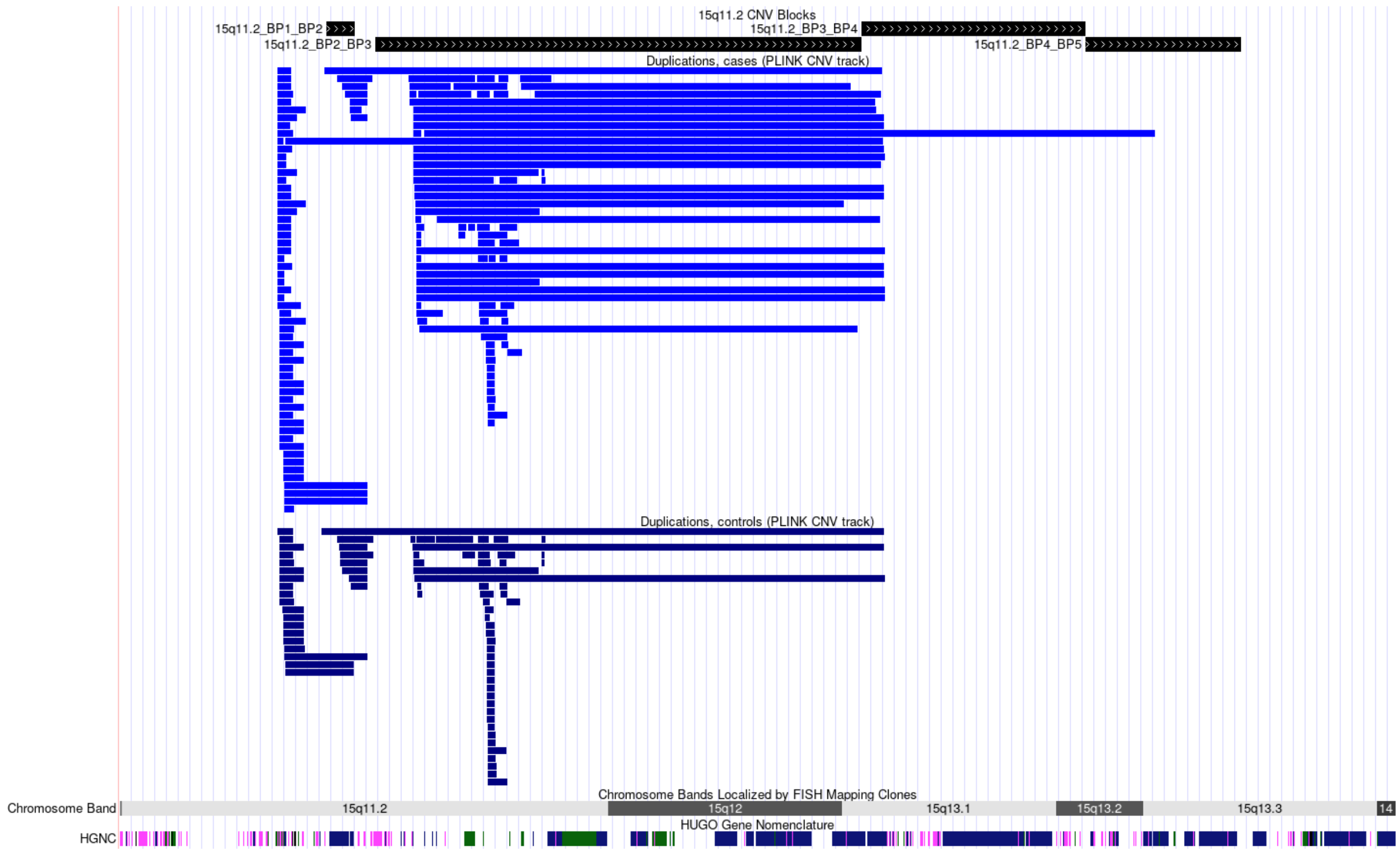

Supplementary Figure 6. CNV duplications at chromosome 15q.
CNV tracks display duplications detected in cases and controls from the EAS samples.
We labeled the five previously reported breakpoints, BP1-BP5, following ref 25.
15q11.2BP2-BP3 is part of the PWS/AS region that was previously reported as associated with schizophrenia.

Reference:

1. Isles, A. R. *et al.* Parental origin of interstitial duplications at 15q11.2-q13.3 in schizophrenia and neurodevelopmental disorders. *PLoS Genet.* **12**, e1005993 (2016).

2. Rees, E. *et al.* Analysis of copy number variations at 15 schizophrenia-associated loci. *Br. J. Psychiatry* **204**, 108–114 (2014).

3. Maiti, S., Kumar, K. H. B. G., Castellani, C. A., O'Reilly, R. & Singh, S. M. Ontogenetic de novo copy number variations (CNVs) as a source of genetic individuality: studies on two families with MZD twins for schizophrenia. *PLoS One* **6**, e17125 (2011).

4. Lusk, L., Vogel-Farley, V., Distefano, C. & Jeste, S. Maternal 15q duplication syndrome. in *GeneReviews(®) (University of* (Washington, Seattle, Seattle (WA, 1993).
