# Supplementary material for: Contribution of copy number variants to schizophrenia in East Asian populations
Source: Nat Genet. 2026 Sep 11;58(10):2514–24. doi: 10.1038/s41588-026-02732-6 (PMC13649597; doi:10.1038/s41588-026-02732-6)
Supplement: Supplementary file 4 [file 41588_2026_2732_MOESM5_ESM.pdf]

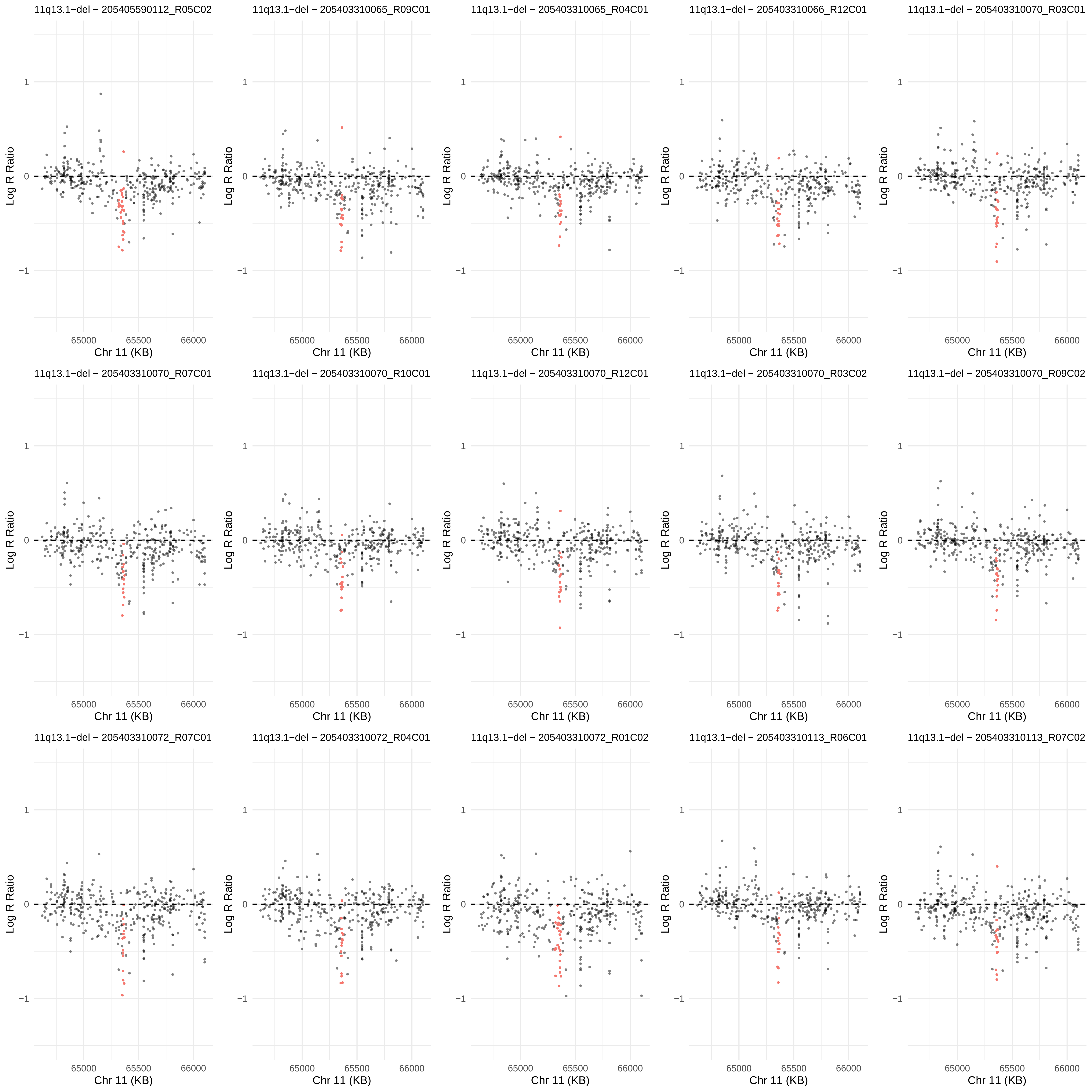

11q13.1−del − 205405590112_R05C02
11q13.1−del − 205403310065_R09C01
11q13.1−del − 205403310065_R04C01
11q13.1−del − 205403310066_R12C01
11q13.1−del − 205403310070_R03C01
11q13.1−del − 205403310070_R07C01
11q13.1−del − 205403310070_R10C01
11q13.1−del − 205403310070_R12C01
11q13.1−del − 205403310070_R03C02
11q13.1−del − 205403310070_R09C02
11q13.1−del − 205403310072_R07C01
11q13.1−del − 205403310072_R04C01
11q13.1−del − 205403310072_R01C02
11q13.1−del − 205403310113_R06C01
11q13.1−del − 205403310113_R07C02
Log R Ratio
1
0
−1
65000
65500
66000
Chr 11 (KB)

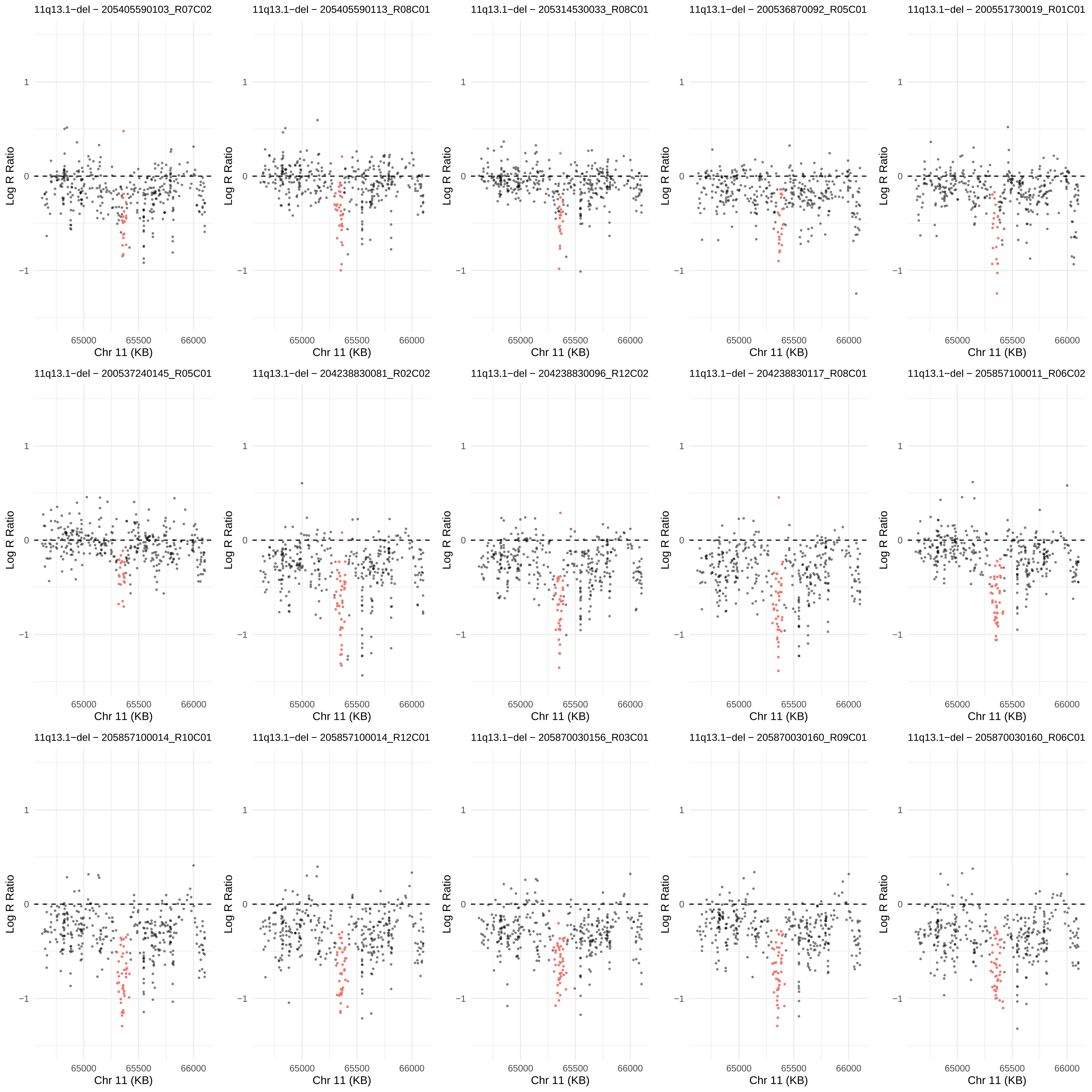
11q13.1−del − 205405590103_R07C02
11q13.1−del − 205405590113_R08C01
11q13.1−del − 205314530033_R08C01
11q13.1−del − 200536870092_R05C01
11q13.1−del − 200551730019_R01C01
11q13.1−del − 200537240145_R05C01
11q13.1−del − 204238830081_R02C02
11q13.1−del − 204238830096_R12C02
11q13.1−del − 204238830117_R08C01
11q13.1−del − 205857100011_R06C02
11q13.1−del − 205857100014_R10C01
11q13.1−del − 205857100014_R12C01
11q13.1−del − 205870030156_R03C01
11q13.1−del − 205870030160_R09C01
11q13.1−del − 205870030160_R06C01
Log R Ratio
Chr 11 (KB)
1
0
−1
65000
65500
66000

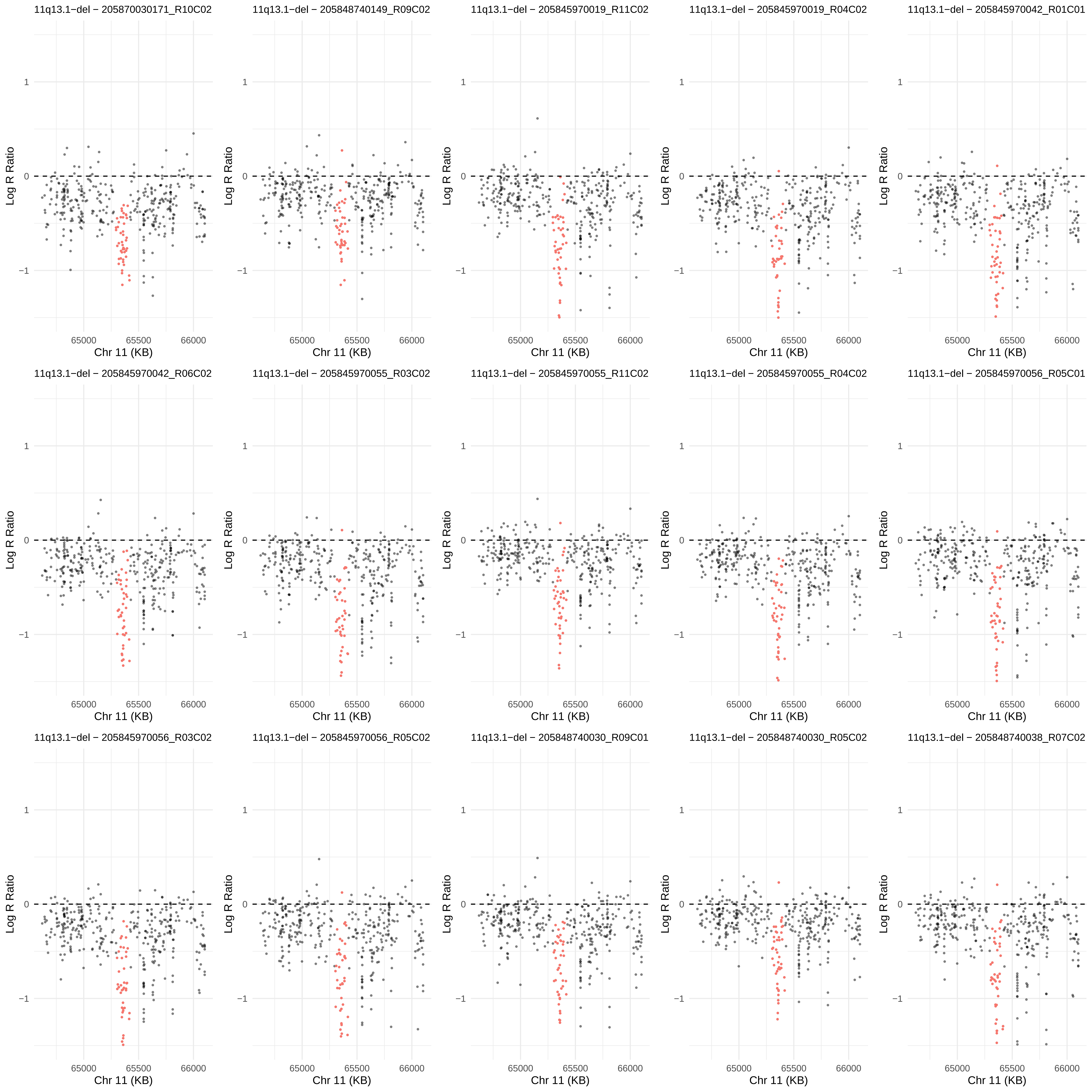
11q13.1−del − 205870030171_R10C02
11q13.1−del − 205848740149_R09C02
11q13.1−del − 205845970019_R11C02
11q13.1−del − 205845970019_R04C02
11q13.1−del − 205845970042_R01C01
11q13.1−del − 205845970042_R06C02
11q13.1−del − 205845970055_R03C02
11q13.1−del − 205845970055_R11C02
11q13.1−del − 205845970055_R04C02
11q13.1−del − 205845970056_R05C01
11q13.1−del − 205845970056_R03C02
11q13.1−del − 205845970056_R05C02
11q13.1−del − 205848740030_R09C01
11q13.1−del − 205848740030_R05C02
11q13.1−del − 205848740038_R07C02
Log R Ratio
Chr 11 (KB)
65000
65500
66000
1
0
−1

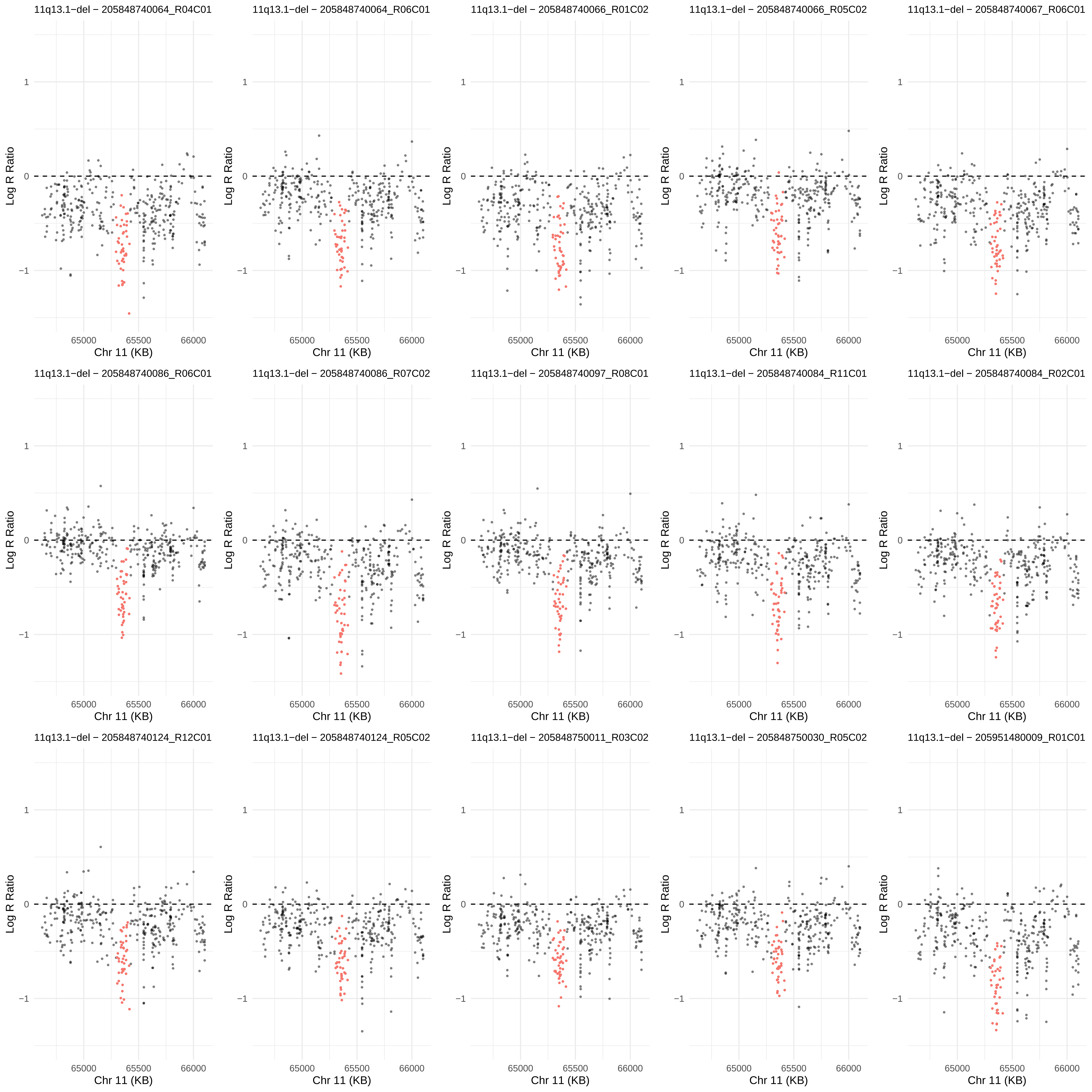
11q13.1−del − 205848740064_R04C01
11q13.1−del − 205848740064_R06C01
11q13.1−del − 205848740066_R01C02
11q13.1−del − 205848740066_R05C02
11q13.1−del − 205848740067_R06C01
11q13.1−del − 205848740086_R06C01
11q13.1−del − 205848740086_R07C02
11q13.1−del − 205848740097_R08C01
11q13.1−del − 205848740084_R11C01
11q13.1−del − 205848740084_R02C01
11q13.1−del − 205848740124_R12C01
11q13.1−del − 205848740124_R05C02
11q13.1−del − 205848750011_R03C02
11q13.1−del − 205848750030_R05C02
11q13.1−del − 205951480009_R01C01
Log R Ratio
Chr 11 (KB)
1
0
−1
65000
65500
66000

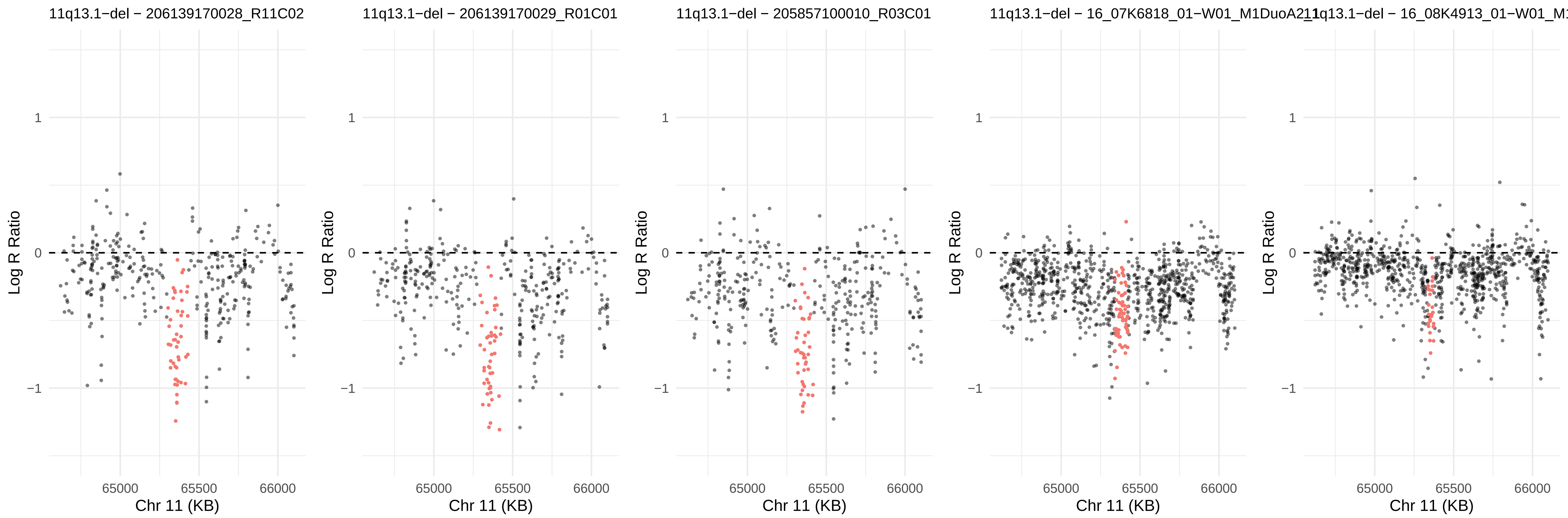
11q13.1−del − 206139170028_R11C02
11q13.1−del − 206139170029_R01C01
11q13.1−del − 205857100010_R03C01
11q13.1−del − 16_07K6818_01−W01_M1DuoA21
11q13.1−del − 16_08K4913_01−W01_M1
Log R Ratio
1
0
−1
65000
65500
66000
Chr 11 (KB)

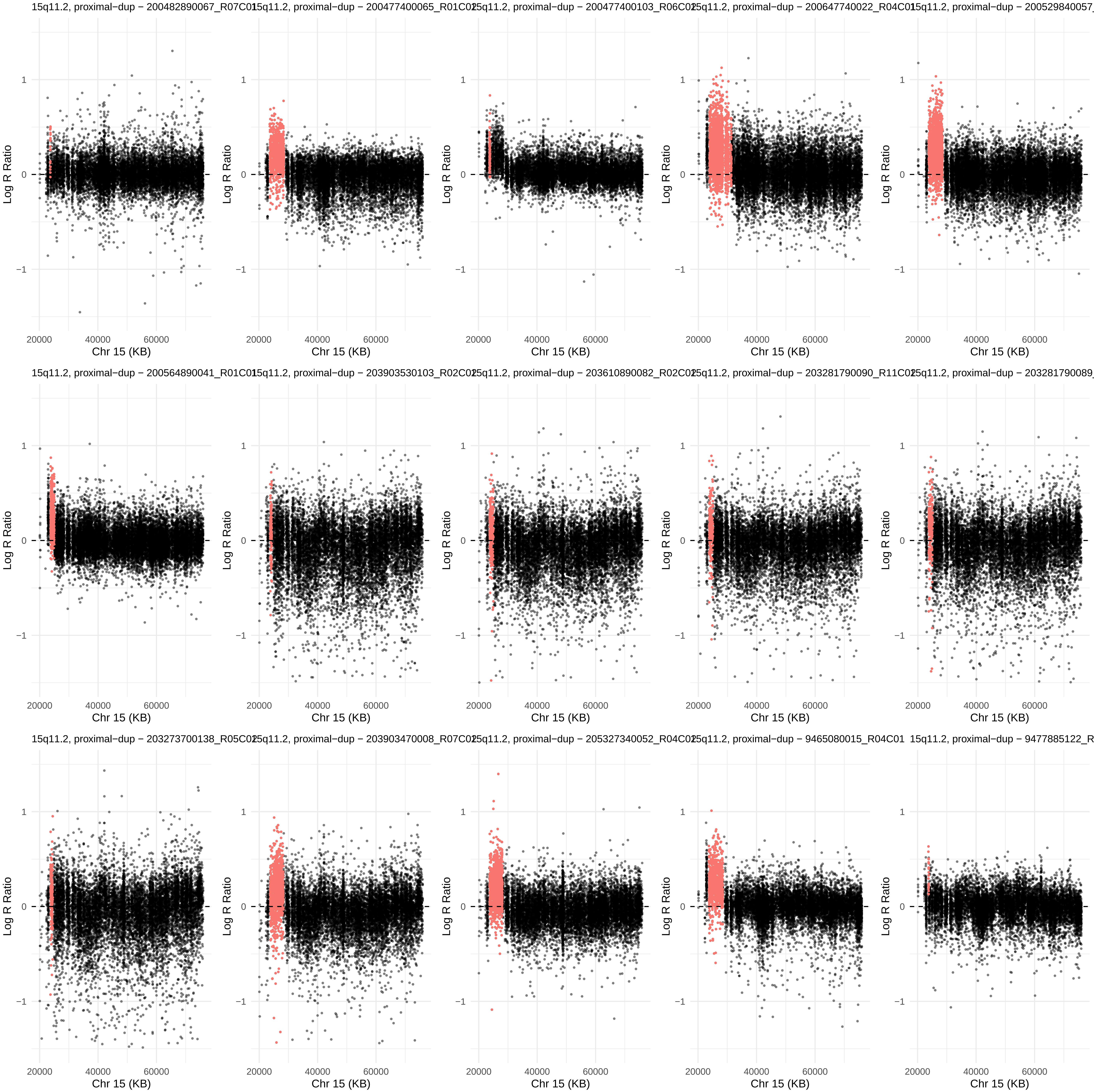
15q11.2, proximal−dup − 200482890067_R07C01
15q11.2, proximal−dup − 200477400065_R01C0
15q11.2, proximal−dup − 200477400103_R06C0
15q11.2, proximal−dup − 200647740022_R04C0
15q11.2, proximal−dup − 200529840057_
15q11.2, proximal−dup − 200564890041_R01C0
15q11.2, proximal−dup − 203903530103_R02C0
15q11.2, proximal−dup − 203610890082_R02C0
15q11.2, proximal−dup − 203281790090_R11C0
15q11.2, proximal−dup − 203281790089_
15q11.2, proximal−dup − 203273700138_R05C0
15q11.2, proximal−dup − 203903470008_R07C0
15q11.2, proximal−dup − 205327340052_R04C0
15q11.2, proximal−dup − 9465080015_R04C01
15q11.2, proximal−dup − 9477885122_R
Log R Ratio
1
0
−1
20000
40000
60000
Chr 15 (KB)

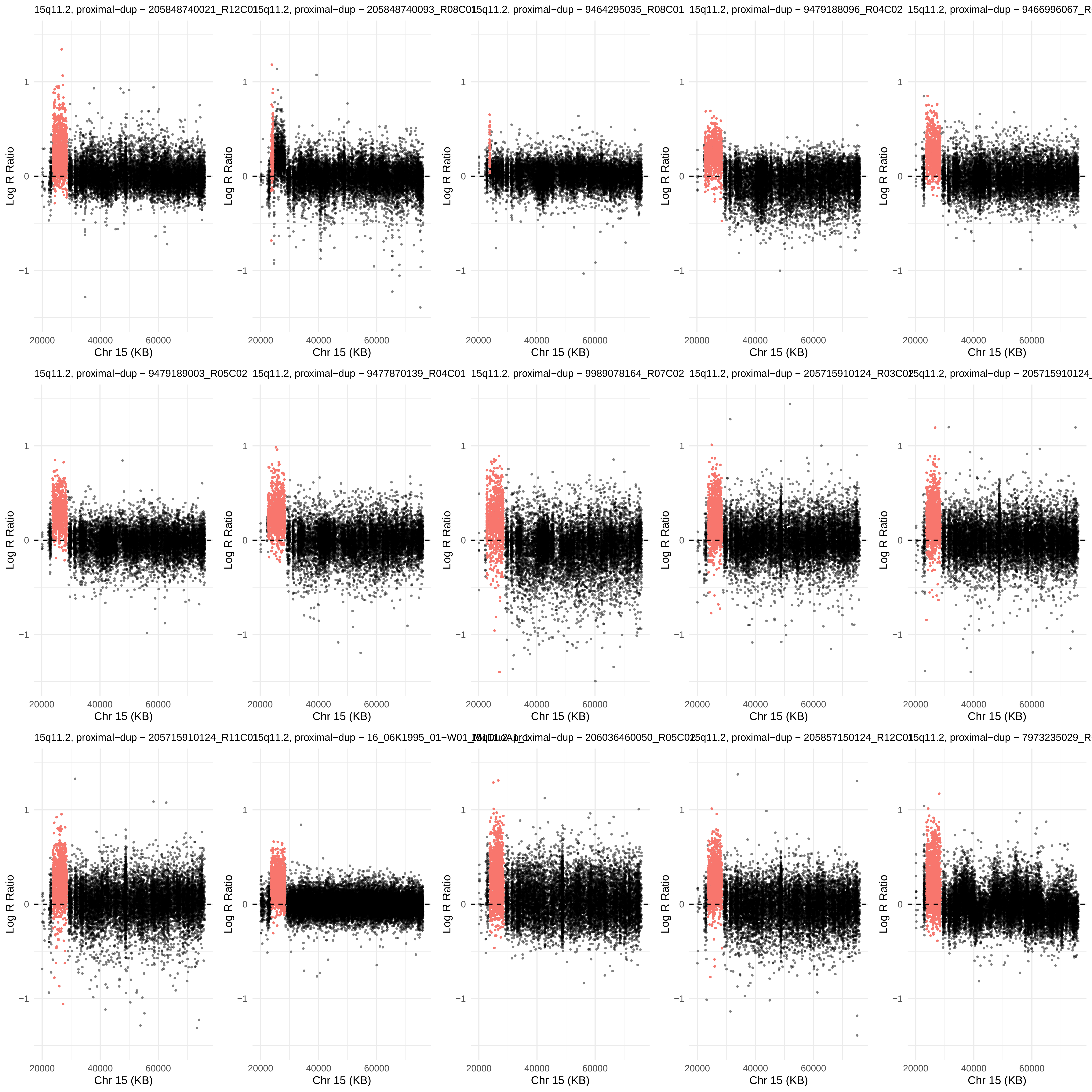
15q11.2, proximal−dup − 205848740021_R12C01
15q11.2, proximal−dup − 205848740093_R08C01
15q11.2, proximal−dup − 9464295035_R08C01
15q11.2, proximal−dup − 9479188096_R04C02
15q11.2, proximal−dup − 9466996067_
15q11.2, proximal−dup − 9479189003_R05C02
15q11.2, proximal−dup − 9477870139_R04C01
15q11.2, proximal−dup − 9989078164_R07C02
15q11.2, proximal−dup − 205715910124_R03C01
15q11.2, proximal−dup − 205715910124_
15q11.2, proximal−dup − 205715910124_R11C01
15q11.2, proximal−dup − 16_06K1995_01−W01_
15q11.2, proximal−dup − 206036460050_R05C01
15q11.2, proximal−dup − 205857150124_R12C01
15q11.2, proximal−dup − 7973235029_R
Log R Ratio
1
0
−1
20000
40000
60000
Chr 15 (KB)

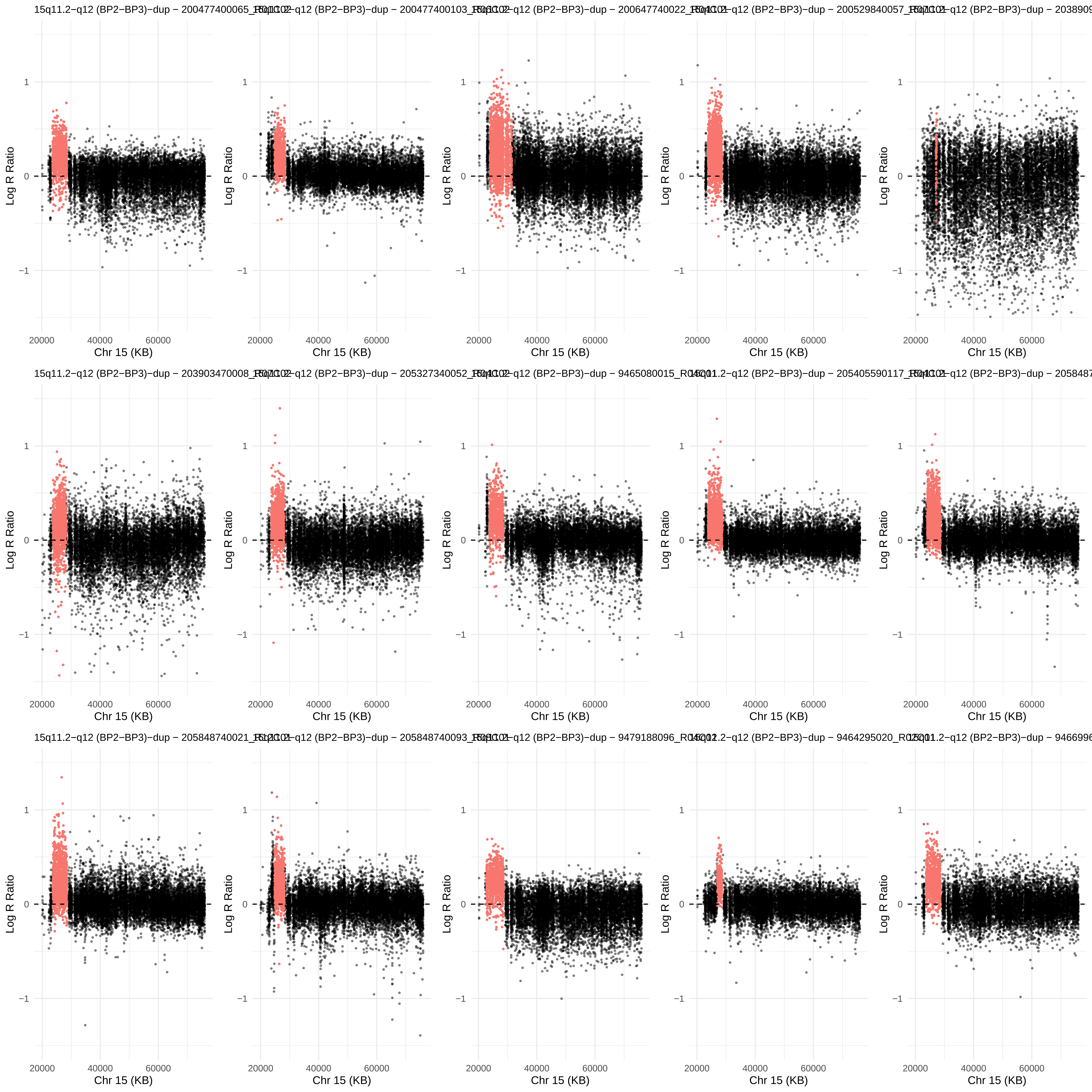
Log R Ratio
Chr 15 (KB)
20000
40000
60000
1
0
−1

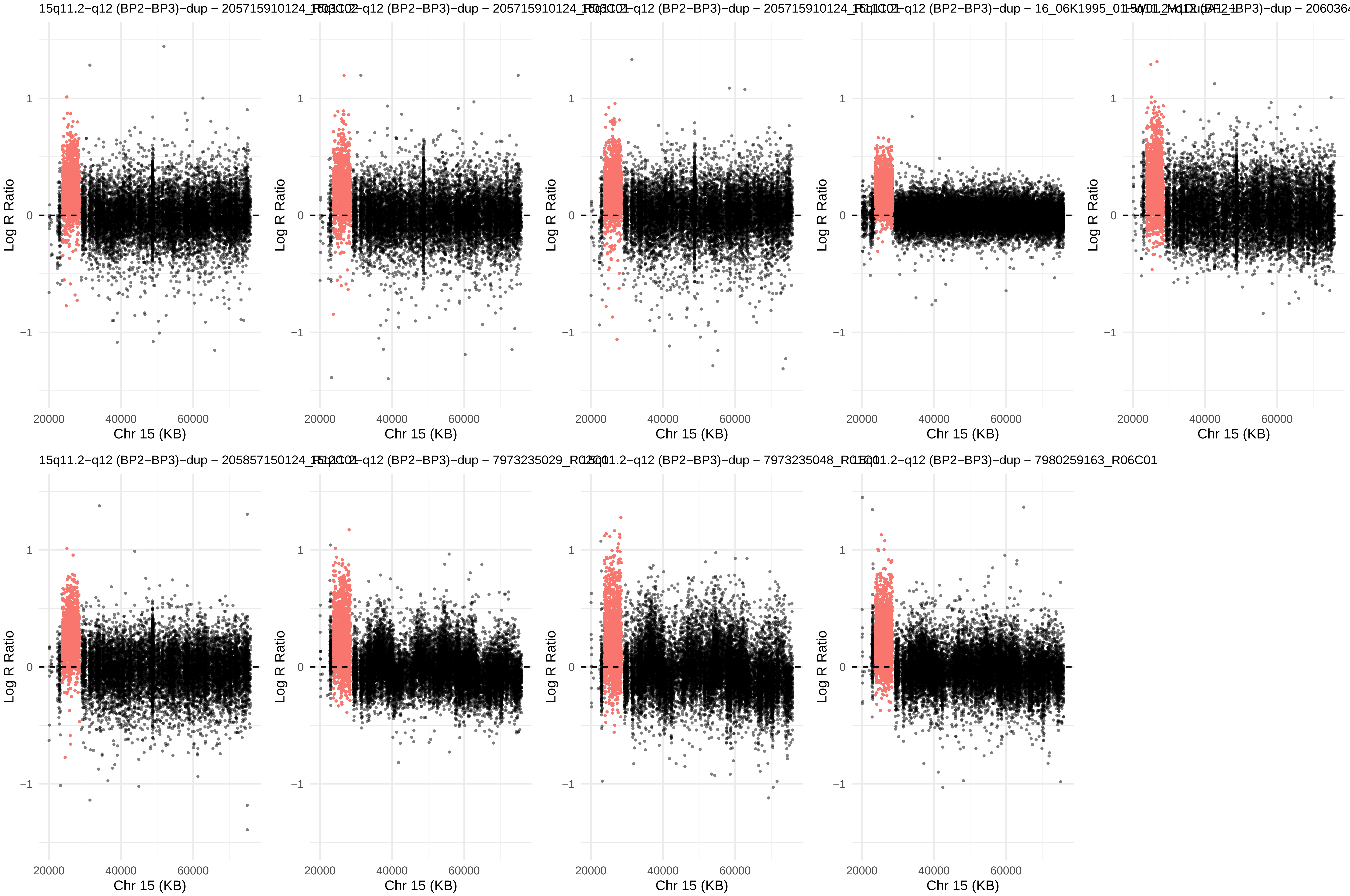
15q11.2−q12 (BP2−BP3)−dup − 205715910124_R
15q11.2−q12 (BP2−BP3)−dup − 205715910124_R
15q11.2−q12 (BP2−BP3)−dup − 205715910124_R
15q11.2−q12 (BP2−BP3)−dup − 16_06K1995_01
15q11.2−q12 (BP2−BP3)−dup − 206036
15q11.2−q12 (BP2−BP3)−dup − 205857150124_R
15q11.2−q12 (BP2−BP3)−dup − 7973235029_R
15q11.2−q12 (BP2−BP3)−dup − 7973235048_R
15q11.2−q12 (BP2−BP3)−dup − 7980259163_R06C01
Log R Ratio
Chr 15 (KB)
1
0
−1
20000
40000
60000

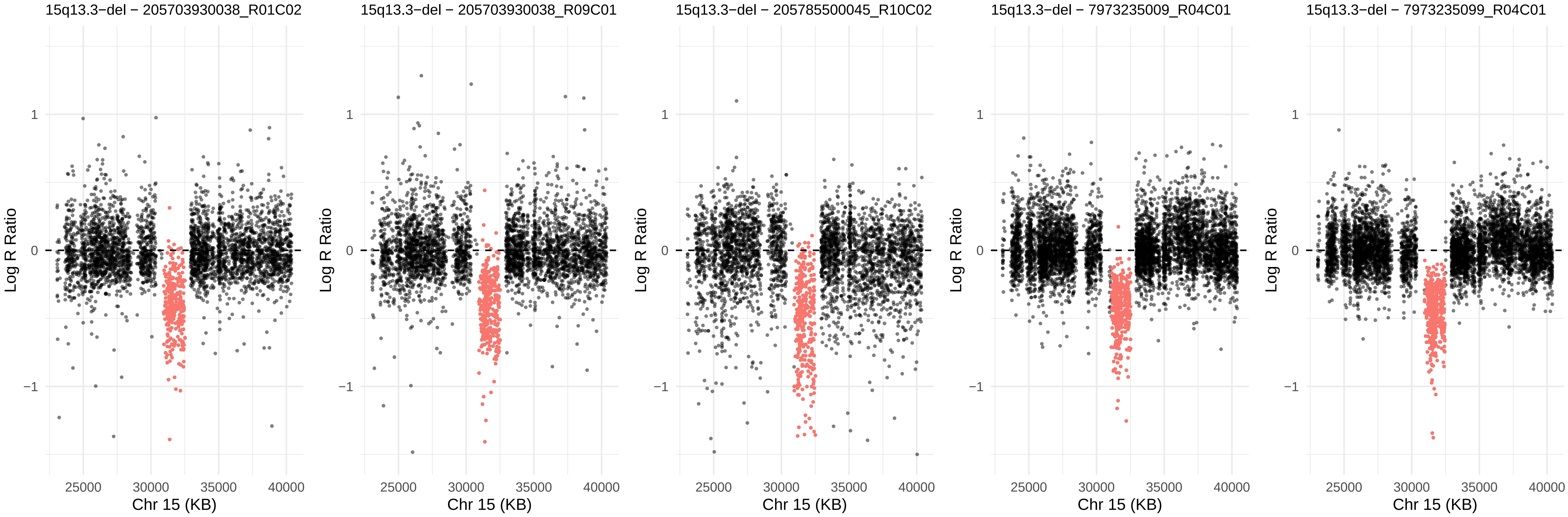
15q13.3−del − 205703930038_R01C02
15q13.3−del − 205703930038_R09C01
15q13.3−del − 205785500045_R10C02
15q13.3−del − 7973235009_R04C01
15q13.3−del − 7973235099_R04C01
Log R Ratio
Chr 15 (KB)
1
0
−1
25000
30000
35000
40000

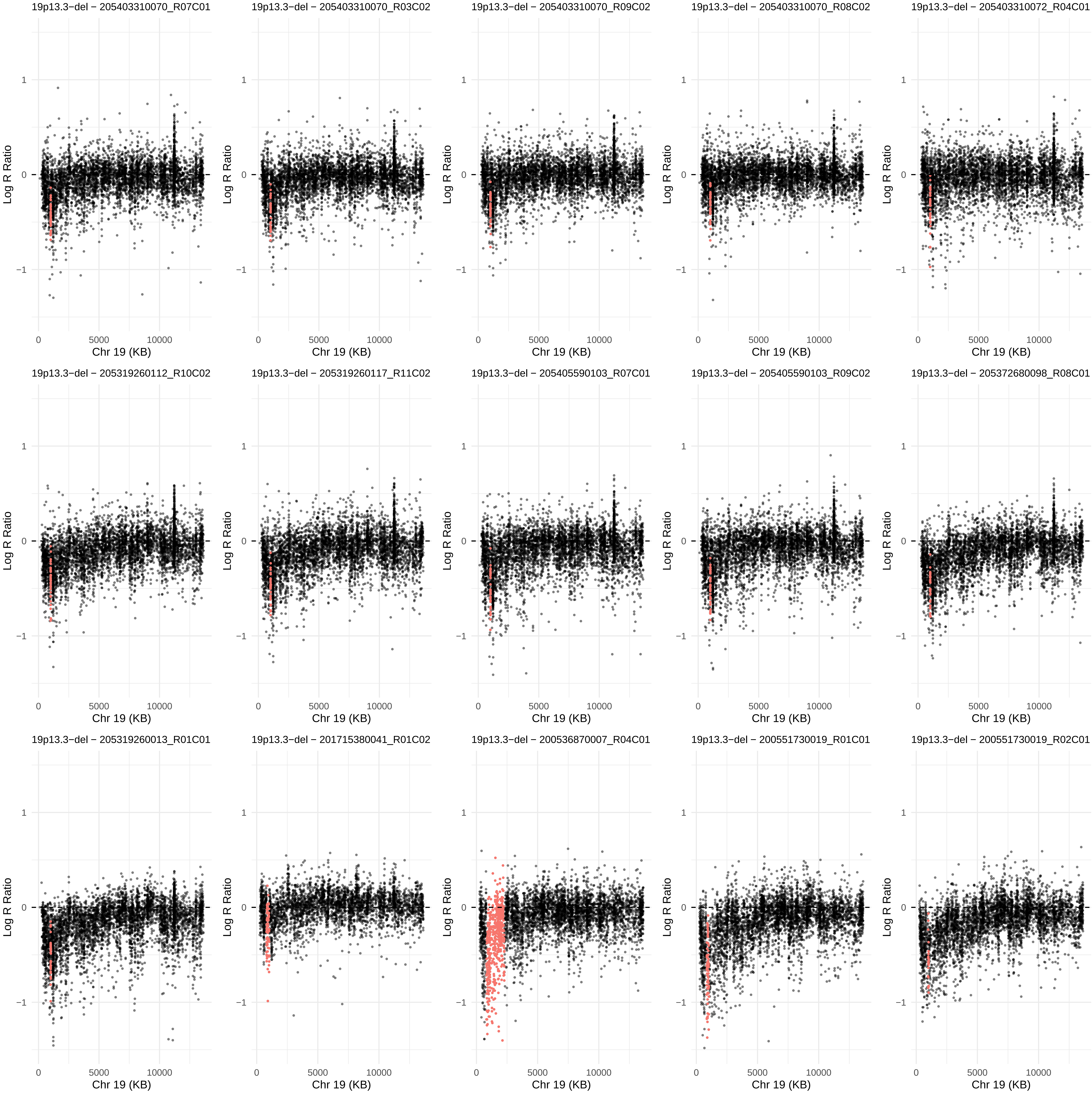
19p13.3−del − 205403310070_R07C01
19p13.3−del − 205403310070_R03C02
19p13.3−del − 205403310070_R09C02
19p13.3−del − 205403310070_R08C02
19p13.3−del − 205403310072_R04C01
19p13.3−del − 205319260112_R10C02
19p13.3−del − 205319260117_R11C02
19p13.3−del − 205405590103_R07C01
19p13.3−del − 205405590103_R09C02
19p13.3−del − 205372680098_R08C01
19p13.3−del − 205319260013_R01C01
19p13.3−del − 201715380041_R01C02
19p13.3−del − 200536870007_R04C01
19p13.3−del − 200551730019_R01C01
19p13.3−del − 200551730019_R02C01
Log R Ratio
1
0
−1
Chr 19 (KB)
0
5000
10000

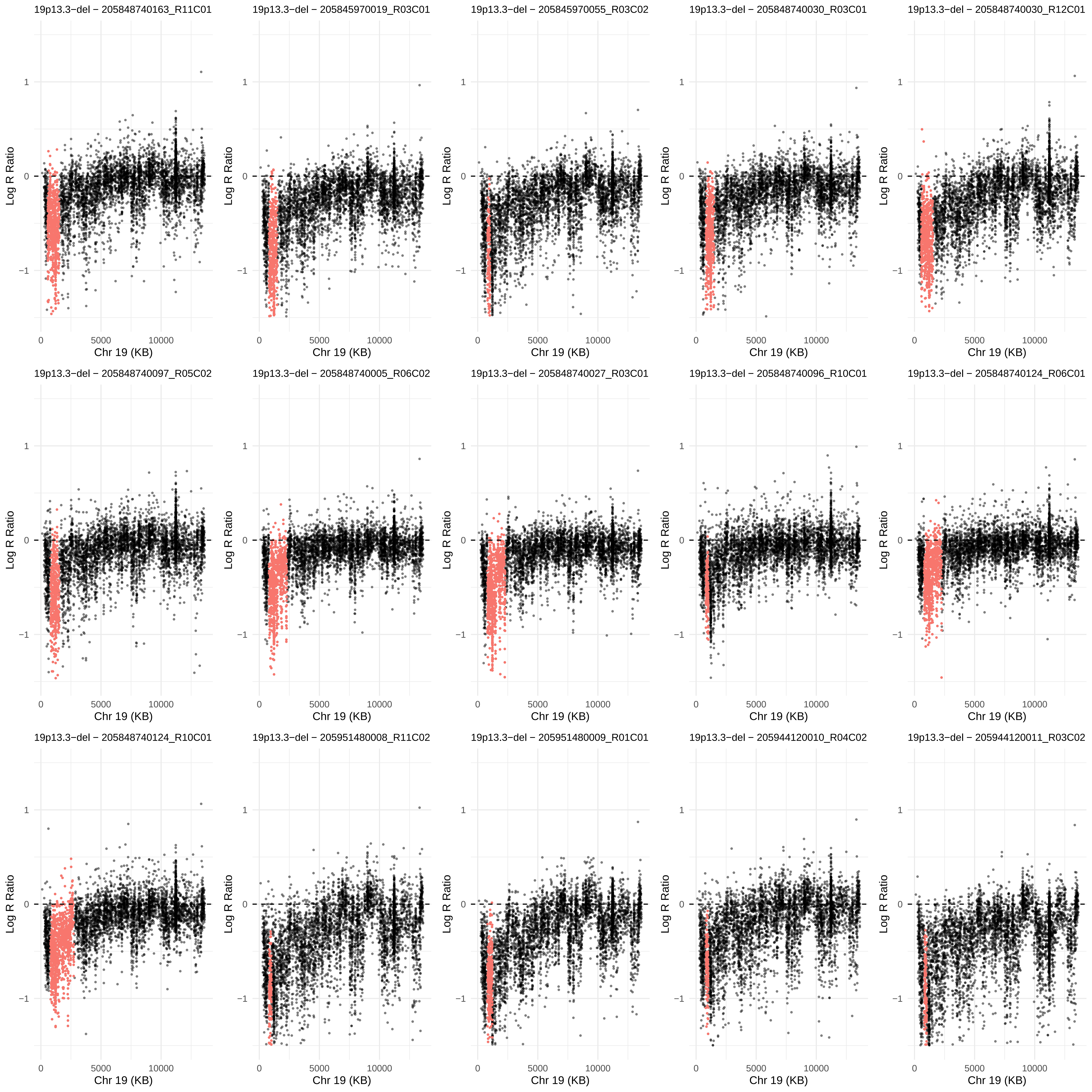
19p13.3−del − 205848740163_R11C01
19p13.3−del − 205845970019_R03C01
19p13.3−del − 205845970055_R03C02
19p13.3−del − 205848740030_R03C01
19p13.3−del − 205848740030_R12C01
19p13.3−del − 205848740097_R05C02
19p13.3−del − 205848740005_R06C02
19p13.3−del − 205848740027_R03C01
19p13.3−del − 205848740096_R10C01
19p13.3−del − 205848740124_R06C01
19p13.3−del − 205848740124_R10C01
19p13.3−del − 205951480008_R11C02
19p13.3−del − 205951480009_R01C01
19p13.3−del − 205944120010_R04C02
19p13.3−del − 205944120011_R03C02
Log R Ratio
1
0
−1
0
5000
10000
Chr 19 (KB)

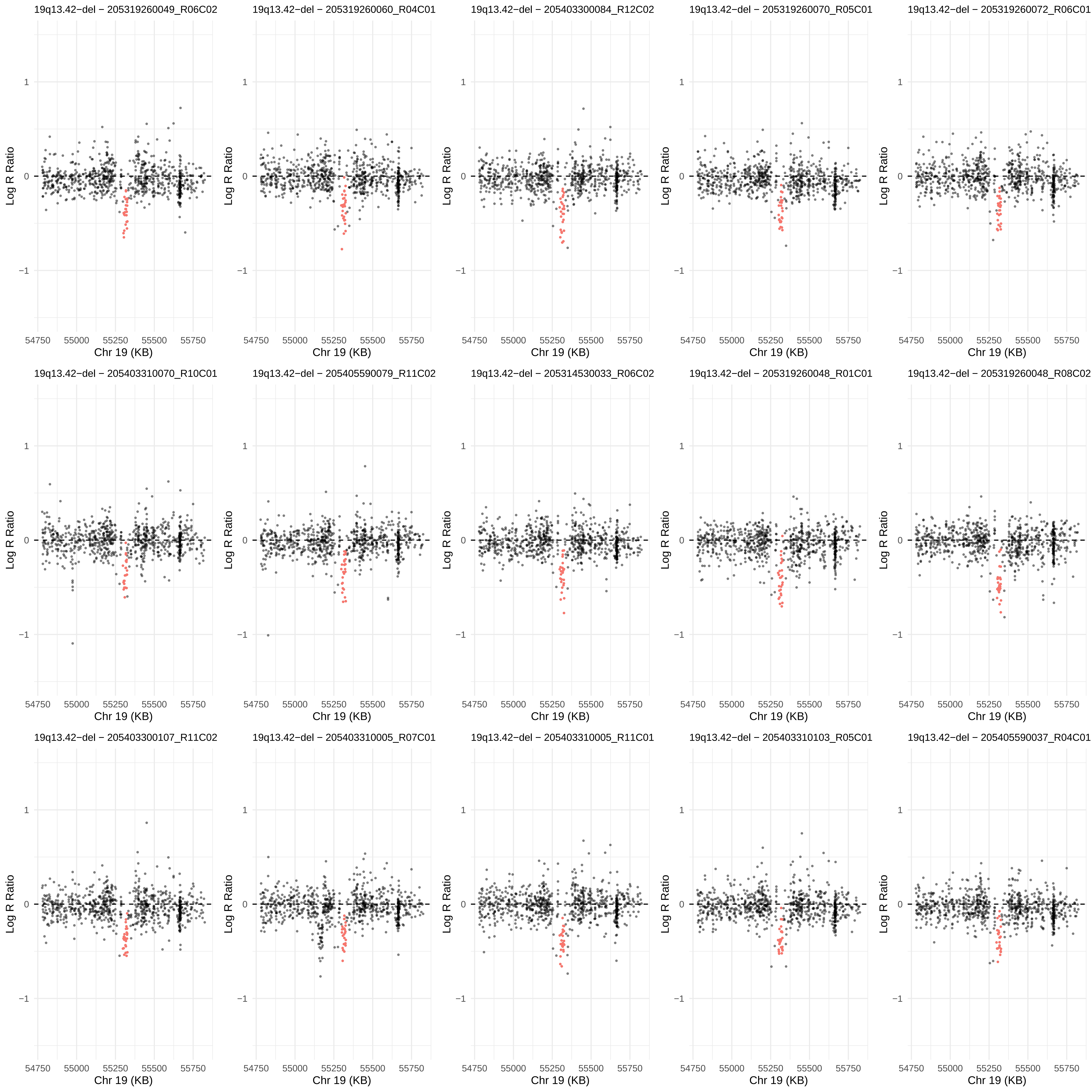
19q13.42−del − 205319260049_R06C02
19q13.42−del − 205319260060_R04C01
19q13.42−del − 205403300084_R12C02
19q13.42−del − 205319260070_R05C01
19q13.42−del − 205319260072_R06C01
19q13.42−del − 205403310070_R10C01
19q13.42−del − 205405590079_R11C02
19q13.42−del − 205314530033_R06C02
19q13.42−del − 205319260048_R01C01
19q13.42−del − 205319260048_R08C02
19q13.42−del − 205403300107_R11C02
19q13.42−del − 205403310005_R07C01
19q13.42−del − 205403310005_R11C01
19q13.42−del − 205403310103_R05C01
19q13.42−del − 205405590037_R04C01
Log R Ratio
1
0
−1
54750
55000
55250
55500
55750
Chr 19 (KB)

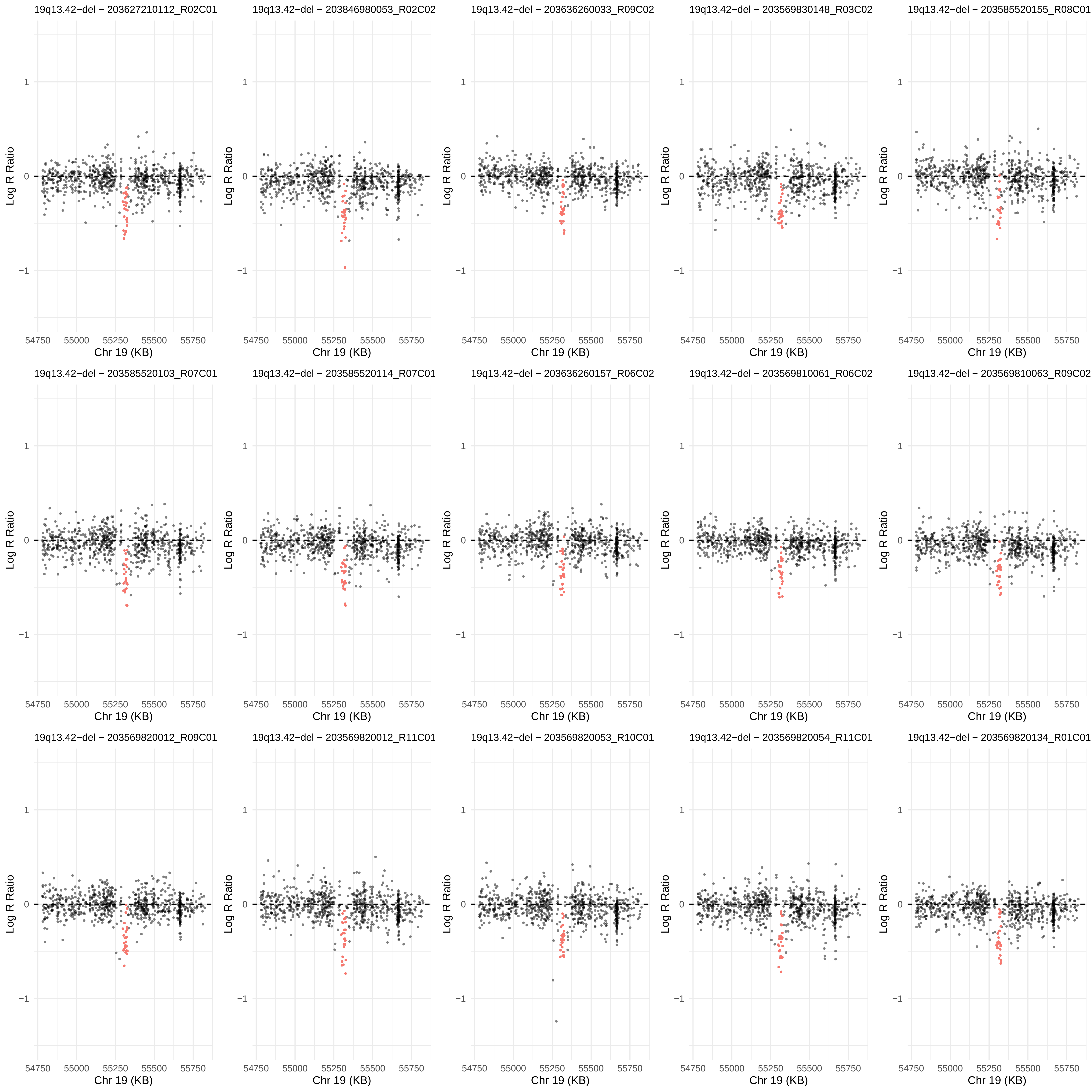
19q13.42−del − 203627210112_R02C01
19q13.42−del − 203846980053_R02C02
19q13.42−del − 203636260033_R09C02
19q13.42−del − 203569830148_R03C02
19q13.42−del − 203585520155_R08C01
19q13.42−del − 203585520103_R07C01
19q13.42−del − 203585520114_R07C01
19q13.42−del − 203636260157_R06C02
19q13.42−del − 203569810061_R06C02
19q13.42−del − 203569810063_R09C02
19q13.42−del − 203569820012_R09C01
19q13.42−del − 203569820012_R11C01
19q13.42−del − 203569820053_R10C01
19q13.42−del − 203569820054_R11C01
19q13.42−del − 203569820134_R01C01
Log R Ratio
1
0
−1
54750
55000
55250
55500
55750
Chr 19 (KB)

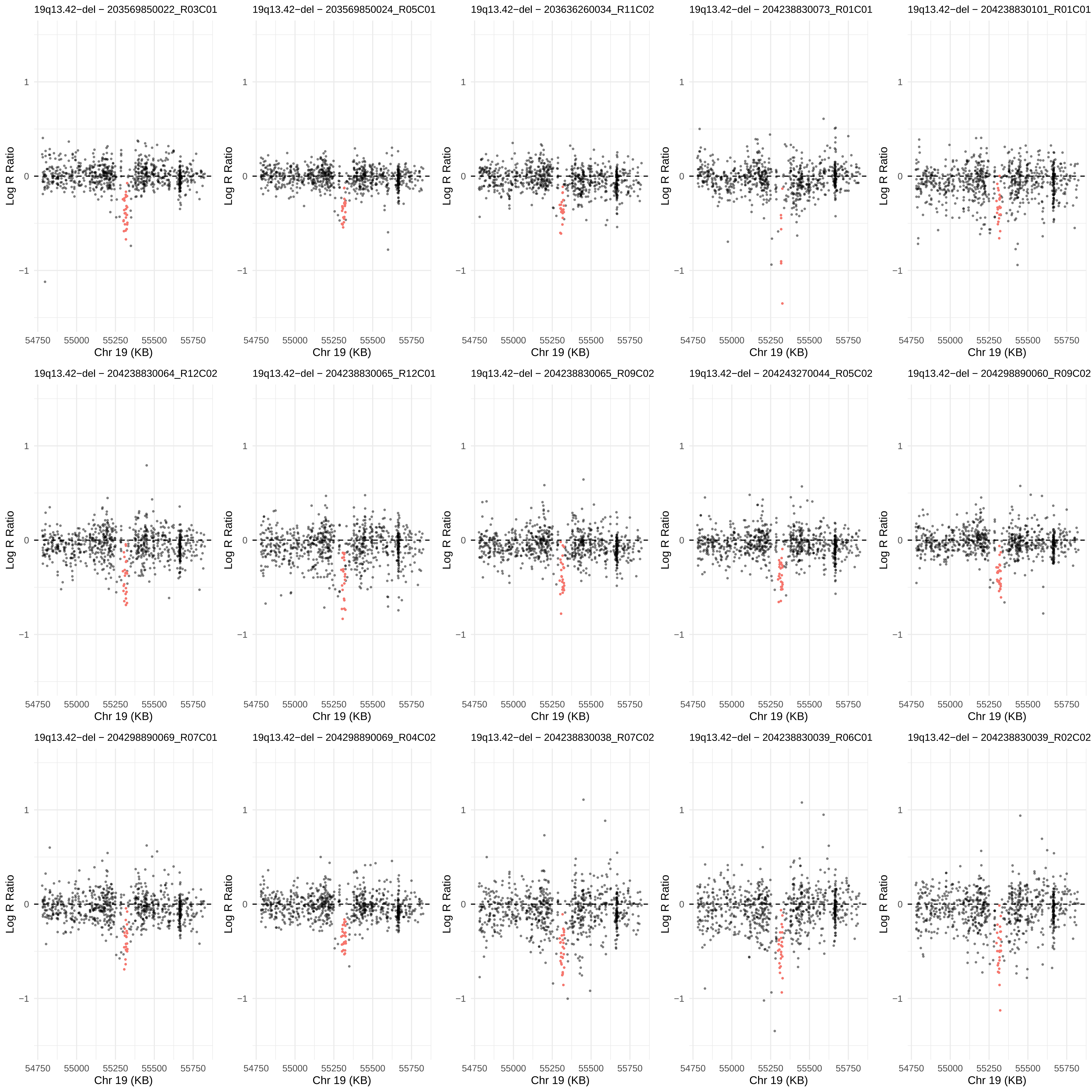

19q13.42−del − 203569850022_R03C01
19q13.42−del − 203569850024_R05C01
19q13.42−del − 203636260034_R11C02
19q13.42−del − 204238830073_R01C01
19q13.42−del − 204238830101_R01C01
19q13.42−del − 204238830064_R12C02
19q13.42−del − 204238830065_R12C01
19q13.42−del − 204238830065_R09C02
19q13.42−del − 204243270044_R05C02
19q13.42−del − 204298890060_R09C02
19q13.42−del − 204298890069_R07C01
19q13.42−del − 204298890069_R04C02
19q13.42−del − 204238830038_R07C02
19q13.42−del − 204238830039_R06C01
19q13.42−del − 204238830039_R02C02
Log R Ratio
1
0
−1
54750
55000
55250
55500
55750
Chr 19 (KB)

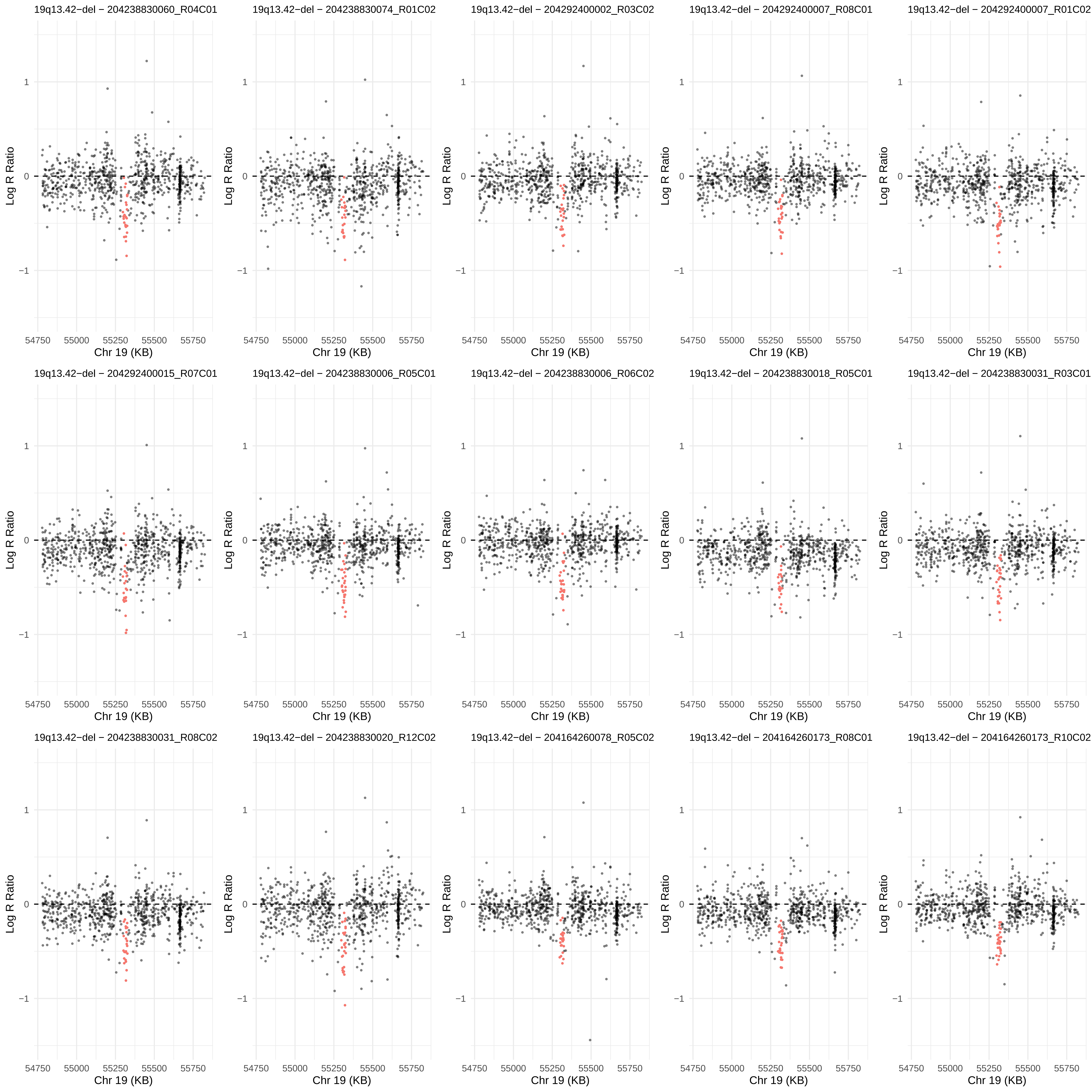
19q13.42−del − 204238830060_R04C01
19q13.42−del − 204238830074_R01C02
19q13.42−del − 204292400002_R03C02
19q13.42−del − 204292400007_R08C01
19q13.42−del − 204292400007_R01C02
19q13.42−del − 204292400015_R07C01
19q13.42−del − 204238830006_R05C01
19q13.42−del − 204238830006_R06C02
19q13.42−del − 204238830018_R05C01
19q13.42−del − 204238830031_R03C01
19q13.42−del − 204238830031_R08C02
19q13.42−del − 204238830020_R12C02
19q13.42−del − 204164260078_R05C02
19q13.42−del − 204164260173_R08C01
19q13.42−del − 204164260173_R10C02
Log R Ratio
1
0
−1
54750
55000
55250
55500
55750
Chr 19 (KB)

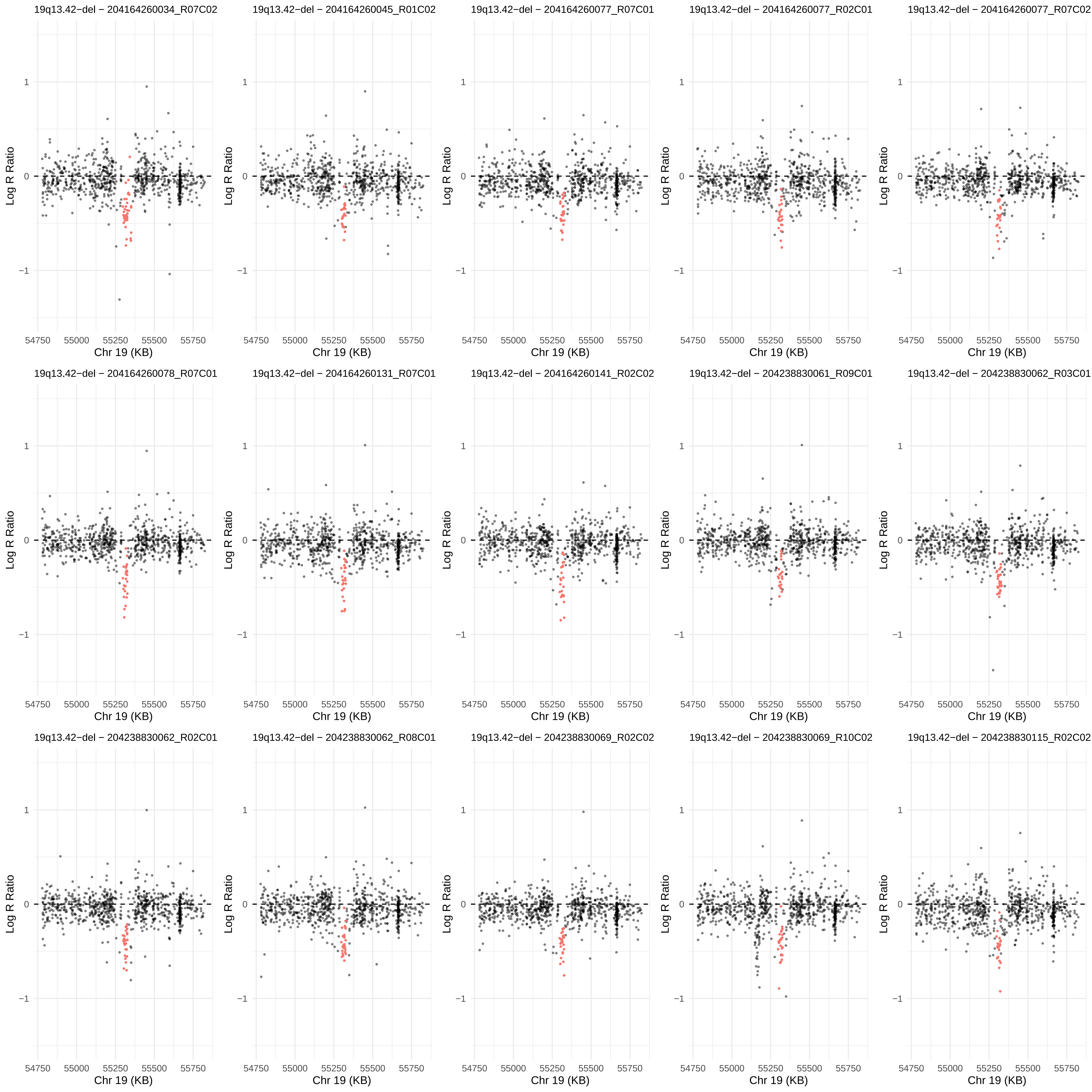
19q13.42−del − 204164260034_R07C02
19q13.42−del − 204164260045_R01C02
19q13.42−del − 204164260077_R07C01
19q13.42−del − 204164260077_R02C01
19q13.42−del − 204164260077_R07C02
19q13.42−del − 204164260078_R07C01
19q13.42−del − 204164260131_R07C01
19q13.42−del − 204164260141_R02C02
19q13.42−del − 204238830061_R09C01
19q13.42−del − 204238830062_R03C01
19q13.42−del − 204238830062_R02C01
19q13.42−del − 204238830062_R08C01
19q13.42−del − 204238830069_R02C02
19q13.42−del − 204238830069_R10C02
19q13.42−del − 204238830115_R02C02
Log R Ratio
1
0
−1
54750
55000
55250
55500
55750
Chr 19 (KB)

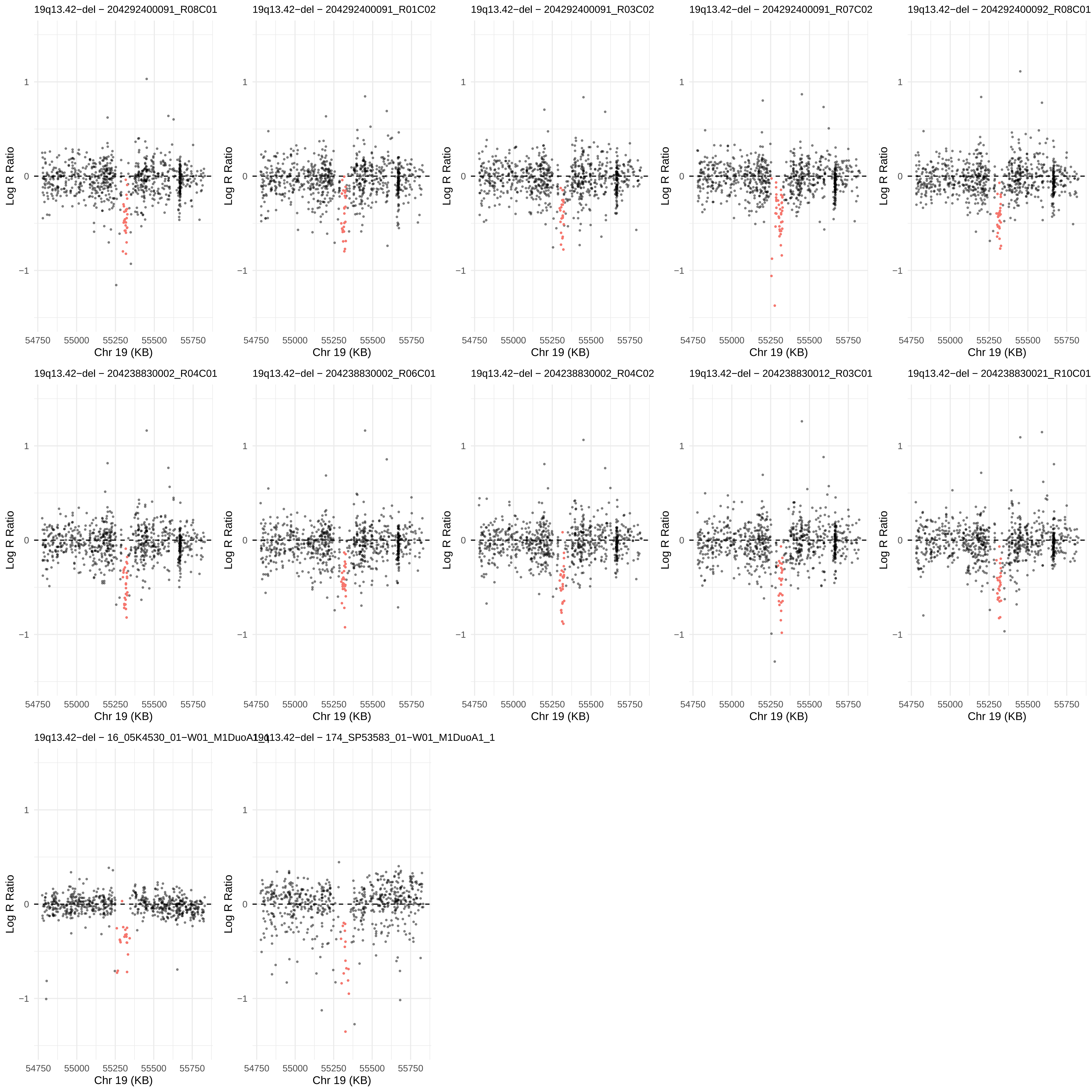

19q13.42−del − 204292400091_R08C01
19q13.42−del − 204292400091_R01C02
19q13.42−del − 204292400091_R03C02
19q13.42−del − 204292400091_R07C02
19q13.42−del − 204292400092_R08C01
19q13.42−del − 204238830002_R04C01
19q13.42−del − 204238830002_R06C01
19q13.42−del − 204238830002_R04C02
19q13.42−del − 204238830012_R03C01
19q13.42−del − 204238830021_R10C01
19q13.42−del − 16_05K4530_01−W01_M1DuoA1_1
19q13.42−del − 174_SP53583_01−W01_M1DuoA1_1
Log R Ratio
1
0
−1
54750
55000
55250
55500
55750
Chr 19 (KB)
