# Supplementary material for: Contribution of copy number variants to schizophrenia in East Asian populations
Source: Nat Genet. 2026 Sep 11;58(10):2514–24. doi: 10.1038/s41588-026-02732-6 (PMC13649597; doi:10.1038/s41588-026-02732-6)

09C91567
chr15:23393199-23762631

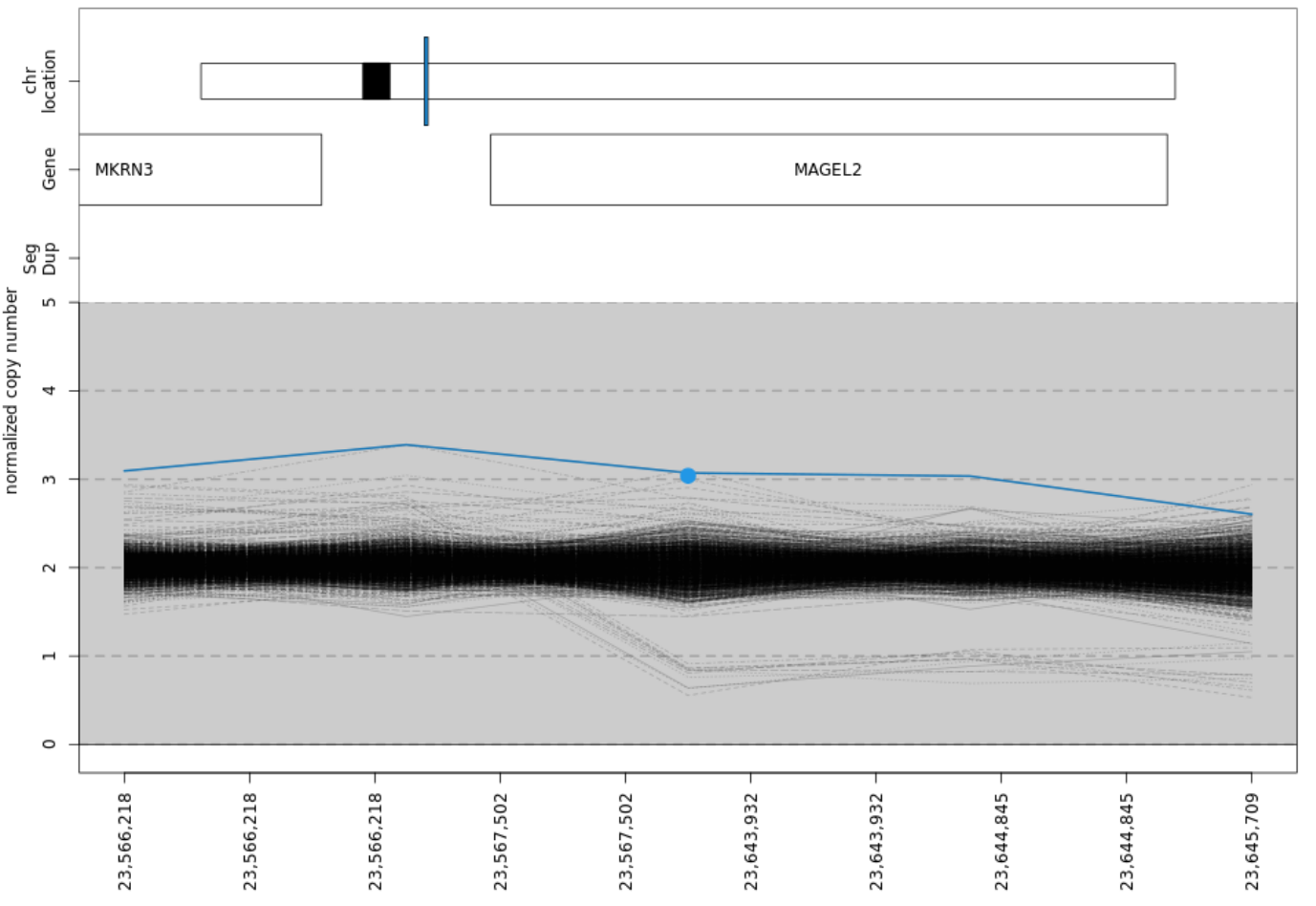

09C91567
chr15:23393199-23762631

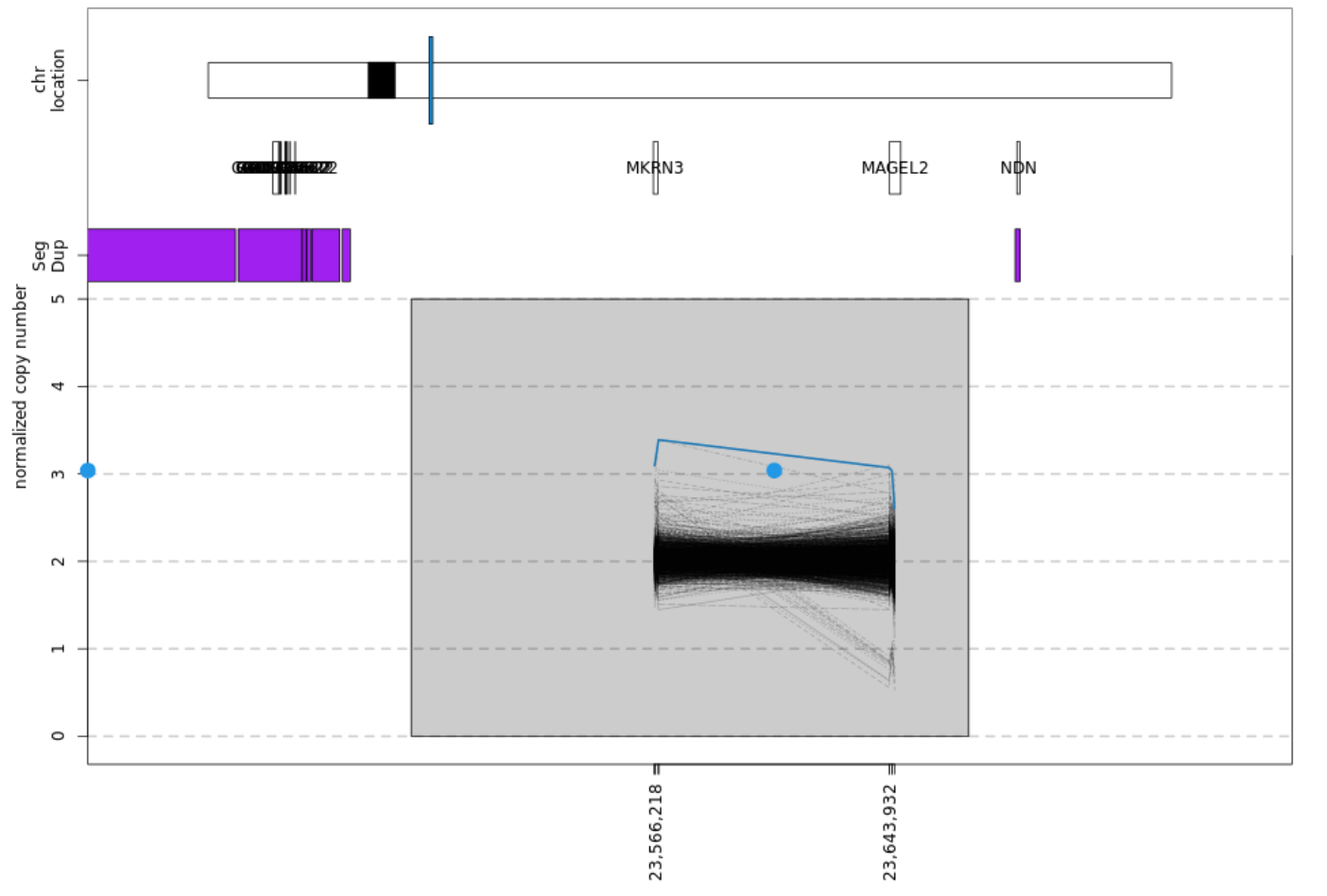

09C96516
chr15:18980693-31436008

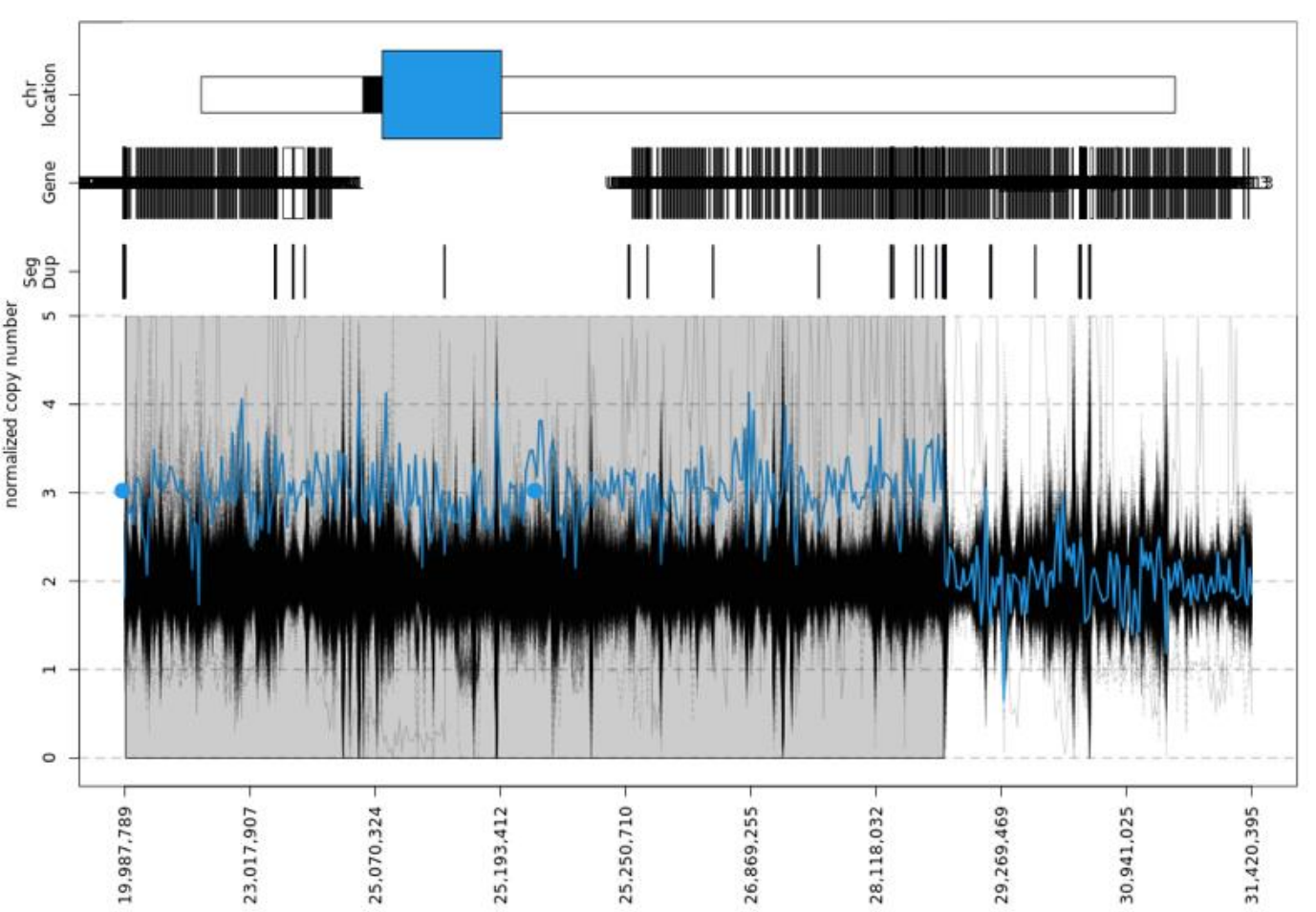

09C96516
chr15:18980693-31436008

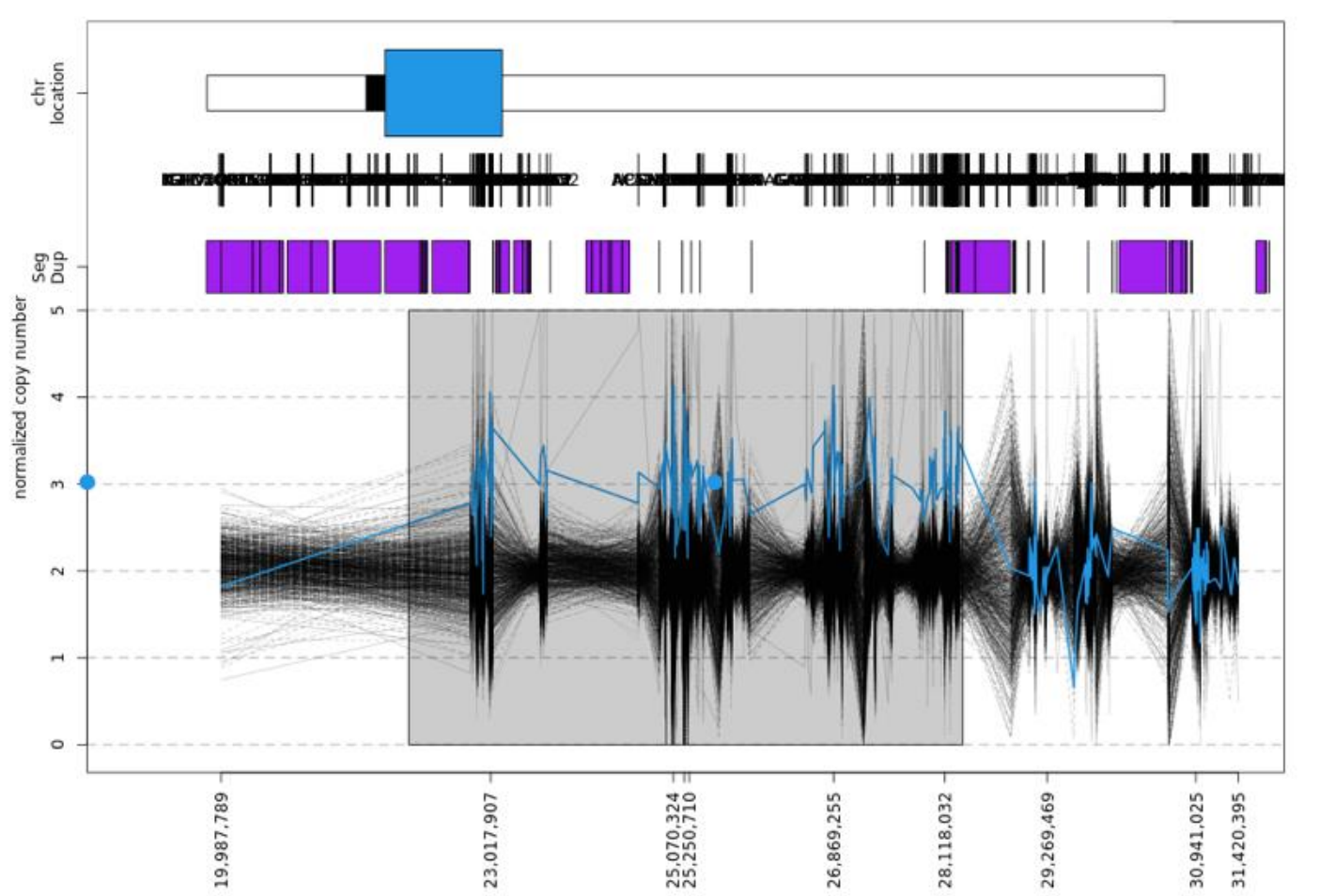

10C107768
chr15:25981739-29052802

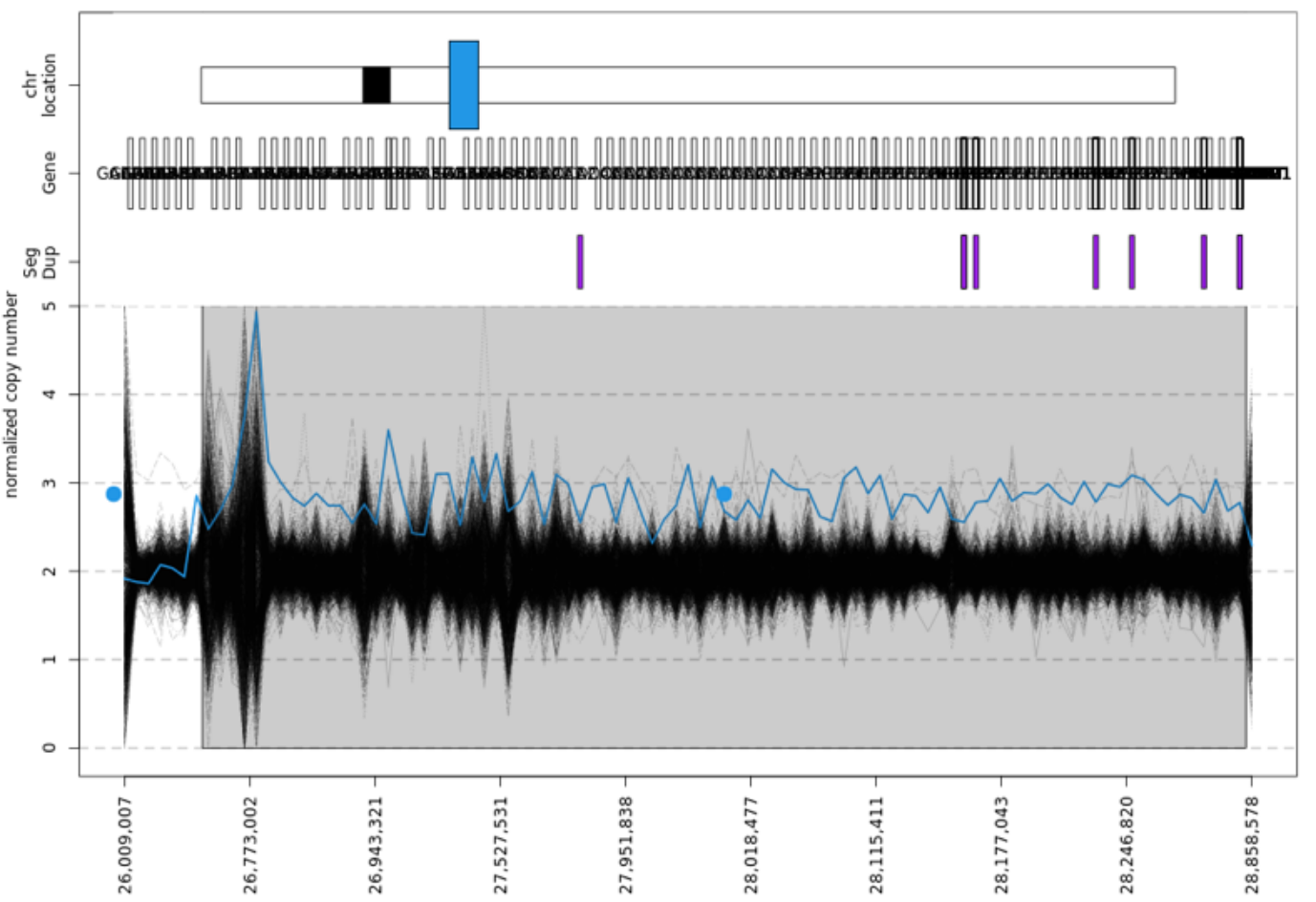

10C107768
chr15:25981739-29052802

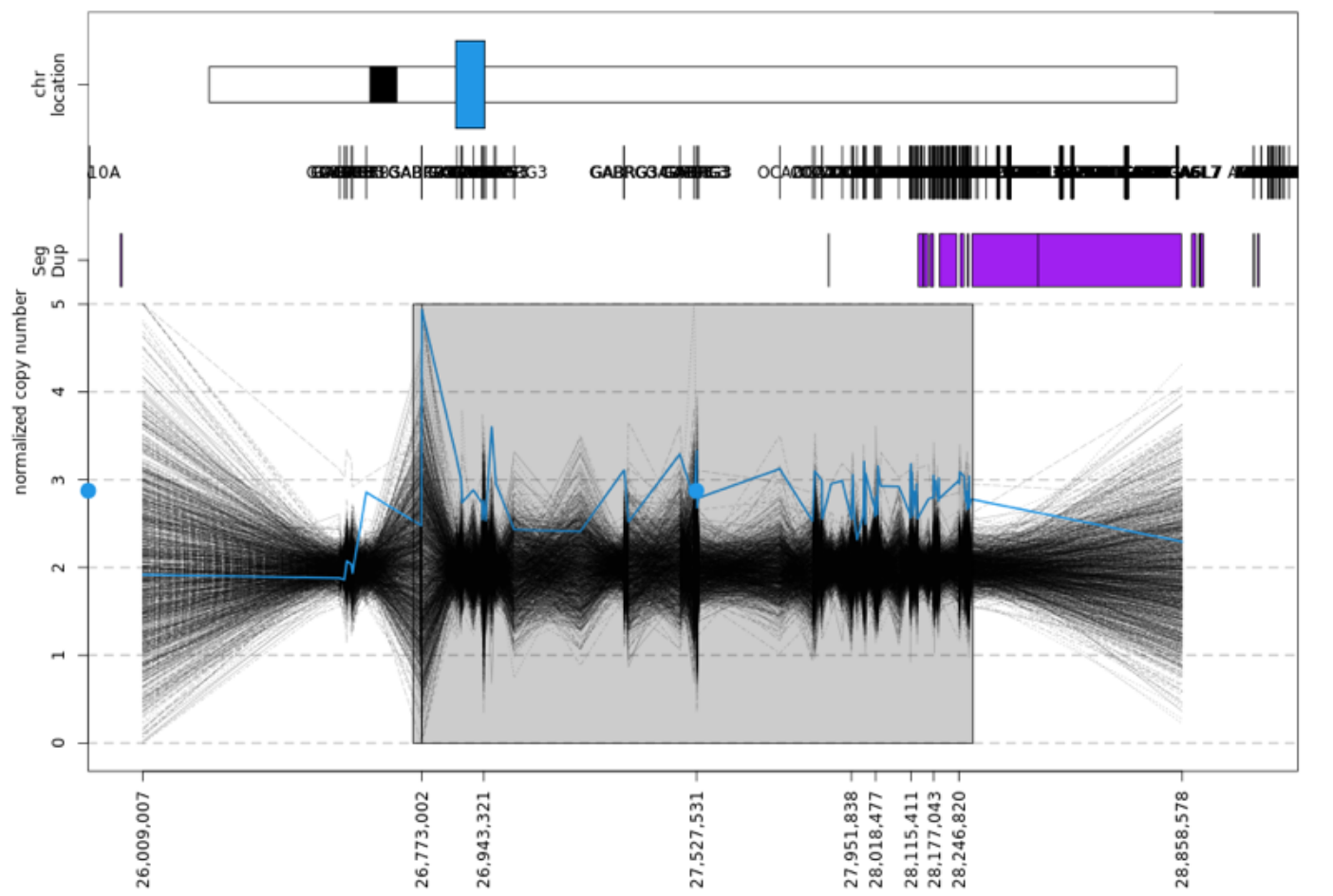

10C113089
chr15:21015436-30708236

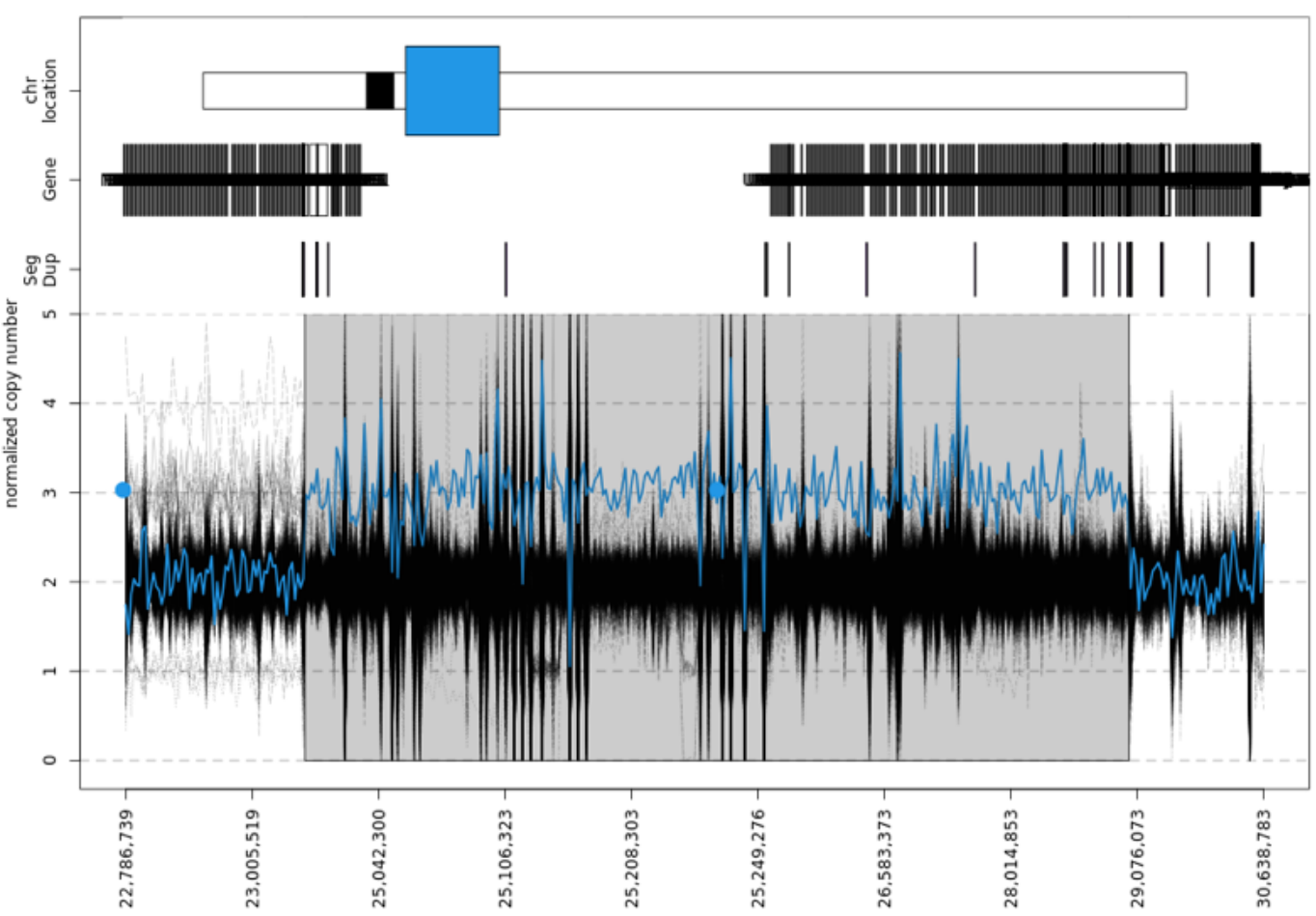

10C113089
chr15:21015436-30708236

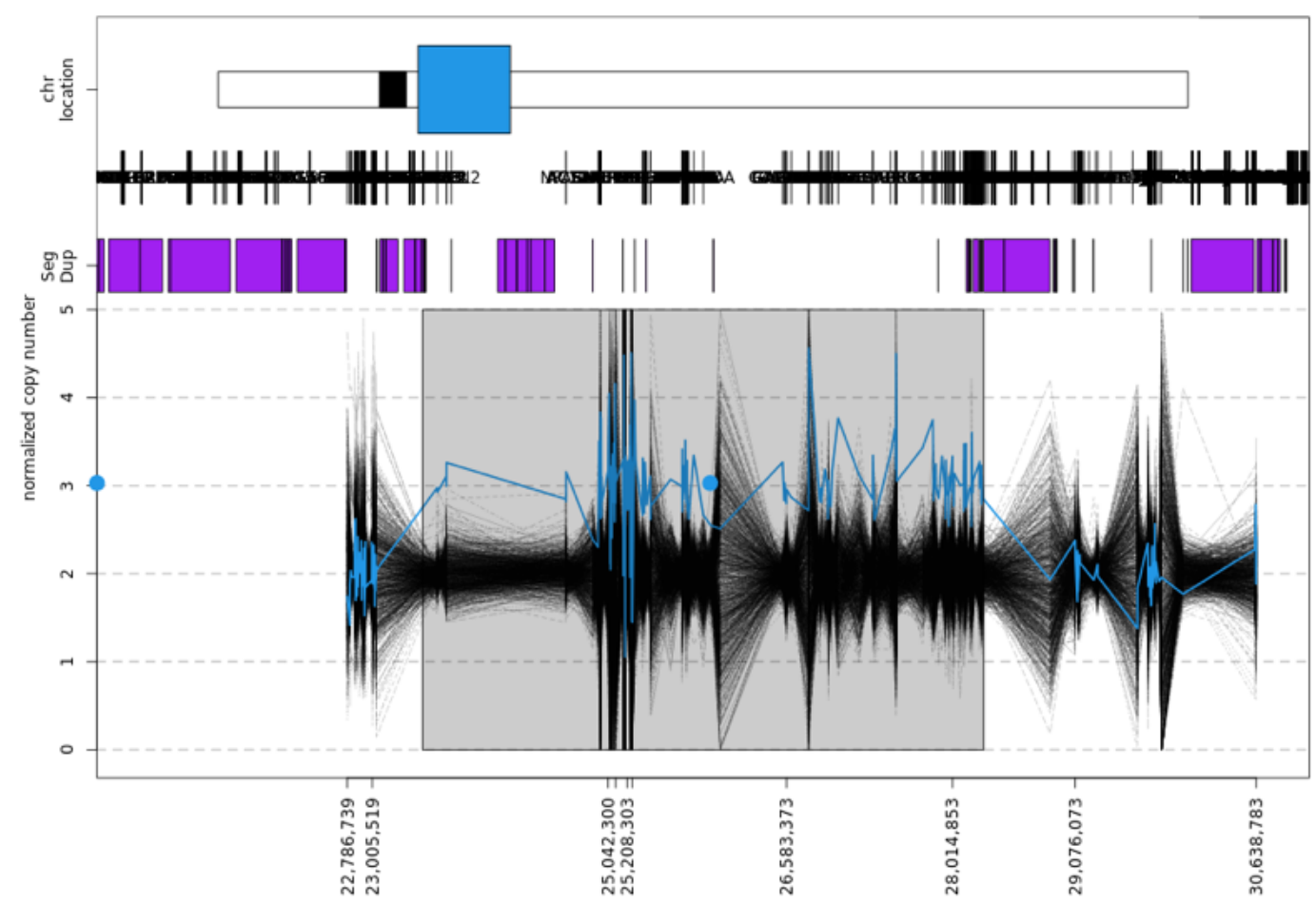

11C120651
chr15:19998505-31096737

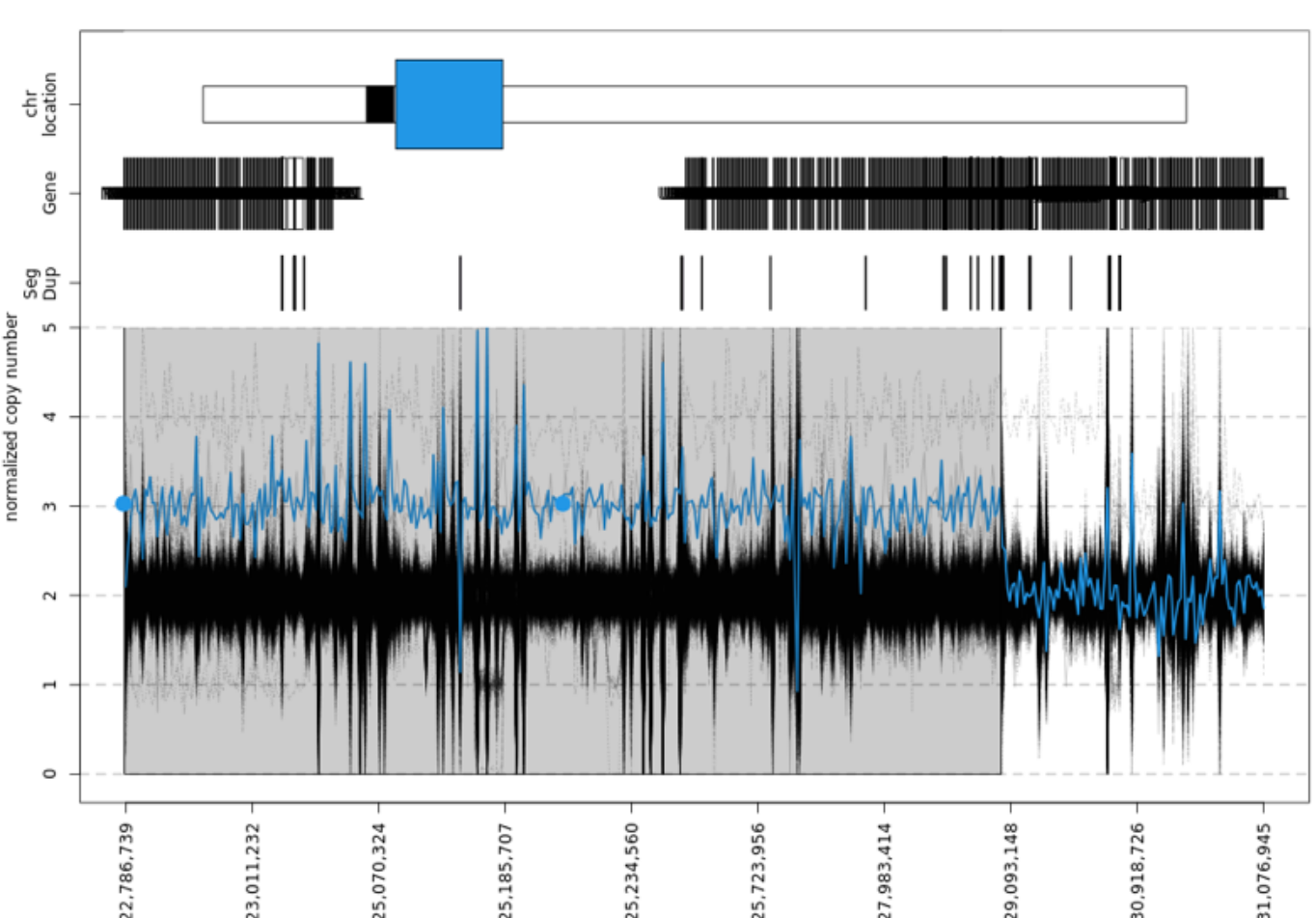

11C120651
chr15:19998505-31096737

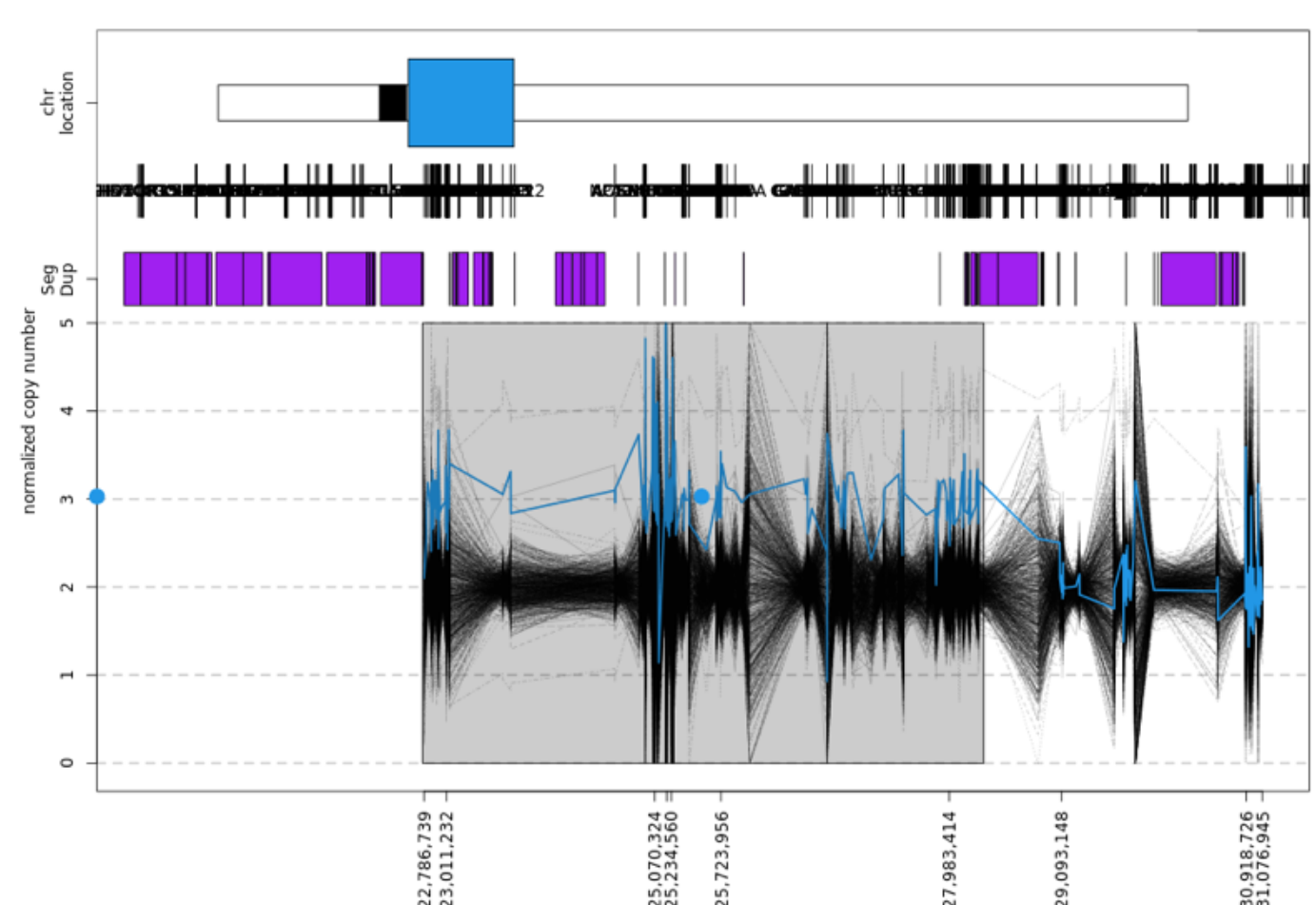

10C113087
chr15:21035825-30701440

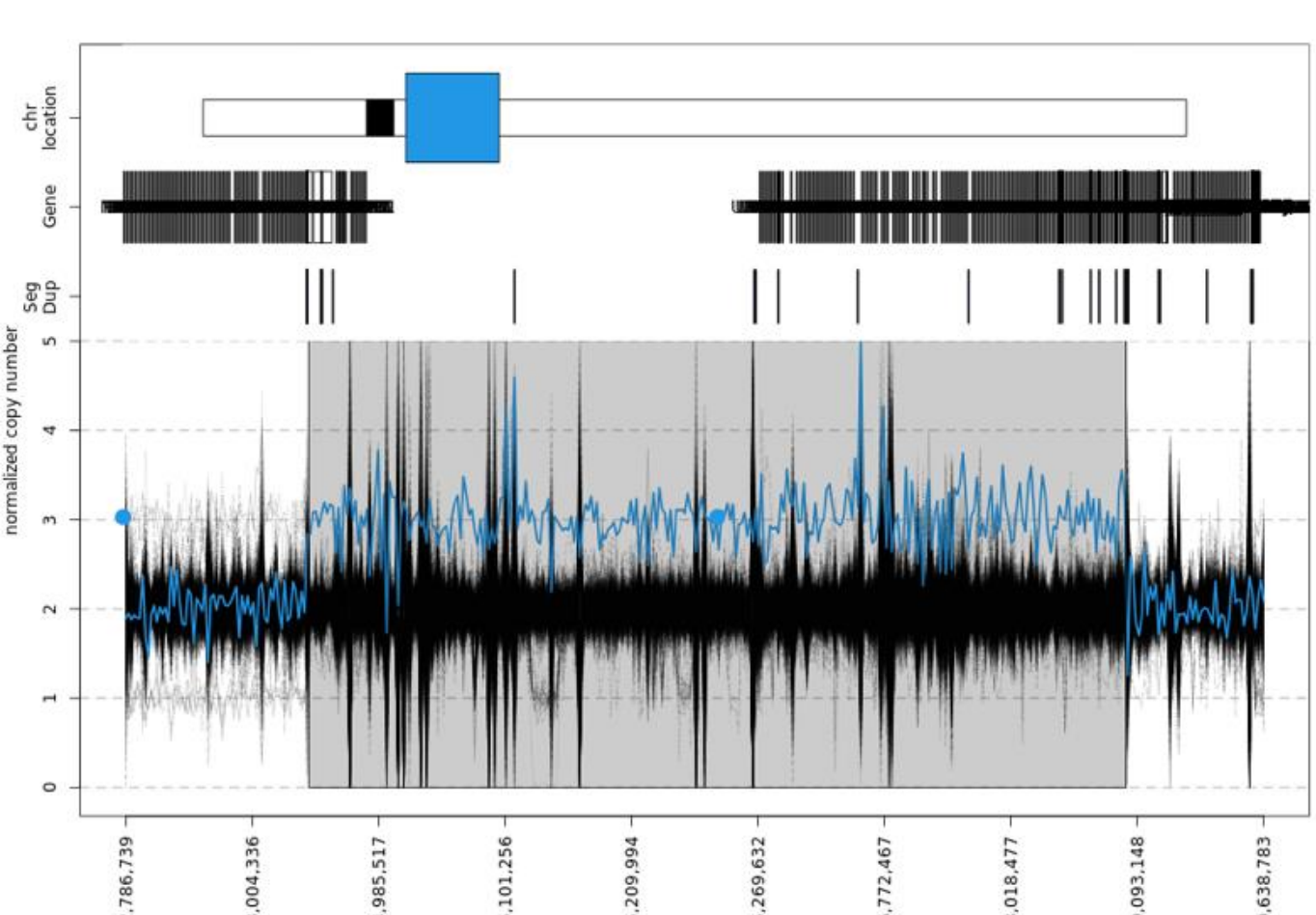

10C113087
chr15:21035825-30701440

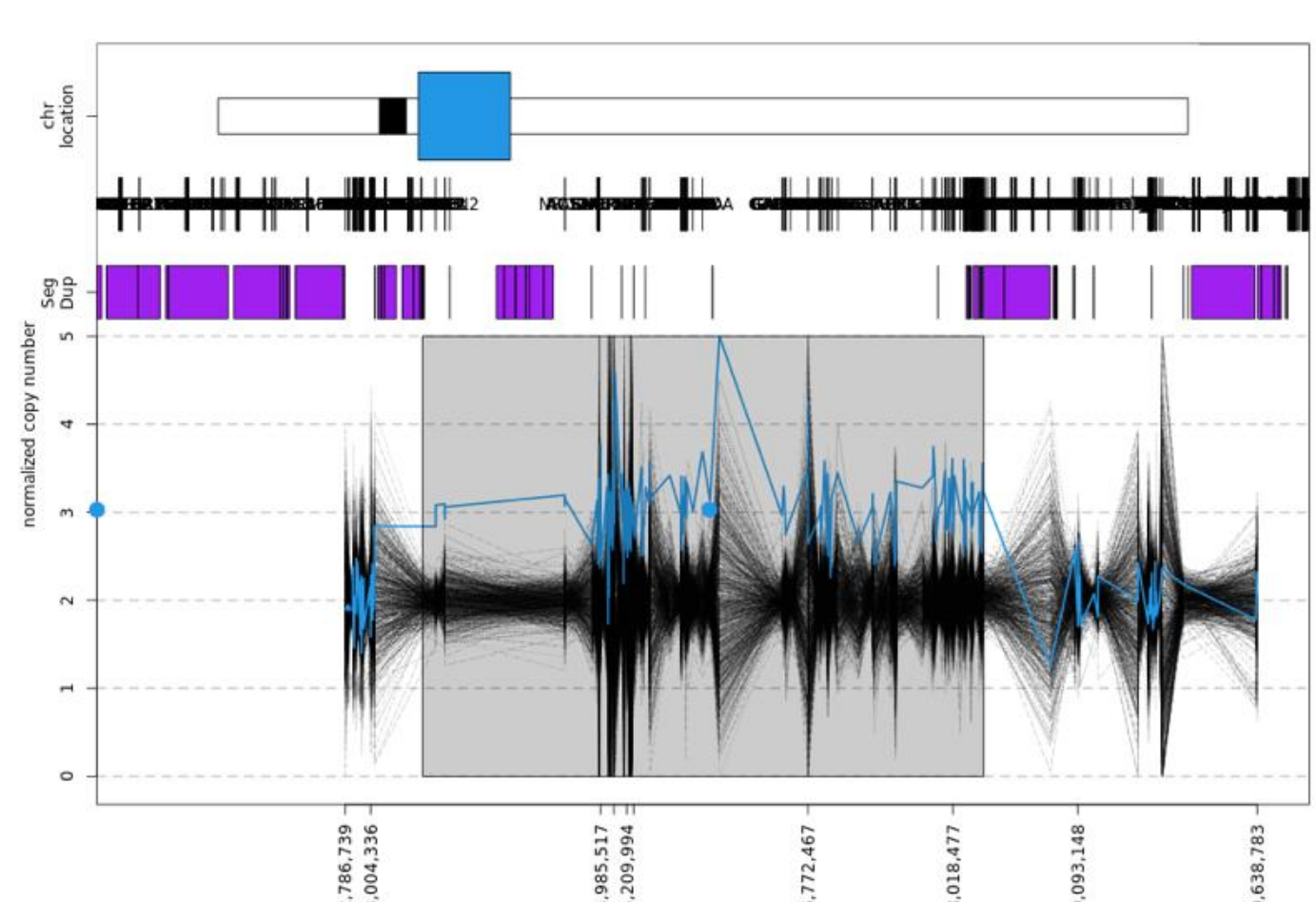

JP-KOB-S-00072
chr15:26644267-27049215

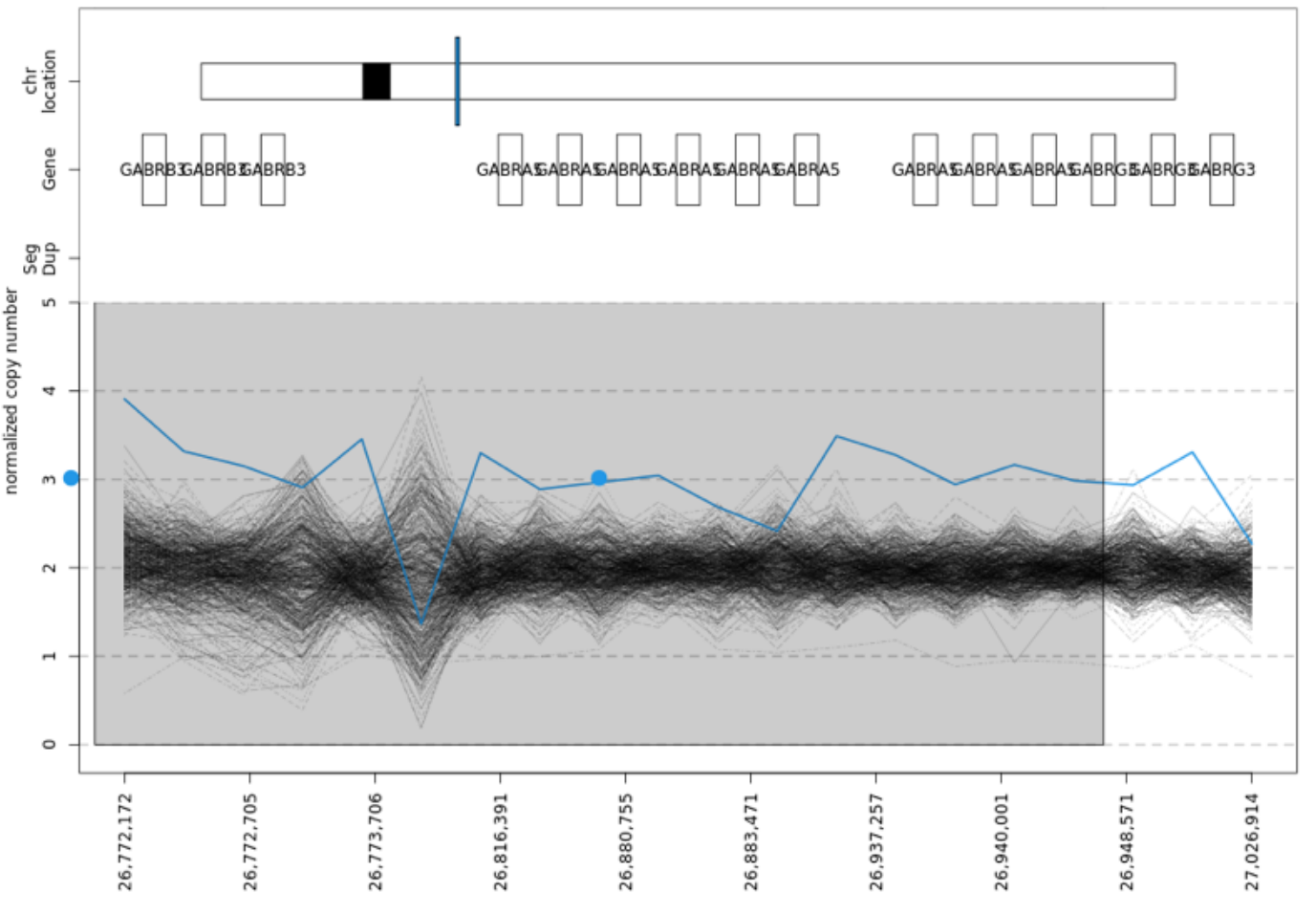

JP-KOB-S-00072
chr15:26644267-27049215

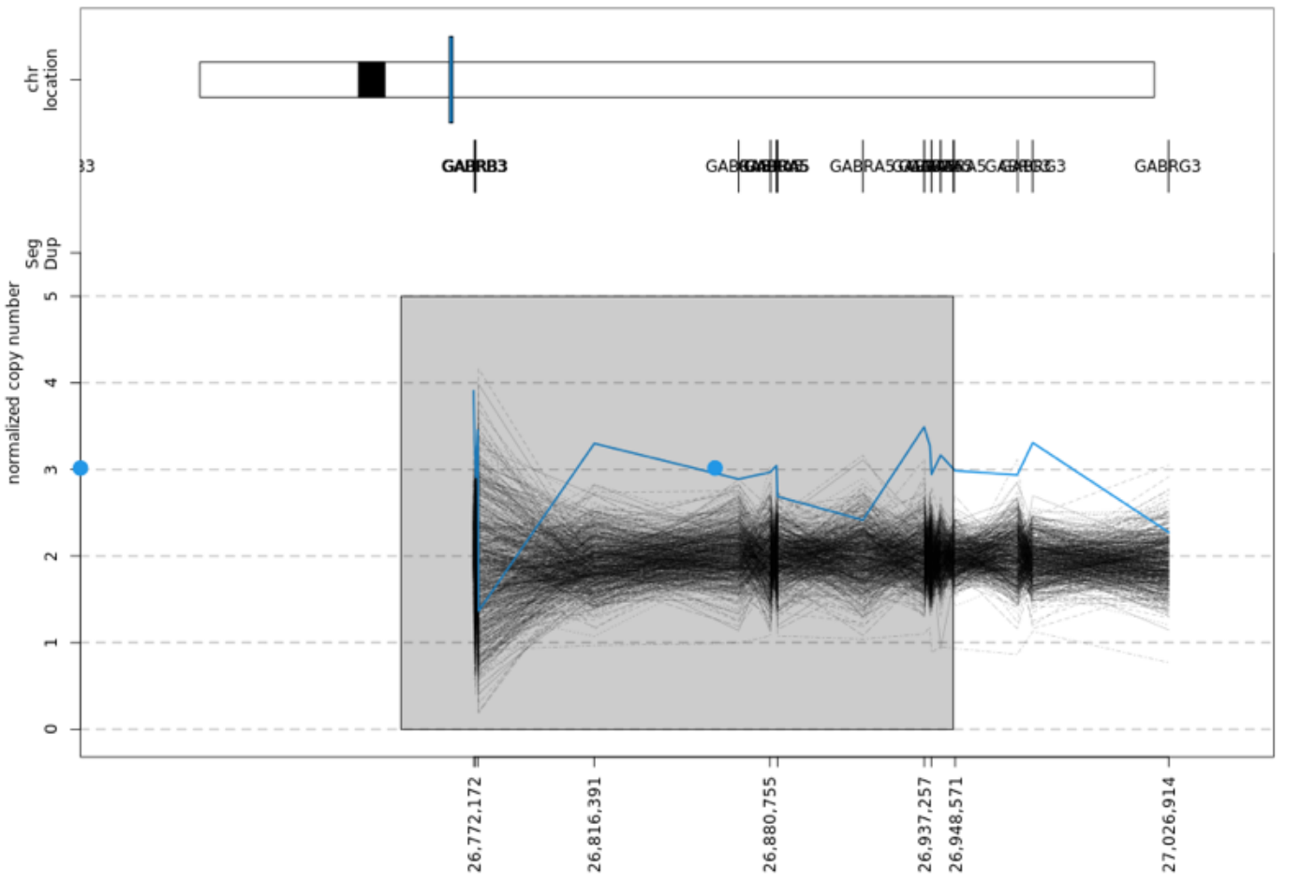

JP-KOB-S-00176
chr15:23235438-24312330

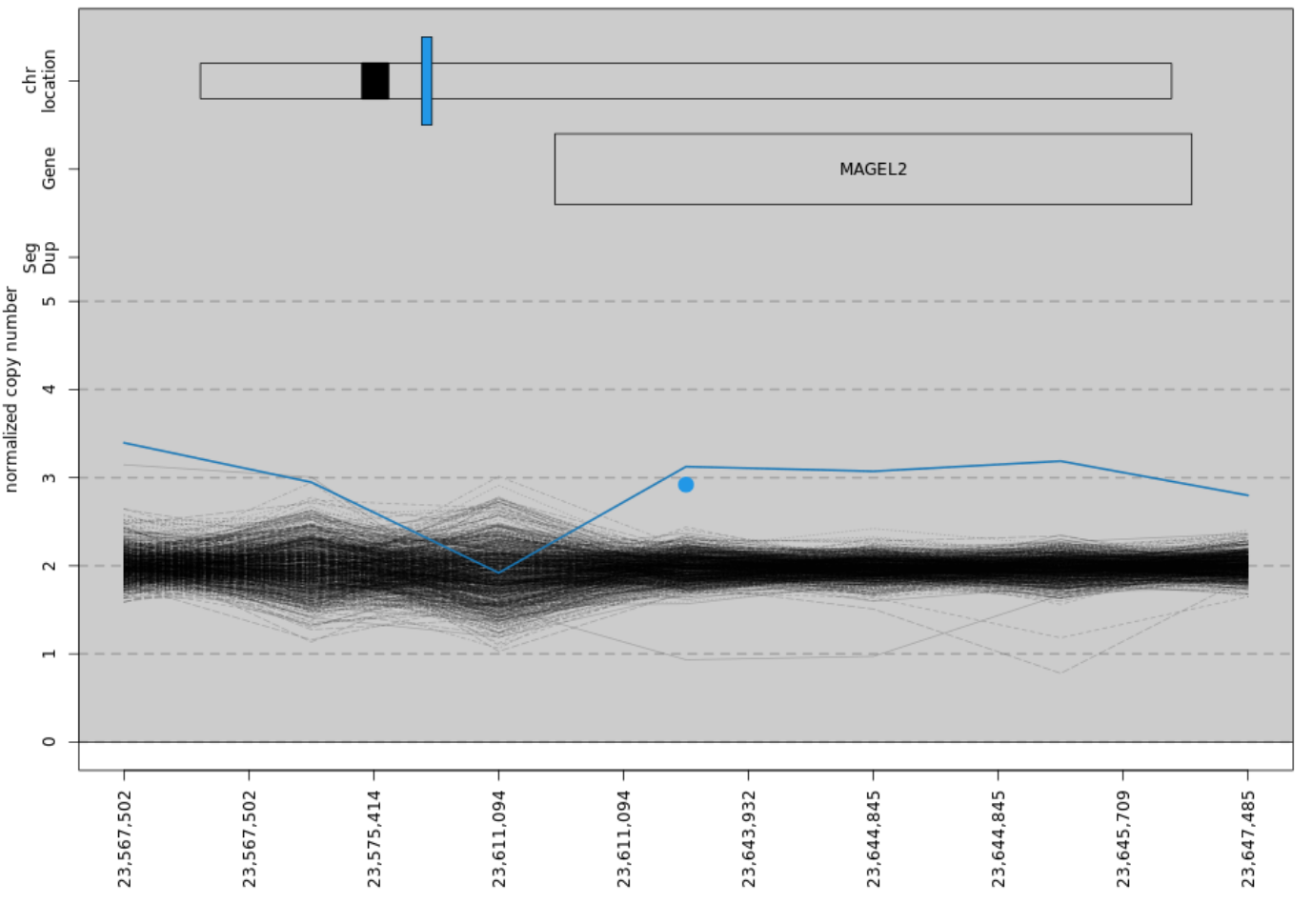

JP-KOB-S-00176
chr15:23235438-24312330

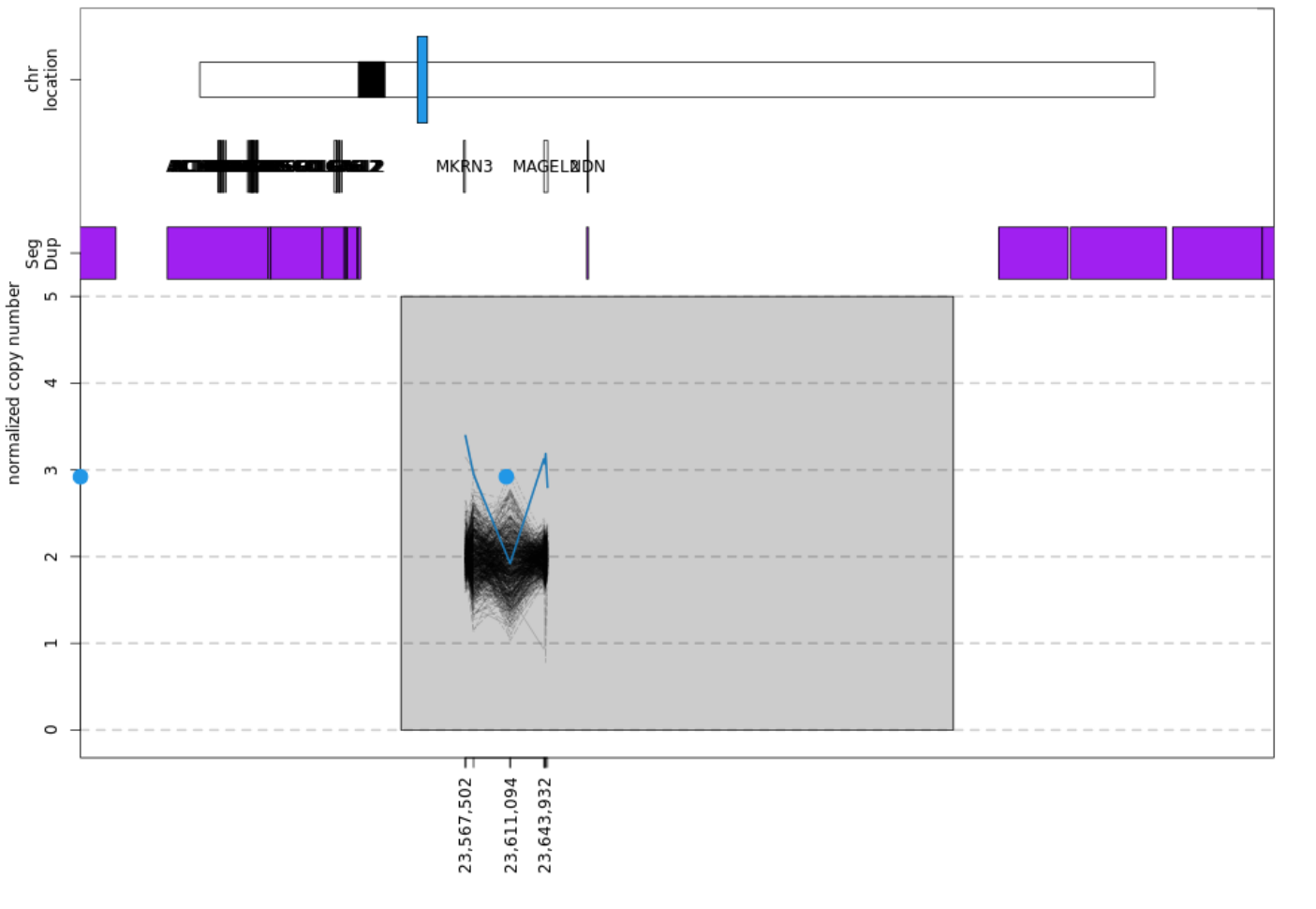

JP-KOB-S-00417
chr1:146177583-149048646

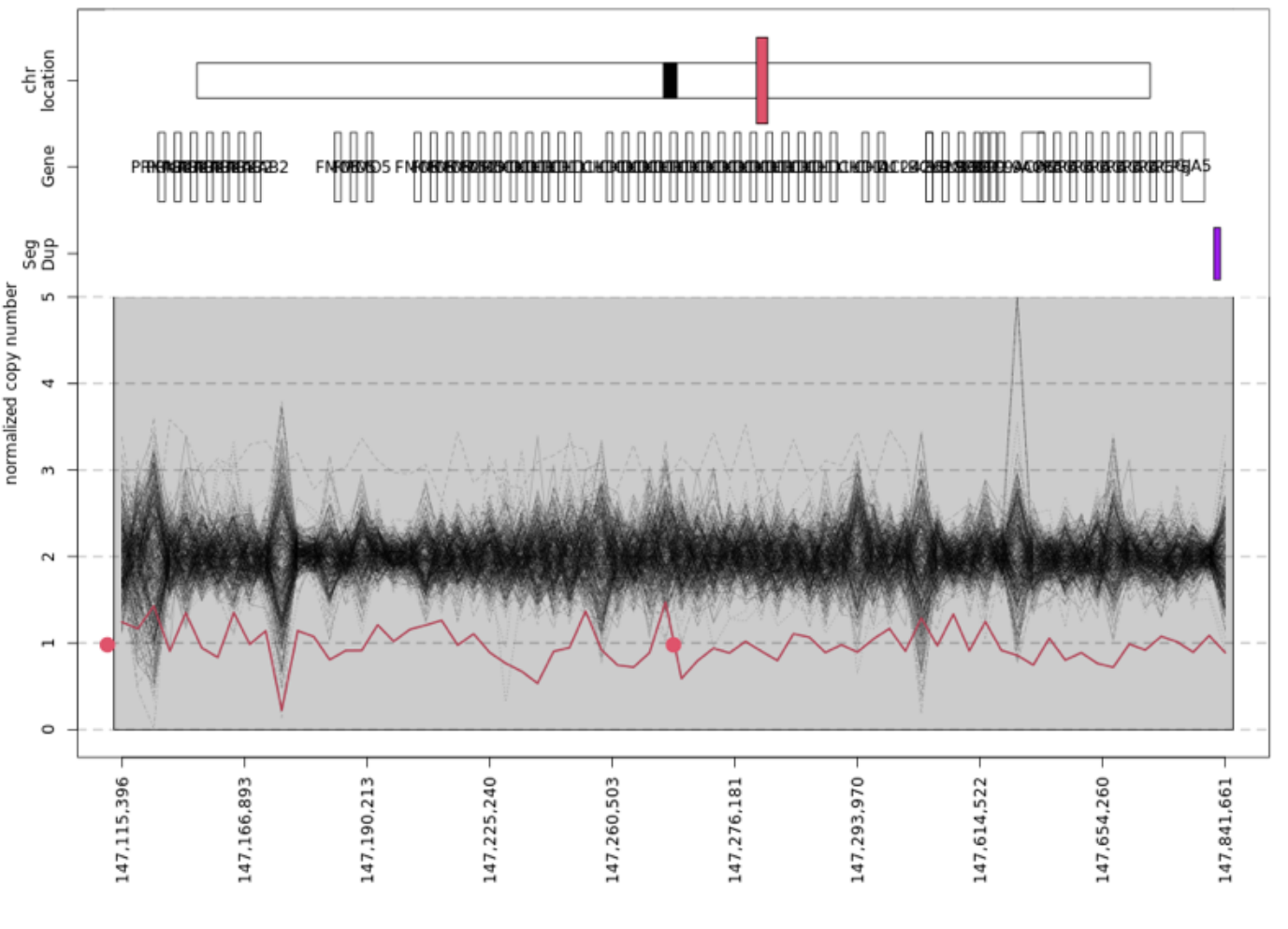

JP-KOB-S-00417
chr1:146177583-149048646

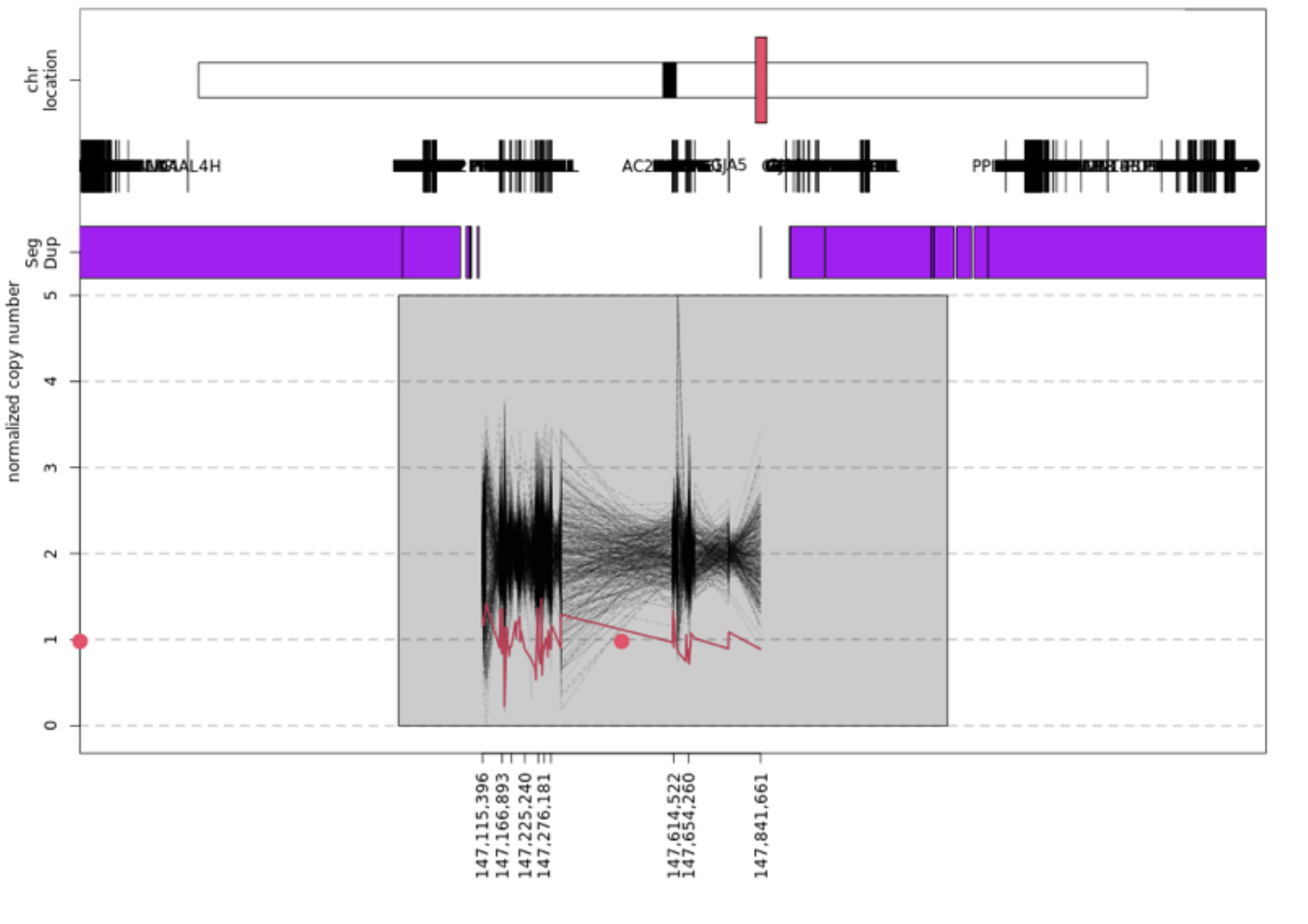

JP-KOB-S-00487
chr15:22850363-25378942

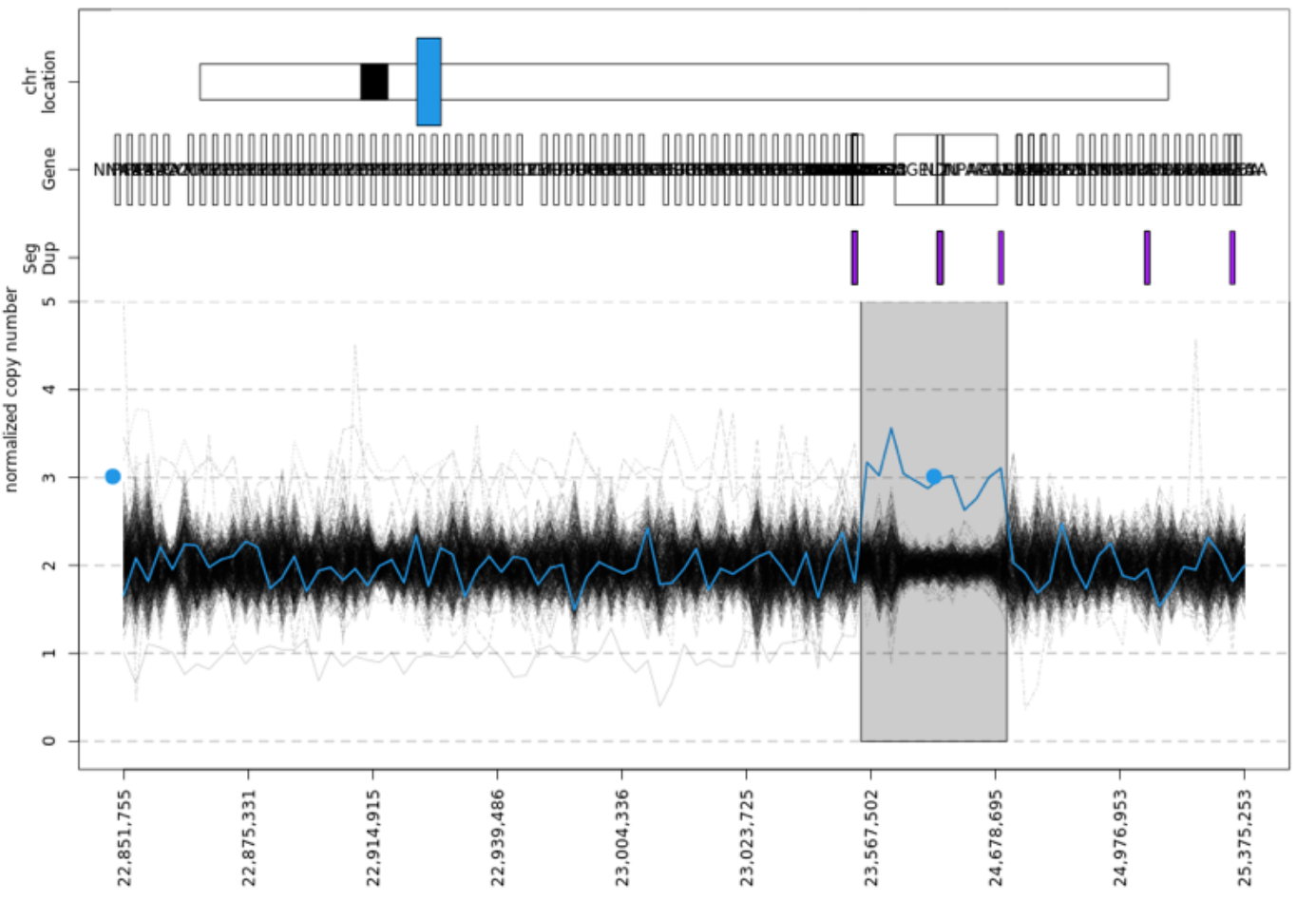

JP-KOB-S-00487
chr15:22850363-25378942

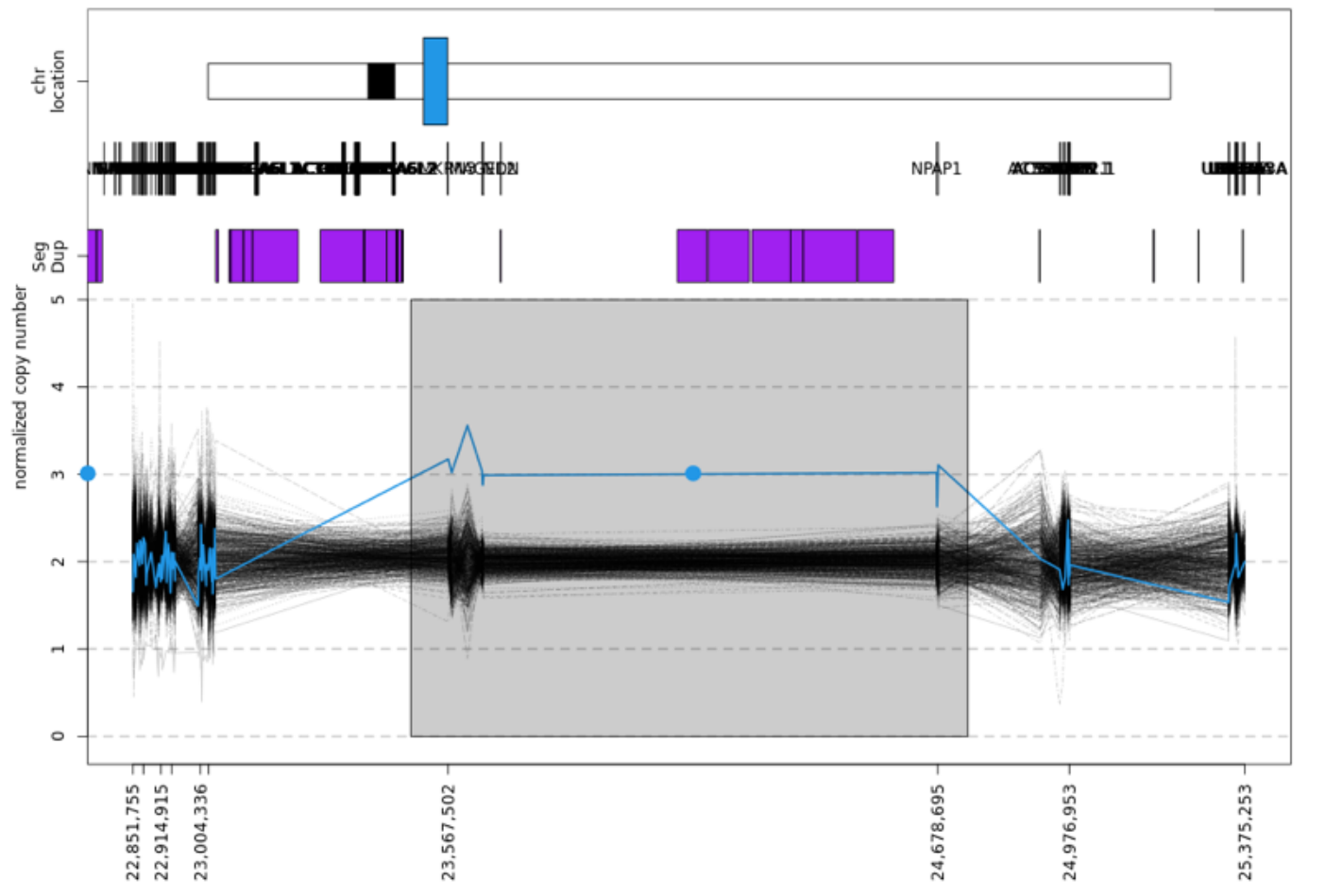

JP-NAR-S-00011
chr15:21269921-30069105

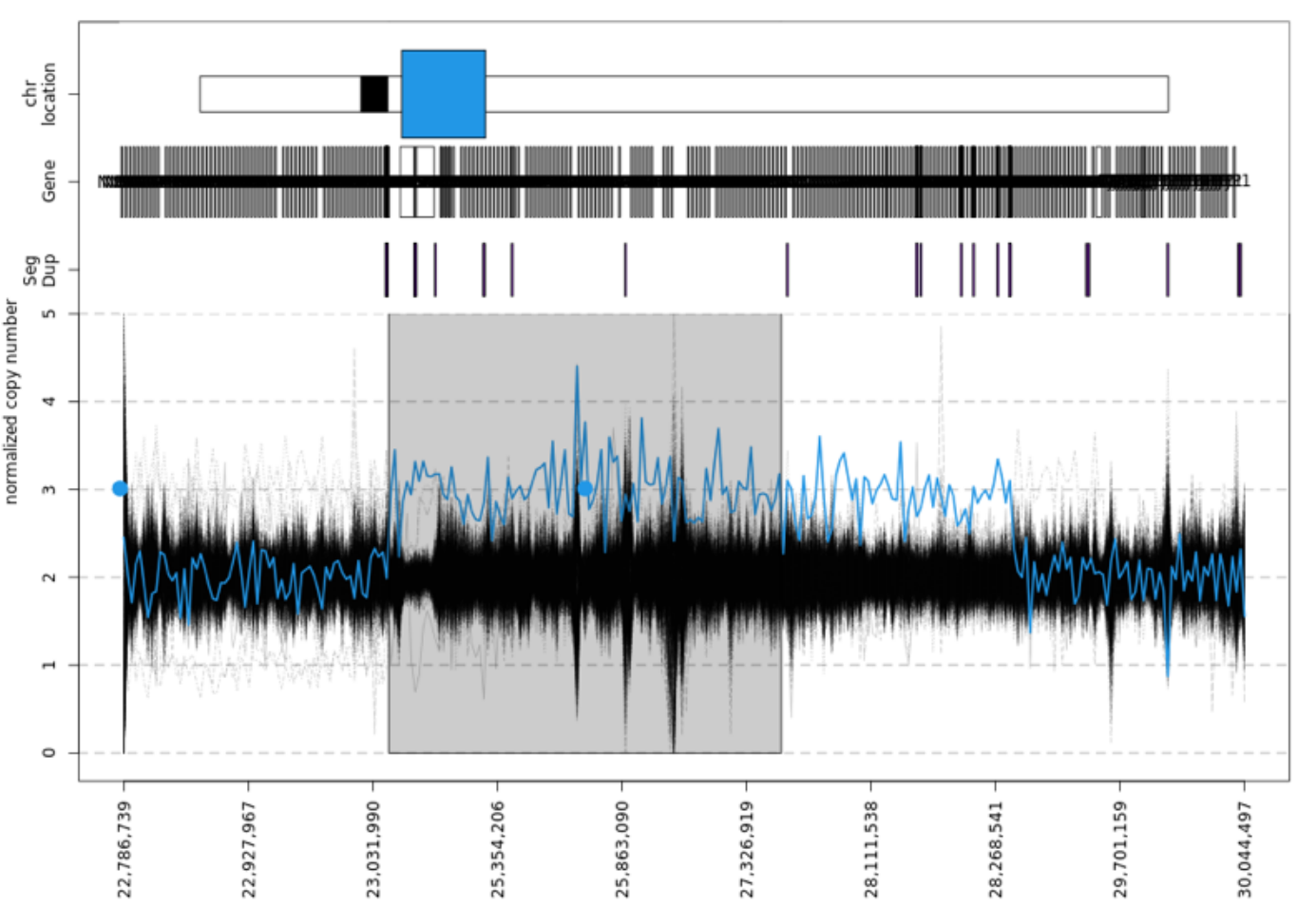

JP-NAR-S-00011
chr15:21269921-30069105

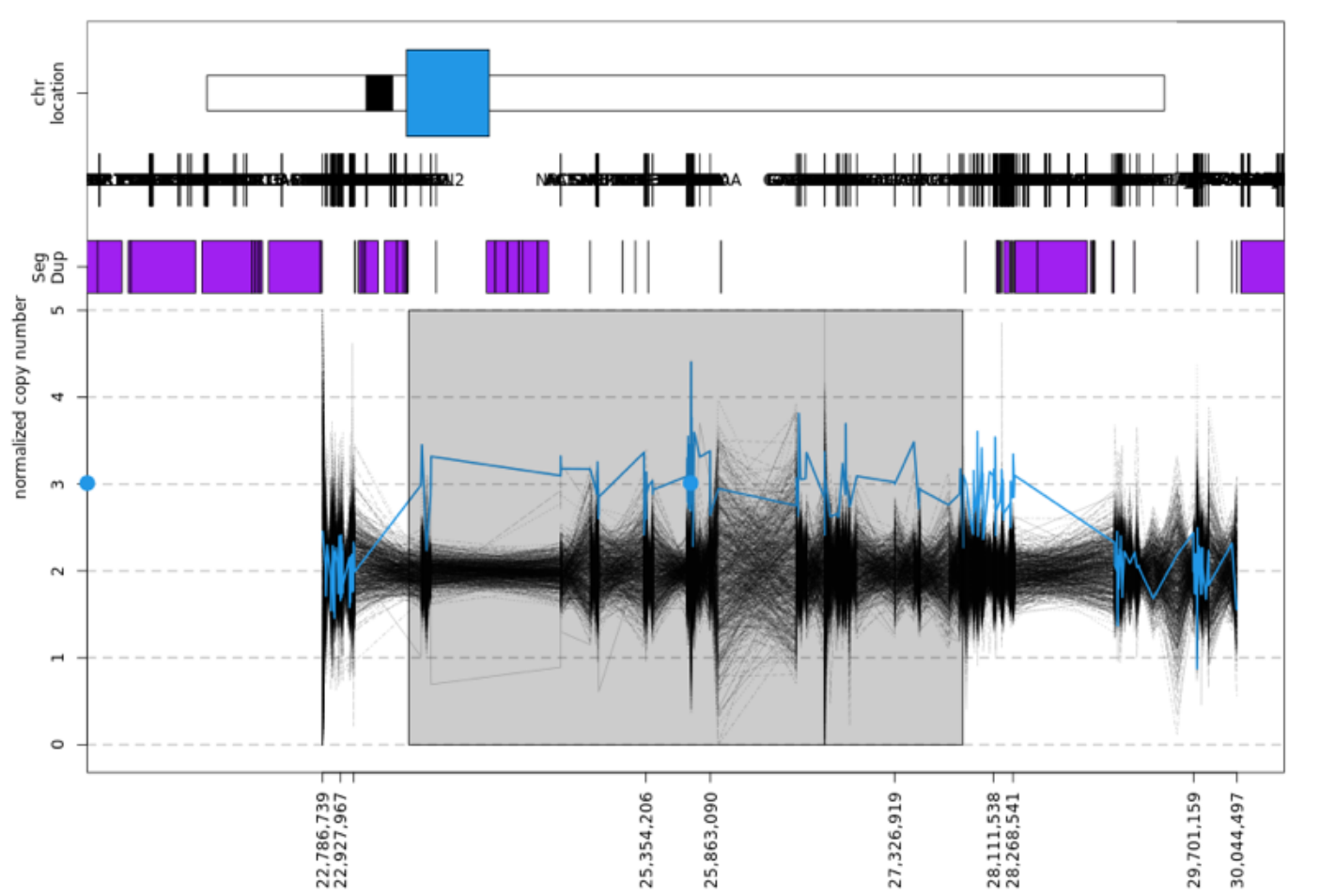

JP-RIK-S-00223
chr1:146171267-149050751

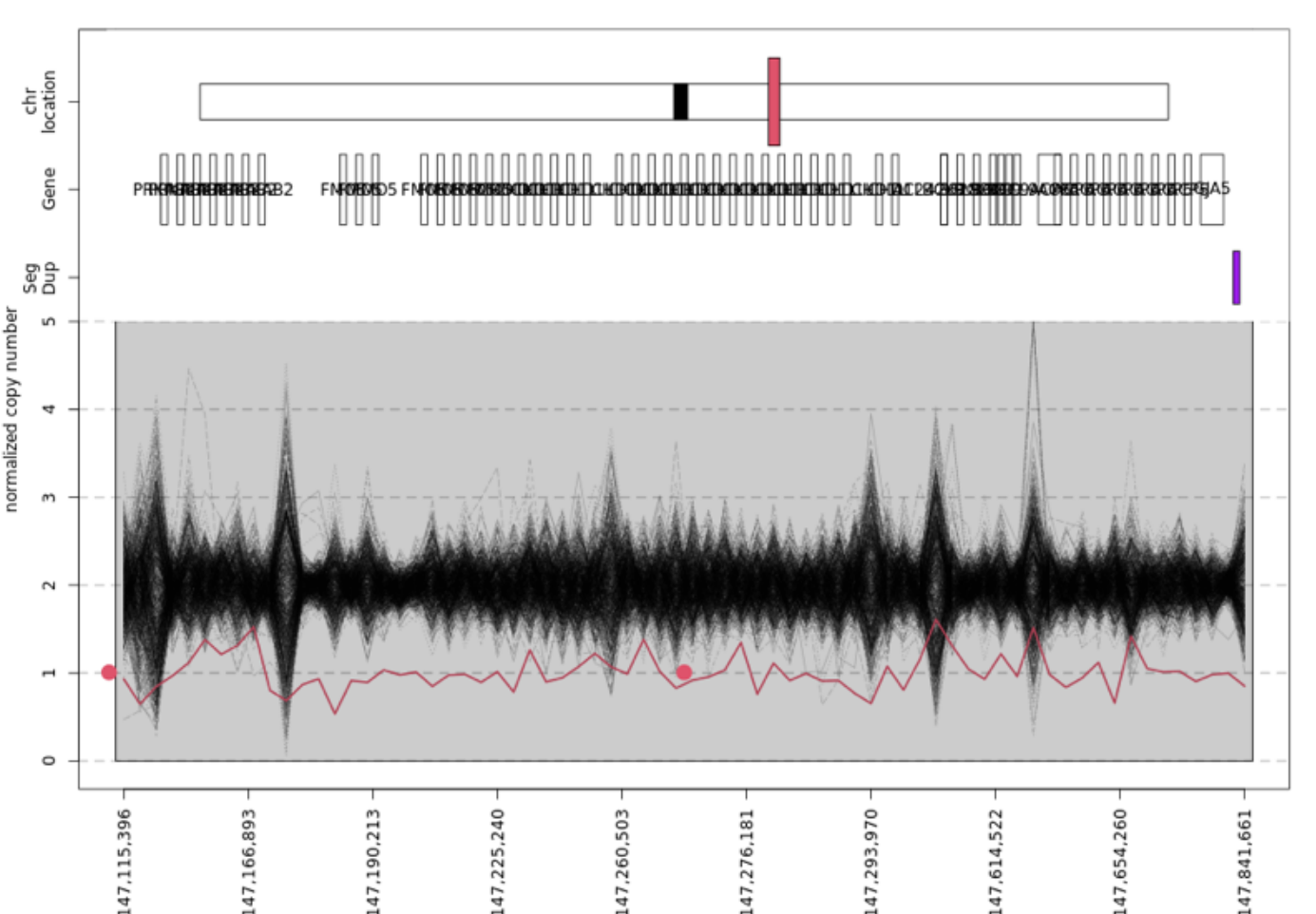

JP-RIK-S-00223
chr1:146171267-149050751

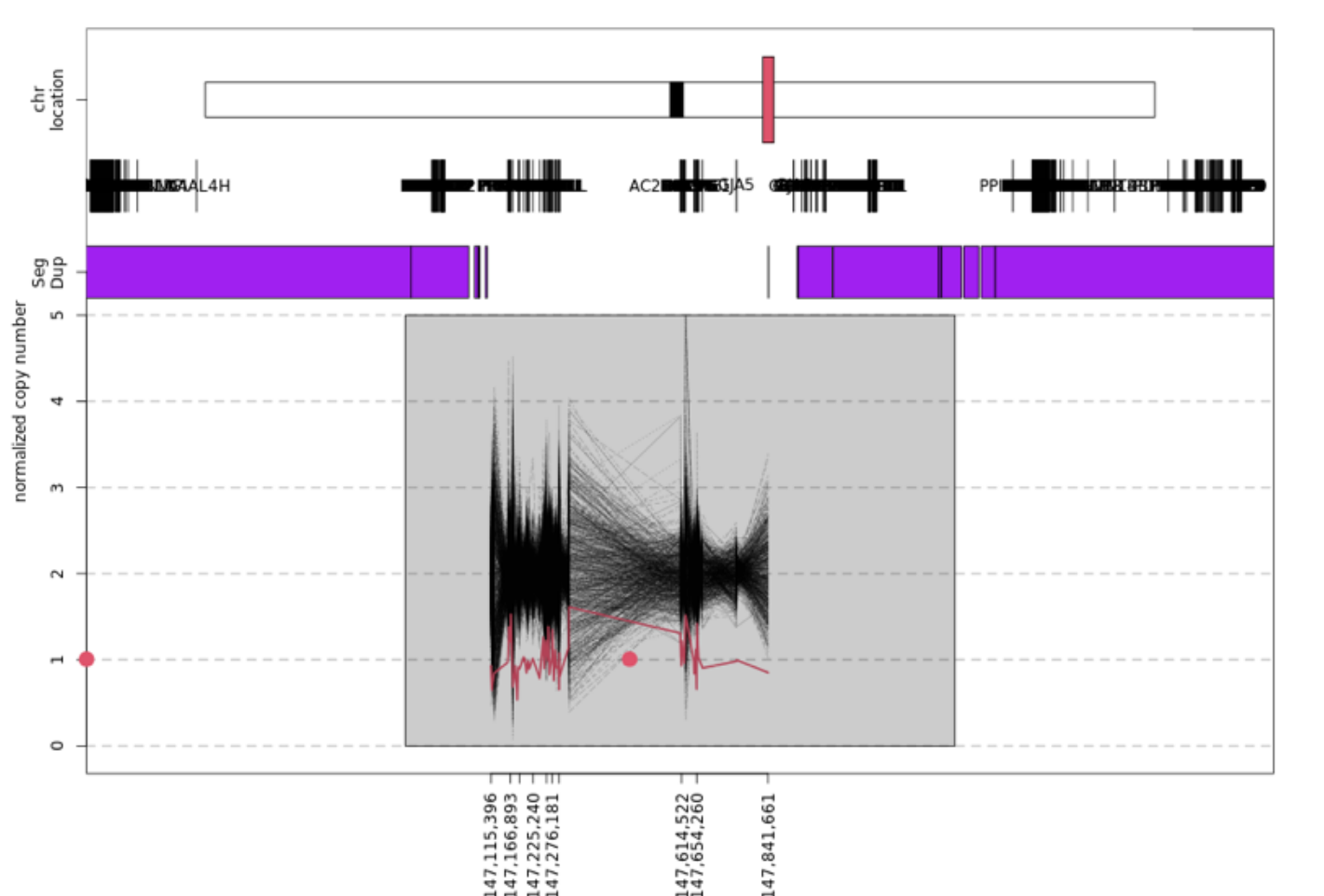

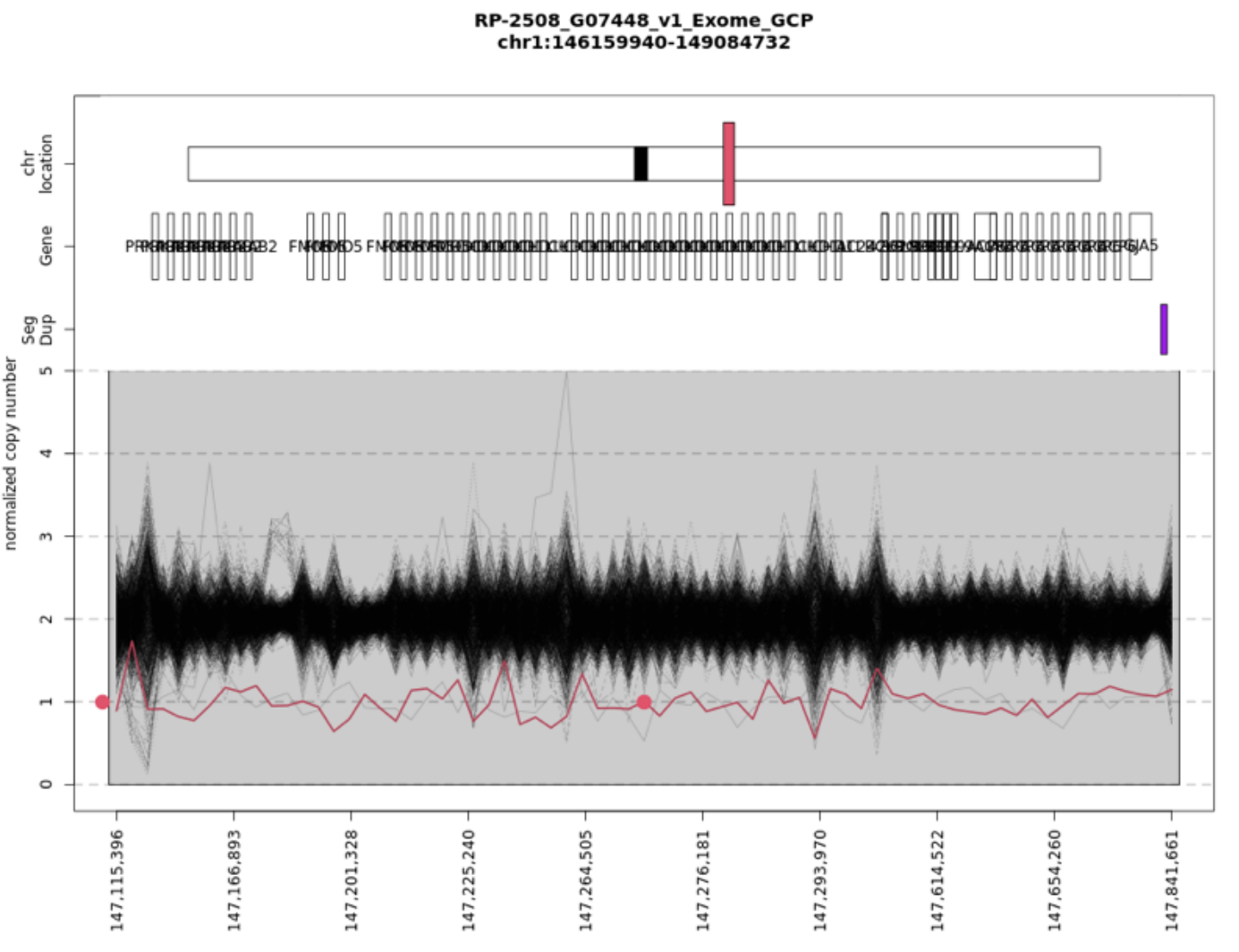
RP-2508_G07448_v1_Exome_GCP
chr1:146159940-149084732
chr location
Gene
Seg Dup
normalized copy number
0
1
2
3
4
5
147,115,396
147,166,893
147,201,328
147,225,240
147,264,505
147,276,181
147,293,970
147,614,522
147,654,260
147,841,661

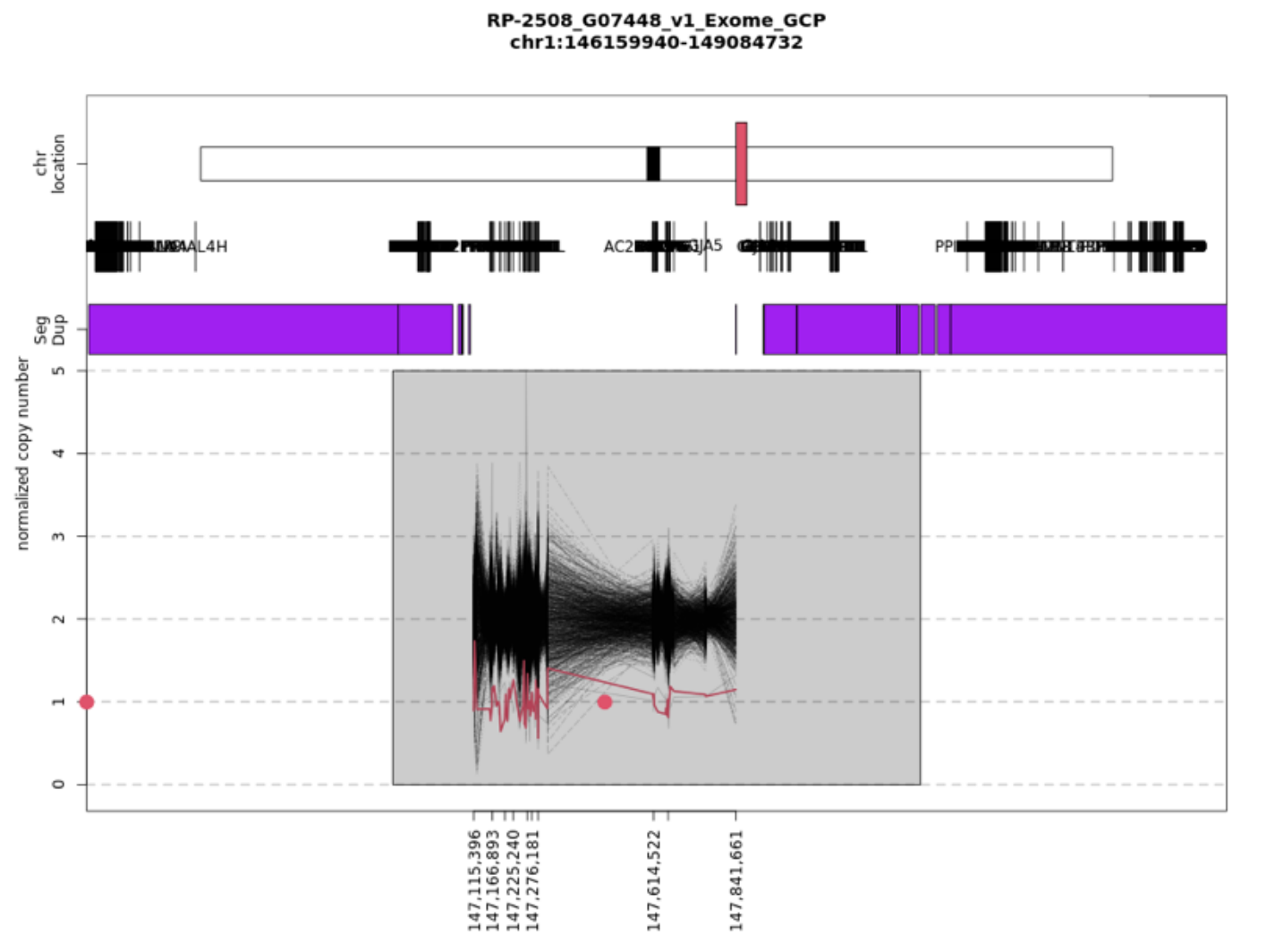
RP-2508_G07448_v1_Exome_GCP
chr1:146159940-149084732
chr location
Gene
Seg Dup
normalized copy number
0
1
2
3
4
5
147,115,396
147,166,893
147,225,240
147,276,181
147,614,522
147,841,661

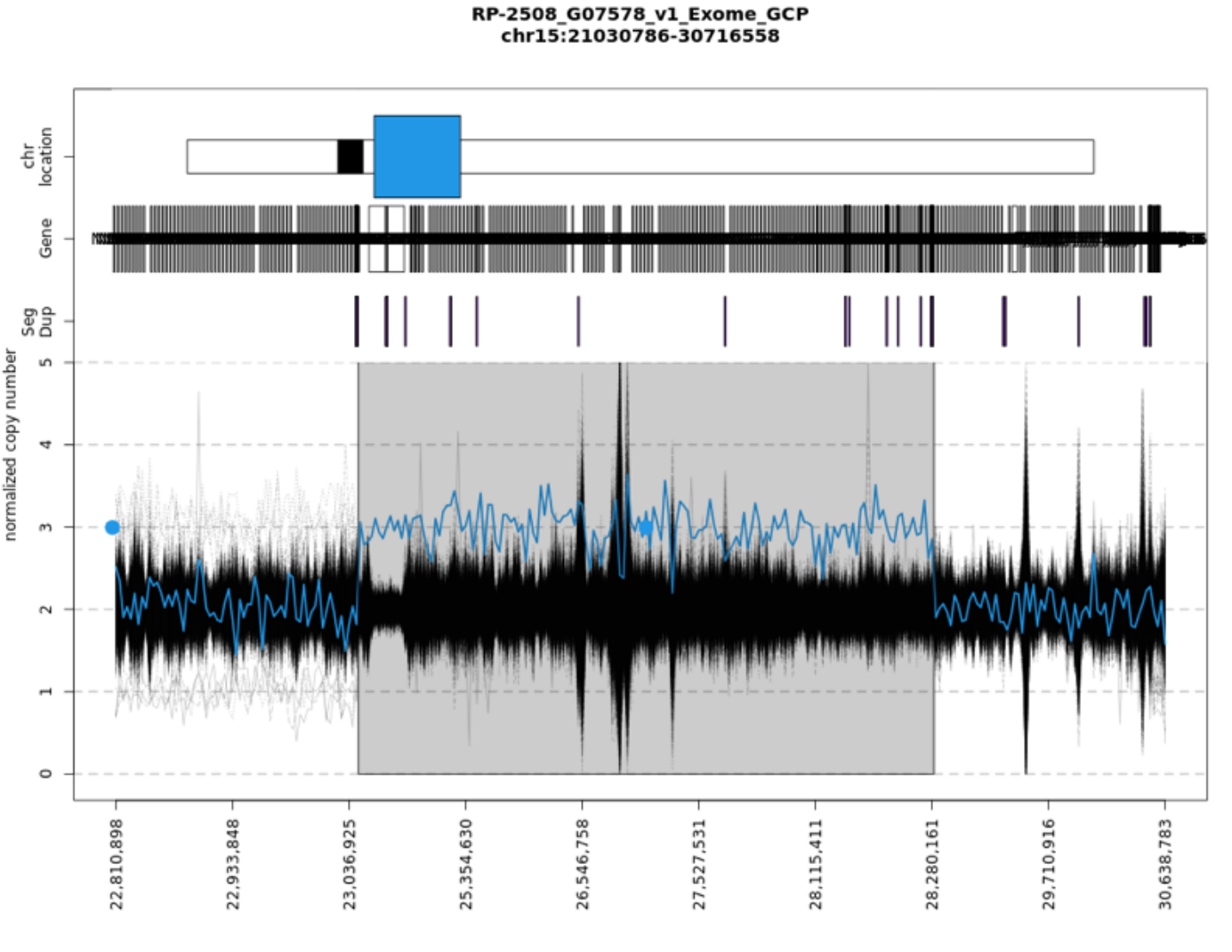
RP-2508_G07578_v1_Exome_GCP
chr15:21030786-30716558
chr location
Gene
Seg Dup
normalized copy number
0
1
2
3
4
5
22,810,898
22,933,848
23,036,925
25,354,630
26,546,758
27,527,531
28,115,411
28,280,161
29,710,916
30,638,783

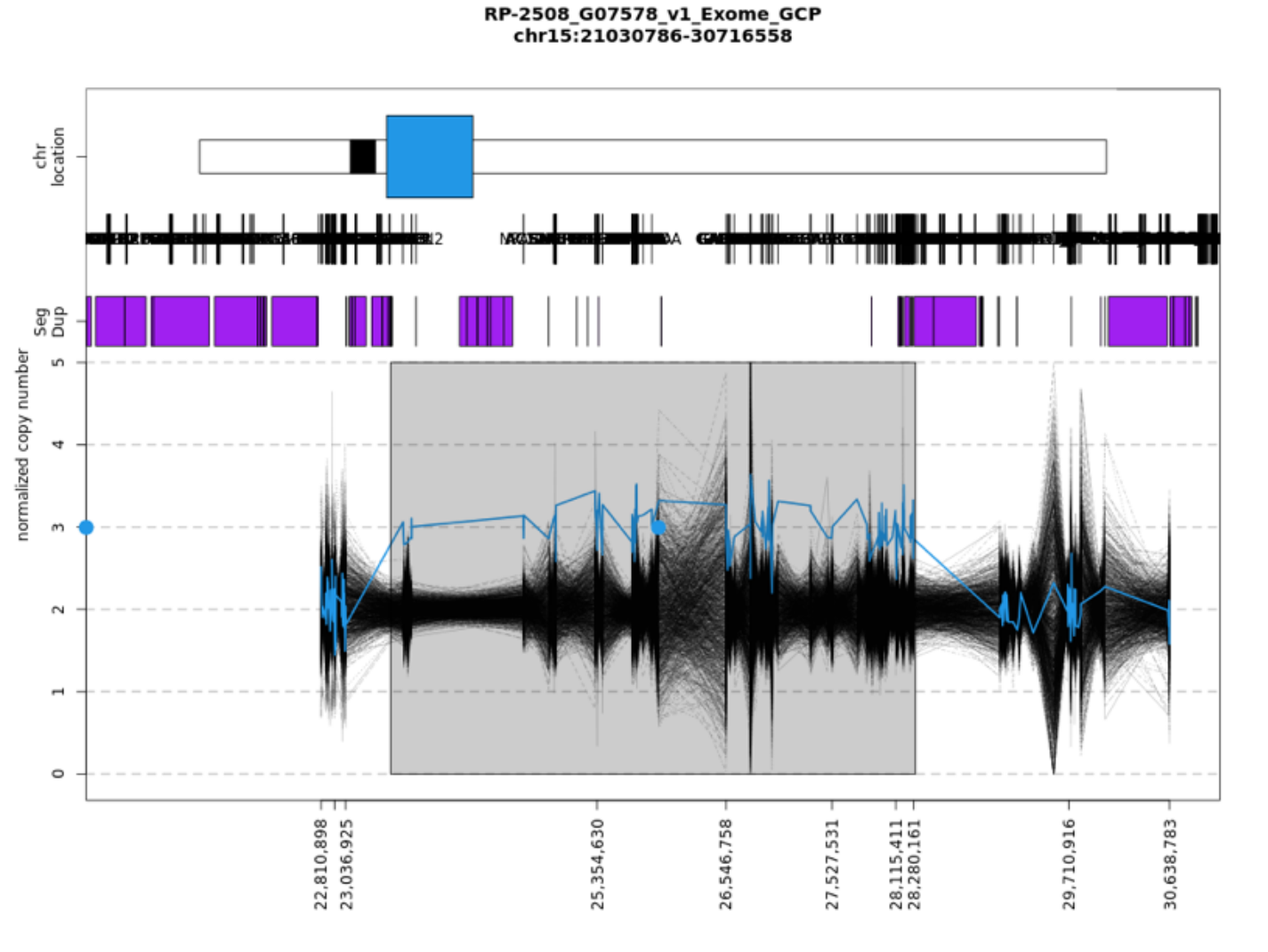
RP-2508_G07578_v1_Exome_GCP
chr15:21030786-30716558
chr location
Gene
Seg Dup
normalized copy number
0
1
2
3
4
5
22,810,898
23,036,925
25,354,630
26,546,758
27,527,531
28,115,411
28,280,161
29,710,916
30,638,783

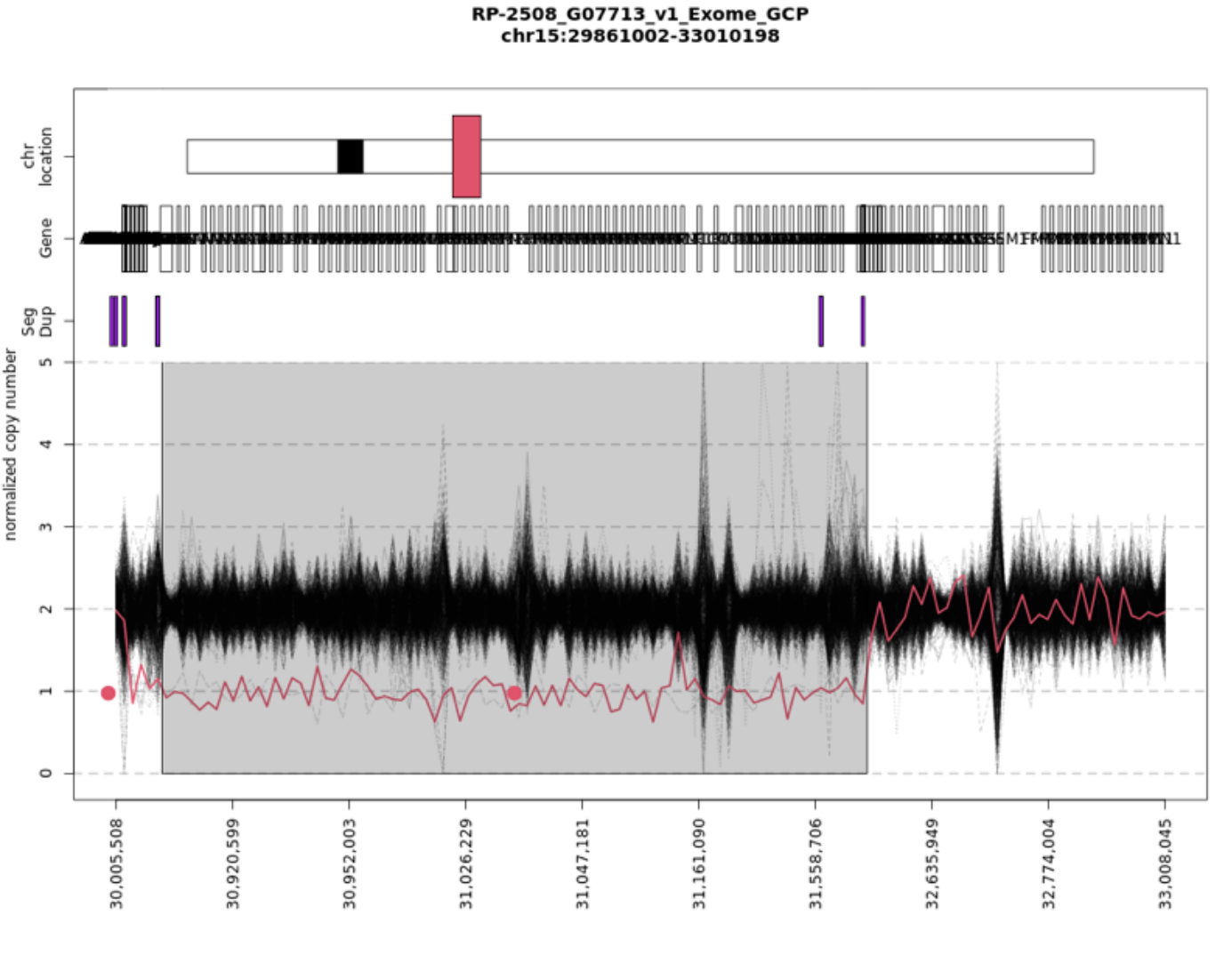
RP-2508_G07713_v1_Exome_GCP
chr15:29861002-33010198
chr location
Gene
Seg Dup
normalized copy number
0
1
2
3
4
5
30,005,508
30,920,599
30,952,003
31,026,229
31,047,181
31,161,090
31,558,706
32,635,949
32,774,004
33,008,045

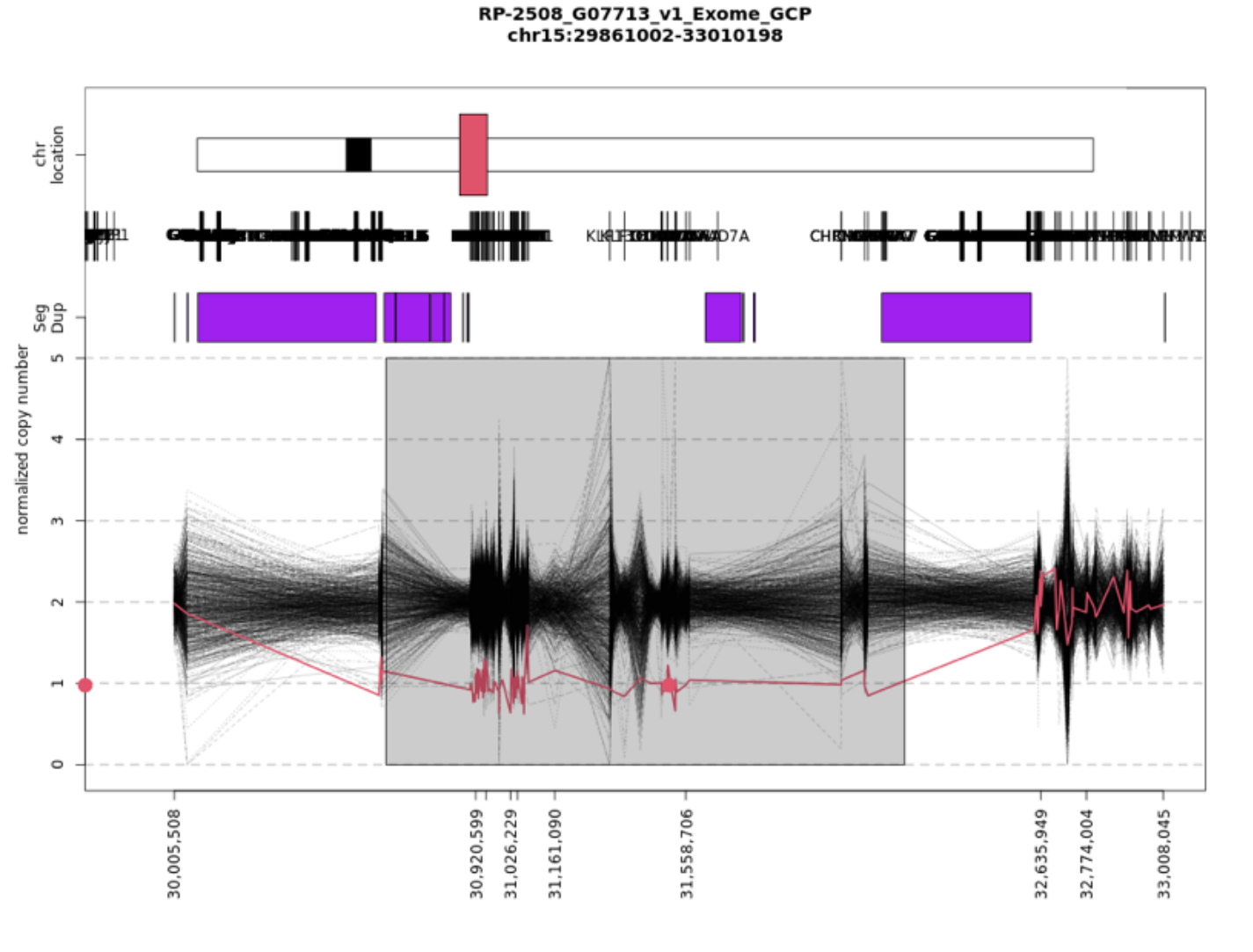
RP-2508_G07713_v1_Exome_GCP
chr15:29861002-33010198
chr location
Gene
Seg Dup
normalized copy number
0
1
2
3
4
5
30,005,508
30,920,599
31,026,229
31,161,090
31,558,706
32,635,949
32,774,004
33,008,045

RP-2508_G05052_v2_Exome_GCP
chr15:21076204-30701419

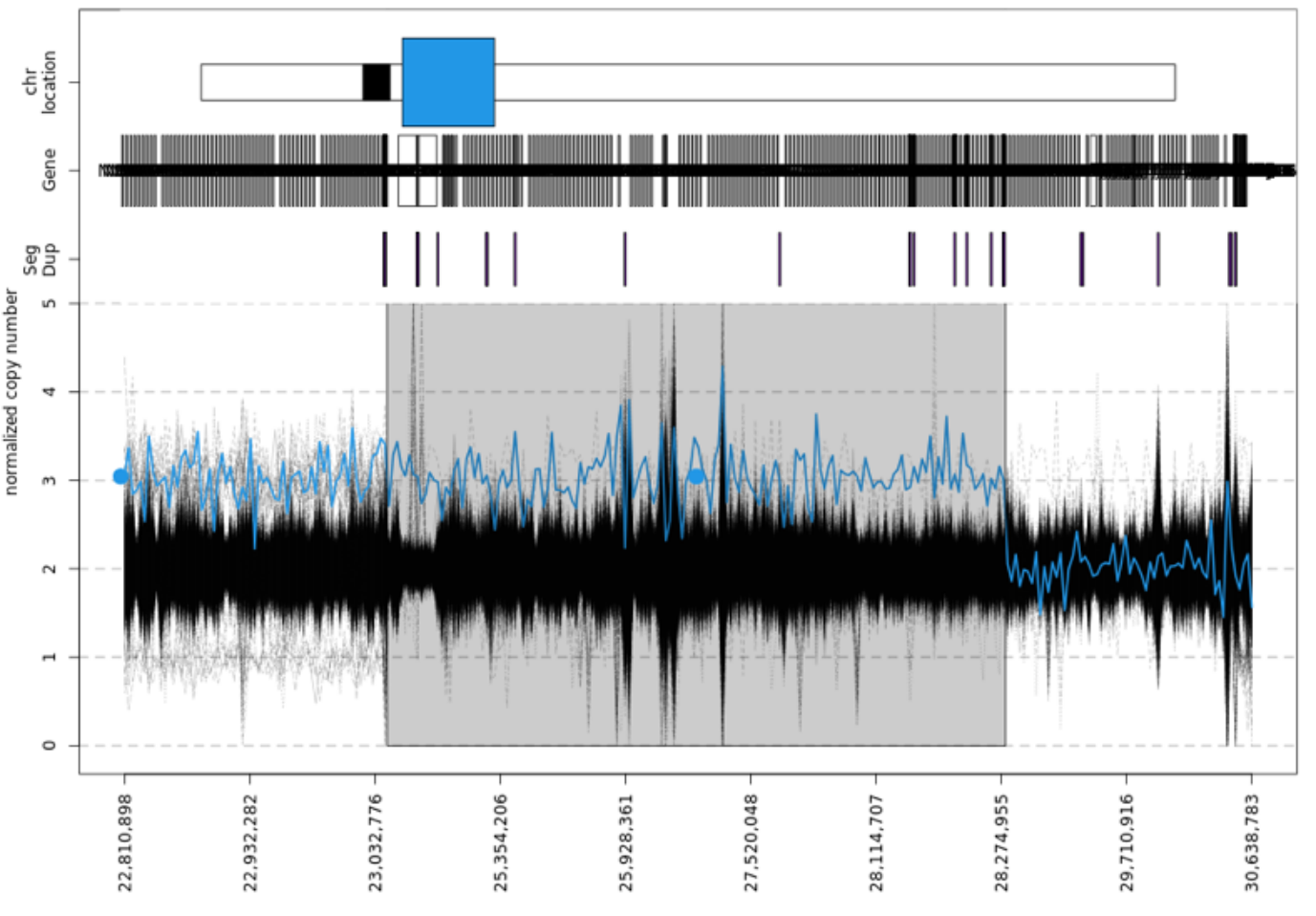

RP-2508_G05052_v2_Exome_GCP
chr15:21076204-30701419

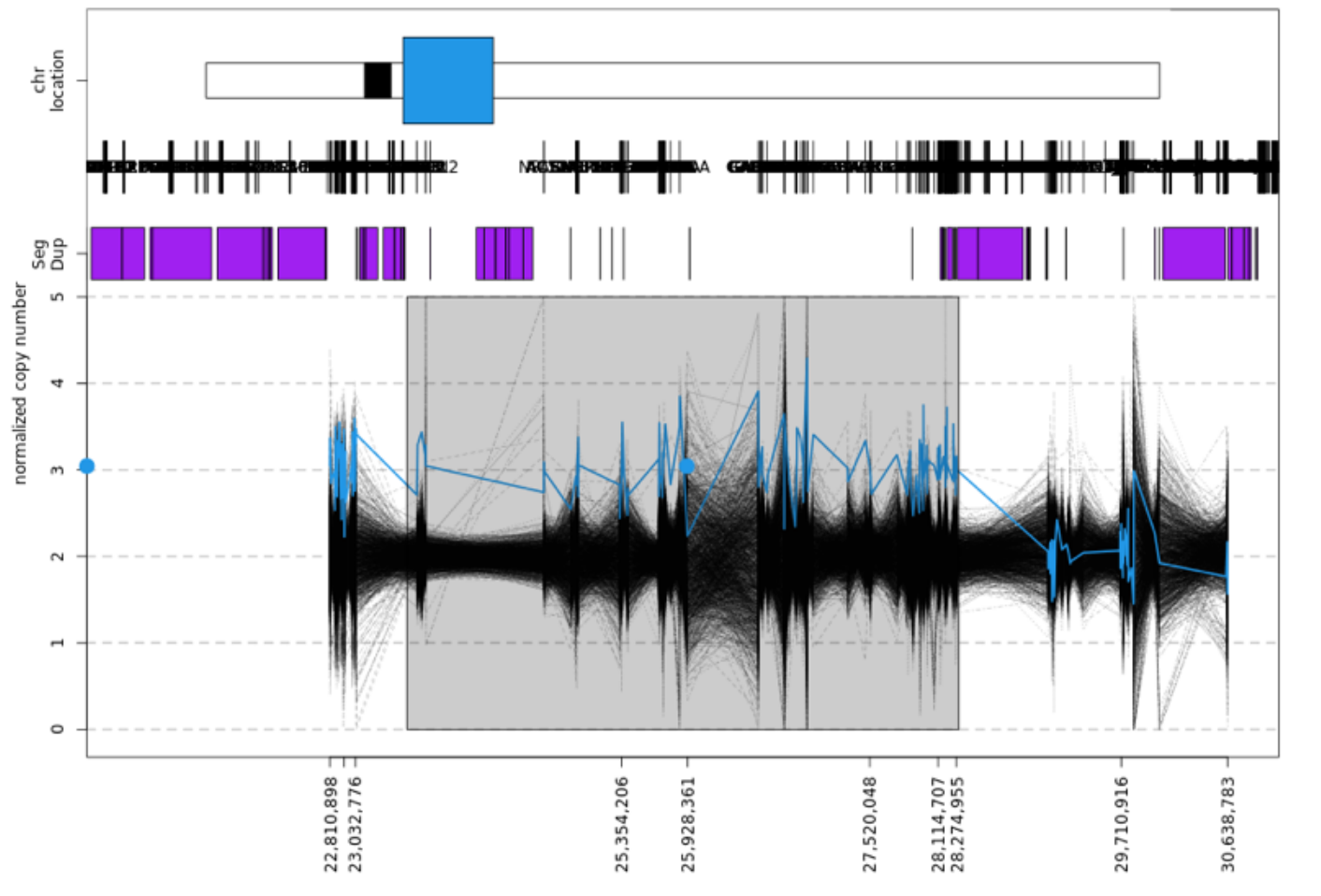

RP-2508_G06410_v1_Exome_GCP
chr1:146159940-149084732

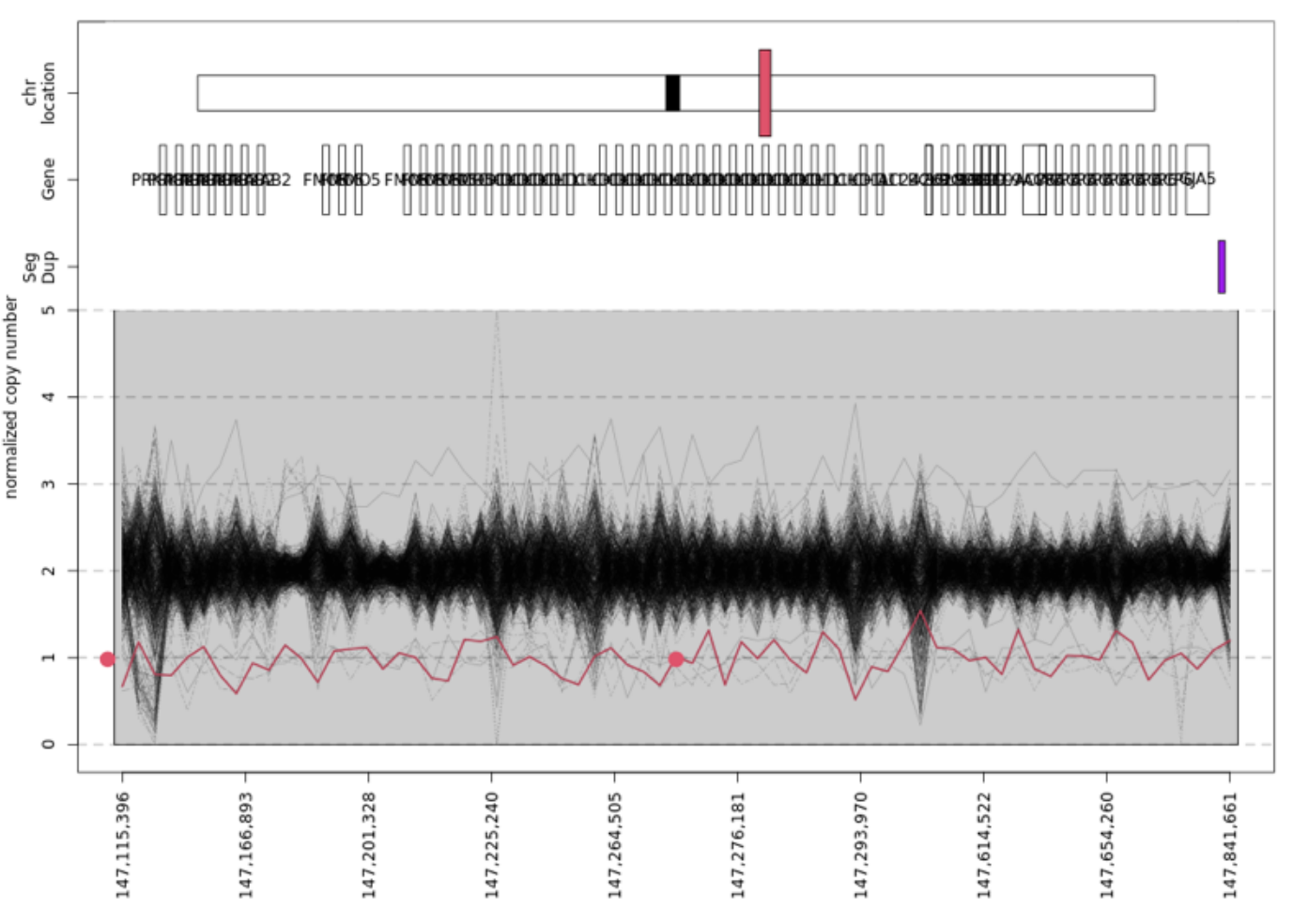

RP-2508_G06410_v1_Exome_GCP
chr1:146159940-149084732

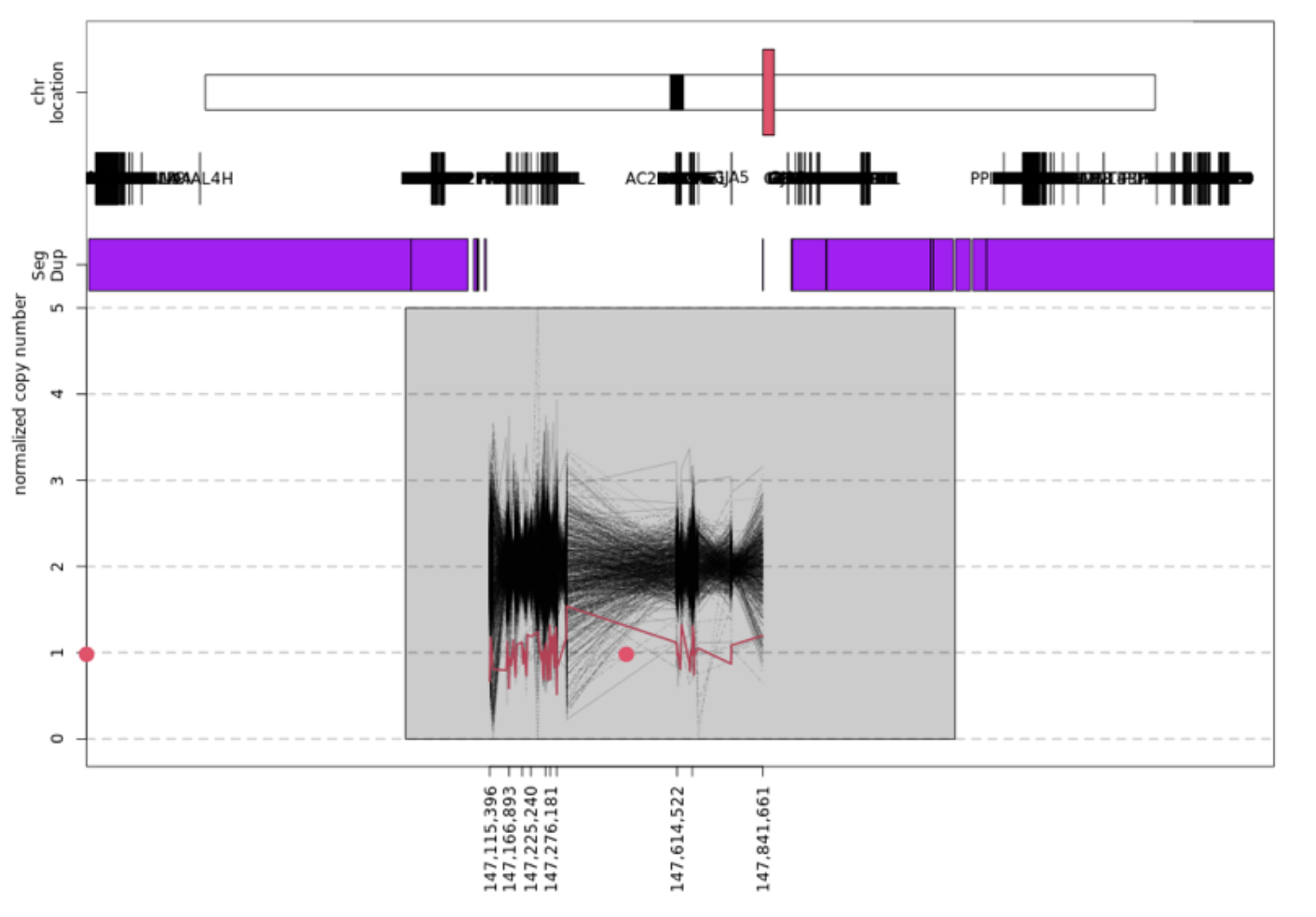

RP-2508_G06411_v2_Exome_GCP
chr1:146159940-149084732

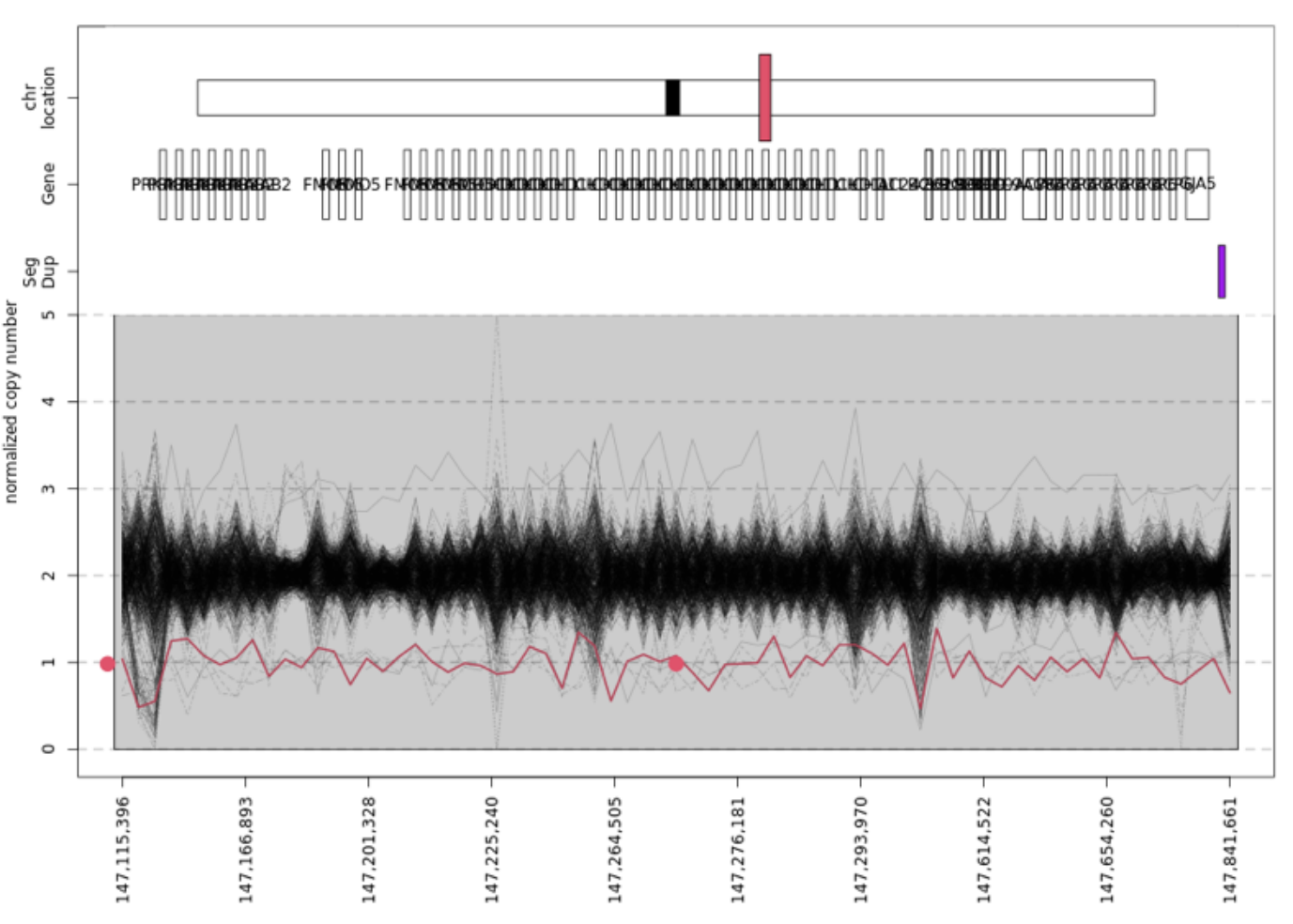

RP-2508_G06411_v2_Exome_GCP
chr1:146159940-149084732

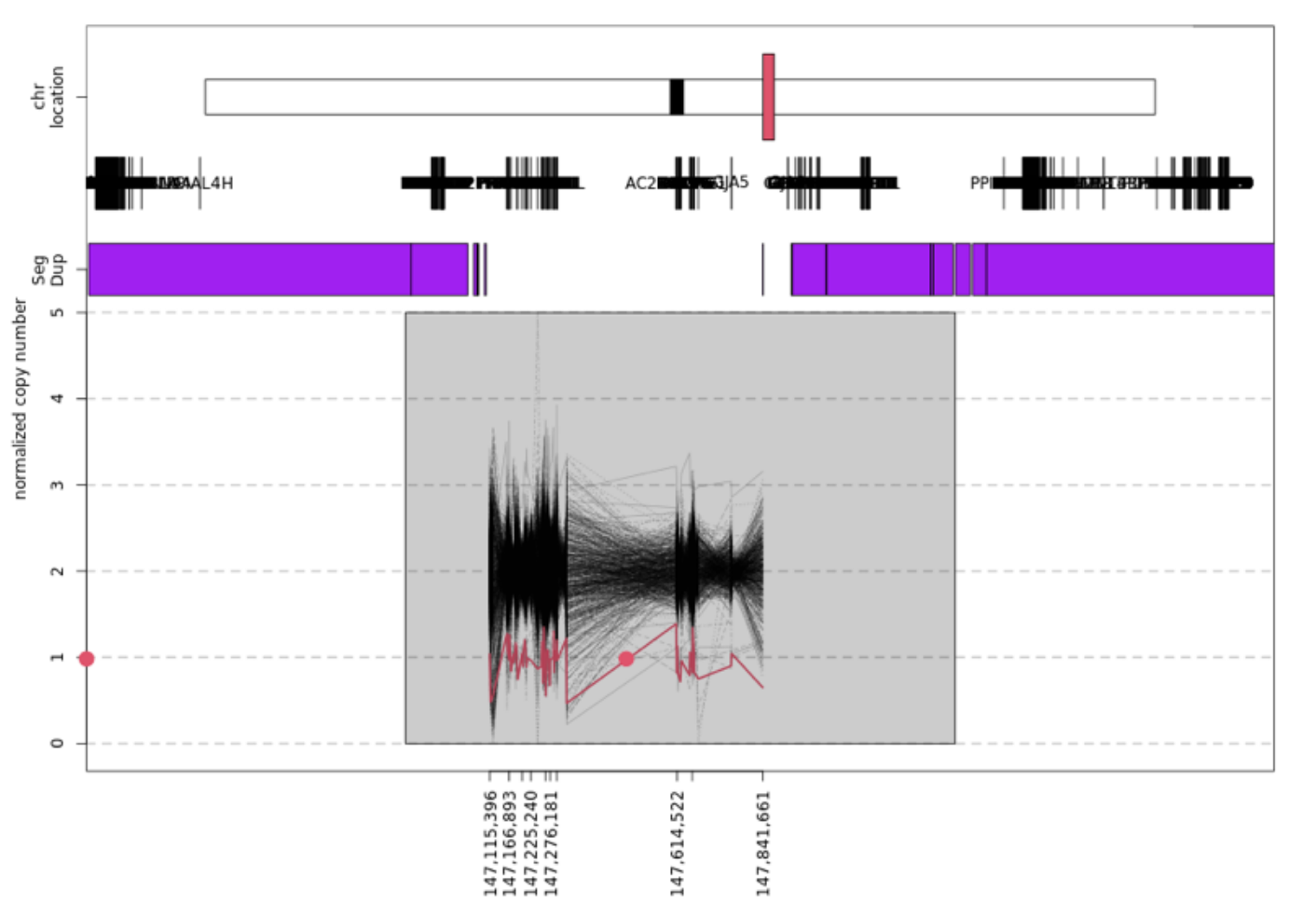

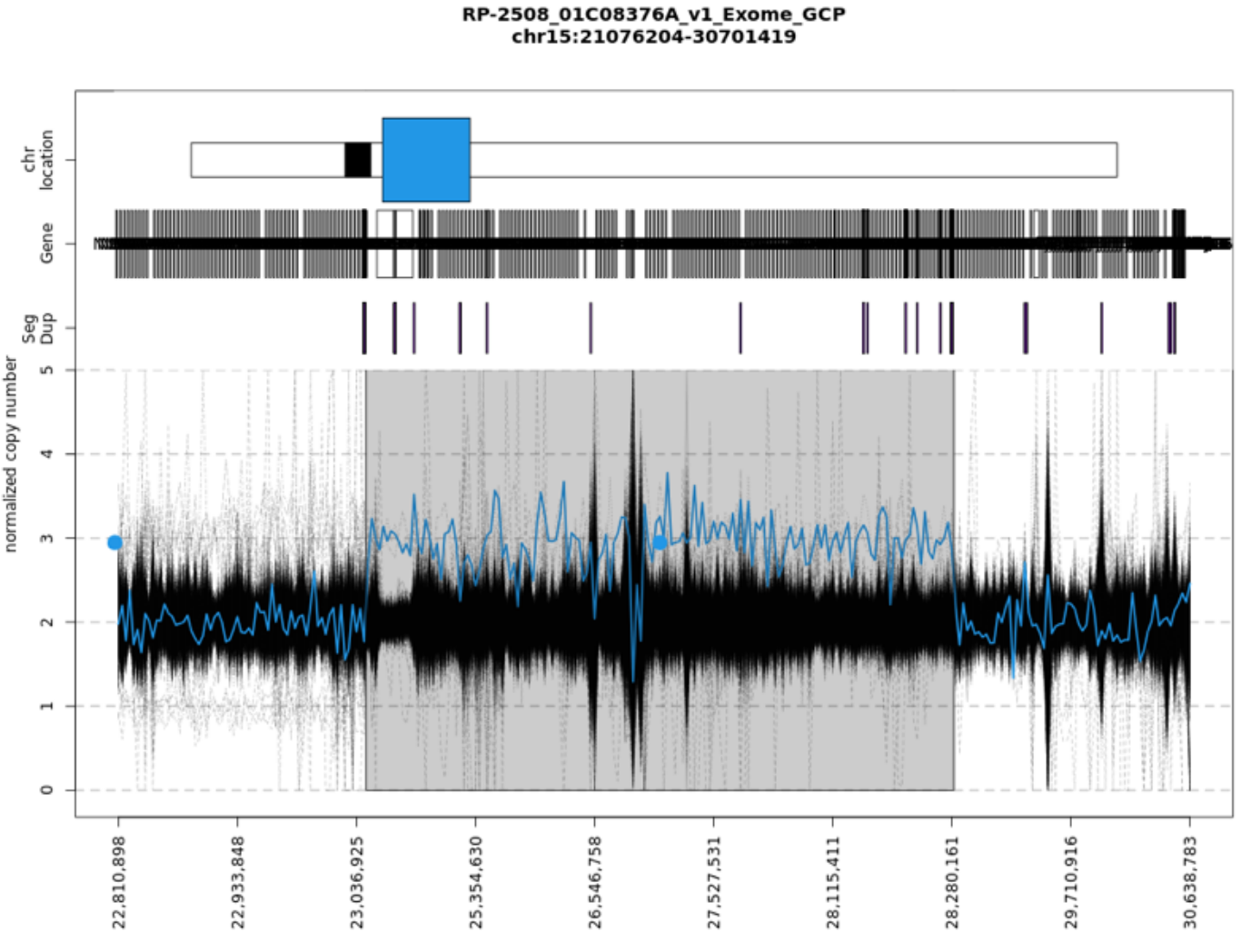
RP-2508_01C08376A_v1_Exome_GCP
chr15:21076204-30701419
chr location
Gene
Seg Dup
normalized copy number
22,810,898
22,933,848
23,036,925
25,354,630
26,546,758
27,527,531
28,115,411
28,280,161
29,710,916
30,638,783

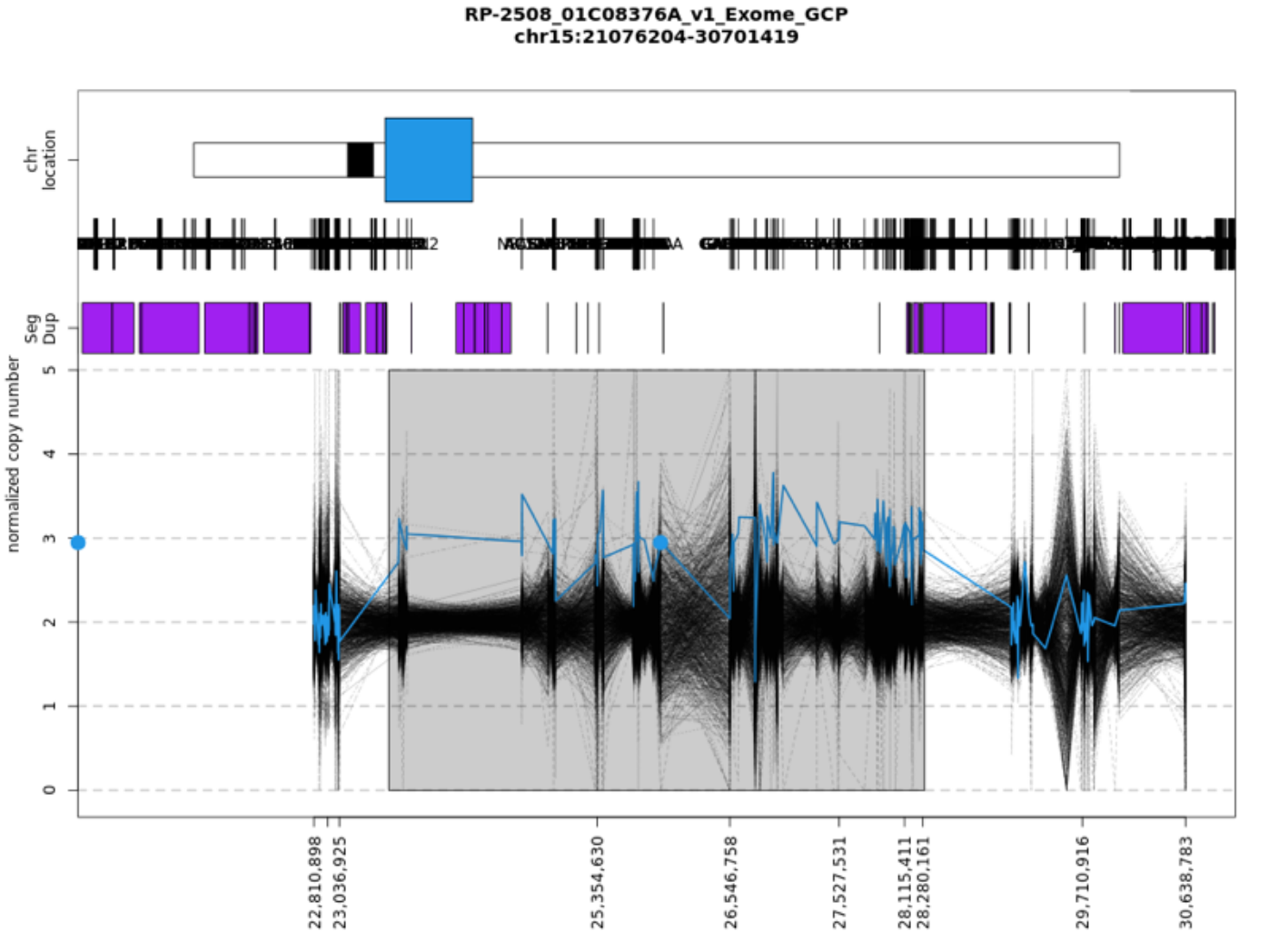
RP-2508_01C08376A_v1_Exome_GCP
chr15:21076204-30701419
chr location
Gene
Seg Dup
normalized copy number
22,810,898
23,036,925
25,354,630
26,546,758
27,527,531
28,115,411
28,280,161
29,710,916
30,638,783

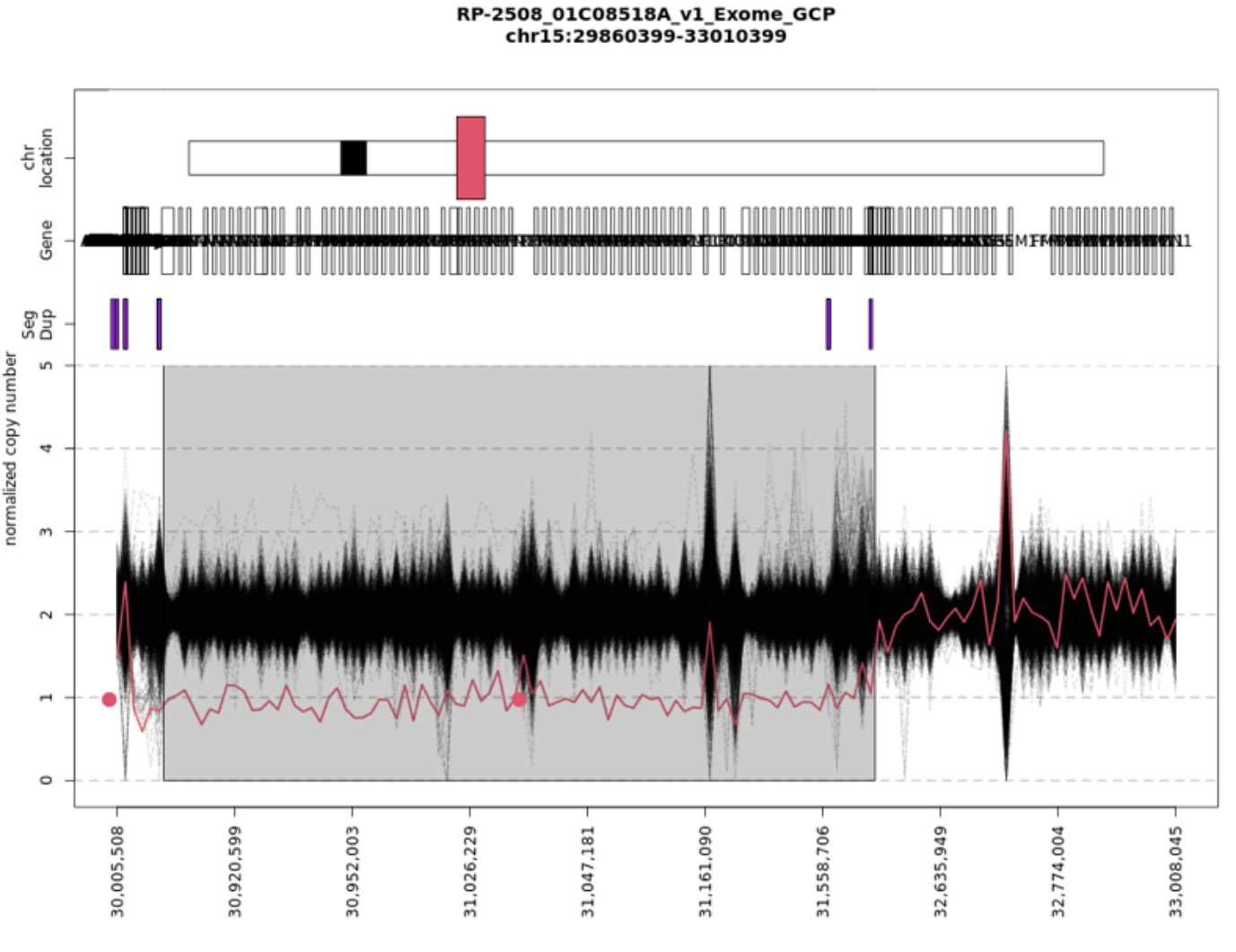
RP-2508_01C08518A_v1_Exome_GCP
chr15:29860399-33010399
chr location
Gene
Seg Dup
normalized copy number
30,005,508
30,920,599
30,952,003
31,026,229
31,047,181
31,161,090
31,558,706
32,635,949
32,774,004
33,008,045

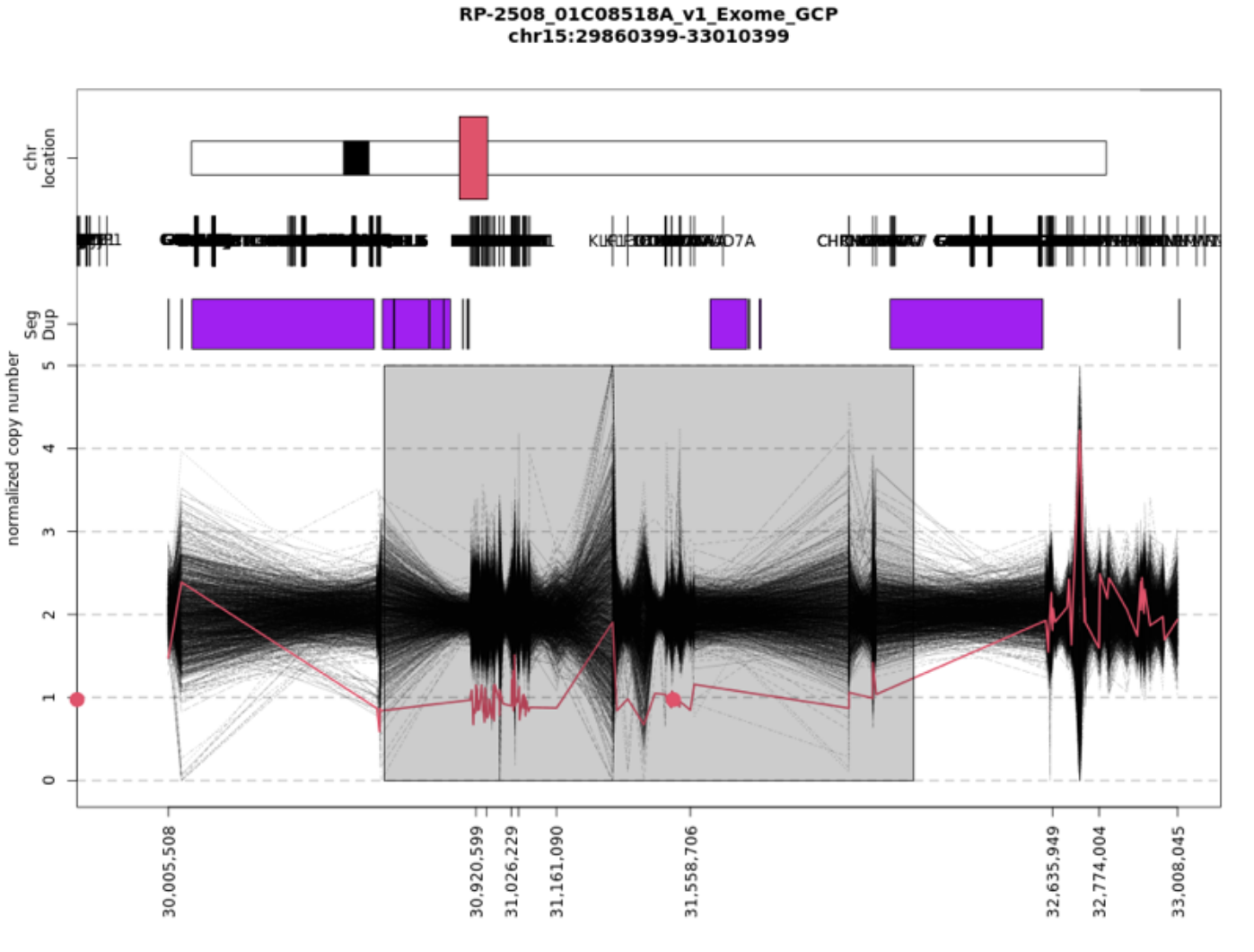
RP-2508_01C08518A_v1_Exome_GCP
chr15:29860399-33010399
chr location
Gene
Seg Dup
normalized copy number
30,005,508
30,920,599
31,026,229
31,161,090
31,558,706
32,635,949
32,774,004
33,008,045

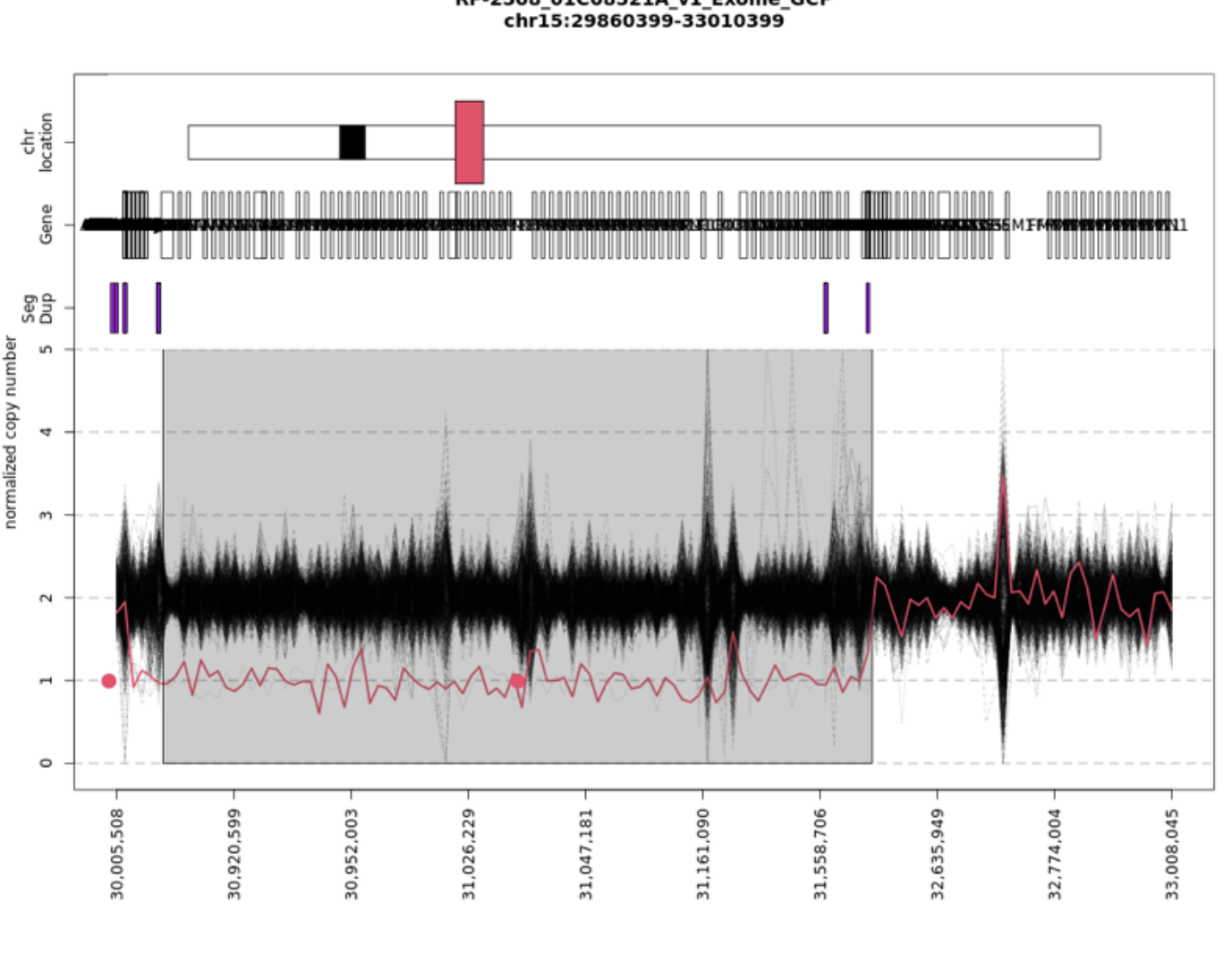
RP-2508_01C08521A_v1_Exome_GCP
chr15:29860399-33010399
chr location
Gene
Seg Dup
normalized copy number
30,005,508
30,920,599
30,952,003
31,026,229
31,047,181
31,161,090
31,558,706
32,635,949
32,774,004
33,008,045

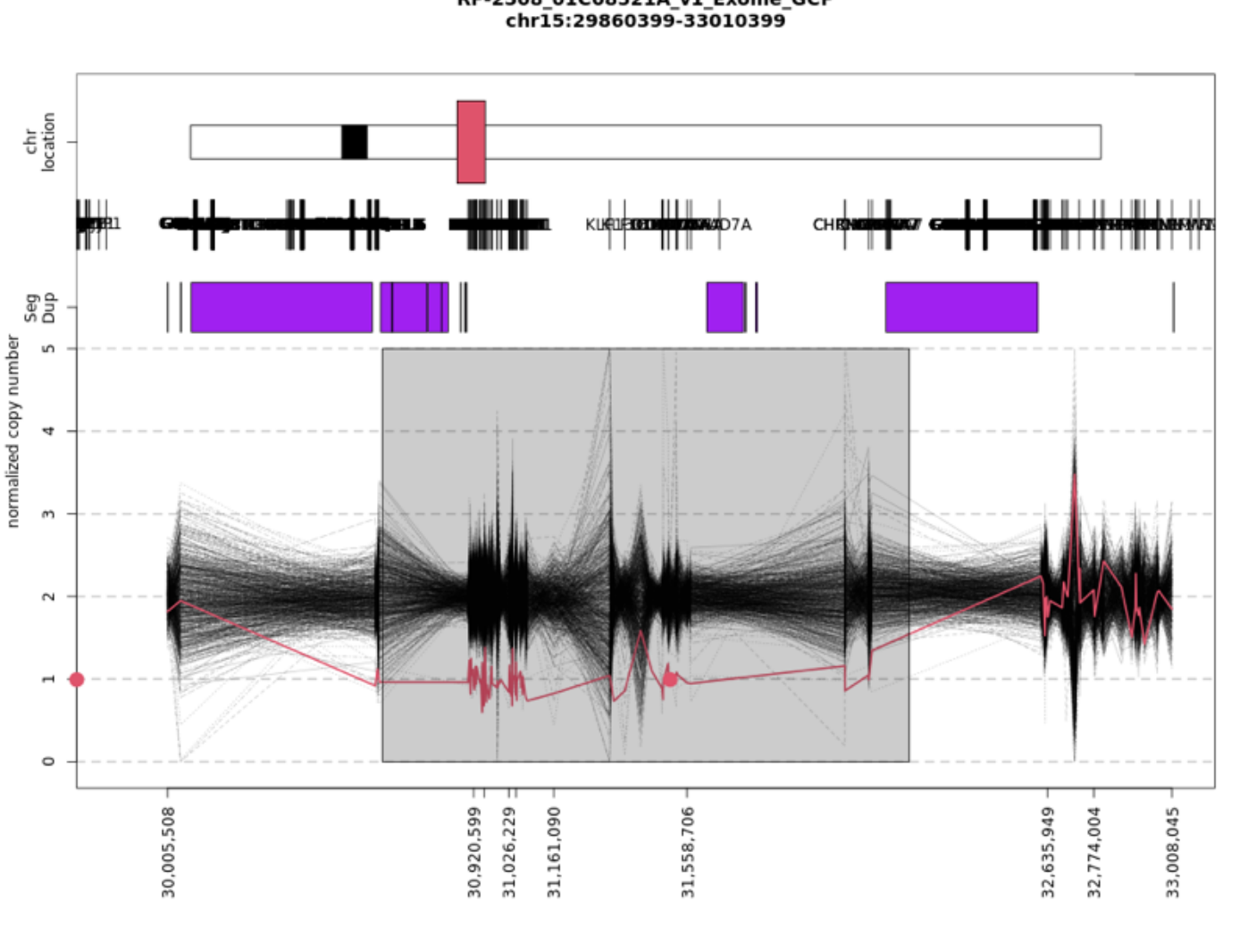
RP-2508_01C08521A_v1_Exome_GCP
chr15:29860399-33010399
chr location
Gene
Seg Dup
normalized copy number
30,005,508
30,920,599
31,026,229
31,161,090
31,558,706
32,635,949
32,774,004
33,008,045

RP-2508_01C06651A_v1_Exome_GCP
chr1:146159940-149084732

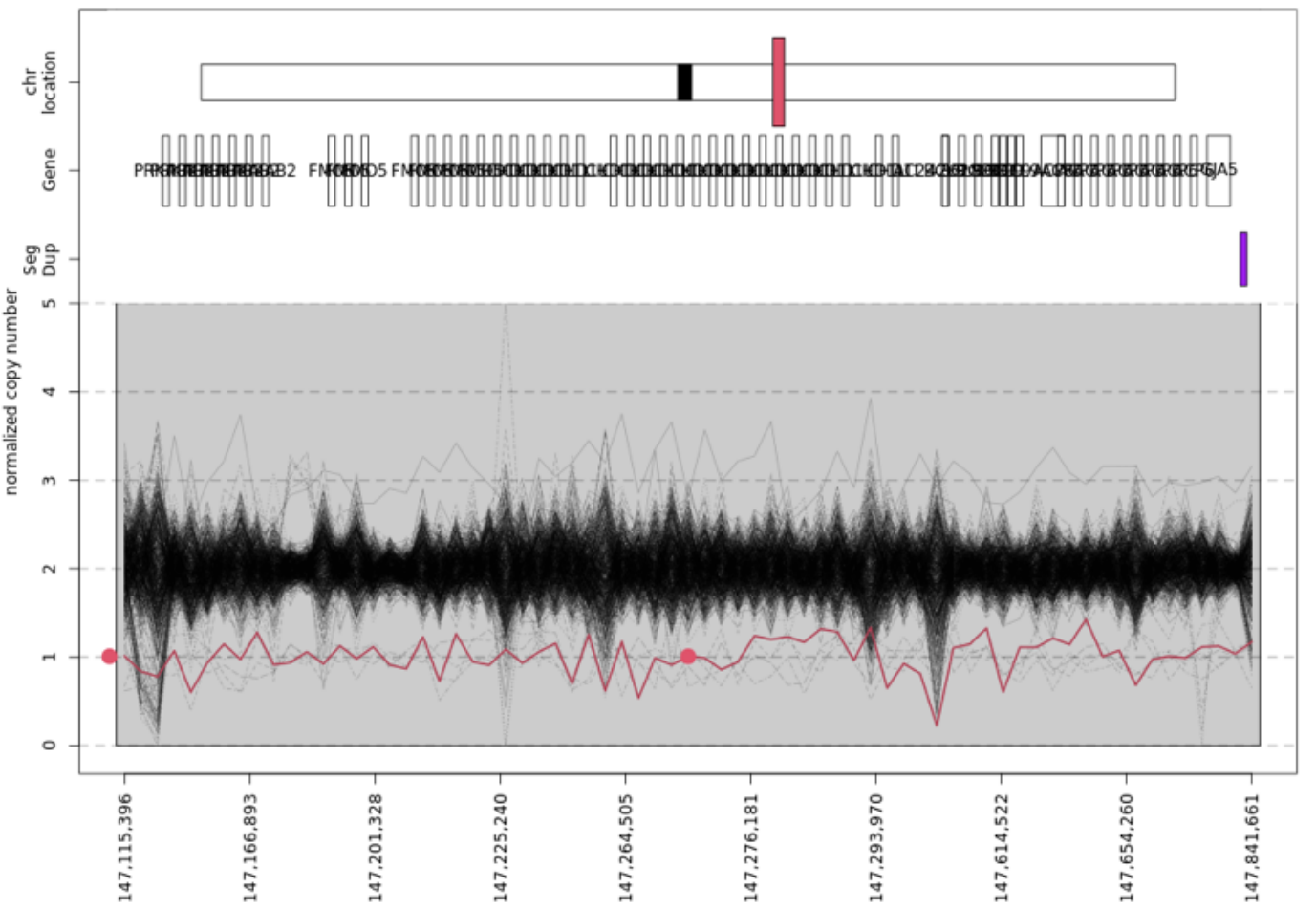

RP-2508_01C06651A_v1_Exome_GCP
chr1:146159940-149084732

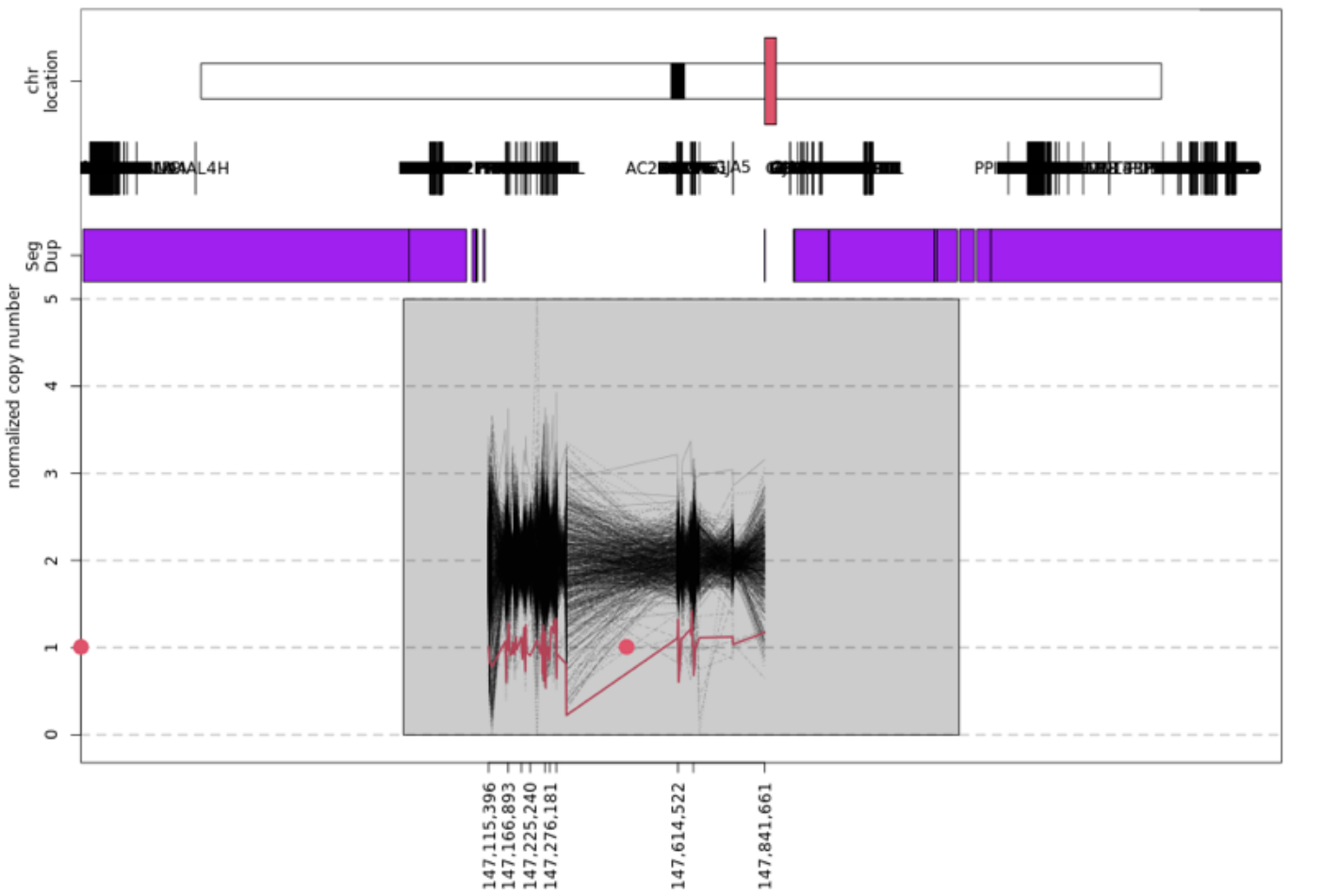

RP-2508_01C08372A_v1_Exome_GCP
chr15:21047512-30710983

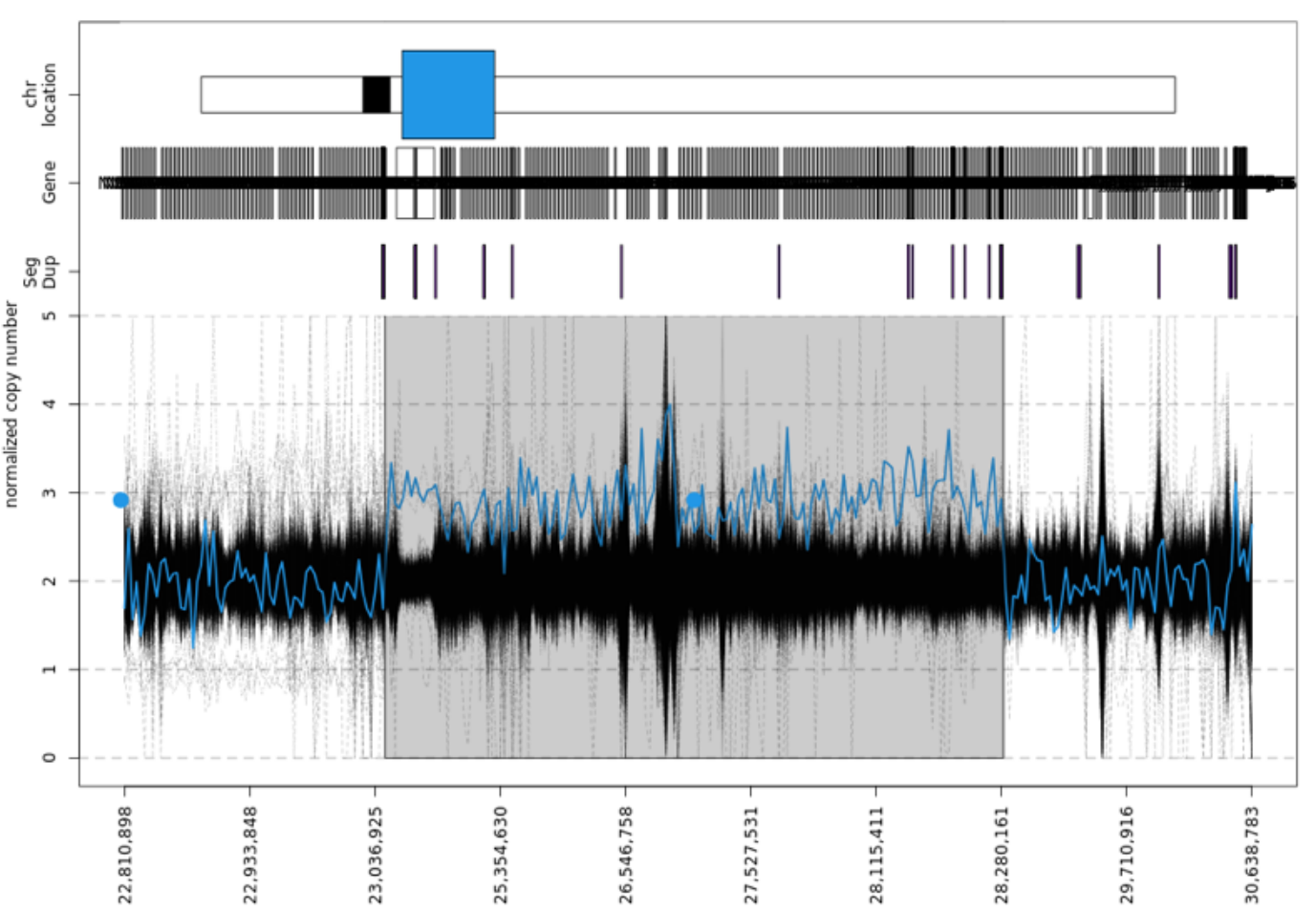

RP-2508_01C08372A_v1_Exome_GCP
chr15:21047512-30710983

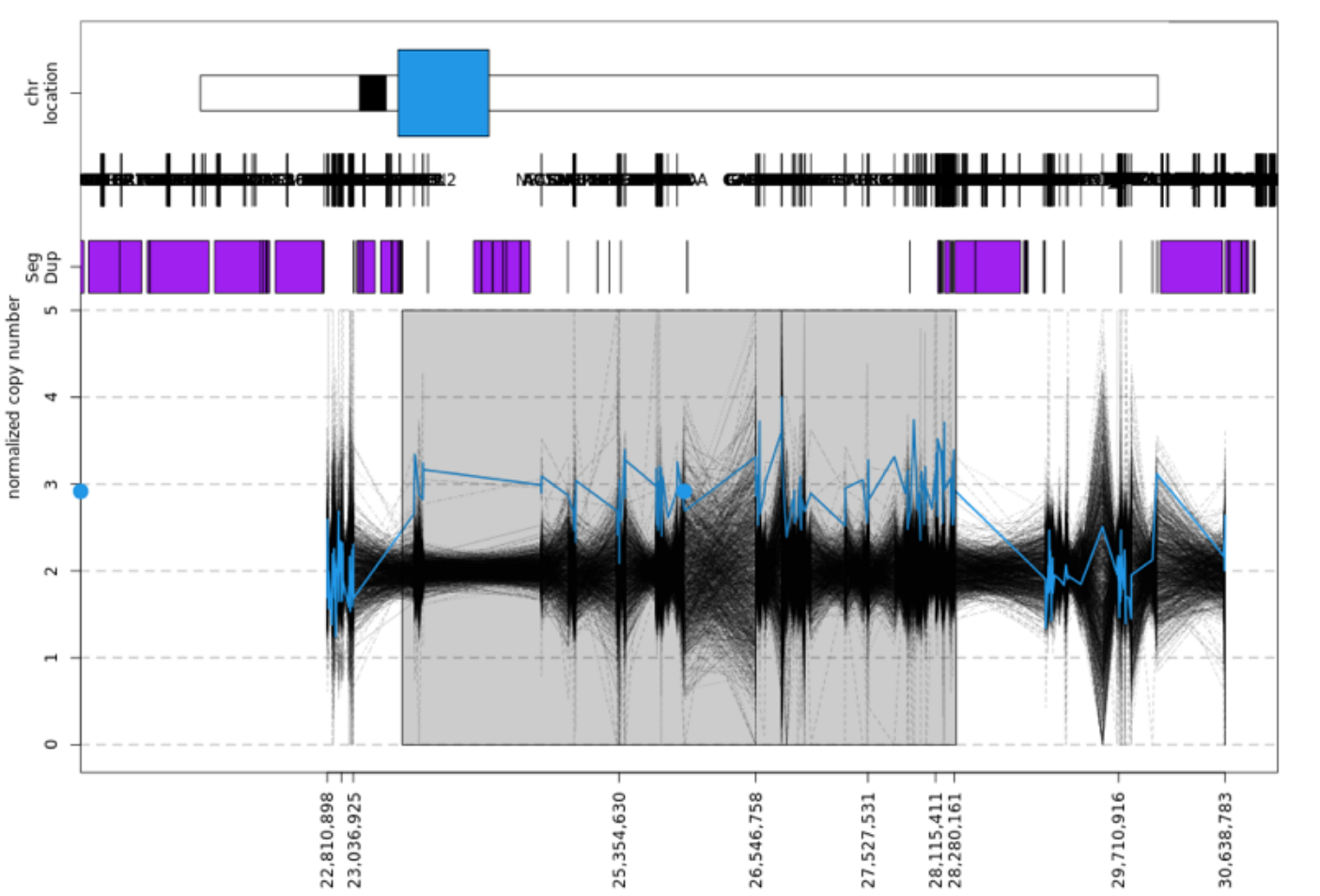

RP-2508_01C08375A_v1_Exome_GCP
chr15:21076204-30701419

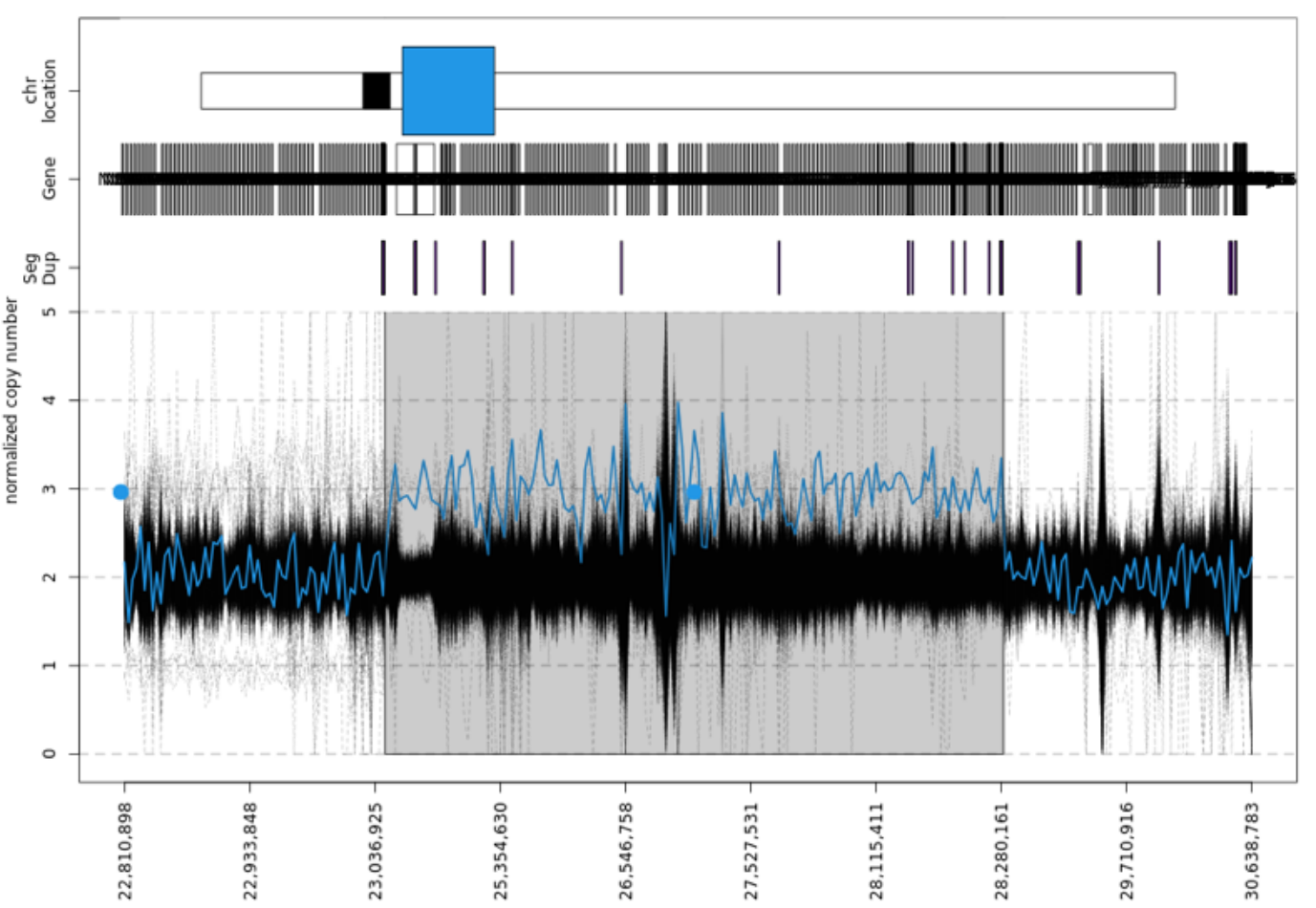

RP-2508_01C08375A_v1_Exome_GCP
chr15:21076204-30701419

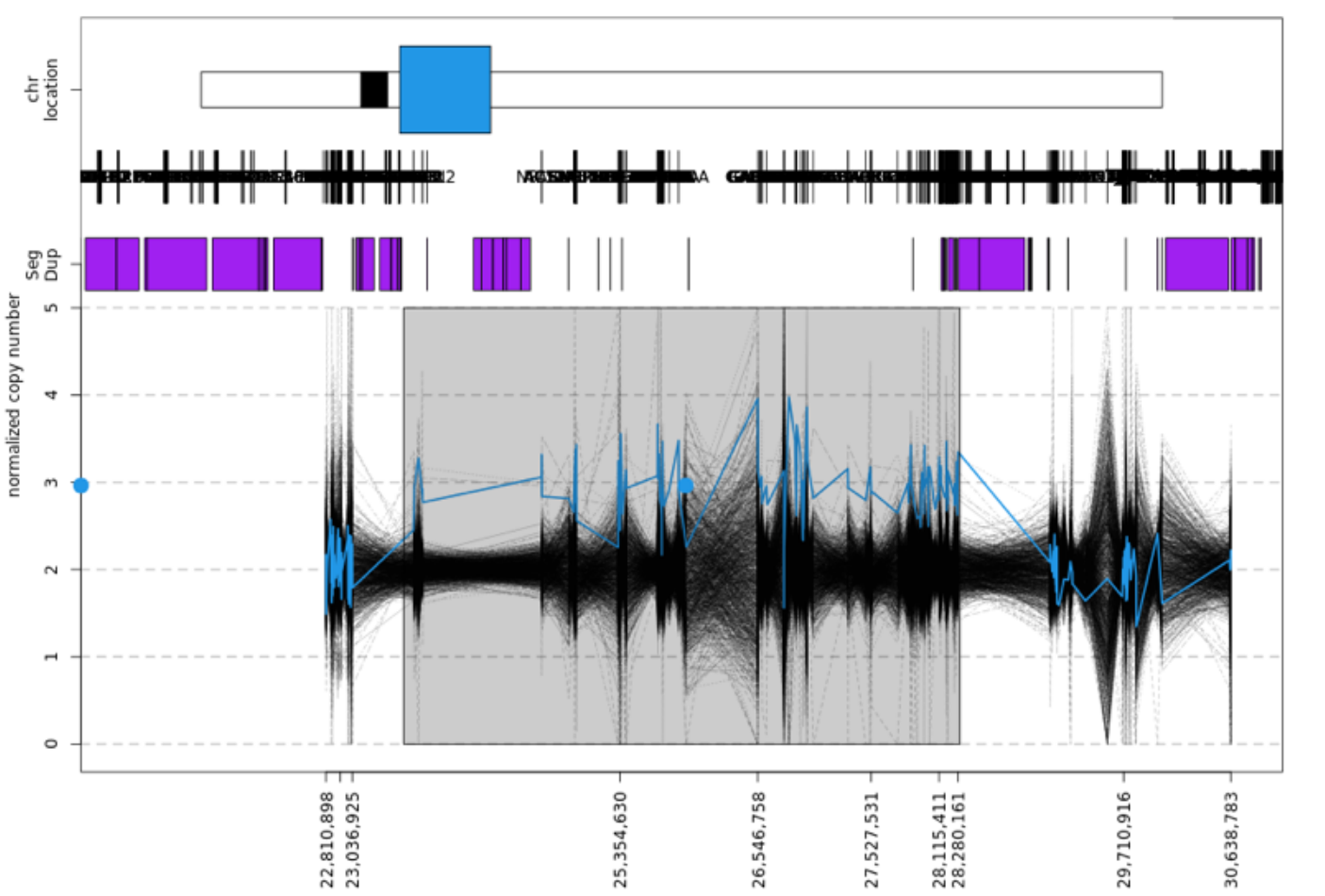

RP-2508_01C06609A_v1_Exome_GCP
chr1:146159940-149084732

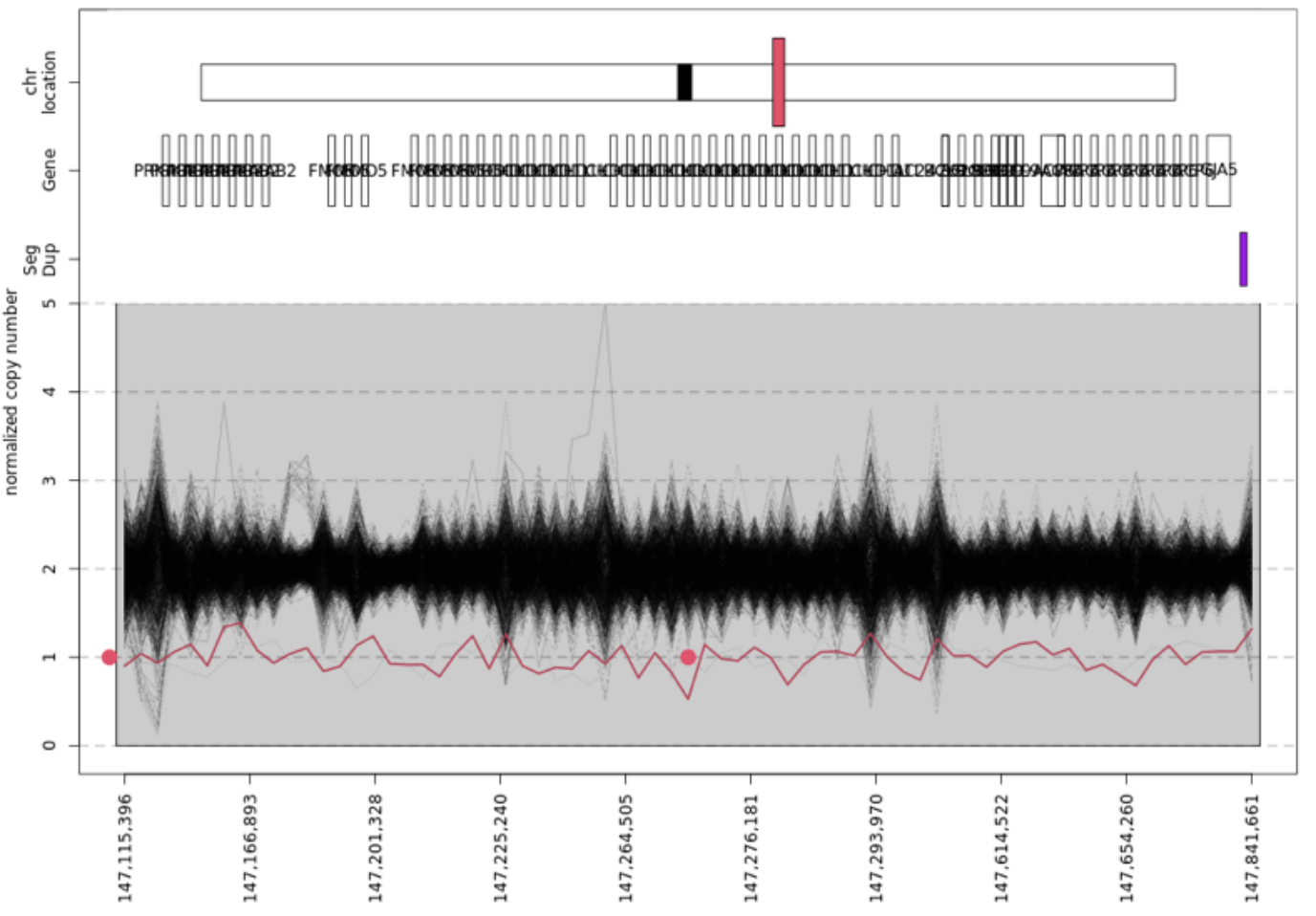

RP-2508_01C06609A_v1_Exome_GCP
chr1:146159940-149084732

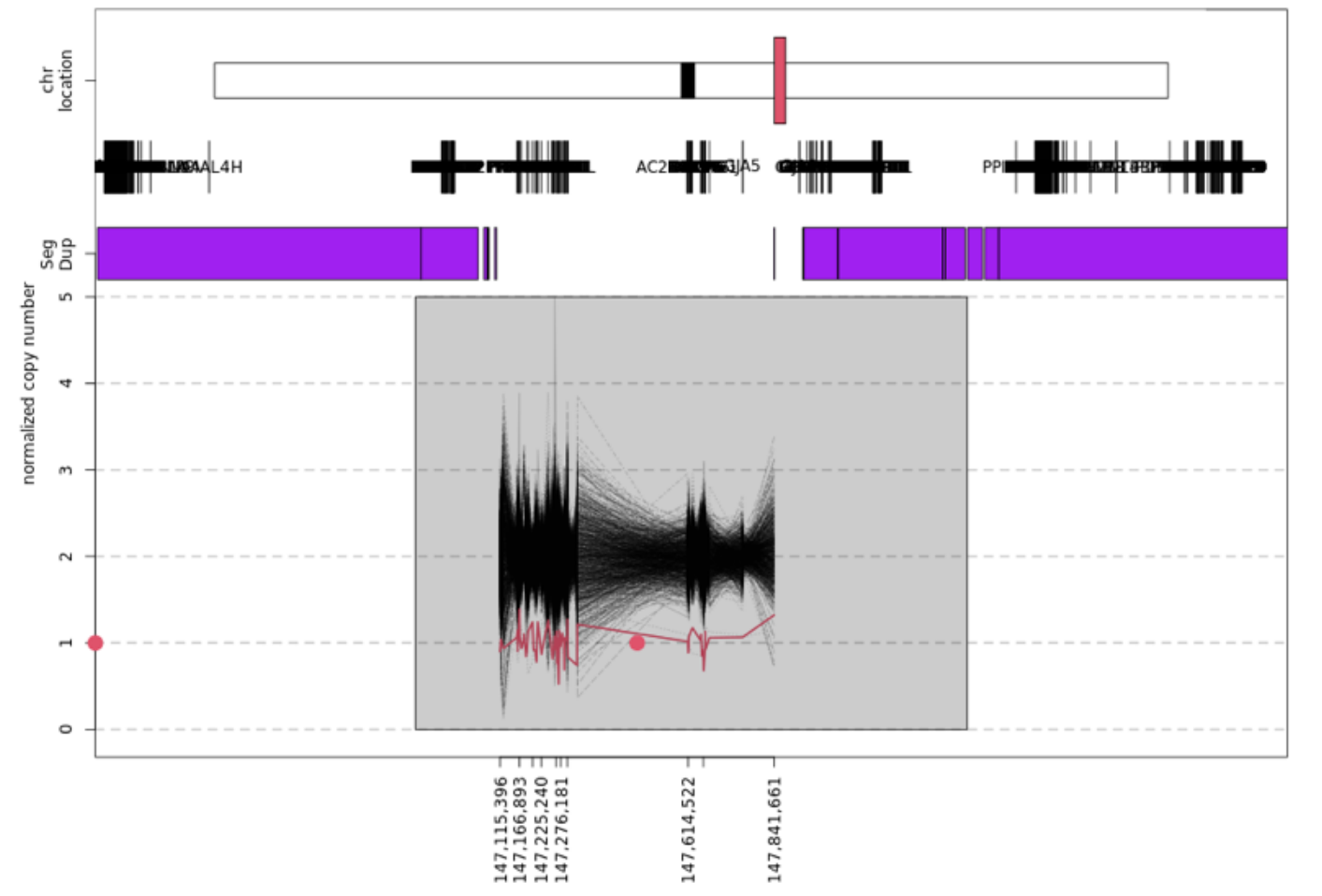

RP-2508_01C06610A_v1_Exome_GCP
chr1:146159940-149084732

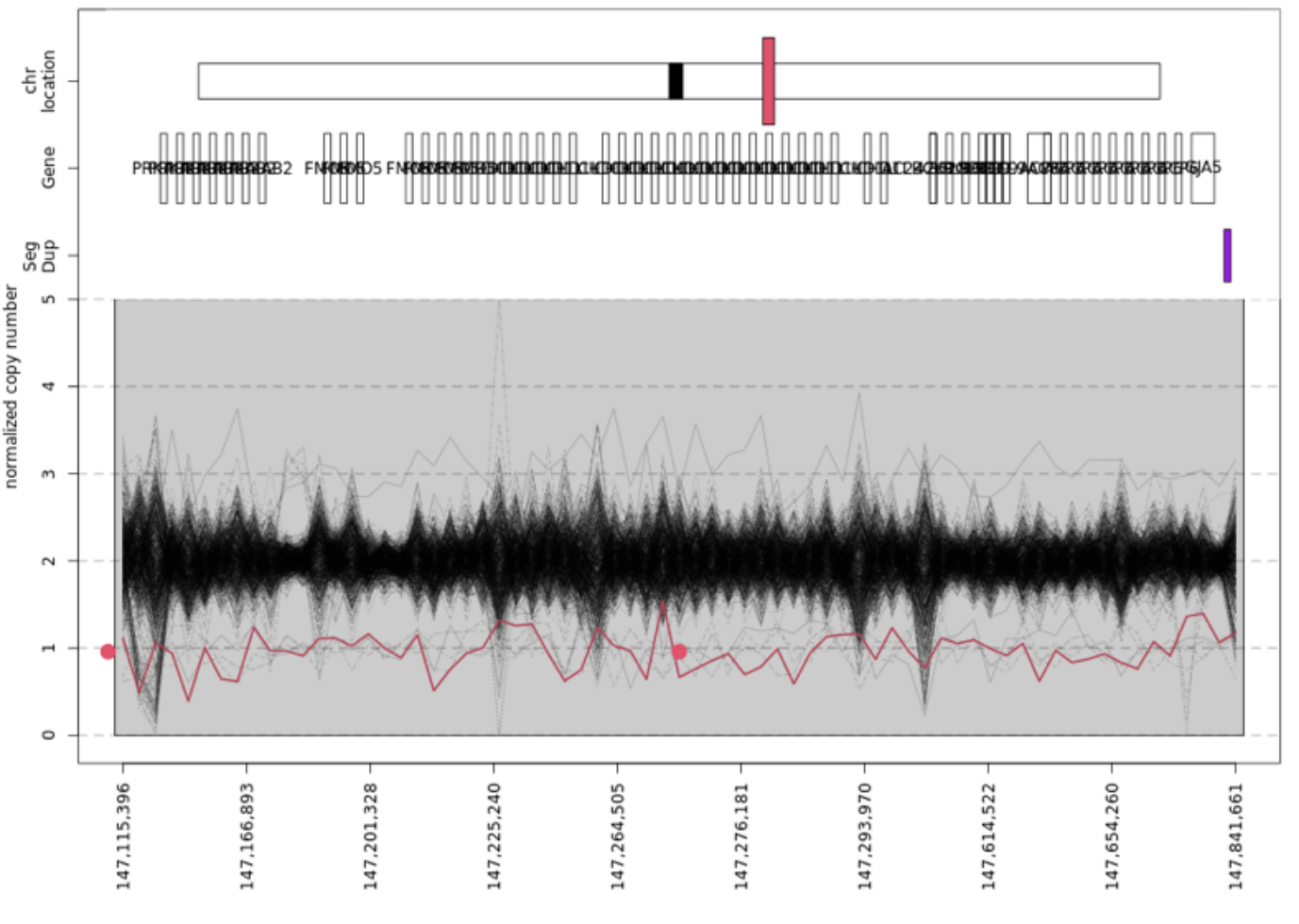

RP-2508_01C06610A_v1_Exome_GCP
chr1:146159940-149084732

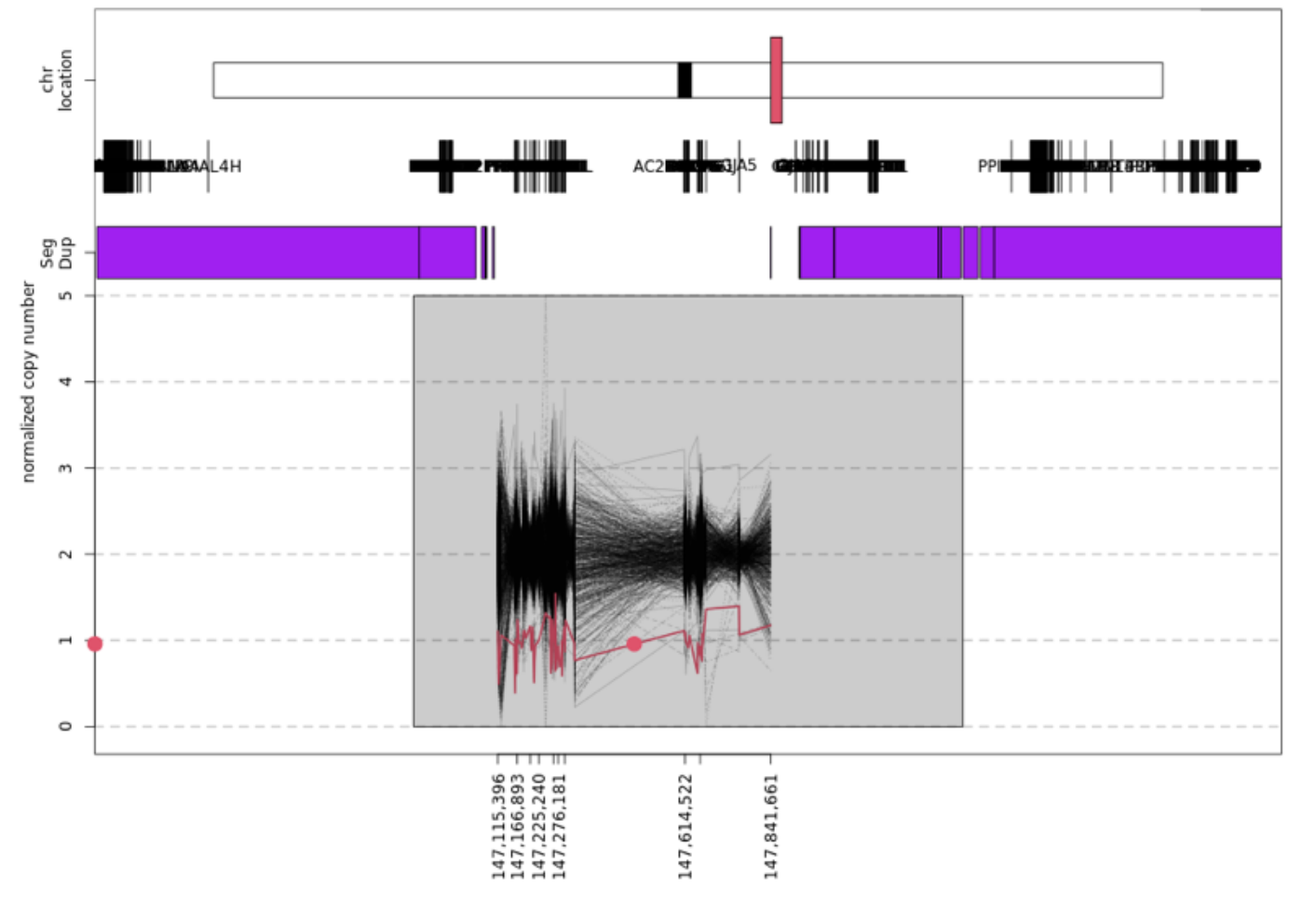

RP-2508_01C06611B_v1_Exome_GCP
chr1:146159940-149084732

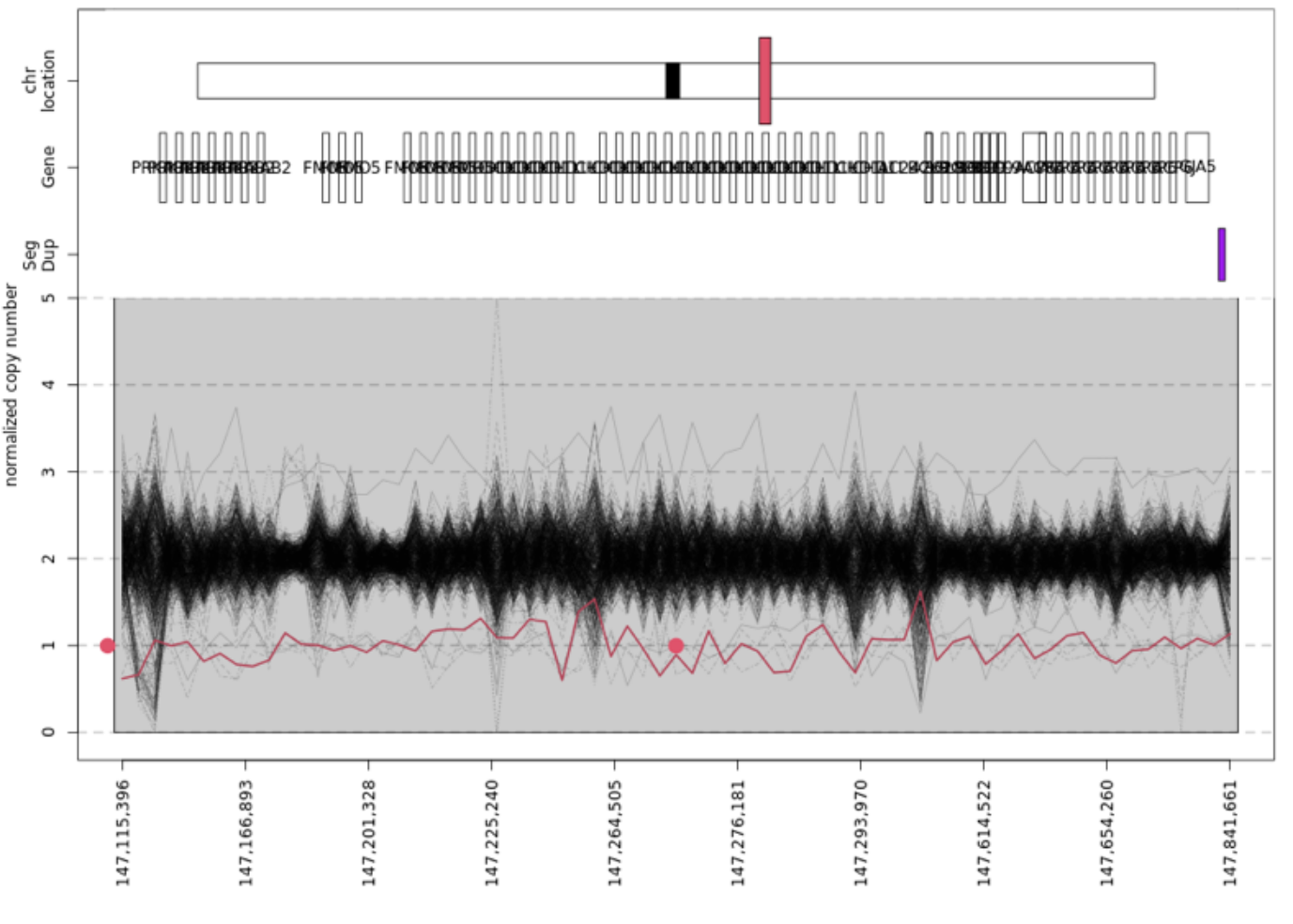

RP-2508_01C06611B_v1_Exome_GCP
chr1:146159940-149084732

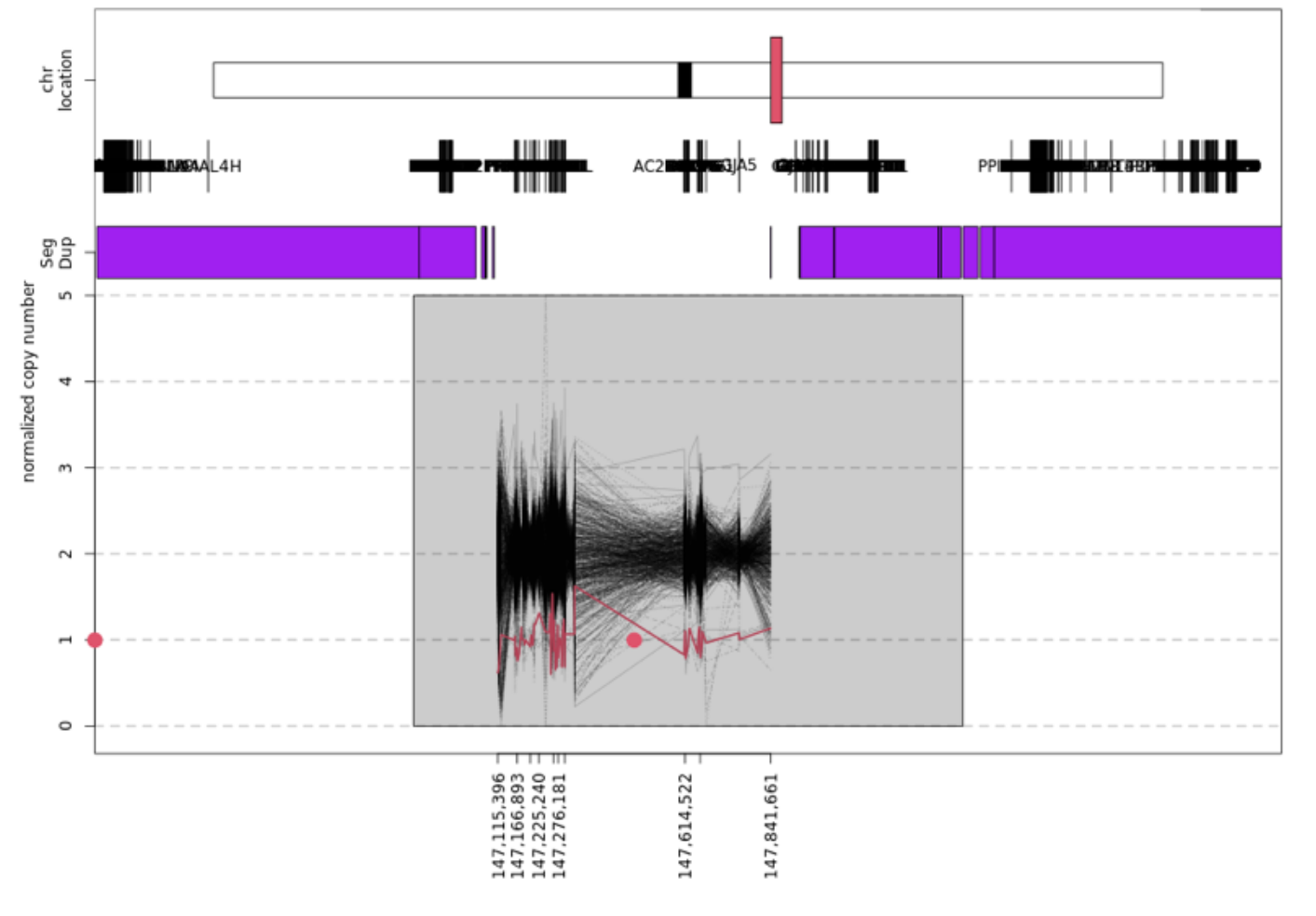

MH0130258
chr1:146367925-149015404

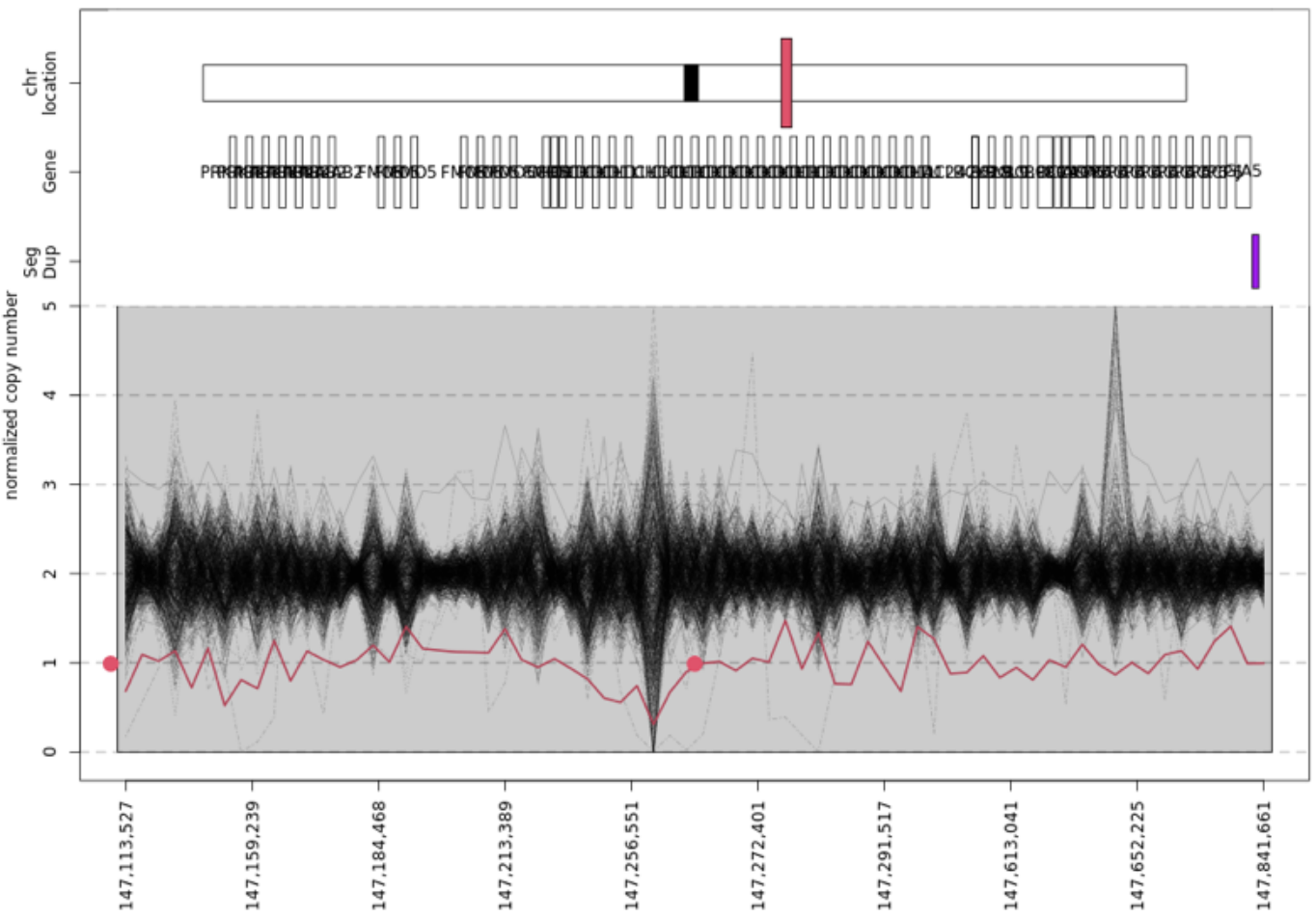

MH0130258
chr1:146367925-149015404

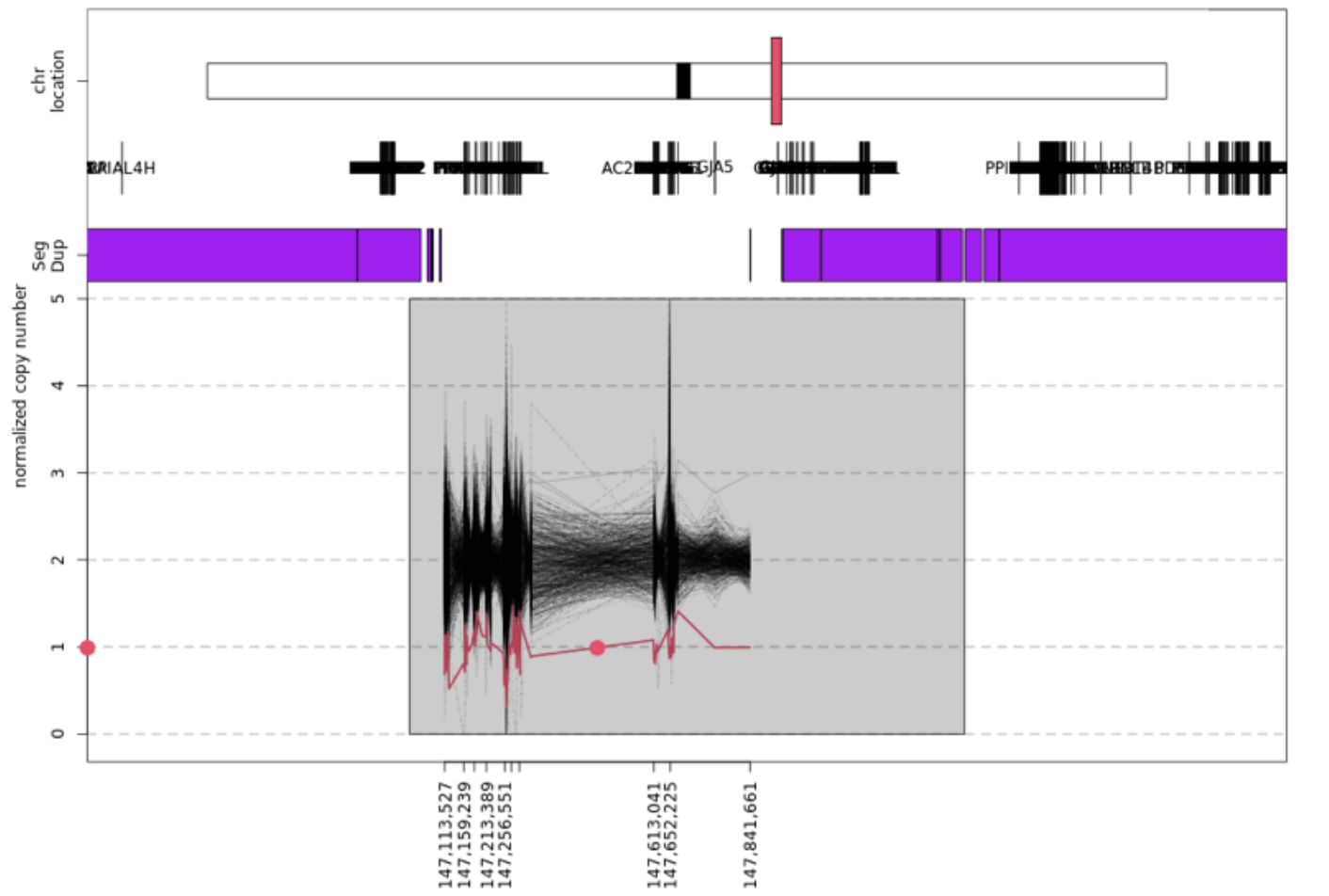

MH0130259
chr1:146166256-149082627

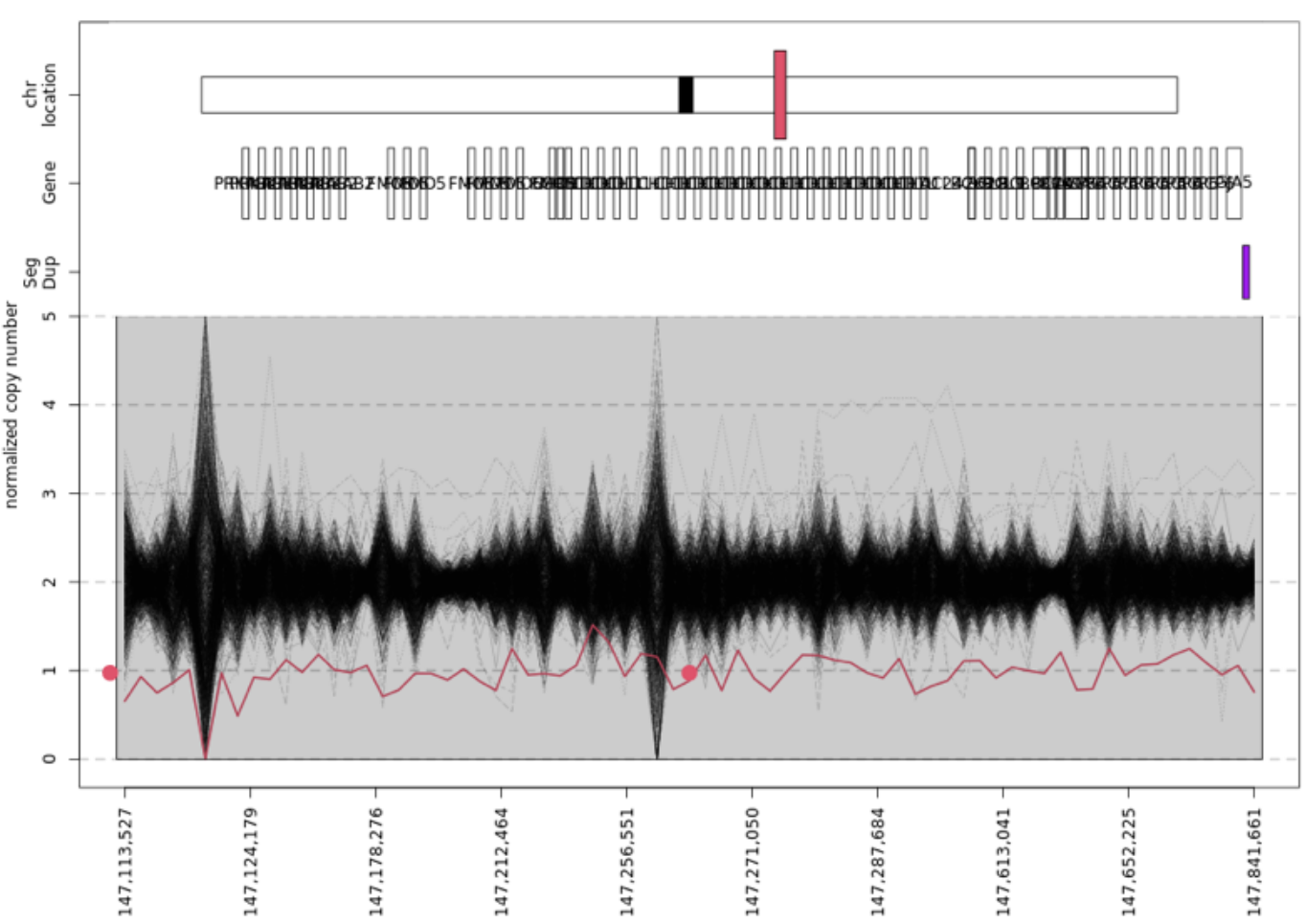

MH0130259
chr1:146166256-149082627

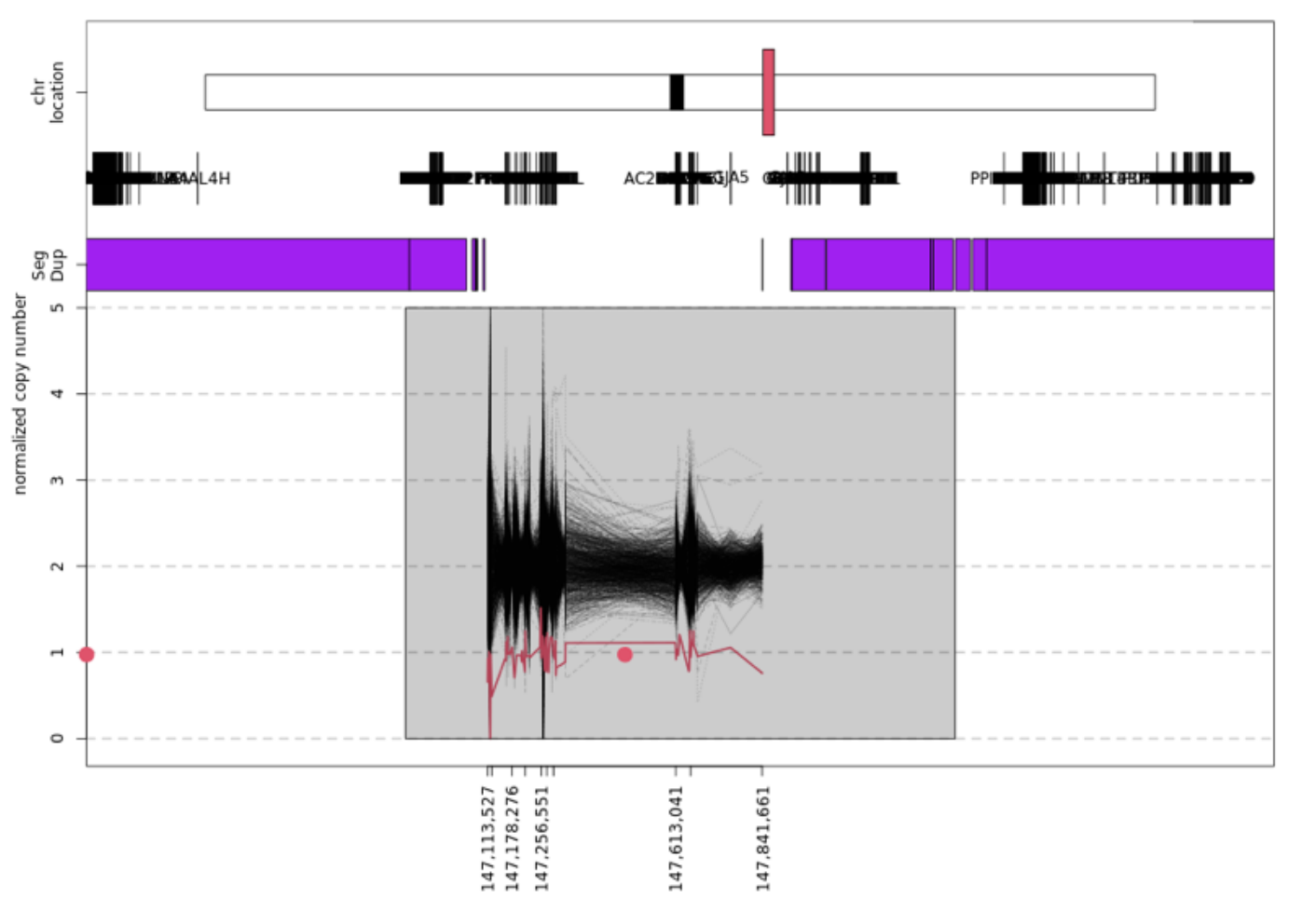

MH0143033
chr15:19998505-31096737

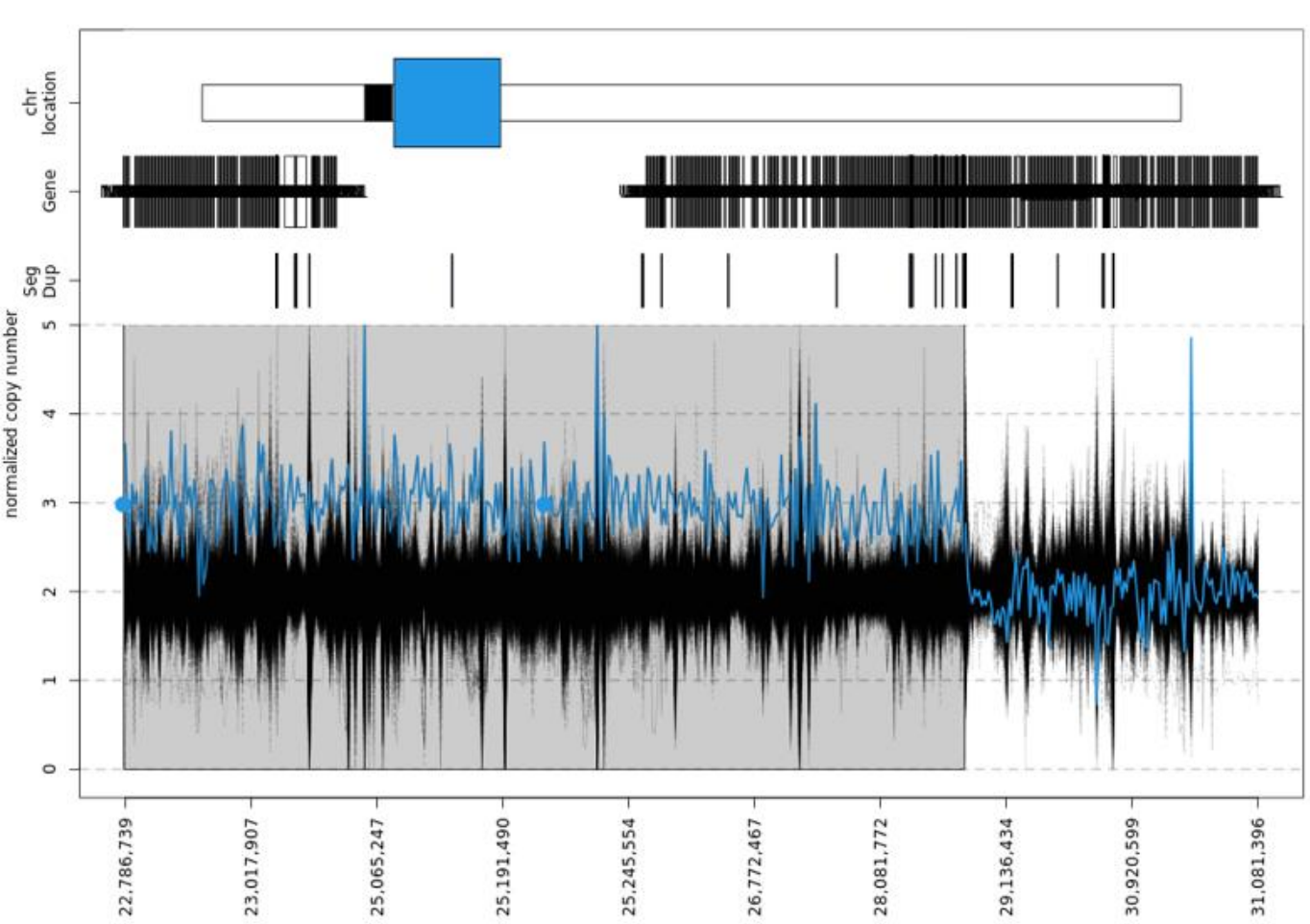

MH0143033
chr15:19998505-31096737

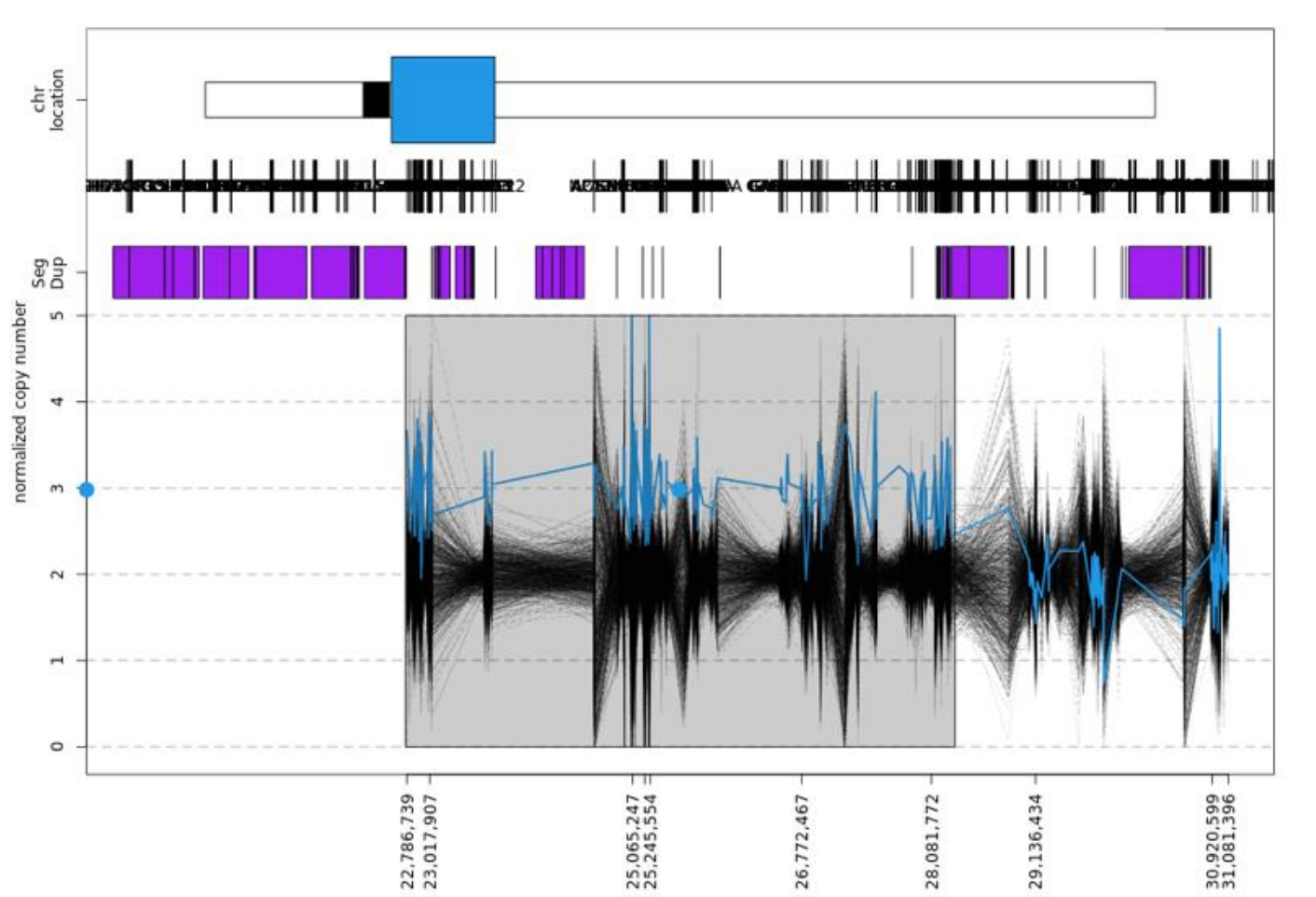

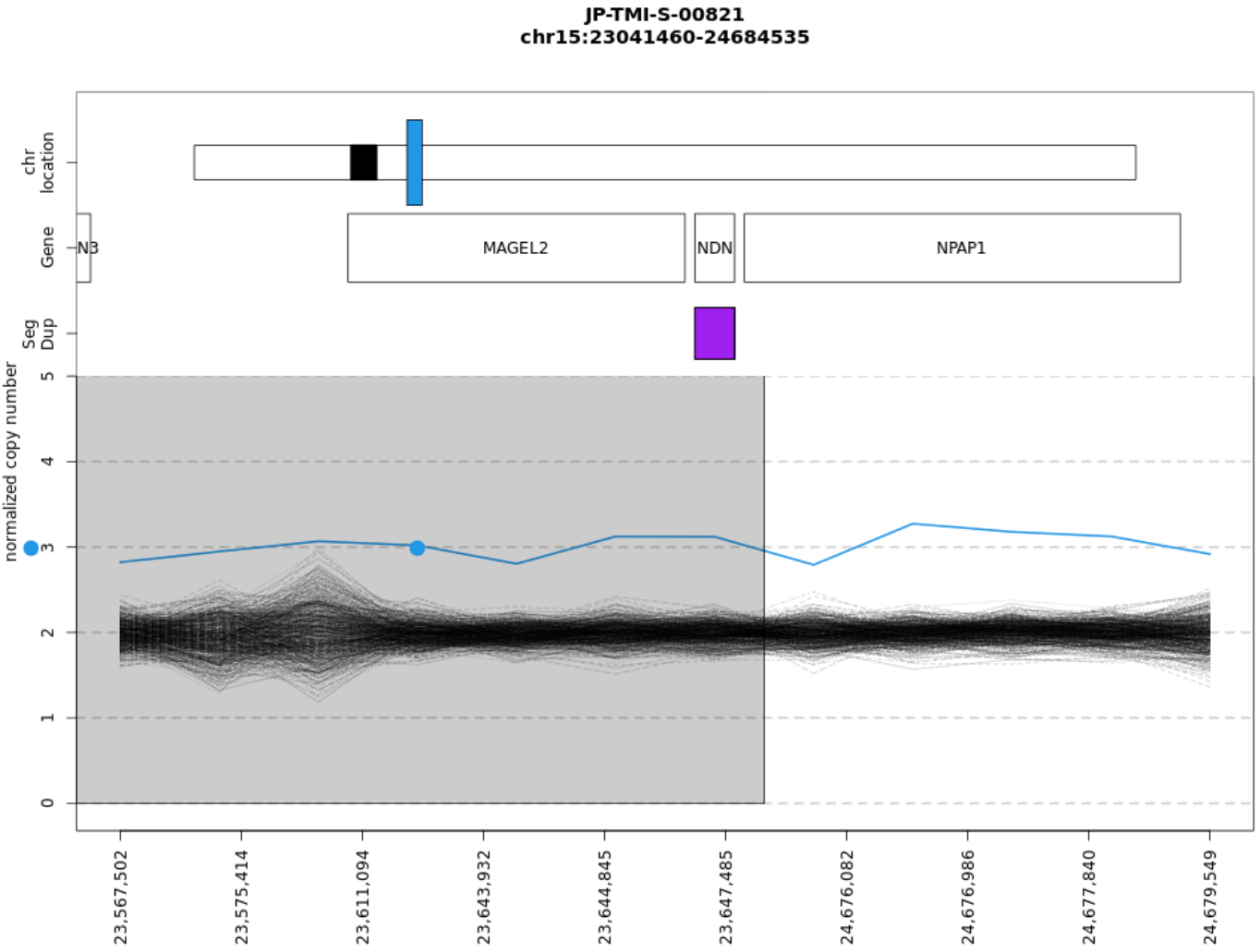
JP-TMI-S-00821
chr15:23041460-24684535
chr location
Gene
MAGEL2
NDN
NPAP1
Seg Dup
normalized copy number
0
1
2
3
4
5
23,567,502
23,575,414
23,611,094
23,643,932
23,644,845
23,647,485
24,676,082
24,676,986
24,677,840
24,679,549

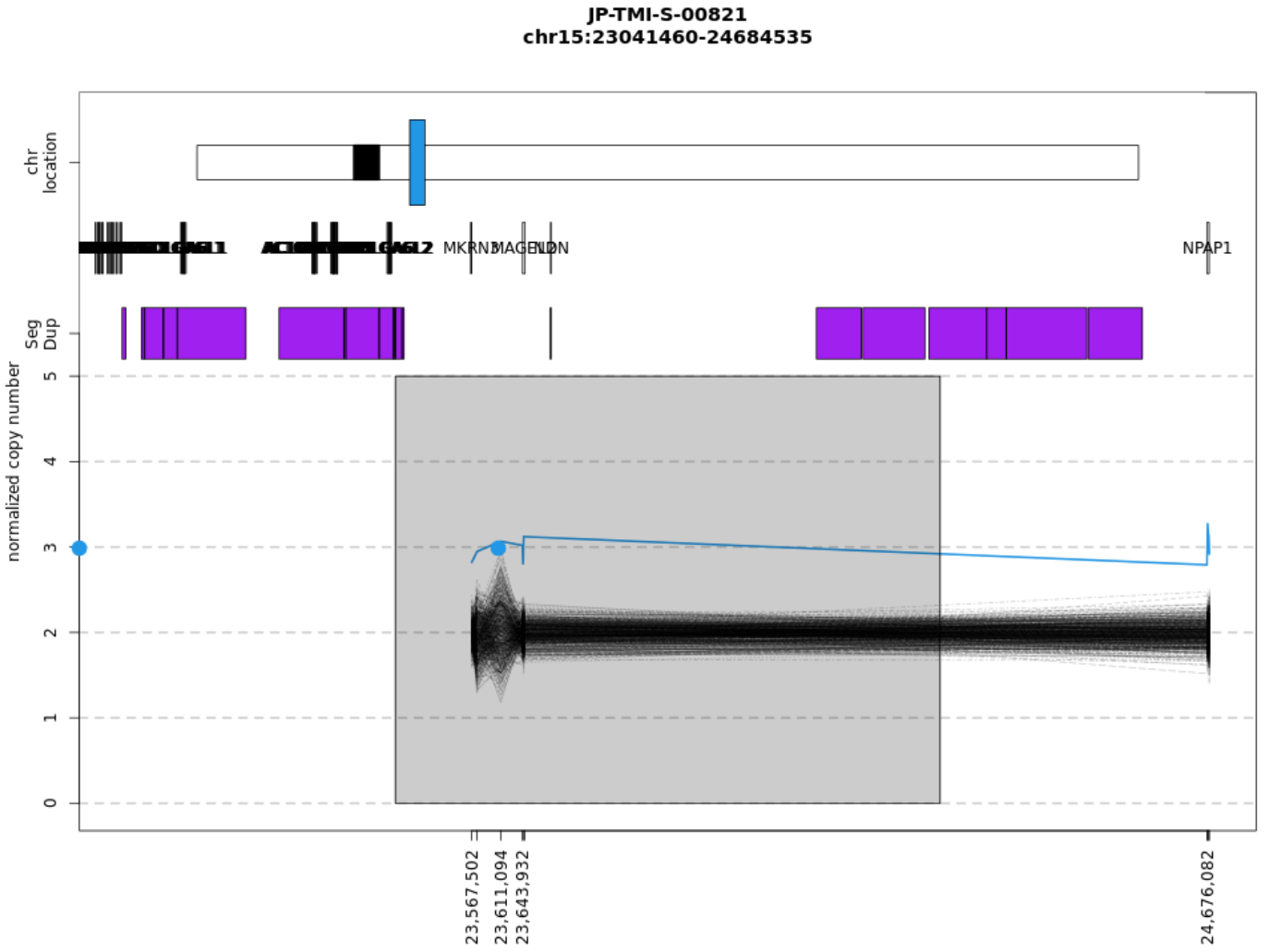
JP-TMI-S-00821
chr15:23041460-24684535
chr location
Gene
NPAP1
Seg Dup
normalized copy number
0
1
2
3
4
5
23,567,502
23,611,094
23,643,932
24,676,082

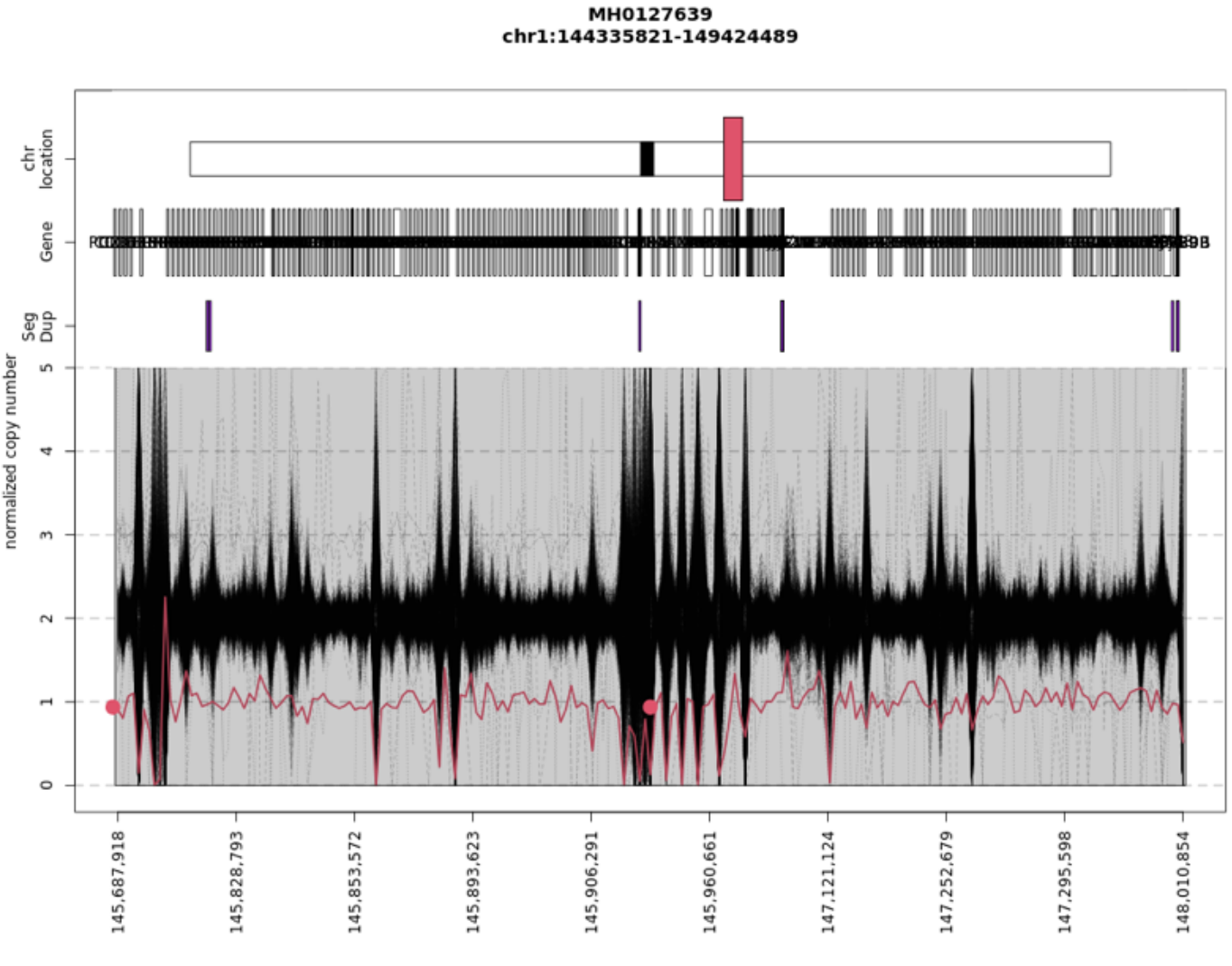
MH0127639
chr1:144335821-149424489
chr location
Gene
Seg Dup
normalized copy number
0
1
2
3
4
5
145,687,918
145,828,793
145,853,572
145,893,623
145,906,291
145,960,661
147,121,124
147,252,679
147,295,598
148,010,854

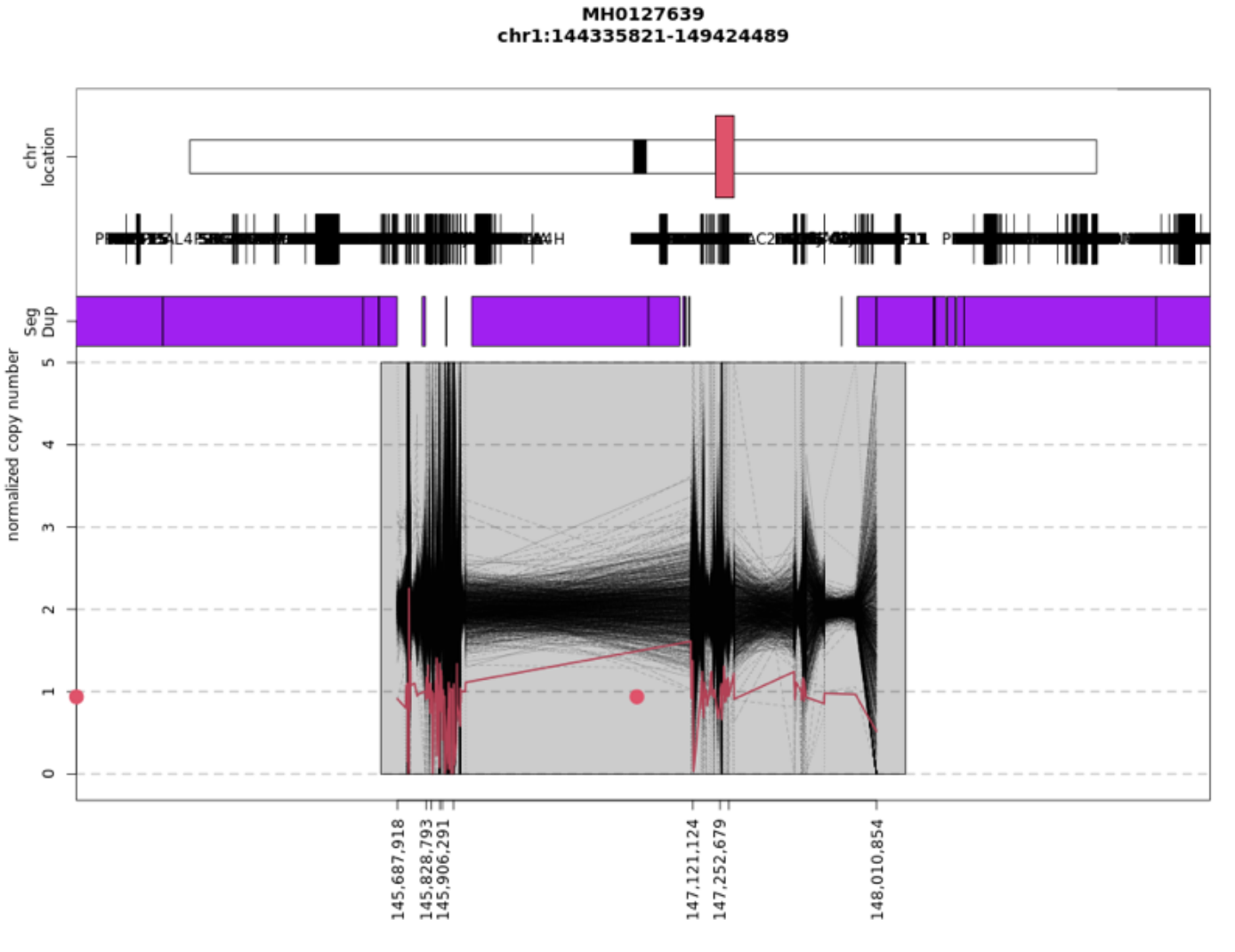
MH0127639
chr1:144335821-149424489
chr location
Gene
Seg Dup
normalized copy number
0
1
2
3
4
5
145,687,918
145,828,793
145,906,291
147,121,124
147,252,679
148,010,854

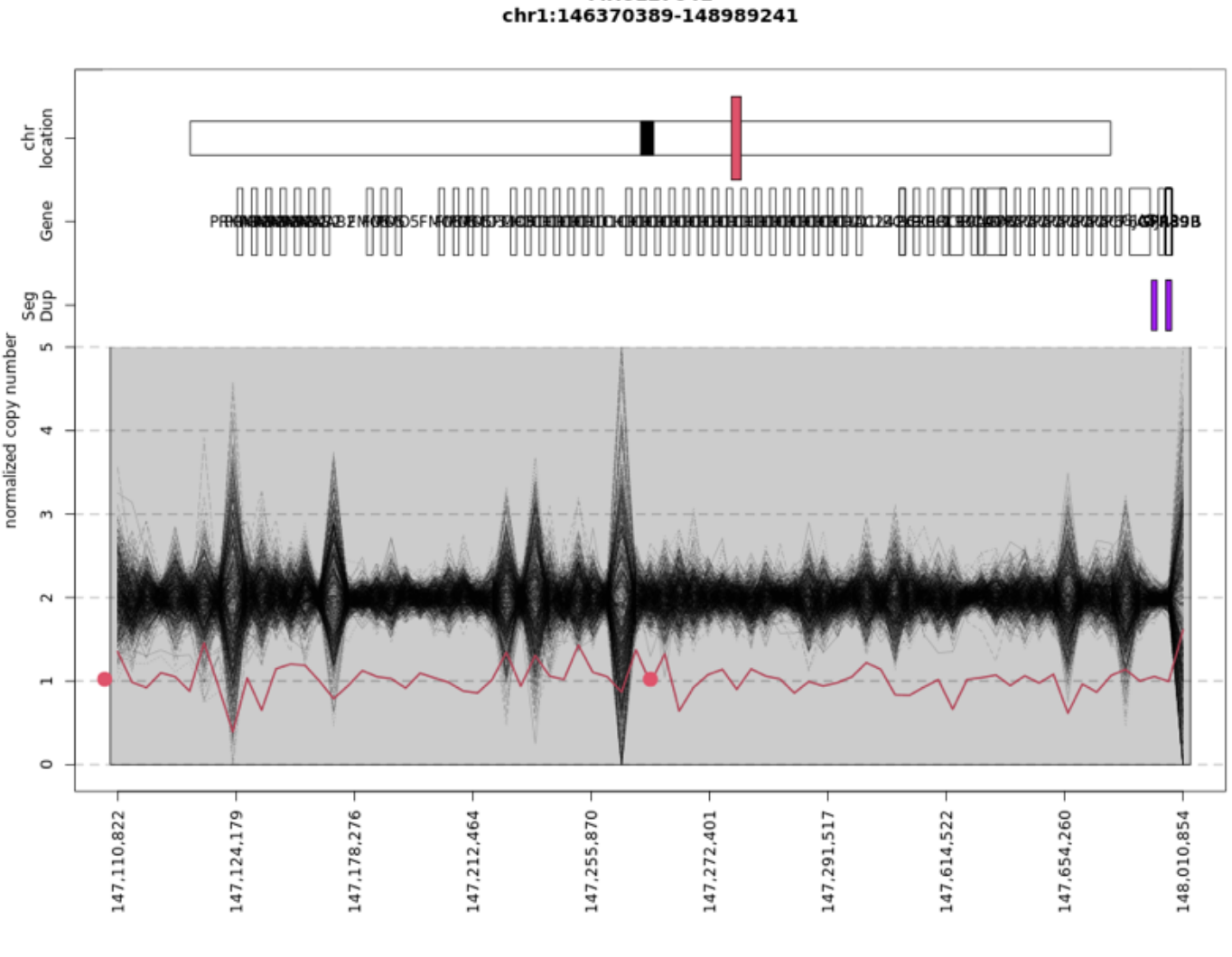
MH0127641
chr1:146370389-148989241
chr location
Gene
Seg Dup
normalized copy number
0
1
2
3
4
5
147,110,822
147,124,179
147,178,276
147,212,464
147,255,870
147,272,401
147,291,517
147,614,522
147,654,260
148,010,854

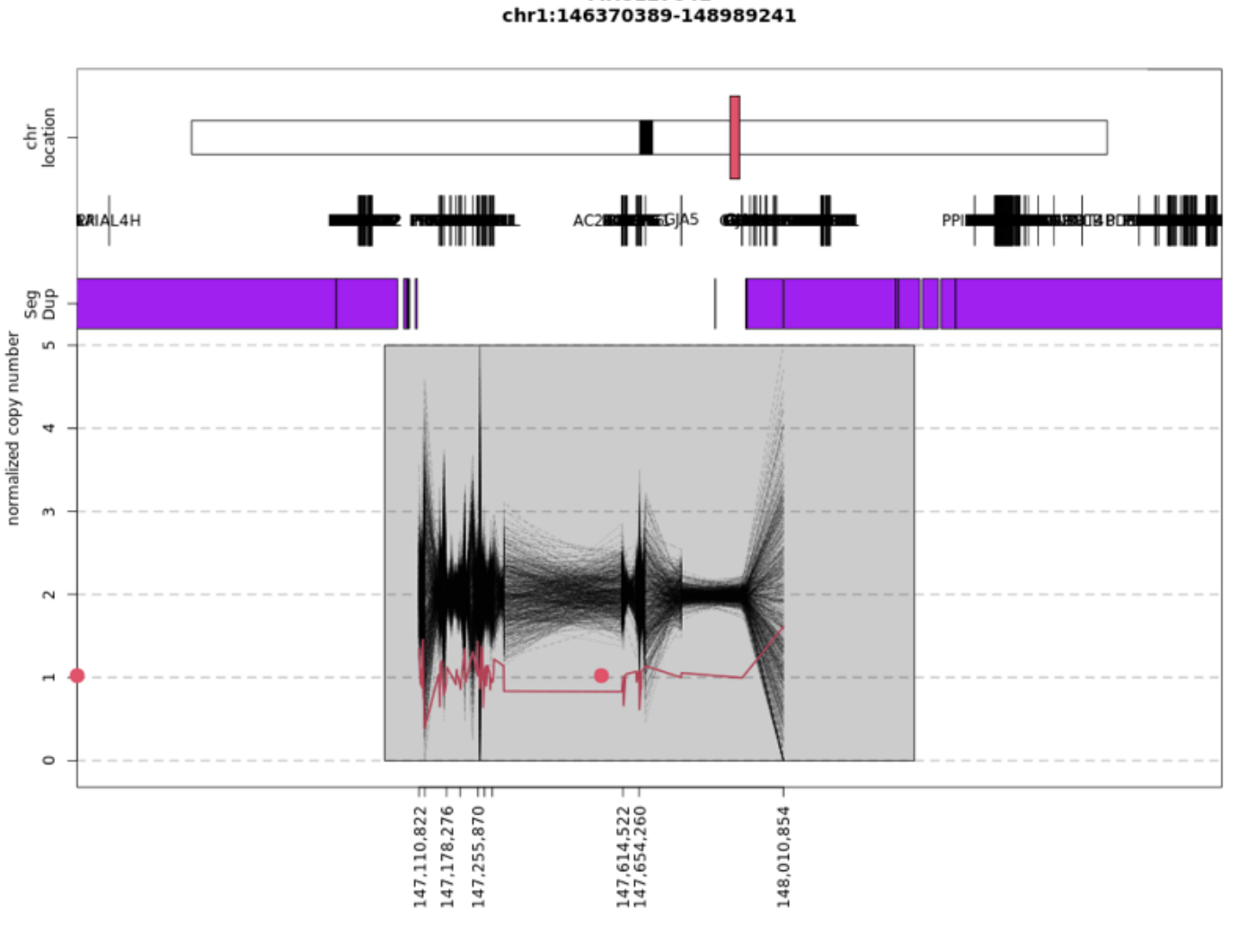
MH0127641
chr1:146370389-148989241
chr location
Gene
Seg Dup
normalized copy number
0
1
2
3
4
5
147,110,822
147,178,276
147,255,870
147,614,522
147,654,260
148,010,854

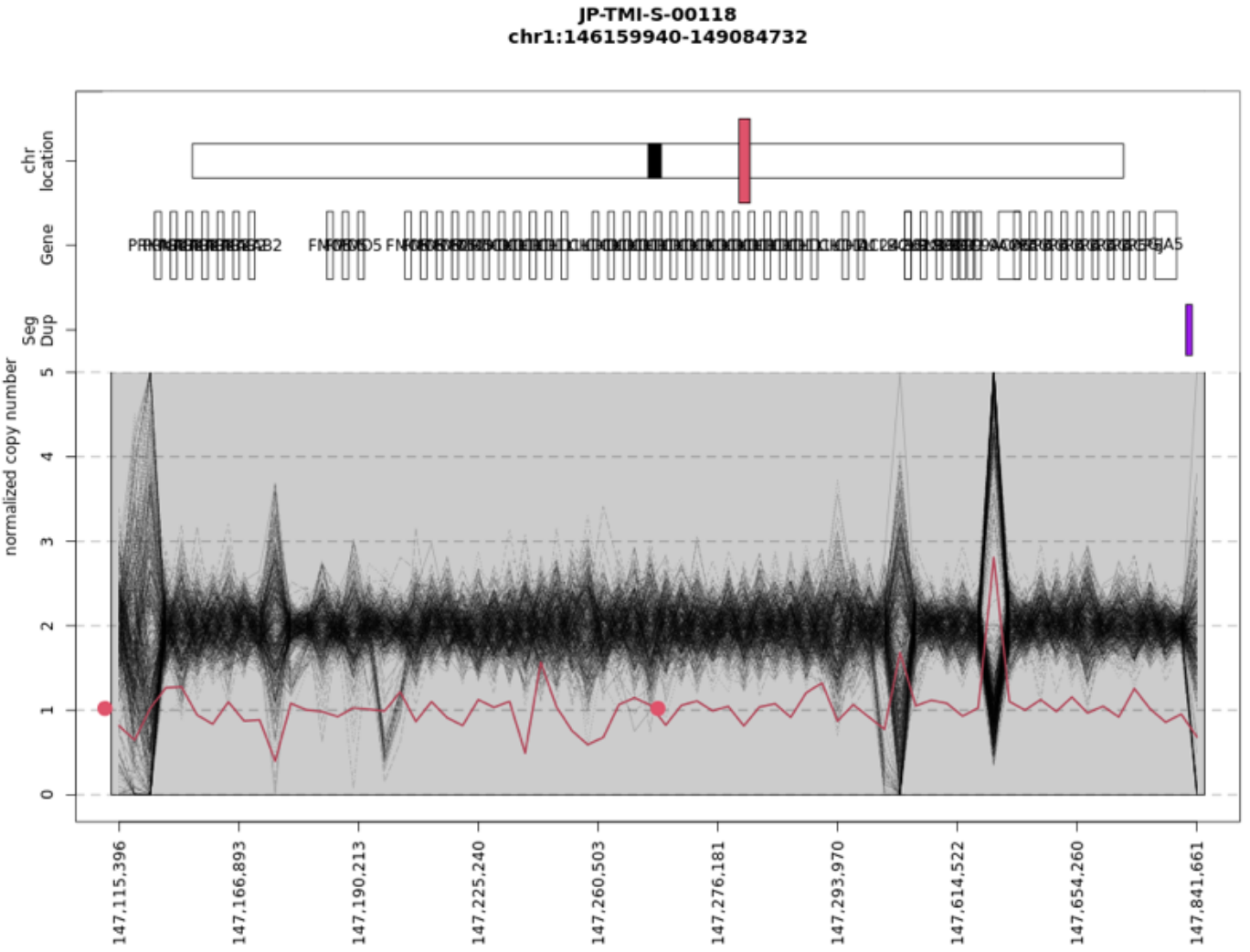
JP-TMI-S-00118
chr1:146159940-149084732
chr location
Gene
Seg Dup
normalized copy number
147,115,396
147,166,893
147,190,213
147,225,240
147,260,503
147,276,181
147,293,970
147,614,522
147,654,260
147,841,661

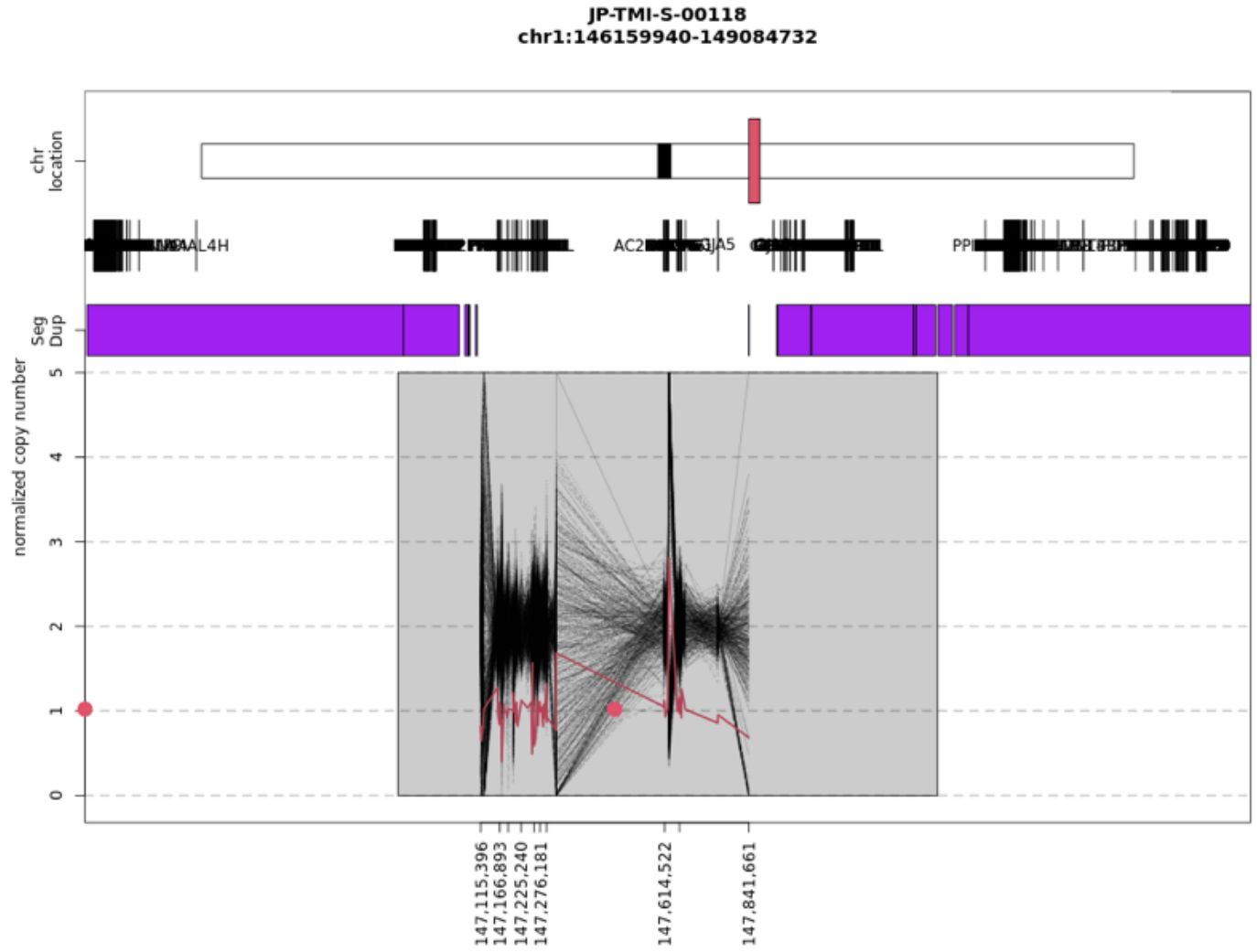
JP-TMI-S-00118
chr1:146159940-149084732
chr location
Gene
Seg Dup
normalized copy number
147,115,396
147,166,893
147,225,240
147,276,181
147,614,522
147,841,661

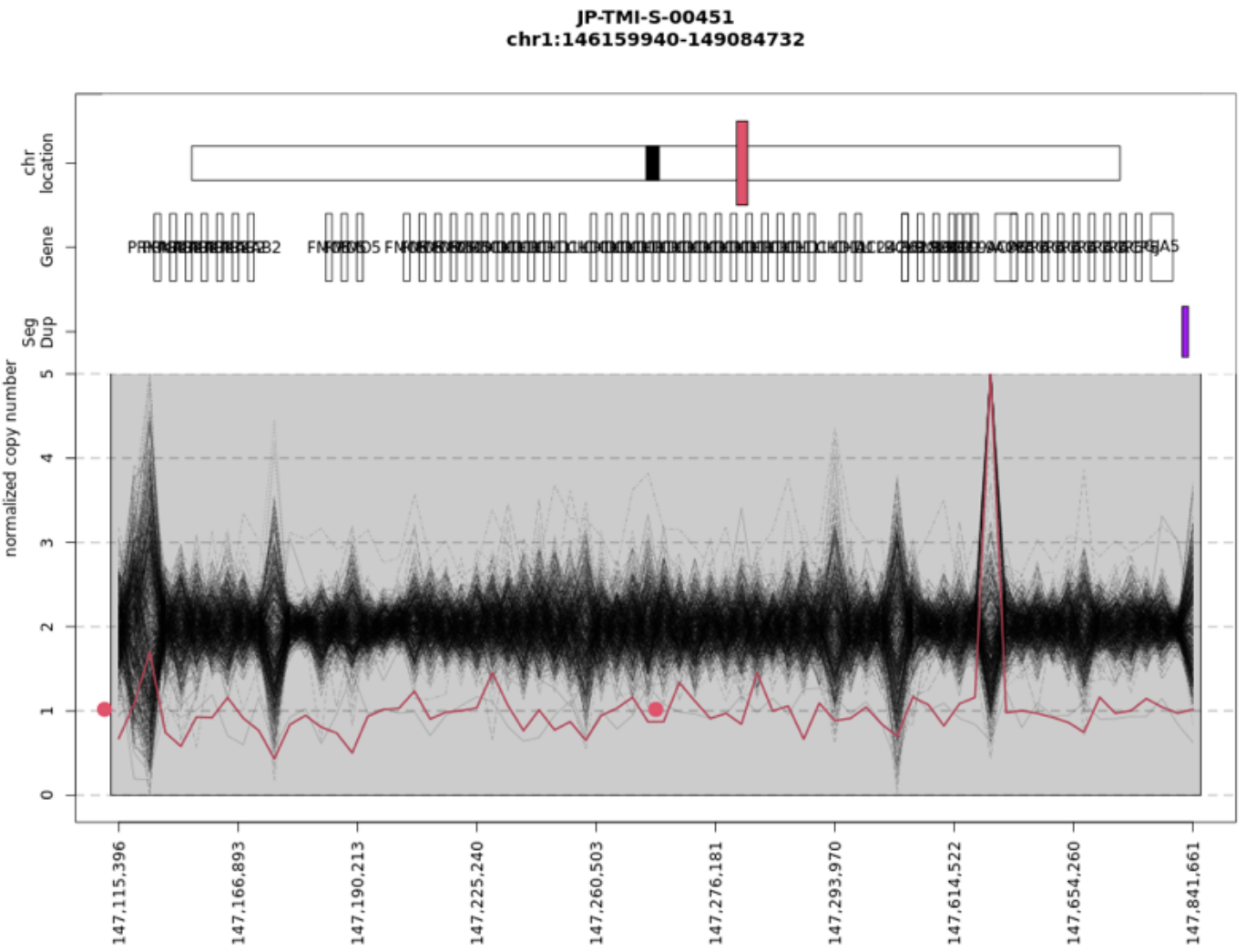
JP-TMI-S-00451
chr1:146159940-149084732
chr location
Gene
Seg Dup
normalized copy number
147,115,396
147,166,893
147,190,213
147,225,240
147,260,503
147,276,181
147,293,970
147,614,522
147,654,260
147,841,661

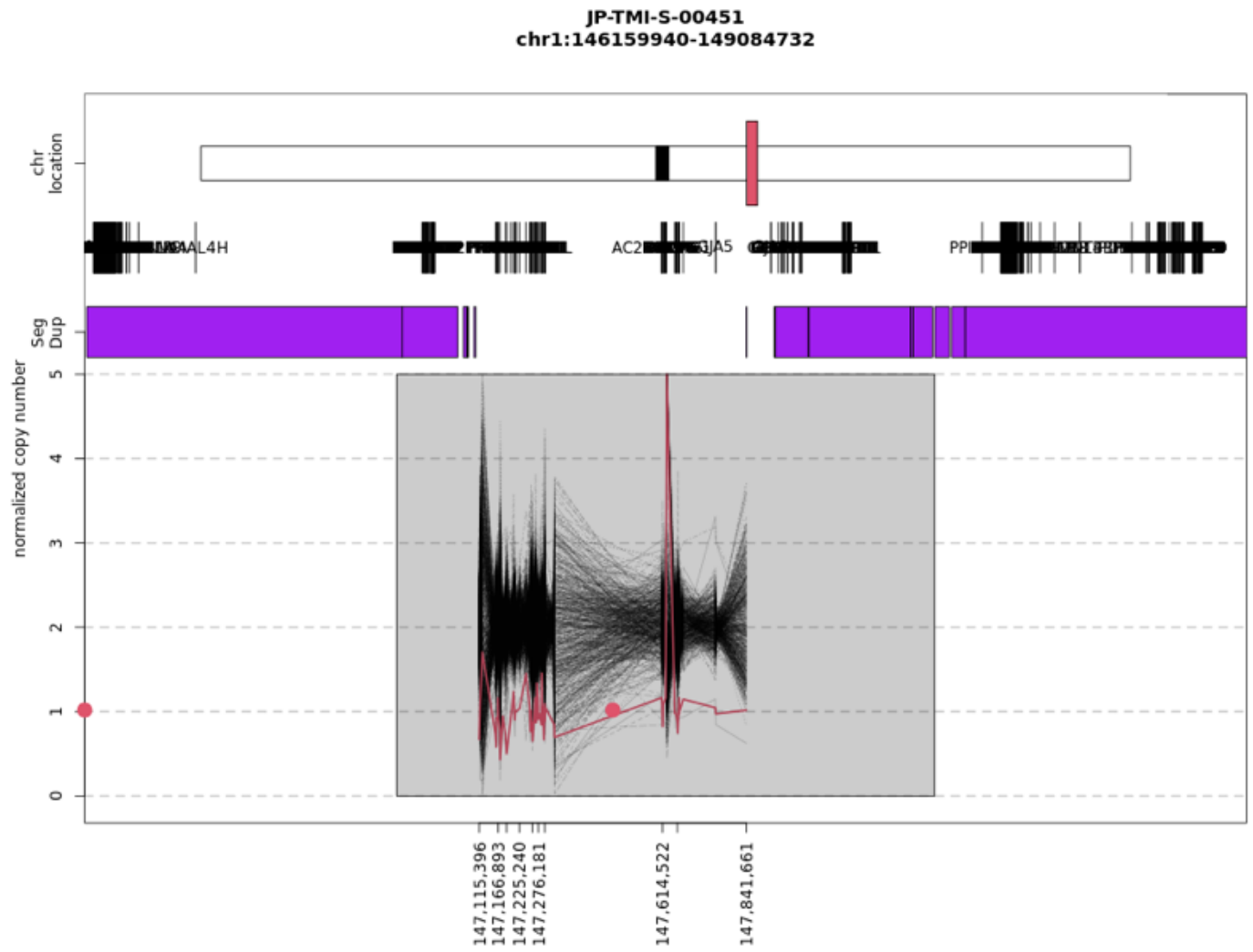
JP-TMI-S-00451
chr1:146159940-149084732
chr location
Gene
Seg Dup
normalized copy number
147,115,396
147,166,893
147,225,240
147,276,181
147,614,522
147,841,661

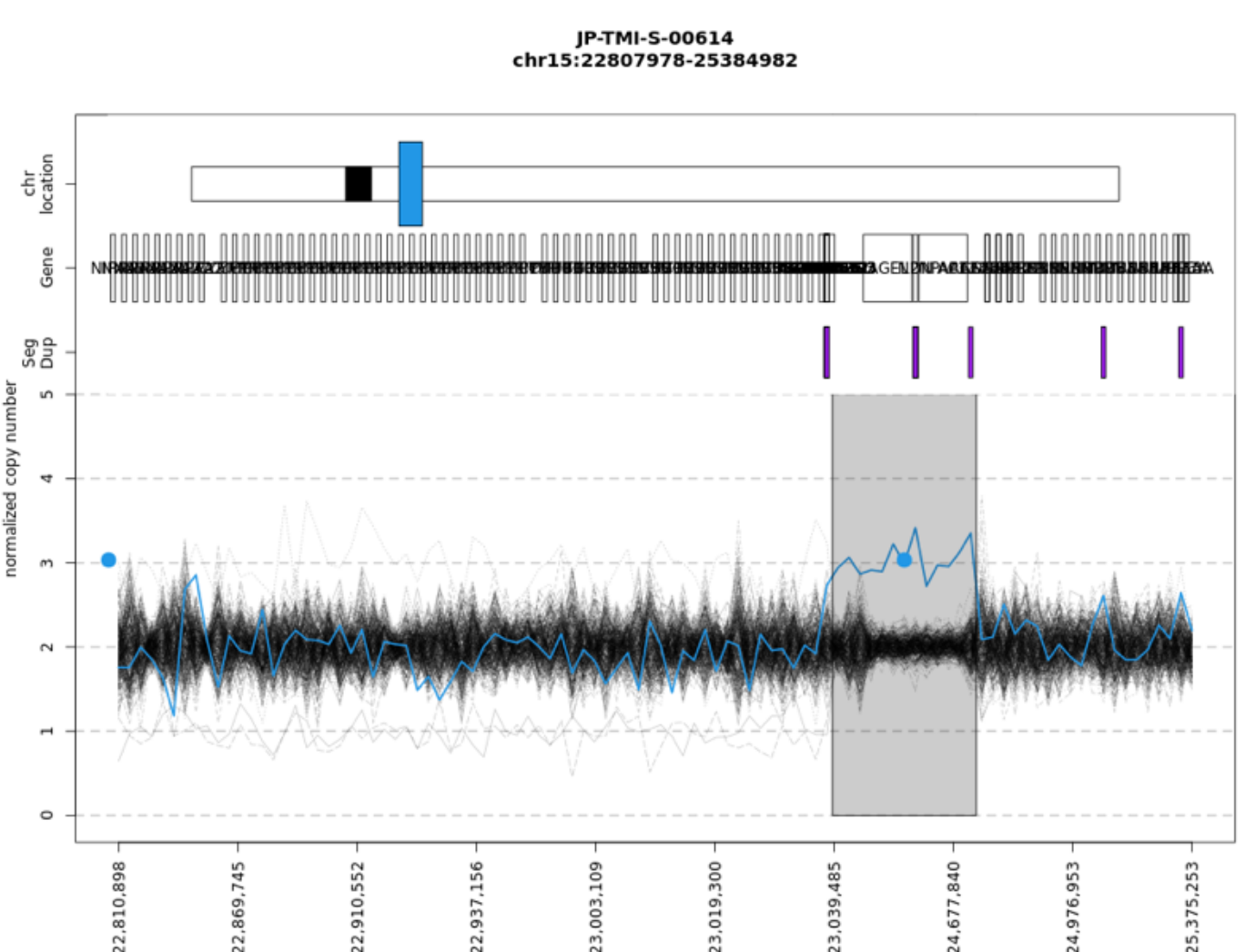
JP-TMI-S-00614
chr15:22807978-25384982
chr location
Gene
Seg Dup
normalized copy number
22,810,898
22,869,745
22,910,552
22,937,156
23,003,109
23,019,300
23,039,485
24,677,840
24,976,953
25,375,253

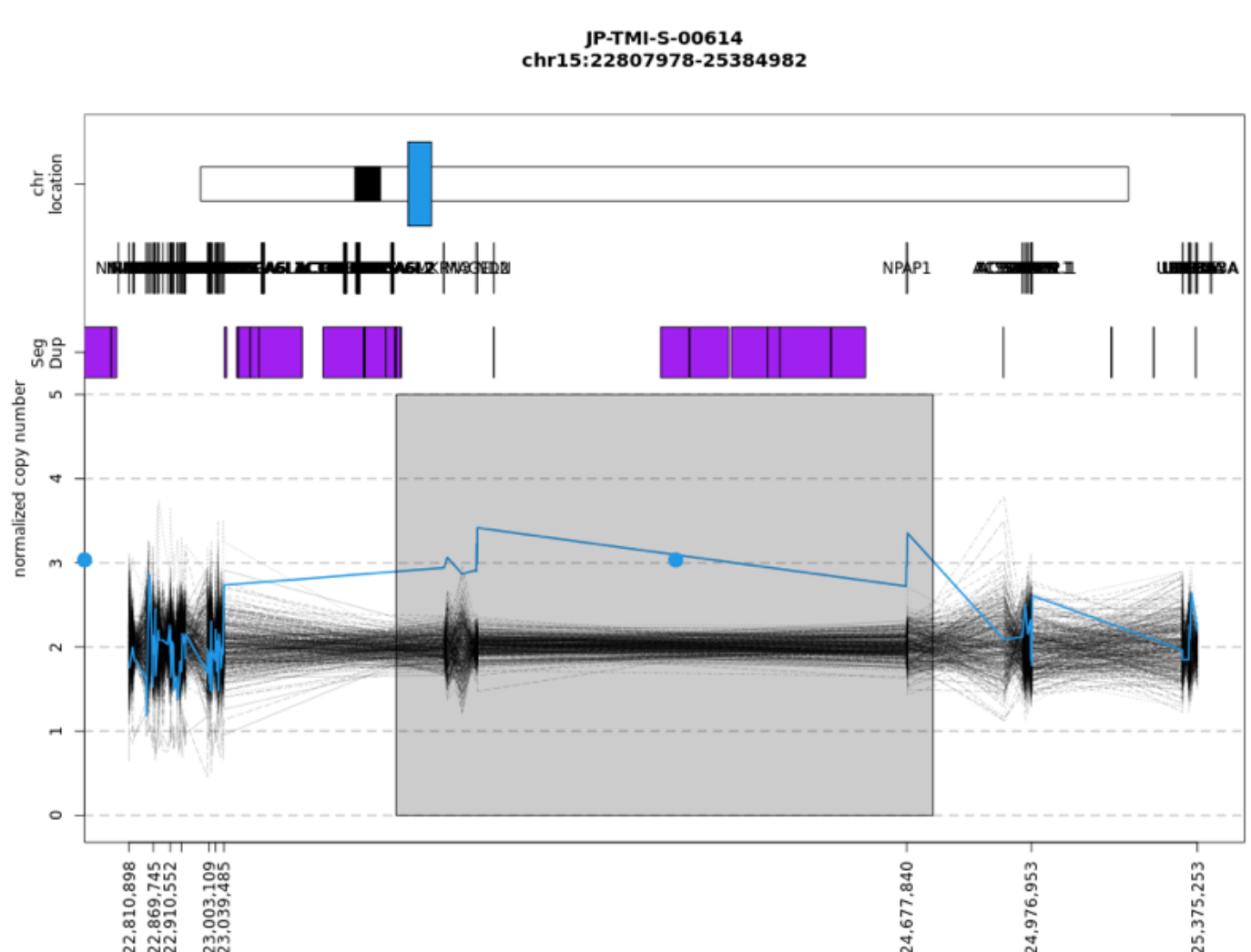
JP-TMI-S-00614
chr15:22807978-25384982
chr location
Gene
Seg Dup
normalized copy number
NPAP1
22,810,898
22,869,745
22,910,552
23,003,109
23,039,485
24,677,840
24,976,953
25,375,253

JP-RIK-S-00587
chr1:146159940-149084732

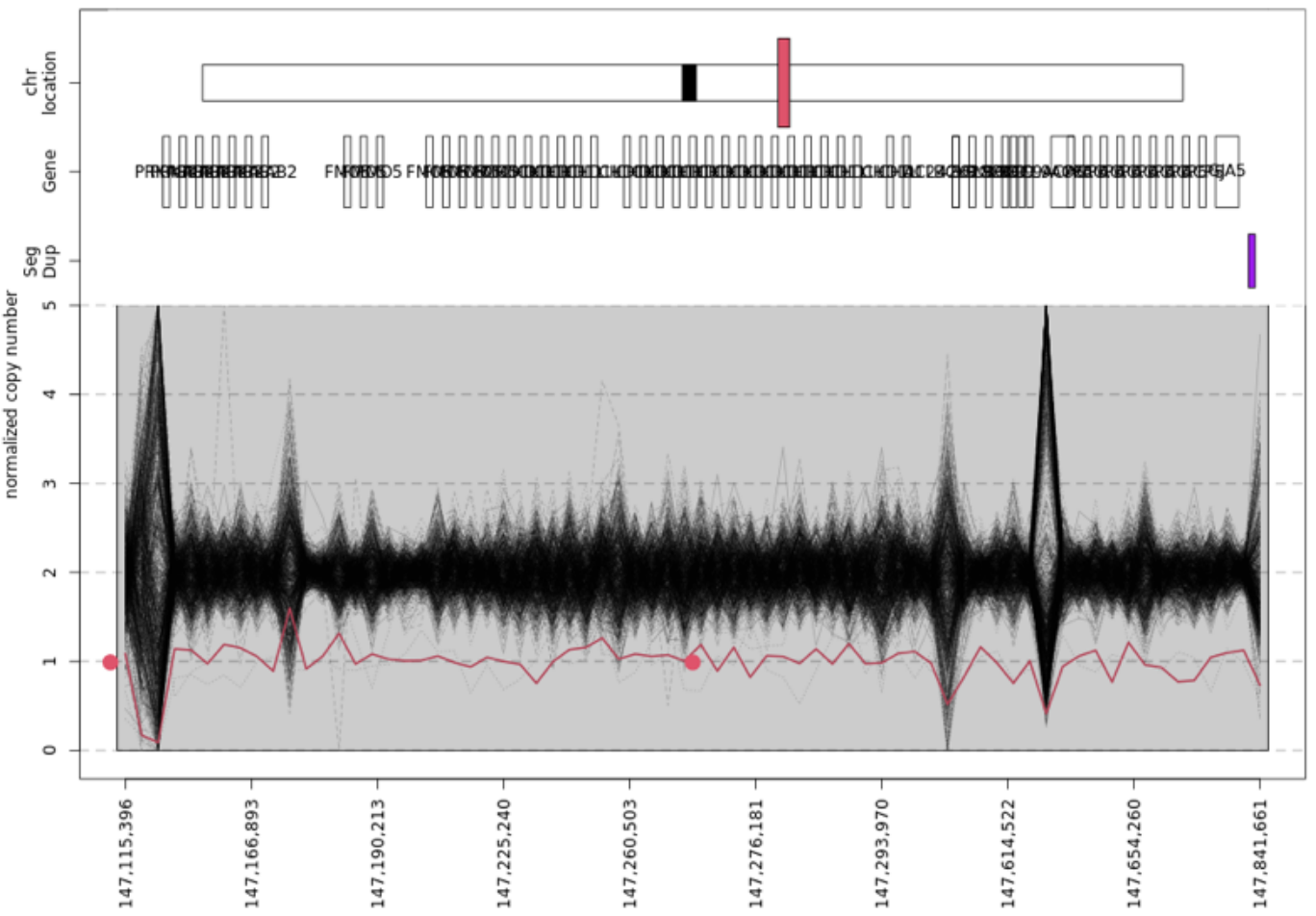

JP-RIK-S-00587
chr1:146159940-149084732

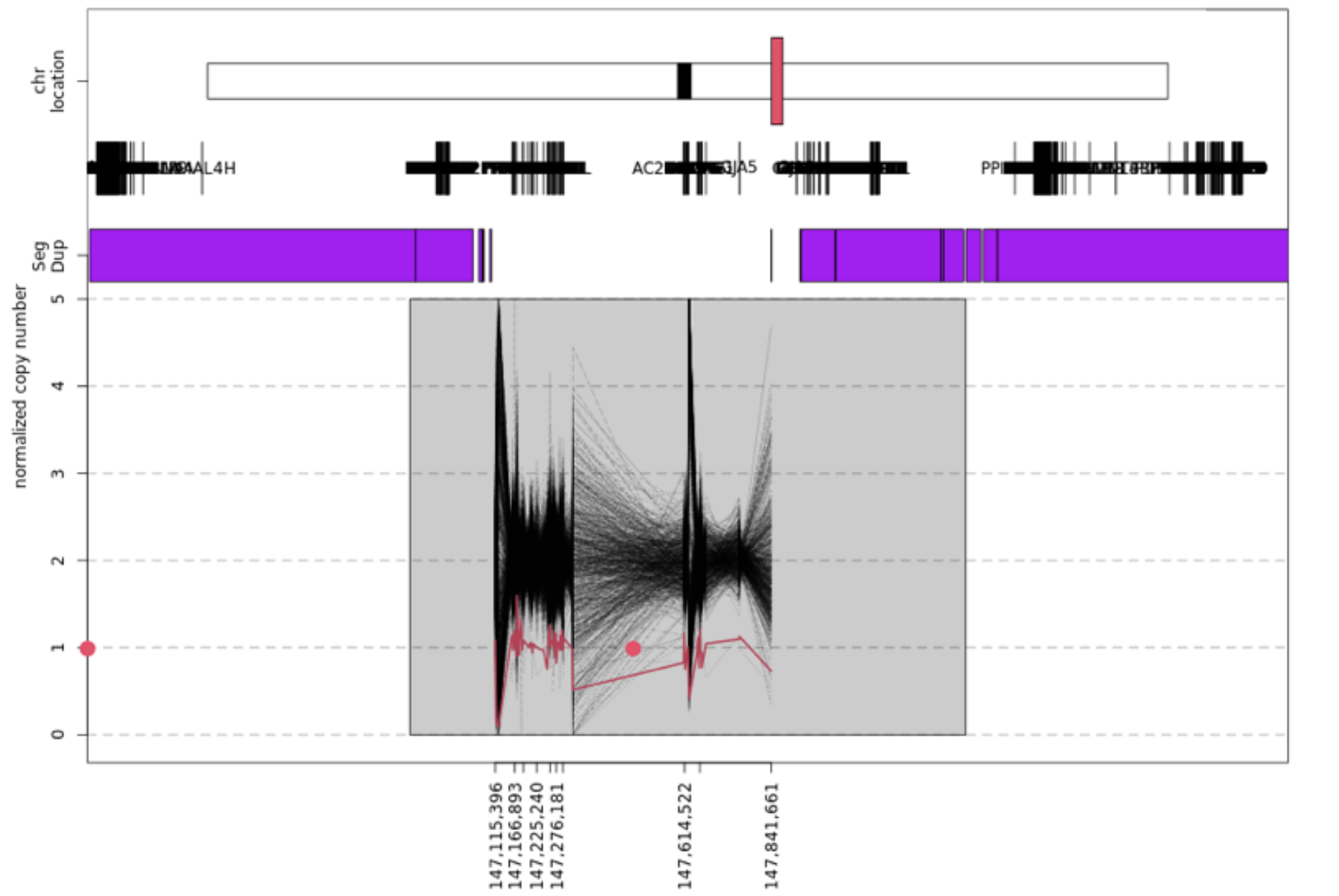

JP-TKU-S-00358
chr15:21253551-30270319

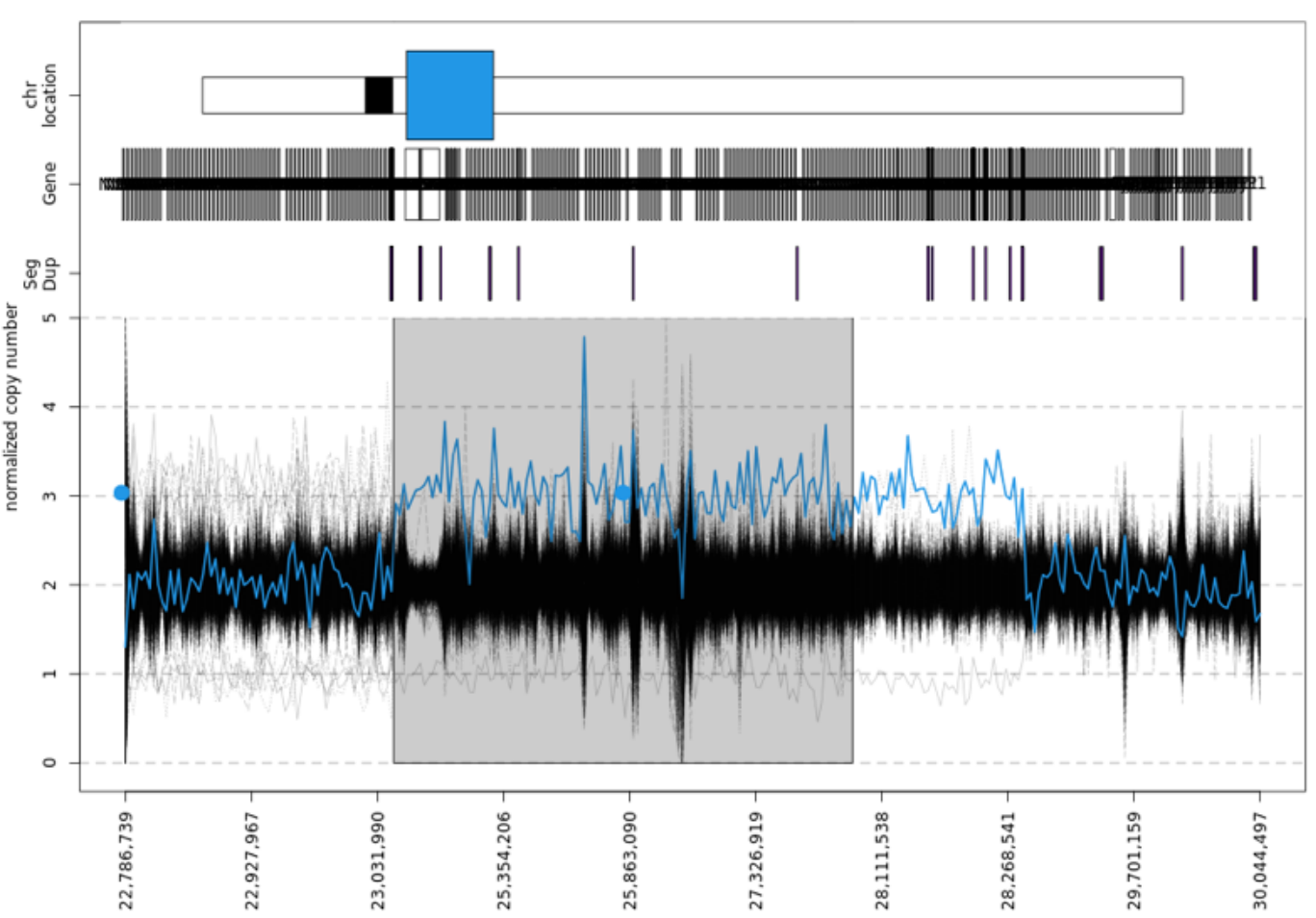

JP-TKU-S-00358
chr15:21253551-30270319

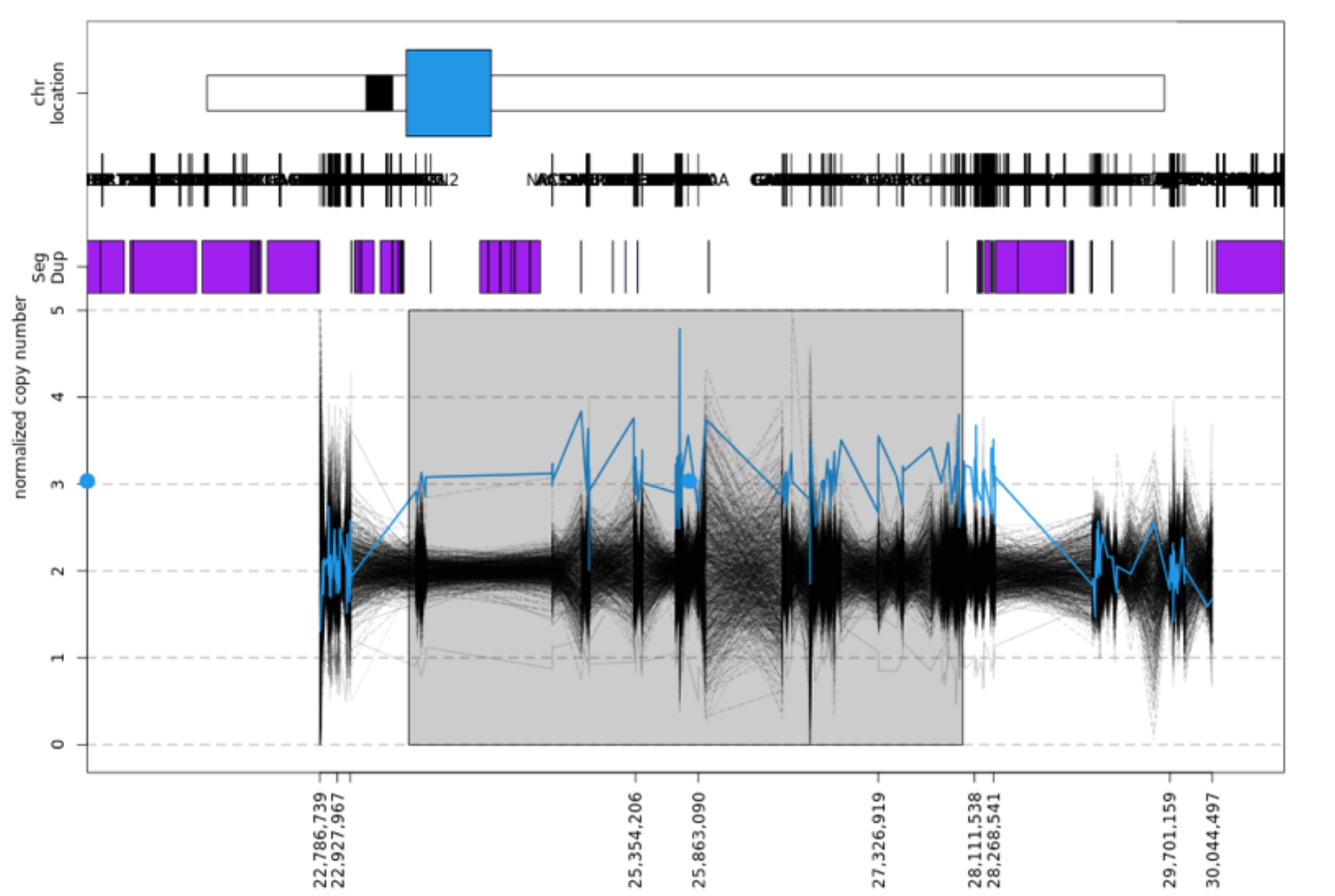

JP-TMI-C-00602
chr15:22807978-25384982

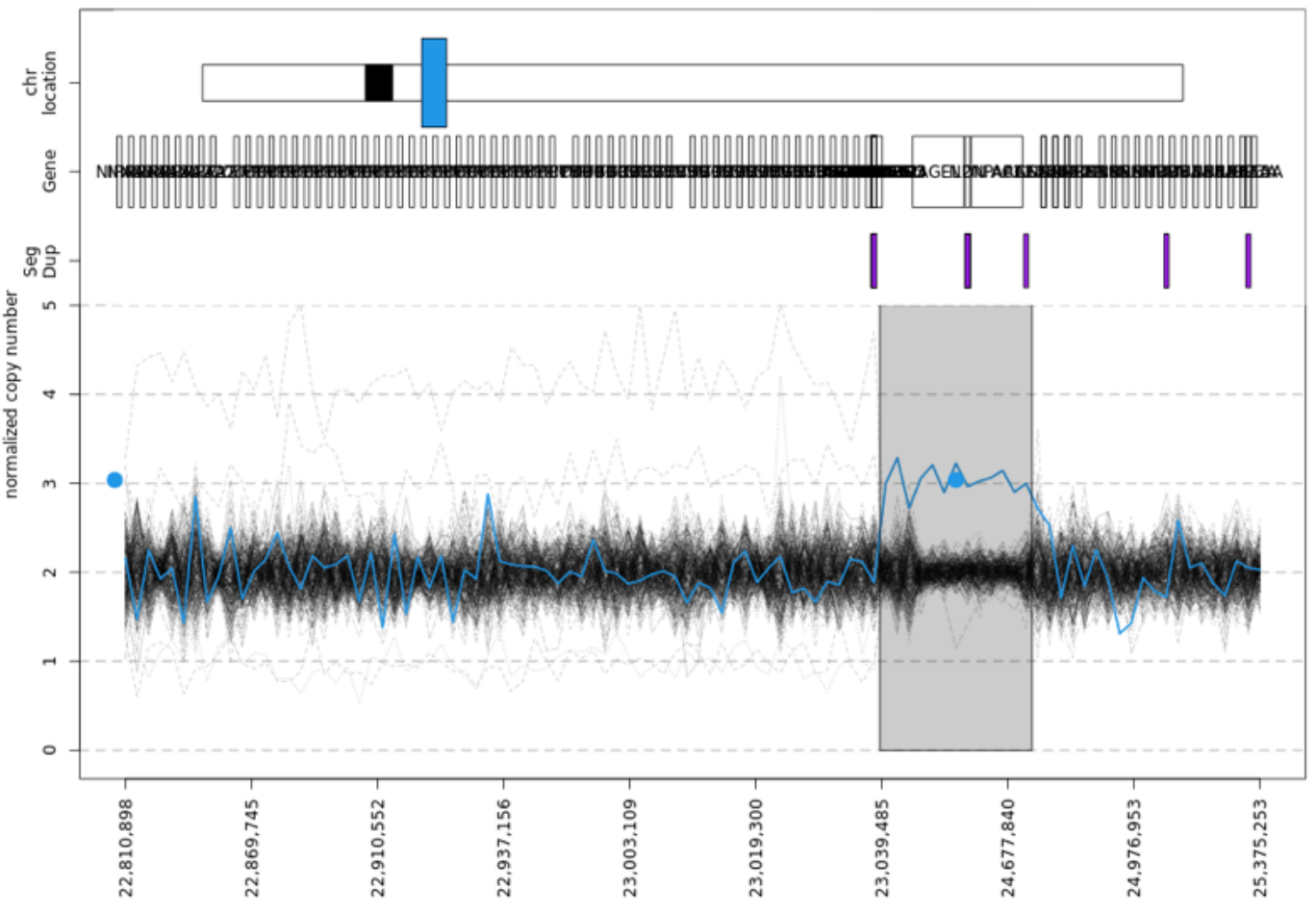

JP-TMI-C-00602
chr15:22807978-25384982

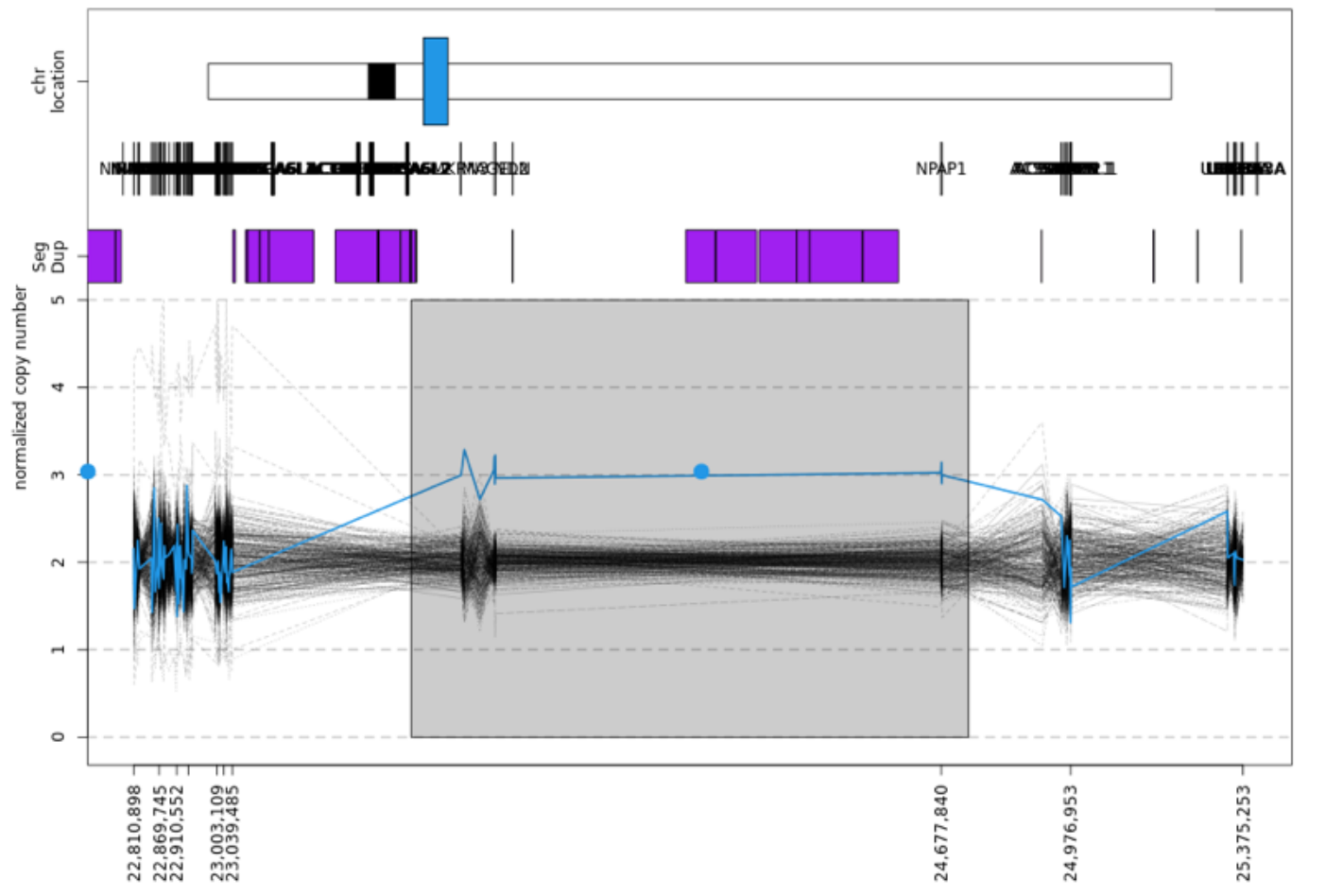

Supplement: Supplementary file 5 [file 41588_2026_2732_MOESM6_ESM.pdf]
